# Transcriptional Profiling of mRNAs and microRNAs in Human Bone Marrow Precursor B Cells Identifies Subset- and Age-Specific Variations

**DOI:** 10.1371/journal.pone.0070721

**Published:** 2013-07-30

**Authors:** Kristin Jensen, Berit Sletbakk Brusletto, Hans Christian Dalsbotten Aass, Ole K. Olstad, Peter Kierulf, Kaare M. Gautvik

**Affiliations:** 1 Department of Medical Biochemistry, Oslo University Hospital, Oslo, Norway; 2 Department of Pediatrics, Oslo University Hospital, Oslo, Norway; 3 Institute of Basic Medical Sciences, University of Oslo, Oslo, Norway; University of Medicine and Dentistry of New Jersey, United States of America

## Abstract

**Background:**

Molecular mechanisms explaining age-related changes in the bone marrow with reduced precursor B cell output are poorly understood.

**Methods:**

We studied the transcriptome of five precursor B cell subsets in individual bone marrow samples from 4 healthy children and 4 adults employing GeneChip® Human Exon 1.0 ST Arrays (Affymetrix®) and TaqMan® Array MicroRNA Cards (Life Technologies™).

**Results:**

A total of 1796 mRNAs (11%) were at least once differentially expressed between the various precursor B cell subsets in either age group (FDR 0.1%, p≤1.13×10^−4^) with more marked cell stage specific differences than those related to age. In contrast, microRNA profiles of the various precursor B cell subsets showed less hierarchical clustering as compared to the corresponding mRNA profiles. However, 17 of the 667 microRNA assays (2.5%) were at least once differentially expressed between the subsets (FDR 10%, p≤0.004). From target analysis (Ingenuity® Systems), functional assignment between postulated interacting mRNAs and microRNAs showed especially association to cellular growth, proliferation and cell cycle regulation. One functional network connected up-regulation of the differentiation inhibitor ID2 mRNA to down-regulation of the hematopoiesis- or cell cycle regulating miR-125b-5p, miR-181a-5p, miR-196a-5p, miR-24-3p and miR-320d in adult PreBII large cells. Noteworthy was also the stage-dependent expression of the growth promoting miR-17-92 cluster, showing a partly inverse trend with age, reaching statistical significance at the PreBII small stage (up 3.1–12.9 fold in children, p = 0.0084–0.0270).

**Conclusions:**

The global mRNA profile is characteristic for each precursor B cell developmental stage and largely similar in children and adults. The microRNA profile is much cell stage specific and not changing much with age. Importantly, however, specific age-dependent differences involving key networks like differentiation and cellular growth may indicate biological divergence and possibly also altered production potential with age.

## Introduction

Access to bone marrow (BM) from healthy children is generally a limiting factor for studies of changes within the human B cell compartment during aging. In contrast to red blood cells, platelets and the myeloid lineage cells, production of the lymphoid lineage is considerably diminished with age both in humans and mice [1], but answers to why and how this happens are still lacking. Almost all present knowledge of age-related transcriptional changes in precursor B cells has been derived from mice, and points to alterations both in key proteins driving the differentiation [2]–[7], and to modification in the supporting microenvironment [8], [9]. So far, only two studies in humans have analyzed global gene expression employing developing precursor B cells from children [10] and adults [11], respectively; neither of the publications includes both age groups. Of increasing interest is also the role of microRNAs (miRs) in hematopoiesis [12], in the immune system in particular [13], and its relation to hematologic malignancy [14]–[16]. However, most reports presently focus on lineage differentiation in murine hematopoietic stem and early progenitor cells [17]–[22], studying the effects of absence or over-expression of specific miRs on B-lineage development, but studies of highly purified human precursor B cell subpopulations are still lacking. We have studied both mRNA and microRNA profiles in sorted precursor B cells subsets from healthy young children and adults, to gain insight into global transcriptional events and miR profiles characteristic for each stage transition. We explored B-lineage enrichment procedures applicable for both children and adults, to achieve sufficient precursor B cell numbers for analyses from individual donors. As the precursor B cell pool is decreasing with age [23], [24], and markedly from about 20 months [24], we chose to compare children of average 18 month to adults of average 50 years in order to capture two windows with very different precursor B cell pool composition, and of clinical relevance.

## Materials and Methods

### Ethics Statement

Written informed consent was obtained from adult participants and from next of kin on behalf of the children involved in this study. The Regional Medical Research Ethics Committee of Eastern Norway specifically approved this study (REK Øst, Accession no. 473–02132). The study was also otherwise performed according to the Norwegian Health Regulations.

### Bone Marrow Samples

We obtained BM samples from 4 healthy children age 18±2 month (mean ± range) and 4 healthy adults age 50±5 years (mean ± range), all ethnically Norwegian individuals. The children were eligible for minor surgery, the adults for elective orthopaedic surgery. None of the individuals received immunosuppressant treatment. Both groups were haematologically healthy. BM was aspirated from the anterior iliac crest/anterior superior iliac spine using syringes containing 1 ml of 5000 IE/ml heparin (2×10 ml syringes children, 6×20 ml syringes adults).

### Isolation of CD10 Positive Cells

The CD10 marker was used for the isolation of B lineage cells as this marker is expressed by all precursor B cells in the BM [11], [25], but not in mature B cells in peripheral blood. The BM samples were diluted in PBS pH 7,4 (Gibco by Life Technologies) with 2% Fetal Bovine Serum (Life Technologies, USA) filtered at 70 µm (BD Biosciences Falcon Cell Strainer 70 um Nylon Cat. no. 352350) and subjected to Ficoll-Paque™ PREMIUM (GE Healthcare, USA) density-gradient centrifugation. CD10^+^ precursor B cells were positively selected using streptavidin coated Dynabeads® FlowComp™ Flexi (Invitrogen Dynal AS, Oslo, Norway) and CD10 antibody (Cat. no. 34199-100, clone SN5c, Abcam Inc. Cambridge, MA, USA) labeled with DSB-X™ Biotin (Molecular Probes Europe BV, Netherlands). The optimal amount of CD10 antibody used per 100×10^6^ mononuclear cells (MNCs) was titrated individually, and mean amount was for the children 26 µg and for the adults 10 µg.

### Immunolabelling, Flow Cytometry and Sorting of Precursor B Cells

**Figure 1:**
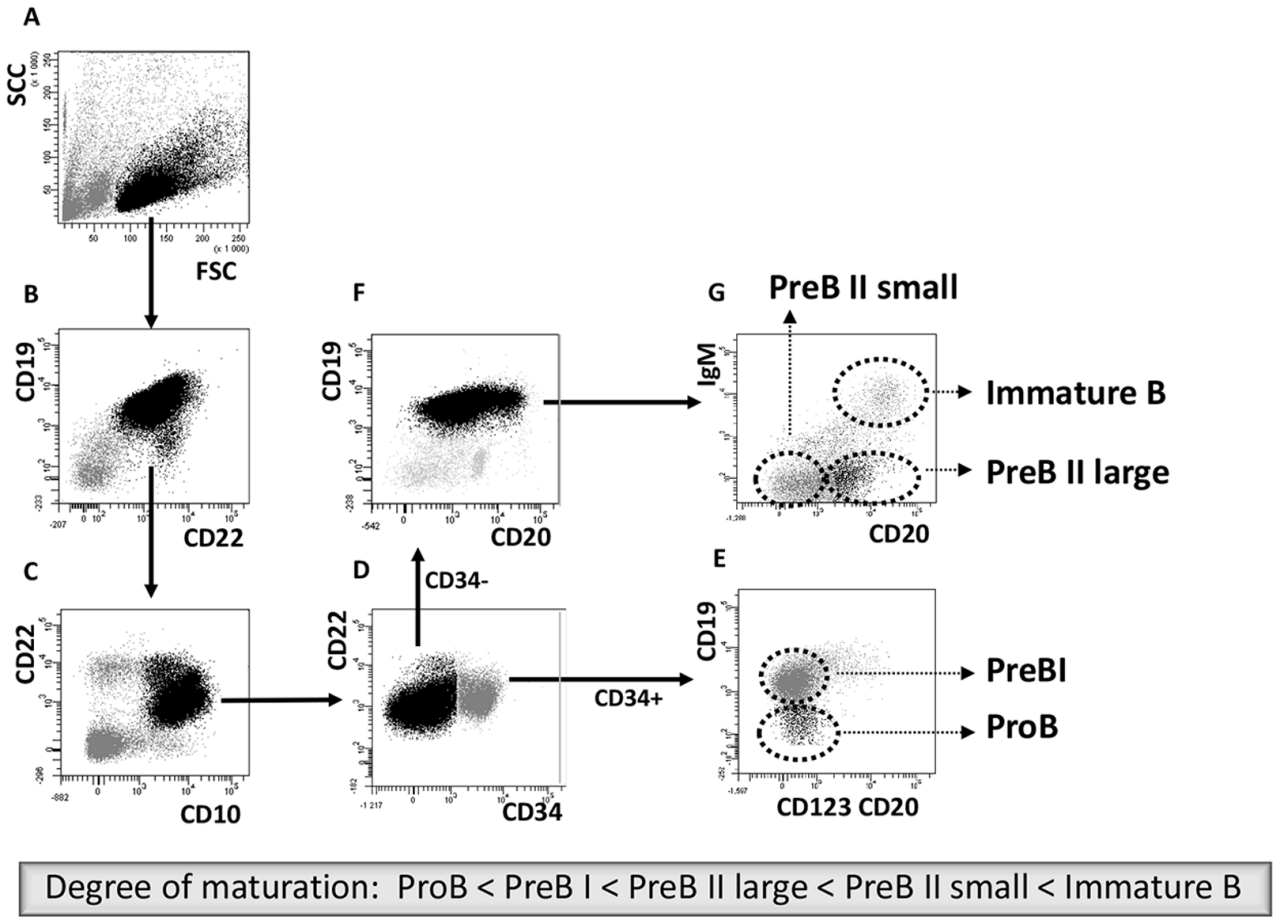
Cell sorting of precursor B cells subsets from CD10 positively selected cells. Immunomagnetic selection and subsequent FACS were used to isolate the five populations from pediatric and adult human BM. Shown are the FACS dot plots with sorting gates to obtain CD34^+^CD19^−^ ProB cells, CD34^+^CD19^+^ PreBI cells, CD34^−^CD19^+^CD20^dim^ PreBII large cells, CD34^−^CD19^+^CD20^−^ PreBII small cells, and CD34^−^CD19^+^CD20^high^IgM^+^ Immature B cells.

CD10 isolated cells were centrifuged, washed and resuspended in 2 Cat.no. 00-4222-57, eBioscience). Cells were immediately stained with 10 µl each of the following antibodies: CD19 APC-AF750 (Cat. no. 27-0199-73, clone HIB19), CD22 APC (Cat. no. 1A-506-T100, clone IS7), CD10 PECy7 (Cat. no. 341112, clone HI10a), CD34-PerCP (Cat. no. 340430, clone 8G12), CD20 PE (Cat. no. 12-0209-73, clone 2H7), CD123 PE (Cat. no. 12-1239-73, clone 6H6), and IgM FITC (Cat. no. 555782, clone G20-127), all from eBioscience, Norway. After incubation on ice for 30 min, 1 ml cold Staining Buffer was added and samples centrifuged at 300×G, 4°C for 10 min. Cell pellets were resuspended in 700 µl Staining Buffer and filtered through a 70µm filter (BD Falcon 5 ml polystyrene round-bottom tube with cell-strainer cap, VWR, Norway) prior to flow cytometric analysis and sorting. In order to avoid loss of valuable cell suspension, instrument compensations were carried out using antibody labeled Anti-Mouse Ig, κ coated beads (BD CompBeads, BD Biosciences, Norway). Five different precursor B cell subsets were isolated on a BD FACSAria™ cell sorter, and events (20.000) further analyzed with the BD FACSDiva™ software, version 5.0.2 (BD, San Jose, CA) (Fig. 1). Cell were displayed in a side and forward scatter dotplot A (linear scale) where the lymphocytes were selected in one gate (black font), followed by identification of CD19 and CD22 positive precursor B cells in dotplot B. These cells were further selected in a CD10 versus CD22 plot (C) to discriminate between contaminating mature blood derived B cells (CD10 negative) and BM precursor B cells (CD10 positive). The cells that expressed CD34 (D) were forwarded into a dotplot CD20-CD123 PE versus CD19. The CD123 marker was included to differentiate precursor B cells, which are all CD123^−^, from CD34^+^ basophilic granulocyte precursors (CD123^+^), which may be included in the lymphogate (dotplot A) [26]. However, these are CD19 negative and were excluded from the ProB sort (E). The presence of CD19 on PreBI cells were used to discriminate them from ProB. The CD34 negative (D) and CD19 positive cells (F) were forwarded into a CD20 versus IgM dotplot. In panel G, Immature B cells were sorted on the basis of CD20^high^ and IgM expression. The remaining two populations, PreB II large and PreB II small were separated based on their CD20 expression. The samples were kept at 4°C and cells were sorted into cold polypropylene tubes (Cat. no. 352063, VWR, Norway) to prevent adherence. The tubes were centrifuged at 300×G, 4°C for 10 min, and pellets with 100 µl supernatant lysed with 700µl QIAzol® Lysis Reagent (www.qiagen.com/handbooks), thoroughly vortexed and stored at –80°C for further mRNA and microRNA isolation. Table S1 shows demographics, number of isolated MNCs and sorted precursor B cell subpopulations from the various donors. FACS purity control was 89% and above.

### RNA Isolation

Total RNA was extracted and purified from each precursor B cell subset using the miRNeasyMini Kit® (Qiagen, Hilden, Germany) and Phase Lock Gel™ Heavy (Cat. no 2302830, 5 PRIME GmbH, Hamburg, Germany) according to the manufactureŕs recommendation. Because of scarcity of material, each total RNA sample was further separated into low molecular weight (**LMW**) RNA ( =  microRNA) and high molecular weight (**HMW**) RNA ( =  mRNA) using Microcon® Centrifugal Filter columns with Ultracel YM-100 membranes (Cat. no. 42413, Millipore, Bedford, Massachusetts, USA) which has a cut-off for single stranded RNA of 300 nucleotides (Fig. S1). The HMW RNA fraction was quantified by NanoDrop® ND-1000 Spectrophotometer, or if too low in concentration, by NanoDrop® ND-3300 Fluorospectrometer (Saveen Werner, Malmø, Sweden) using the RiboGreen® method (Molecular Probes®, Invitrogen detection technologies, Eugene, OR, USA). Sample concentration (A260/A280 ratio) ranged from 3.9 to149.5 ng/µl. Quality was assessed with Agilent 2100 Bioanalyzer® using either the Agilent RNA 6000 Nano Kit or Agilent RNA 6000 Pico Kit (Agilent Technologies, Palo Alto, CA, USA) depending on sample concentration. The RNA integrity number (RIN) had mean value 8.4± SD 0.89 (n = 39) indicating high RNA purity and integrity.

### Amplification of mRNAs for Gene Expression Analysis

The Ovation®Pico WTA System protocol (NuGEN®) was chosen for cDNA synthesis and amplification. This robust and sensitive method utilizes a linear, isothermal amplification of only original transcripts unlike the exponential amplification used by *in vitro* transcription (IVT). For first strand cDNA synthesis 5 ng HMW RNA was utilized with a primer mix containing both poly T sequences and random sequences for whole transcriptome coverage. Following second strand synthesis, the second cDNA strand (sense strand) was used as template for amplification of single-stranded antisense cDNA products homologous to the first strand cDNA utilizing the Ribo-SPIA™ technology (http://nugeninc.virtual.vps-host.net/tasks/sites/nugen/assets/Flash/techanim_ribo_spia.swf). For detailed methods, please see the manufactory protocols (http://www.nugeninc.com/nugen/index.cfm/support/user-guides/). The amplified SPIA cDNA was further purified with the use of QIAGEN MinElute Reaction Cleanup Kit (cat no 28204) which is specifically adapted for use with NuGEN®. The amplified and purified cDNA samples had mean concentration 334 ng/µl (SD 24.6 ng/µl, CV 7.4%) and mean cDNA yield 9.3 µg (SD 0.68 µg). To convert cDNA into sense transcript cDNA (ST-cDNA), the WT-Ovation™ Exon module was applied using 3 µg cDNA as input, with a combination of random primers and DNA polymerase. The resulting dsDNA products were purified with the QIAGEN MinElute Reaction Cleanup Kit (Cat. no. 28204) (Qiagen, Hilden, Germany). The resulting sense strand cDNA was further fragmented and biotinylated using the Encore™ Biotin Module (NuGEN®) and 5 µg input cDNA.

### Microarray Analyses and Statistical Analysis of Data

Amplified, fragmented and biotin-labeled cDNA targets were prepared for analysis on microarrays according to the Affymetrix GeneChip® Expression Analysis Technical Manual (P/N 702232 Rev.2). The solutions were hybridized to GeneChip® Human Exon 1.0 ST Arrays (Affymetrix®, Santa Clara, CA) covering 1 million exons, then washed and stained. The arrays were scanned using the Affymetrix Gene Chip Scanner 3000 7C. The scanned images were processed using the AGCC (Affymetrix GeneChip Command Console) software, and CEL files were imported into Partek® Genomics Suite™ software (Partek, Inc. MO, USA). The Robust Multichip Analysis (RMA) algorithm was applied for generation of signal values and normalization. On each array 21.989 transcripts could be detected. Gene expression was analyzed in core mode (see www.affymetrix.com) using signal values above 22.6 across arrays to filter out low and non-expressed genes, reducing the number to 15.830 transcripts. For expression comparisons of different maturation stages, a one-way ANOVA model was used, and for age group comparisons a modified two-way ANOVA model. Gene lists for mRNA were generated with the criteria of a 0.1% False Discovery Rate (FDR) (p-value ≤1.13×10^−4^) for the various maturation stage comparisons, and 1% FDR (p≤1.13×10^−5^) for the age group comparisons. For direct comparison of successive maturation stages or pairwise comparisons children versus adults, results were expressed as fold change, and gene lists generated with the criteria of fold change ≥ |2| and p-values <0.05. The complete gene expression material is available online at ArrayExpress (http://www.ebi.ac.uk/arrayexpress/) with accession number E-MTAB-1422.

### Validation with Quantitative RT-PCR

Due to limitation of material, only key transcripts could be validated with quantitative RT-PCR (qRT-PCR) using TaqMan® Gene Expression Assays (384-well plates) (Applied Biosystems) The arrays were run on the ViiA™ 7 Real-time PCR System (Applied Biosystems). The relative mRNA expression was calculated with the Comparative Ct method (fold change = 2^−ΔΔCt^) using B2M (Beta 2 microglobulin) as endogenous control [27]. Table S2.

### Quantification of microRNAs

The Megaplex™ Pools for microRNA Expression Analysis were applied for quantification of microRNAs (Applied Biosystems). First, 3 ng starting material (LMW RNA) was used for reverse transcription (RT) with stem-looped RT primers enabling synthesis of cDNA for mature miRNAs. Next, unbiased pre-amplification was performed using gene-specific forward and reverse primers prior to loading onto TaqMan® Array Human MicroRNA A+B Cards Set v3.0 (Cat. no. 4444913, Life Technologies™) for PCR amplification and real time analysis. The arrays were run on a ViiA Real Time PCR system thermocycler (Applied Biosystems®) for accurate quantitation of 667 human microRNAs and three endogenous controls to aid in data normalization+one negative non-human control. U6 snRNA (mammu6) was chosen as endogenous control in our experiments due to least variation. Relative quantitation was then applied using the comparative C_T_ method (ΔΔC_T_) [27]. For expression comparisons of different subsets, profiles were compared using (a) a one-way ANOVA model with 10% FDR (p≤0.004) or (b) fold change with cut-off ≥ |2| and p-values <0.05.

### Ingenuity Pathway Analysis (IPA)

Gene networks and canonical pathways representing key genes were identified through the use of Ingenuity Pathway Analysis, IPA (Ingenuity® Systems, www.ingenuity.com). Briefly, the data sets containing gene identifiers and corresponding fold change and p-values were uploaded into the web-delivered application and each gene identifier was mapped to its corresponding gene object in the Ingenuity Pathway Analysis (IPA) software. Fisher’s exact test was performed to calculate a p-value assigning probability of enrichment to each biological function and canonical pathway within the IPA library.

## Results

### Global Gene Expression Profiling of Precursor B cell Populations

**Figure 2:**
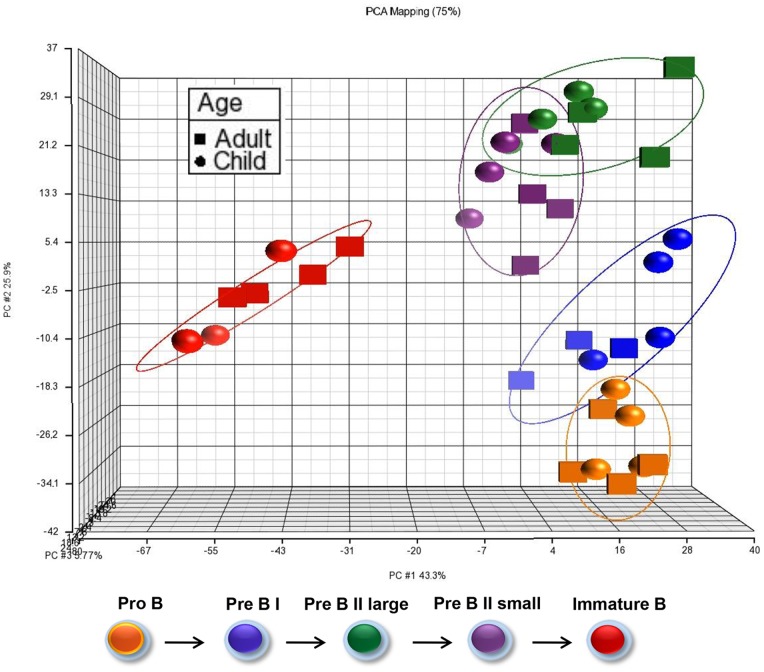
Principle component analysis (PCA) of regulated genes. The clustering represents the overall expression pattern of significantly regulated mRNAs at FDR 0.1% (p-value ≤1.13×10^−4^) in five subsets of precursor B cells. Color codes represent the various maturation stages as indicated under the plot. The Partek® Genomics Suite™ program draws the elipsoids encompassing the individual datapoints. Note the dots for the children (spheres) and adults (angular balls) are tightly grouped together.

Gene expression profiles of the five precursor B cell subsets from BM (Fig. 1) were determined using the GeneChip® Human Exon 1.0 ST Arrays (Affymetrix®), containing 15.830 detectable transcripts after core mode analysis and filtering (see materials and methods). The five subsets were distinguished by the significant differential expression of 1796 genes (11%) that were at least once differentially expressed between the various stages of maturation in either age group (one-way ANOVA, FDR 0.1%, p-value ≤1.13×10^−4^) (Table S3). Hierarchic clustering was performed to group the expression patterns of the five cell subsets. Fig. 2 illustrates the clustering as principle component analysis (PCA) representing the overall expression pattern of each sample. There is a compellingly distinct separation between the cell subsets, and a remarkably similar clustering between children and adults. Moreover, there seems to be a gradual change as the cells progress through the various maturation stages. Therefore, the analysis revealed a stronger variance between subsets than between age groups (see also Fig. S2 for heatmap of differentially expressed genes). See also Table S4 for expression of key B lineage related genes during differentiation of precursors B cells in adult and children. Changes in gene expression during differentiation are highly comparable to the results in earlier studies [10]. See also reports from functional analysis (Ingenuity® Systems) of transcripts changing with differentiation in children (Table S5), with differentiation in adults (Table S6), and with pair-wise comparisons of cells in the same differentiation stages between children and adults (Table S7).

### Functional Analysis of Stage-dependent Differential Gene Expression in Precursor B cells

We further analyzed the 1796 differentially expressed genes through IPA (Ingenuity® Systems). Of these, 1605 genes were associated with bio-functional groups and networks. The gene sets were especially associated with cell cycle, cellular growth and proliferation, cellular development, cell death, and cellular assembly and organization (Table 1). The table also shows the canonical pathways most represented. Notably, the strongest association was with BRCA1 and DNA damage response and cell cycle checkpoint regulation, including phosphatidylinositol 3 kinase (PI3K) signaling cascade – a pathway central in regulation of cell proliferation, growth, differentiation, and survival [28].

**Table 1:** Functional analysis of 1796 differentially expressed mRNAs (FDR 0.1%, p-value≤1.13×10^−4^).

Top Bio Function		
Molecular and cellular function		
Name	p-value	# molecules
Cell Cycle	7.46×10^−45^–1.18×10^−3^	354
Cellular Growth and Differentiation	2.30×10^−27^–7.53×10^−4^	538
Cellular Development	2.20×10^−22^–1.13×10^−3^	409
Cellular Death	9.61×10^−20^–1.14×10^−3^	406
Cellular Assembly and Organization	3.41×10^−19^–1.13×10^−3^	226
**Top Canonical Pathways**		
**Name**	**p-value**	**ratio**
Role of BRCA1 in DNA Damage Response	3.06×10^−11^	25/59 (0.424)
PI3K Signaling in B Lymphocytes	9.46×10^−11^	39/136 (0.287)
Cell Cycle Control of Chromosomal Replication	1.19×10^−08^	15/30 (0.5)
Cell Cycle: G2/M DNA Damage Checkpoint Regulation	1.89×10^−08^	19/49 (0.388)
B cell Receptor Signaling	2.28×10^−08^	38/159 (0.253)

### Age-related Alterations in mRNA Expression Common to all Subset Comparisons

**Figure 3:**
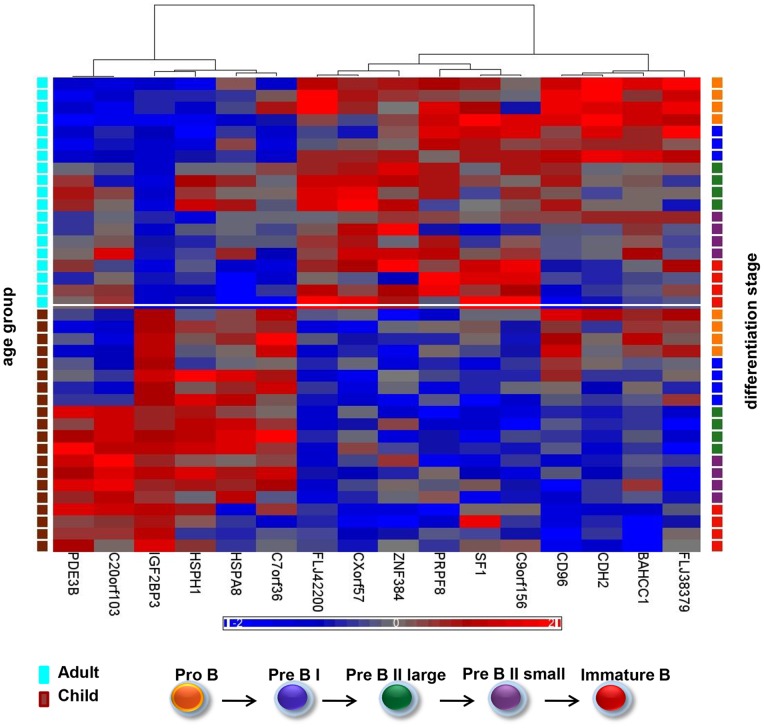
Age-related alterations in gene expression in precursor B cells. Heatmap based on hierarchical clustering of the16 most differentially expressed mRNAs *common* to all stage comparisons in children versus adults (FDR1%, p≤1.13×10^−05^). Six transcripts were higher expressed in all pediatric precursor B cell subsets and ten were generally over-expressed in adults. The color codes indicating differentiation stage (right) and age group (left) are explained below.

Even though the global mRNA profiles showed a striking similarity between children and adults for all subsets (Fig. 2), we reanalyzed the data searching for specific age-related alterations. We then identified differentially expressed genes common to all five pairwise comparisons (ProB children versus adults and PreBI children versus adults etc.) in the two age groups resulting in 16 differentially expressed mRNAs (two-way ANOVA, FDR 1%, p≤1,13×10^−5^) (Fig. 3). Of these, six transcripts were expressed at higher levels in all subsets in children, among them the insulin-like growth factor 2 mRNA binding protein 3, *IGF2BP3* which was 7.2 fold up (p = 1×10^−21^). The other transcripts had fold change less than |2| (Table S8). Ten transcripts were higher expressed in the adult subsets: three of them with fold change more than |2|: the two hypothetical proteins FLJ42200 (2.8 fold up, p = 1.35×10^−10^) and FLJ38379 (2.3 fold up, p = 2.64×10^−6^), and the spliceosome associated transcript PRPF8 (2.2 fold up, p = 1.35×10^−6^).

### Global microRNA Profiles of Precursor B cell Subsets

**Figure 4:**
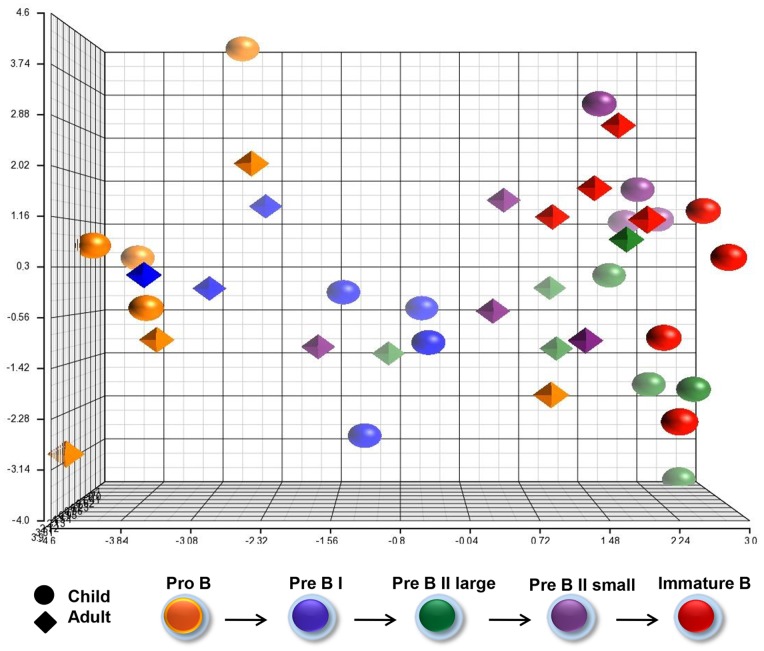
MicroRNA profiles of precursor B cell subsets. Principle component analysis (PCA) showing the overall expression pattern of 17 microRNAs (18 assays) that were at least once differentially expressed between the various subsets (FDR 10%, p≤0,004). The color codes indicating differentiation stage (right) and age group (left) are explained below.

In contrast to the mRNA profiles (Fig. 2), showing distinct subset characteristics, the corresponding microRNA profiles were much more diversely scattered regarding both subset- and age- comparisons (Fig. 4). The results show 17 microRNAs that were at least once differentially expressed between the various stages of maturation (one-way ANOVA, FDR 10%, p≤3.6×10^−3^) (Table S9). Two microRNAs were accompanied by the corresponding star form (Fig. S3); miR-200c/miR-200c* and miR-126/miR-126*; the first pair with opposite and the second pair with similar expression during precursor B cell differentiation.

**Figure 5:**
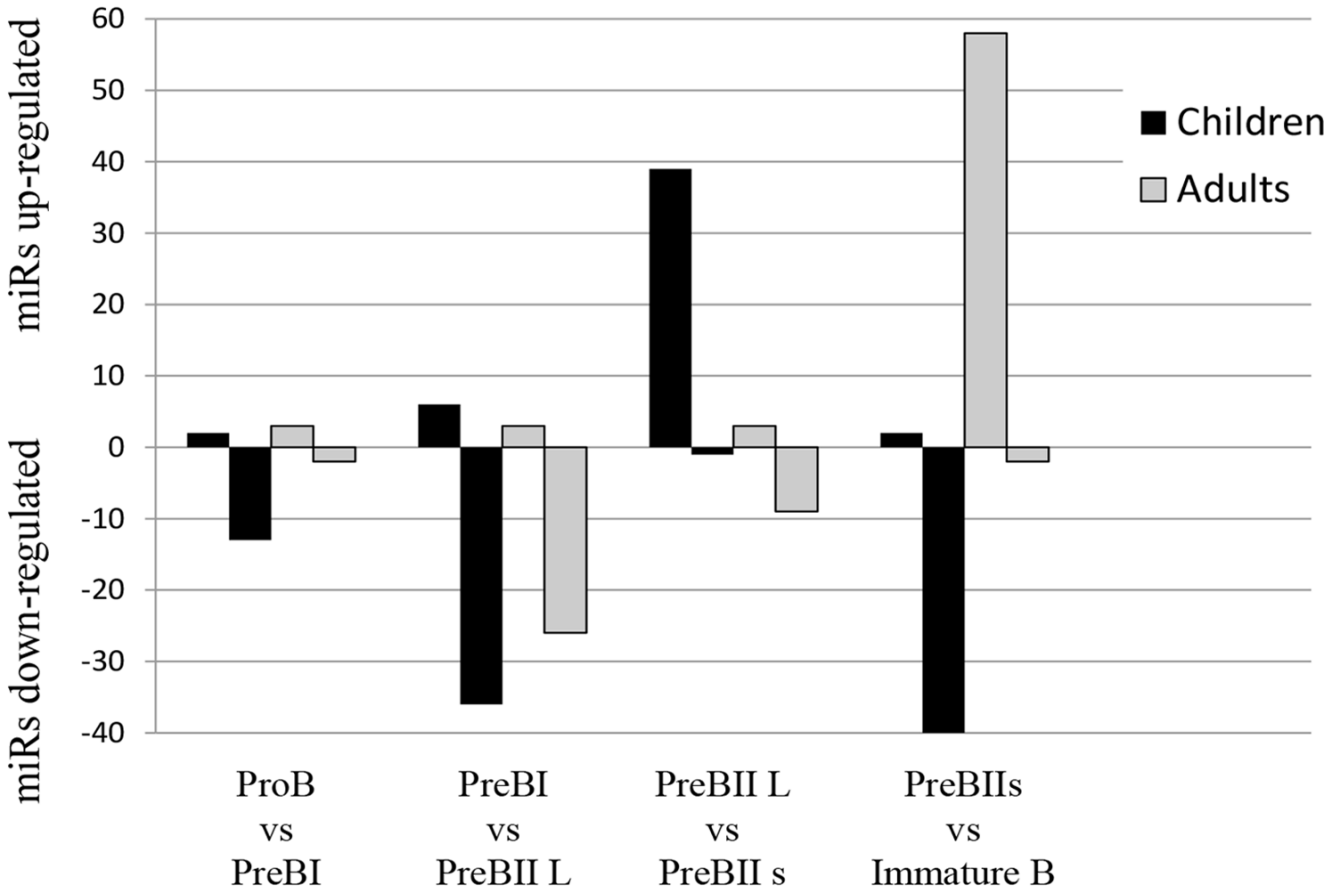
Number of microRNAs up-regulated and down-regulated in each precursor B cell differentiation step in children (black) and adults (grey), respectively. Fold change>|2|, p<0.05.

When comparing each differentiation stage to the successive one, using fold change ≥ |2| and p<0.05 as cut-off, the number of microRNAs changing in each traverse could be compared between children and adults (Fig. 5) (Table S10). For both age groups, few microRNAs increased in the transit ProB to PreBI and PreBI to PreBII large, respectively, and the microRNAs were not the same. During differentiation to PreBII small cells, 39 microRNAs increased in children as compared to 3 in adults (none in common). In the last traverse to Immature B cells, the picture was opposite with 58 microRNAs increasing in adults as opposed to 2 in children (miR-126 in common). Down-regulation of microRNAs was much more prominent than up-regulation in the second transit PreBI to PreBII large in both age groups, and in children in the last transit to Immature B cells. In the first transit to PreBI cells, 13 microRNAs were down-regulated in children as compared to 2 in adults (miR-133b in common). In the successive step to PreBII large cells, 36 microRNAs decreased in children and 26 in adults, respectively. Of these were 13 microRNAs in common and included miR-146a and miR-155, both associated with B-lineage development [16]. The last traverse to Immature B cells involved down-regulation of 40 microRNAs in children as compared to only 2 in adults (miR-126 in common). Thus, the last stage transitions showed marked and opposite differences between children and adults, with a dominant down-regulation of microRNA in pediatric Immature B cells as compared to a prominent up-regulation in adults. See also reports from functional analysis (Ingenuity® Systems) of microRNAs changing with differentiation in children (Table S11), with differentiation in adults (Table S12), and with pair-wise comparisons of cells in the same differentiation stages between children and adults (Table S13).

### Inverse Trend in Expression of the miR-17-92 Cluster in Children and Adults

**Figure 6:**
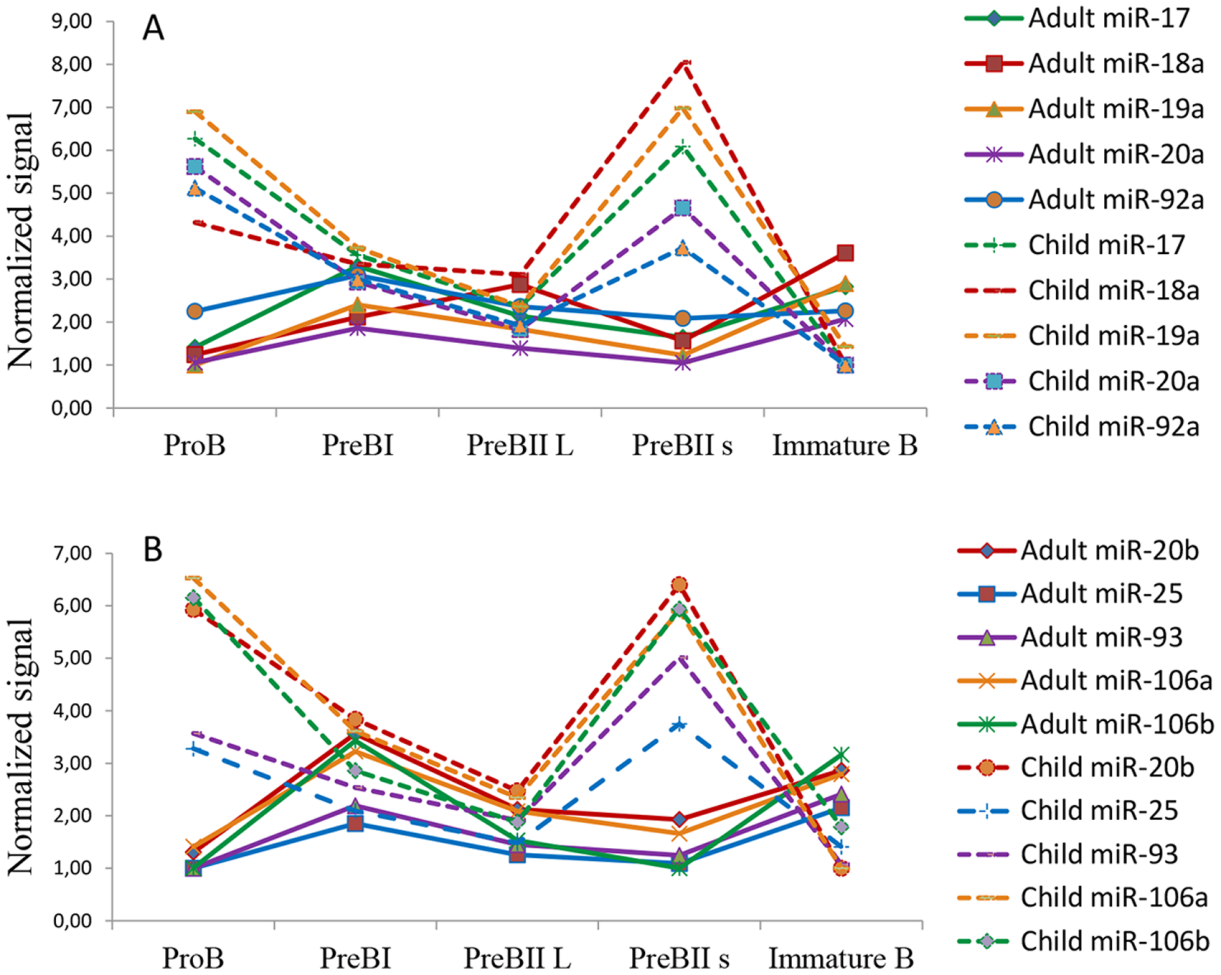
Normalized expression profiles of the (A) miR-17-92 cluster and its (B) paralogs in five precursor B cell subsets in adults (solid lines) and children (dotted lines). All 10 microRNAs followed the same expression profile in both age groups and showed a near inverse pattern with age.

The miR-17-92 cluster and its paralogs [29] are multifunctional clusters of microRNAs known to promote proliferation in hematopoietic tissue in general [30] and in precursor B cells in particular [31]. Comparing mean expression for each cluster member in children and adults, we found a suggestive inverse pattern between the age groups during differentiation (Fig. 6). Notably, the graphs only show trends, as there was high standard deviation within the age groups. At the PreBII small stage, significant age-related differences were found for all miRs (up 3.1–12.9 fold in children, p = 0.0084–0.0270) except miR-92a. Interestingly, five star-form partners were similarly significantly higher expressed in pediatric PreBII small cells as compared to adults (miR-17a*/miR-18a*/miR-19b*/miR-20b*/miR-93*) (up 3–29 fold, p = 0.0018–0.042). Additionally, miR-20b* was higher expressed in pediatric PreBII large cells (up 14.1 fold, p = 0.0106), and miR-18b*/miR-19a*/miR-19b-1*/miR-20b*/miR-25* were higher expressed in adult Immature B cells (up 4.6–17.3 fold, p = 0.0465–0.0093). The expression profiles of known miR-17-92 targets, however, did not show age-related differences (Fig. S4). The mRNAs (E2F1, E2F2, E2F3, PTEN, BCL2L11 and CDKN1A) are all involved in regulation of apoptosis, cell cycle and proliferation.

### Combined Analysis of mRNA and microRNA Expression during Precursor B cell Development

Finally, we searched our database for functional interactions between the differentially expressed mRNAs and microRNAs related to increasing maturation using IPA (Ingenuity® Systems), and selecting only experimentally observed/highly predicted relationships. Not unexpectedly, more or less the same molecular and cellular functional categories were detected for this joint analysis as for the analysis represented by the mRNAs alone such as: cellular growth/proliferation and cell cycle regulation.

**Figure 7:**
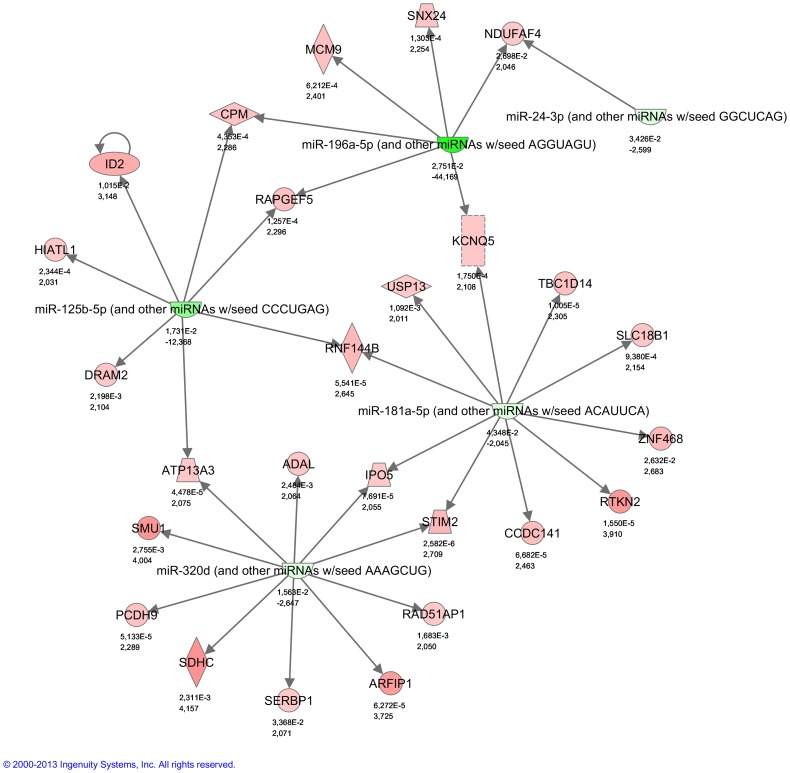
Functional network of up-regulated mRNAs (red) and down-regulated (green) microRNAs during differentiation to PreBII large cells in adults. Note connection of miR-125b-5p to ID2 and involvement of the hematopoiesis related miR-181a-5p and miR-196a-5p, and the cell cycle regulating miR-24-3p. Note, all the coloured interacting partners in this network were detected in the present study.

When each maturation step was analyzed for functional interactions between differentially and inversely expressed mRNAs and microRNAs, the resulting networks, but one, yielded informative value. In adults only, a striking network related to hematopoietic development and function (Fig. 7), connected down-regulation of miR-125b-5p to up-regulation of the differentiation inhibitor ID2 and other mRNAs [32]. Notably, the network also included the hematopoiesis associated miR-181a-5p [17] and miR-196a-5p [33], and the cell cycle associated miR-24-3p [34] and finally miR-320d. The diagram shows a very strong representation of the data generated in the present study.

## Discussion

The ability to isolate and sort small and limited amounts of human precursor B cell subsets from healthy children and adults gives the advantage of studying and comparing physiological changes at the molecular level in regular BM. From young children and adults we were able to obtain sufficient RNA to perform global mRNA and microRNA profiling in five sorted subsets of precursor B cells without pooling the samples.

Somewhat surprisingly, global subset-related mRNA profiles in children and adults were remarkably similar as shown in the PCA plot (Fig. 2), and demonstrated a gradual change along an axis of cell progression towards maturation. Immature B cells had the highest geometric distance to any other subset in agreement with completion of both heavy and light immunoglobulin chain rearrangements [35].

A striking finding was *IGF2BP3*, expressed 7.2 fold higher (p = 1×10^−21^) overall in children as compared to adults. IGF2BP3 (alias IMP3), represses translation of IGF-2 during late embryonic development in mice and humans [36], and has been shown to promote the translation of IGF-2 leader 3 mRNA in a cell model of leukemia [37]. It has also been suggested that the age-related decline in expression of IGF2BP3 is part of widespread genetic reprogramming occurring in many organs simultaneously during postnatal growth [38]. In contrast to peripheral lymphoid tissue and lymphomas [39], IGF2BP3 expression has not been studied previously in precursor B cells from normal pediatric BM, and its physiological role in this tissue remains mainly unknown.

The global microRNA expression profiles, unlike the mRNA signatures, did not distinguish between the five precursor B cell subsets (Fig. 4). However, 17 microRNAs were differentially expressed between the various maturation stages. Among them was the pair miR-126/miR-126*, shown to regulate the transcription factor HOXA9 [40]; playing a role in normal and malignant hematopoiesis [22], [40]–[42]. In our samples, HOXA9 was gradually down-regulated in parallel with miR-126/miR-126* during differentiation in both age groups in agreement with observations in murine hematopoietic stem cells (HSCs) [40]. The functional implication for differentiation of B-lineage cells is, however, presently unknown.

We could only partly confirm the stage-specific profiles of six B cell associated microRNAs as recently reported from murine B2 B-lineage cells [31], which are regarded as equivalent to human B-lineage cells. The authors [31] applied deep sequencing of small RNA libraries generated from pooled murine BM or spleen cells, sorted into ten stages of B2-lineage cells and B1 B cells. Notably, the material used for the generation of the sequence libraries was obtained during a single sort, which is a caveat as pointed out by the authors [31]. Also we found several stage-specific trends; not all of them statistically significant, pointing to the need for verification in larger studies.

Interesting were also our analyses of the miR-17-92 cluster and its paralogs (Fig. 6), showing a very different profile for children and adults. Behind the graphs in Fig. 6, showing mean expression for each miR, there was considerable individual variation, except at the PreBII small stage, where the expression was significantly higher in children. Still, the consistent expression pattern for all miR-17-92 cluster members in children and adults, respectively, is intriguing. Moreover, several star-form species (miRs*), representing the less abundant strand of the hairpin pre-miR structure [43], were co-expressed with the dominant strand and differentially expressed at the PreBII large, PreBII small and Immature B cell stage. Ventura *et al* [44] has shown that absence of miR-17-92 inhibits B cell development at the ProB to PreB cell transition in mice, and causes increased apoptosis at this transit. Xiao *et al* [45] studied miR-17-92 gain-of-function and found that modest over-expression resulted in premature death of transgenic animals associated with lymphoproliferative disease and autoimmunity. The implications of our findings in humans clearly remain to be explored further. Functional analysis of this cluster combined with mRNAs changing inversely, did not yield any more information, and expression of known miR-17-92 targets did not change with age.

Finally, functional analysis of the combined lists of mRNAs and microRNAs changing during precursor B cell differentiation, revealed enrichment for cellular growth, proliferation and cell cycle. Cell cycle regulation was among top canonical pathways indicating that this function differed significantly among the various maturation stages and/or age groups. Indeed, a detailed analysis of the differentiation step from PreBI to PreBII large cells, revealed that only in adults, there was enrichment for cell cycle, DNA replication, recombination and repair, indicating activation of different transcriptional programs in children and adults in this material. Interestingly, this analysis generated in adult PreBII large cells, an extensive network connecting up-regulation of the differentiation inhibitor, ID2 and other mRNAs to decreased expression of several miRs known to be involved in hematopoiesis and cell cycle checkpoint control.

Recently, e.g. a causal relationship has been described [15] linking down-regulation of the DLEU2/miR-15a/16-1 cluster to chronic lymphocytic leukemia, the most common B cell-derived malignancy of adults. Of interest, miR-15a and miR-16-1* were 5.1 fold (p = 0.0070) and 4.0 fold (p<0.05) higher expressed, respectively, in pediatric PreBII small cells as compared to adults. Likewise, up-regulation of miR-210 has been associated with acute lymphocytic leukemia [14], the most common malignancy in children, and was 563 fold up (p = 0.0016) in pediatric PreBII small cells as compared to the adult counterpart.

In conclusion, mRNAs and microRNAs in five human precursor B cell subsets, show major differences in age- and stage-dependent profiles. They connect in functional molecular networks, involving apoptosis, cell cycle regulation and proliferation, with a major representation of molecular partners detected in this study.

## Supporting Information

Figure S1 **Workflow for the mRNA and microRNA isolation.** The flow chart shows the steps followed to isolate both mRNA and microRNA from each of five precursor B cell subsets from single individuals.
(TIF)

Figure S2 **Heatmap of 1796 differentially expressed genes comparing** **five precursor B cell subsets** **from both children and adults** (FDR 0.1%, p-value ≤1.13×10^−4^). Differentiation stage is indicated at the right of the figure with increasing maturation from bottom to top. Age is indicated at the left (children blue, adults brown). Note the similar pattern with age.
(TIF)

Figure S3 **Heatmap of 17 differentially expressed microRNAs comparing** **five precursor B cell subsets** **from both children and adults** (FDR 10%, p≤3.6×10^−3^). Note that the microRNAs pairs miR-200c/miR-200c* and miR-126/miR-126* were accompanied by the corresponding star form; the first pair with opposite and the second pair with similar expression during precursor B cell differentiation.
(TIF)

Figure S4 **The expression profiles of known miR-17-92 targets.** None of the mRNAs showed age-related statistically significant differences.
(TIF)

Table S1 (PDF)

Table S2 (PDF)

Table S3 (PDF)

Table S4 (PDF)

Table S5 (PDF)

Table S6 (PDF)

Table S7 (PDF)

Table S8 (PDF)

Table S9 (PDF)

Table S10 (PDF)

Table S11 (PDF)

Table S12 (PDF)

Table S13 (PDF)
