# Supplementary figures and images for: Transcriptional Profiling of mRNAs and microRNAs in Human Bone Marrow Precursor B Cells Identifies Subset- and Age-Specific Variations
Source: PLoS One. 2013 Jul 30;8(7):e70721. doi: 10.1371/journal.pone.0070721 (PMC3728296; doi:10.1371/journal.pone.0070721)

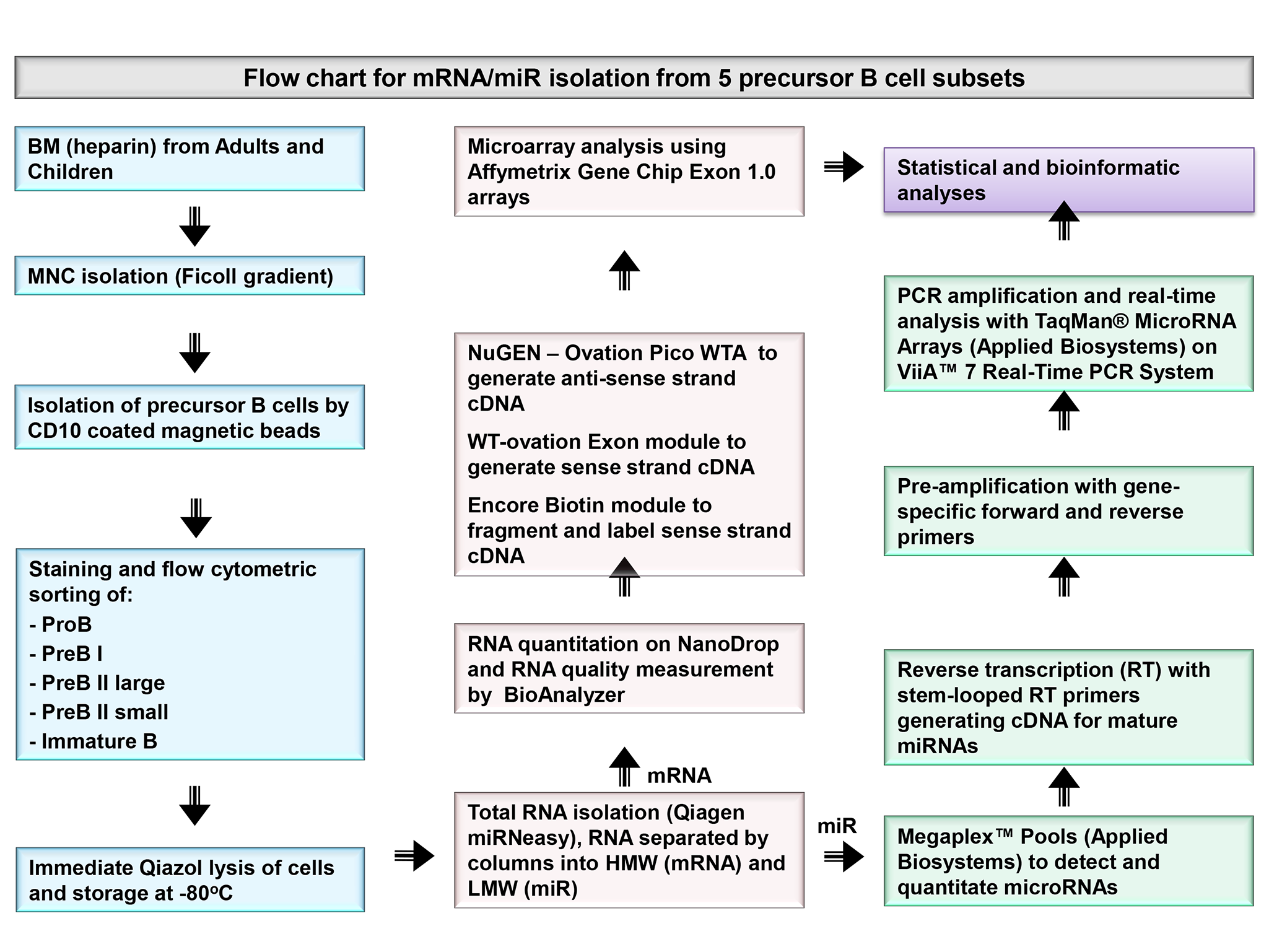

Supplement: Figure S1 — Workflow for the mRNA and microRNA isolation. The flow chart shows the steps followed to isolate both mRNA and microRNA from each of five precursor B cell subsets from single individuals. (TIF) [file pone.0070721.s001.tif]

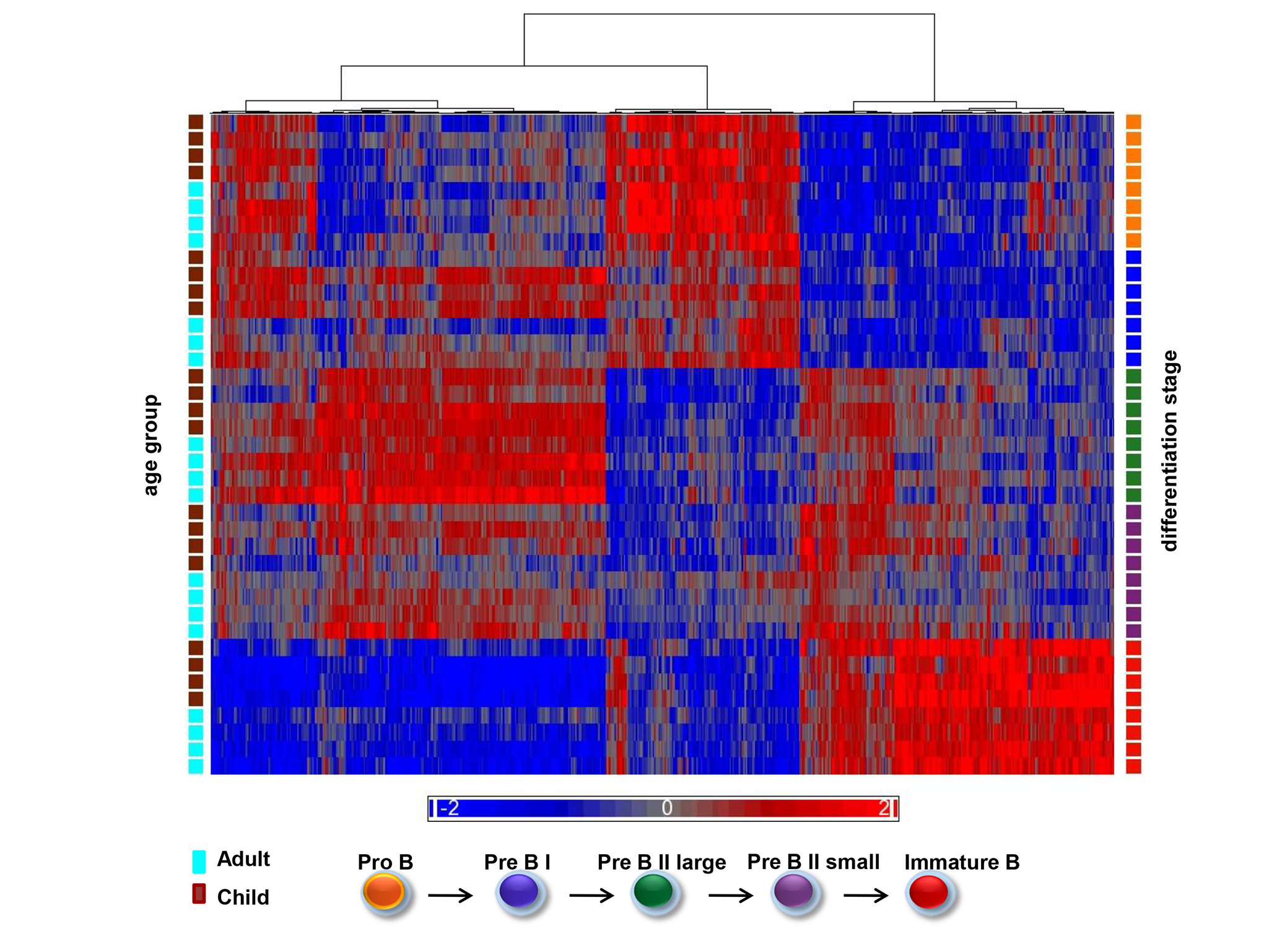

Supplement: Figure S2 — Heatmap of 1796 differentially expressed genes comparing five precursor B cell subsets from both children and adults (FDR 0.1%, p-value ≤1.13×10^−4^). Differentiation stage is indicated at the right of the figure with increasing maturation from bottom to top. Age is indicated at the left (children blue, adults brown). Note the similar pattern with age. (TIF) [file pone.0070721.s002.tif]

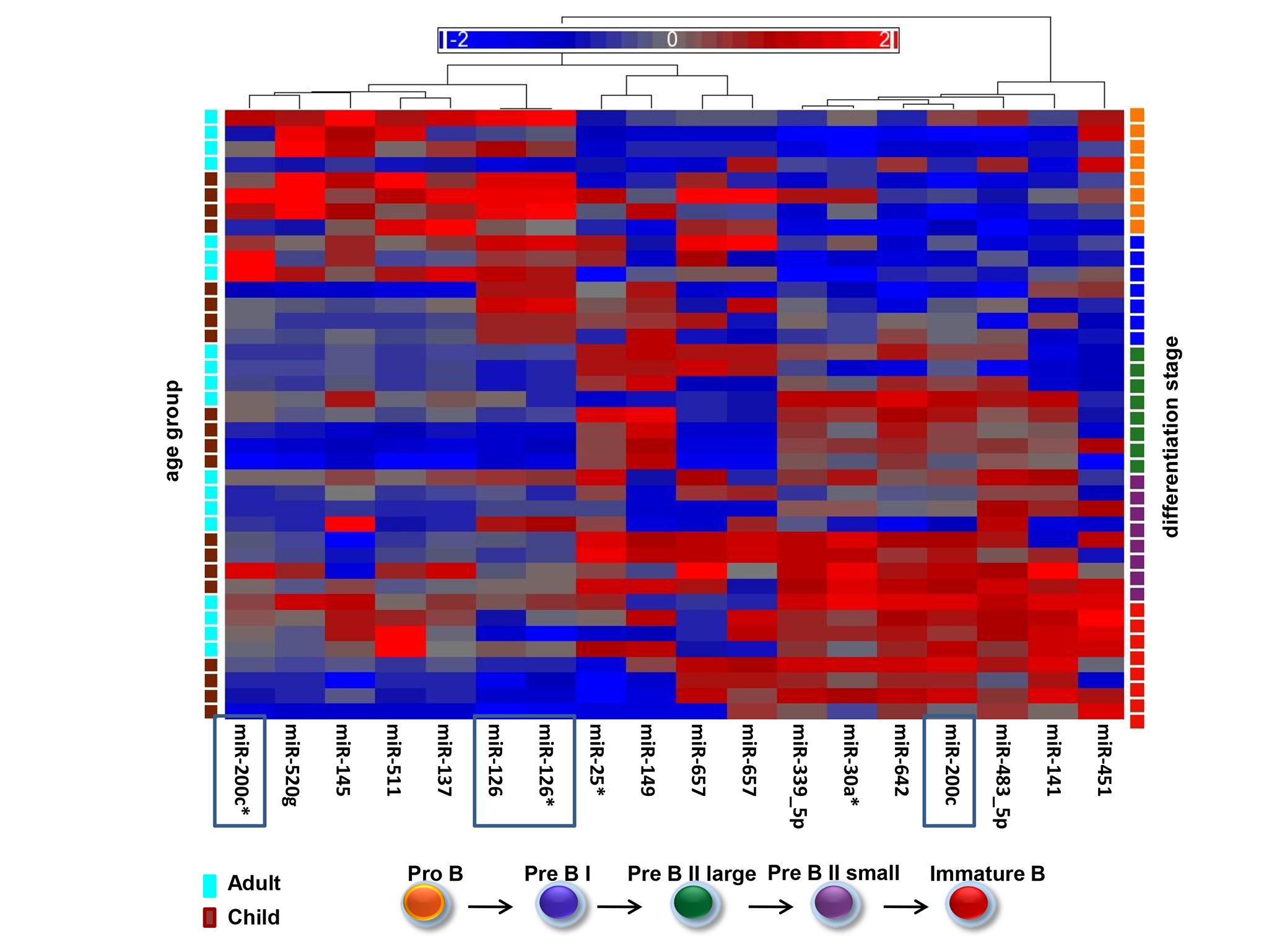

Supplement: Figure S3 — Heatmap of 17 differentially expressed microRNAs comparing five precursor B cell subsets from both children and adults (FDR 10%, p≤3.6×10^−3^). Note that the microRNAs pairs miR-200c/miR-200c* and miR-126/miR-126* were accompanied by the corresponding star form; the first pair with opposite and the second pair with similar expression during precursor B cell differentiation. (TIF) [file pone.0070721.s003.tif]

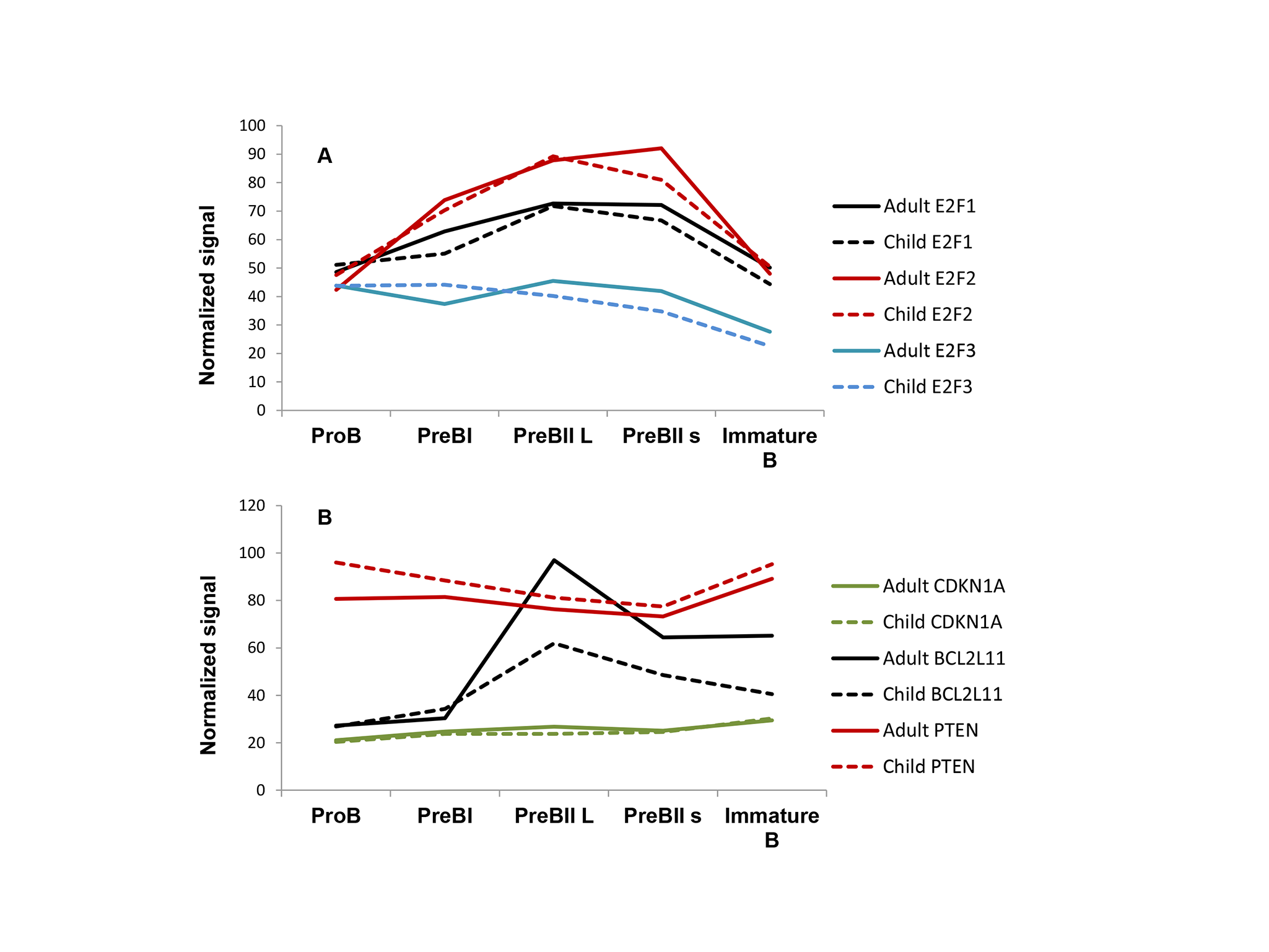

Supplement: Figure S4 — The expression profiles of known miR-17-92 targets. None of the mRNAs showed age-related statistically significant differences. (TIF) [file pone.0070721.s004.tif]
