# Supplementary material for: Transcriptional Profiling of mRNAs and microRNAs in Human Bone Marrow Precursor B Cells Identifies Subset- and Age-Specific Variations
Source: PLoS One. 2013 Jul 30;8(7):e70721. doi: 10.1371/journal.pone.0070721 (PMC3728296; doi:10.1371/journal.pone.0070721)
Supplement: Table S1 — (PDF) [file pone.0070721.s005.pdf]

Supplementary table I. Demografics and number of isolated mononuclear cells (MNCs) and sorted precursor B cell subpopulations from adults and children

			Total number of flow sorted cells				
**Adults**	Age	Mononuclear cells	ProB	PreB I	PreB II L	PreB II s	Immature B
Man	56 years	164×10^6	211.864	Not sorted	105.397	178.638	59.416
Woman	54 years	156×10^6	13.025	50.685	80.346	109.728	51.857
Man	44 years	271×10^6	122.980	300.282	137.873	514.619	77.590
Woman	48 years	710×10^6	54.332	200.000	76.623	285.987	79.440
**Average cell number adults**		**325×10^6**	100.550	183.656	100.060	272.243	67.076
**Children**	Age	Mononuclear cells	ProB	PreB I	PreB II L	PreB II s	Immature B
Boy	17 months	130×10^6	99.057	564.438	344.197	498.562	278.970
Boy	15 months	170×10^6	79.582	585.525	708.309	785.014	625.249
Boy	19 months	186×10^6	534.262	165.971	1315.686	794.081	733.146
Boy	16 months	168×10^6	1057.960	192.031	772.734	522.146	435.663
**Average cell number children**		**164×10^6**	442.715	376.991	785.232	649.951	518.257
