# Supplementary material for: Transcriptional Profiling of mRNAs and microRNAs in Human Bone Marrow Precursor B Cells Identifies Subset- and Age-Specific Variations
Source: PLoS One. 2013 Jul 30;8(7):e70721. doi: 10.1371/journal.pone.0070721 (PMC3728296; doi:10.1371/journal.pone.0070721)
Supplement: Table S2 — (PDF) [file pone.0070721.s006.pdf]

PCR validation of key transcripts - Adults versus children

	EBF1			E2A		
	ΔΔCT	FC	unpaired t-test	ΔΔCT	FC	unpaired t-test
**ProB**	-0,160	1,117081	0,287691203	-3,315	9,95	0,063895773
**PreB I**	-0,036	1,02554	0,454349715	-1,034	2,05	0,189728278
**PreBII L**	-0,010	1,006929	0,489627348	0,619	-1,54	0,162682265
**PreBII s**	0,341	-1,26628	0,06819044	0,417	-1,34	0,341694815
**Immature B**	-0,275	1,209696	0,243698257	-1,563	2,95	0,096114318

	ID2			IGF2BP3		
	ΔΔCT	FC	unpaired t-test	ΔΔCT	FC	unpaired t-test
**ProB**	-3,954	15,49293	0,011339245	3,589	-12,0317	#DIV/0!
**PreB I**	-1,593	3,01672	0,219537517	6,050	-66,2468	#DIV/0!
**PreBII L**	-1,069	2,098089	0,0948071	7,920	-242,15	#DIV/0!
**PreBII s**	0,344	-1,26904	0,369699669	(no samples left for validation)		
**Immature B**	-1,726	3,308362	0,031911201	2,793	-6,93128	0,005623228

	RAG2		
	ΔΔCT	FC	unpaired t-test
**ProB**	0,031	-1,02168	0,48267539
**PreB I**	-0,350	1,274267	0,248197313
**PreBII L**	1,528	-2,88371	0,113192279
**PreBII s**	0,762	-1,69565	0,177676945
**Immature B**	-1,339	2,529209	0,052066473

PCR validation of key transcripts - Differentiation

Absolute mean values

**EBF1**	ProB	PreBI	PreB II L	PreB II s	Immature B
Adults	0,06381	0,059691	0,046036162	0,043222462	0,035455
Children	0,057197	0,059151	0,04583172	0,053846005	0,028873

**E2A**	ProB	PreBI	PreB II L	PreB II s	Immature B
Adults	0,001691	0,000902	0,000839755	0,001652827	0,000582
Children	0,00017	0,00057	0,001462974	0,002370764	0,000183

**IGF2BP3**	ProB	PreBI	PreB II L	PreB II s	Immature B
Adults	0,000281	7,36E-05	1,20978E-05		0,000379
Children	0,00381	0,005117	0,003097705	0,00307267	0,00238

**ID2**	ProB	PreBI	PreB II L	PreB II s	Immature B
Adults	0,001599	0,000729	0,002665233	0,00093904	0,000274

Children	0,000173	0,000468	0,001148164	0,000903971	7,8E-05

**RAG2**	ProB	PreBI	PreB II L	PreB II s	Immature B
Adults	0,003667	0,003302	0,001631444	0,002863117	0,00036
Children	0,003551	0,002884	0,002998612	0,005378109	0,00012

Normalized values to lowest signal

**EBF1**	ProB	PreBI	PreB II L	PreB II s	Immature B
Adults	2,210046	2,06741	1,594463815	1,497011235	1,227979
Children	1,981015	2,048694	1,587382965	1,86495797	1

**E2A**	ProB	PreBI	PreB II L	PreB II s	Immature B
Adults	9,9752	5,320025	4,952525349	9,747684281	3,429546
Children	1	3,358831	8,628009114	13,98177707	1,081861

**IGF2BP3**	ProB	PreBI	PreB II L	PreB II s	Immature B
Adults	23,21202	6,086729	1		31,3438
Children	314,9727	422,9666	256,0559962	253,9866121	196,7482

**ID2**	ProB	PreBI	PreB II L	PreB II s	Immature B
Adults	20,50488	9,342577	34,1674553	12,03820014	3,510373
Children	2,219623	6,004678	14,71910446	11,58862994	1

**RAG2**	ProB	PreBI	PreB II L	PreB II s	Immature B
Adults	30,68071	27,62191	13,64838188	23,95233777	3,011707
Children	29,70468	24,13044	25,08587131	44,99233291	1

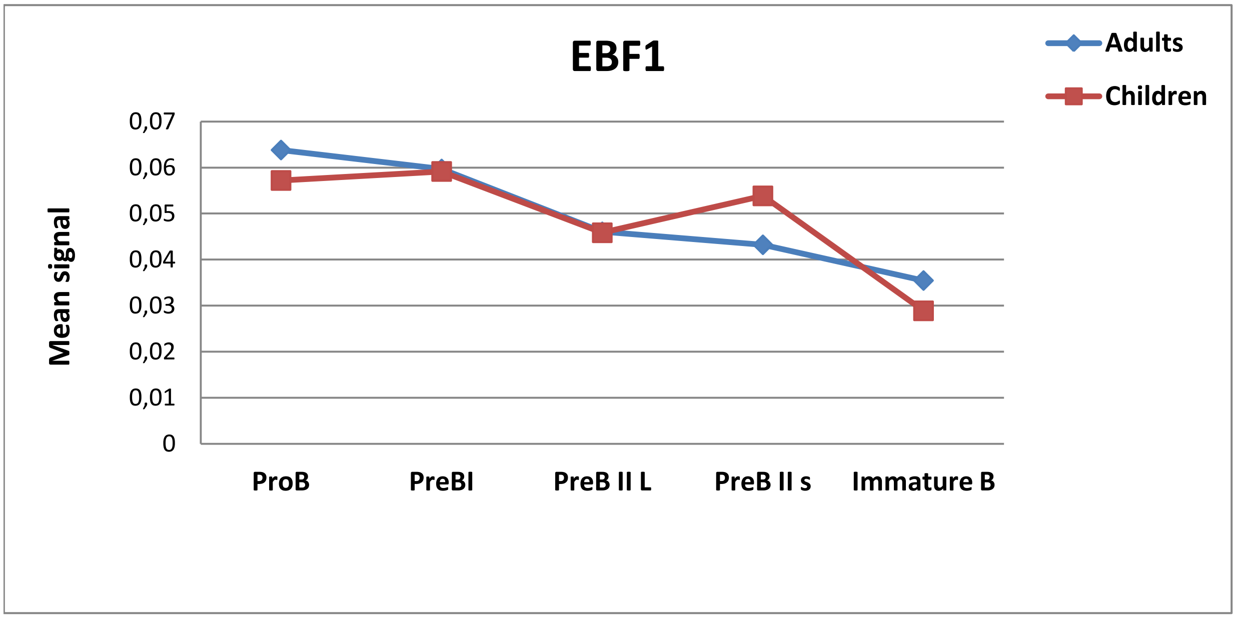

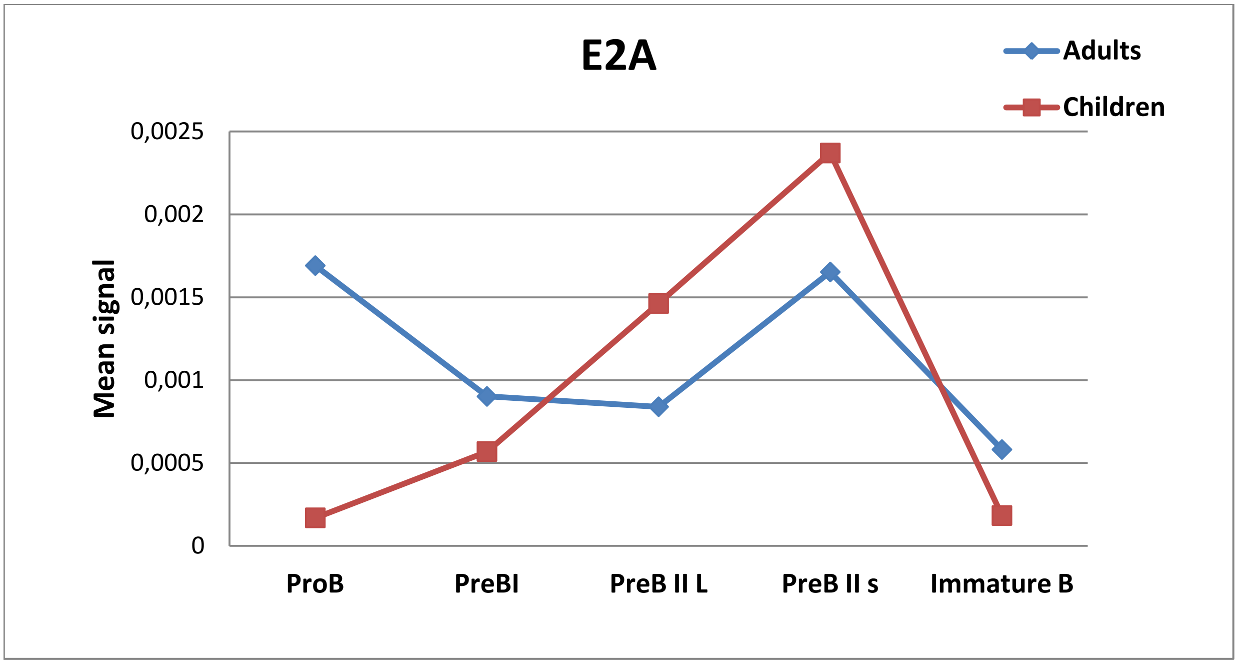
E2A
Adults
Children
Mean signal
0,0025
0,002
0,0015
0,001
0,0005
0
ProB
PreBI
PreB II L
PreB II s
Immature B

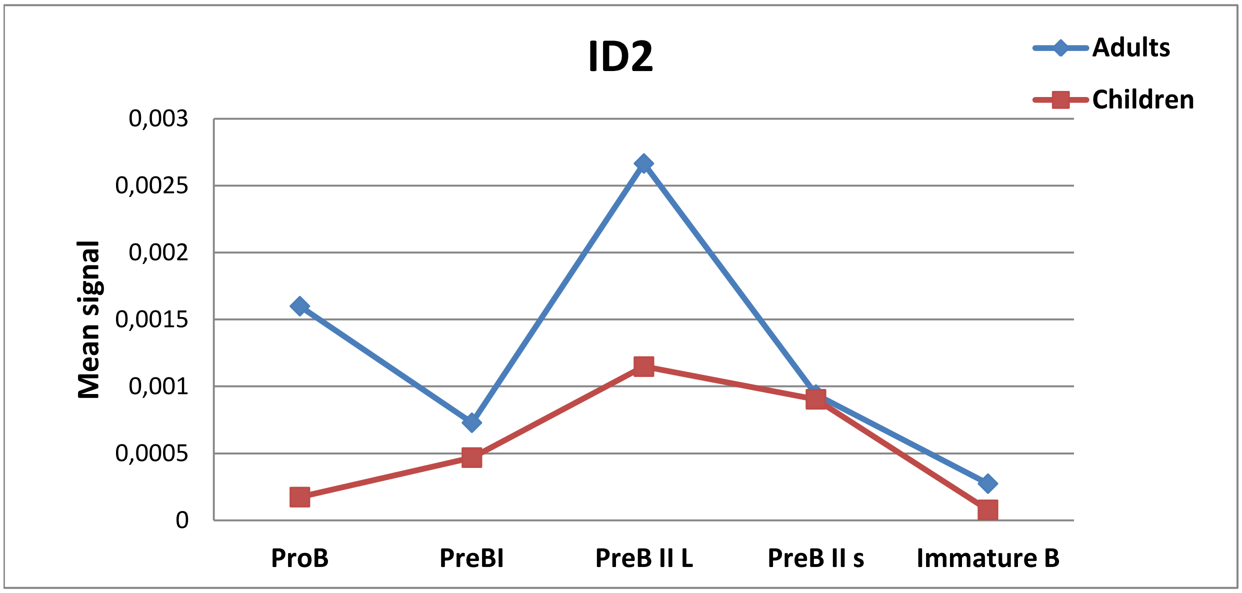
ID2
Adults
Children
Mean signal
0,003
0,0025
0,002
0,0015
0,001
0,0005
0
ProB
PreBI
PreB II L
PreB II s
Immature B

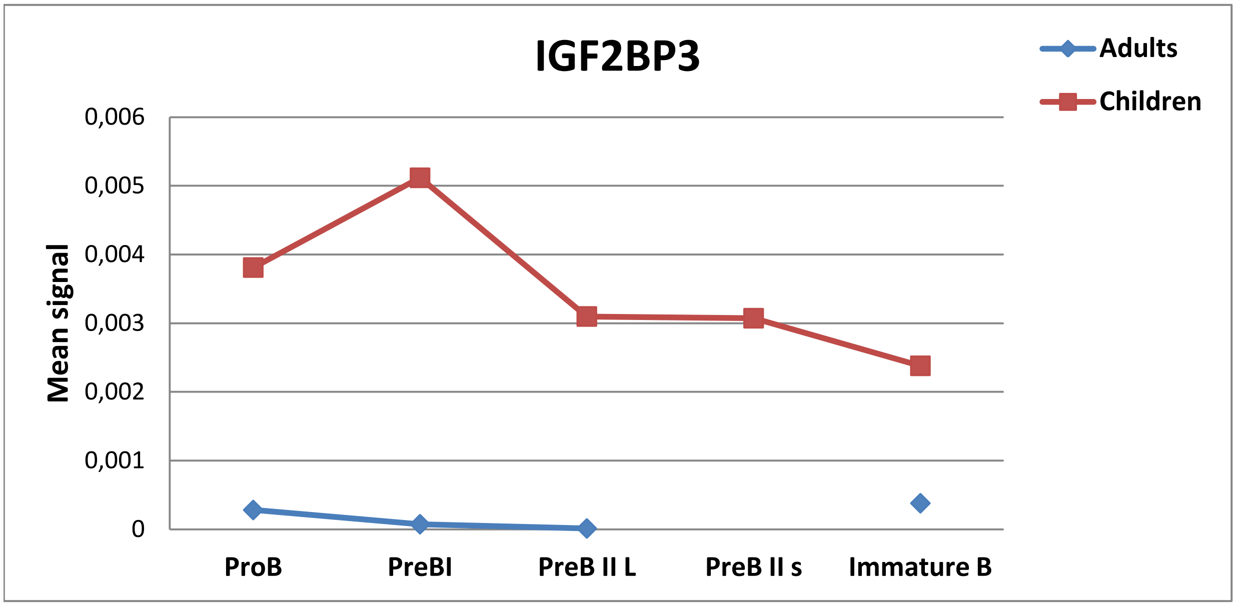
IGF2BP3
Adults
Children
Mean signal
0,006
0,005
0,004
0,003
0,002
0,001
0
ProB
PreBI
PreB II L
PreB II s
Immature B

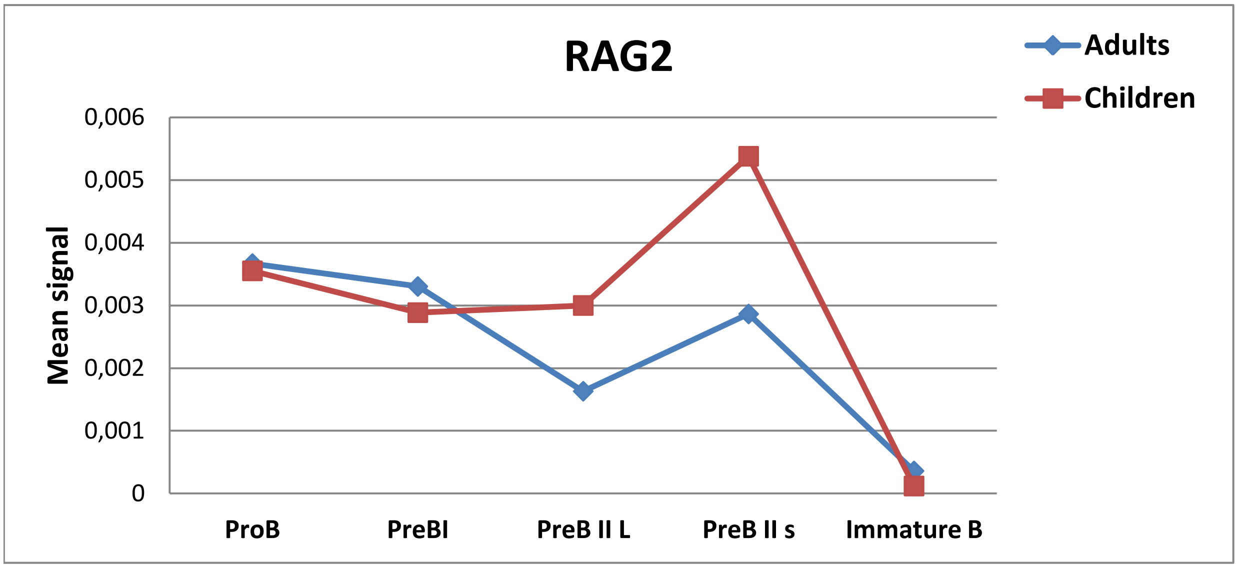
RAG2
Adults
Children
Mean signal
0,006
0,005
0,004
0,003
0,002
0,001
0
ProB
PreBI
PreB II L
PreB II s
Immature B
