# Supplementary material for: Transcriptional Profiling of mRNAs and microRNAs in Human Bone Marrow Precursor B Cells Identifies Subset- and Age-Specific Variations
Source: PLoS One. 2013 Jul 30;8(7):e70721. doi: 10.1371/journal.pone.0070721 (PMC3728296; doi:10.1371/journal.pone.0070721)
Supplement: Table S3 — (PDF) [file pone.0070721.s007.pdf]

Differentially expressed genes during each stage transit in adults and children, respectively (p-value < 0.05, fold change ≥ |2|)

ADULTS

Adult PreBI vs ProB

Transcript Cluster	Gene Symbol	p-value	fold change	Mean signal ProB	Mean signal PreBI
3578152	**TCL1A**	0,00007	3,803	11	41
4014387	RPSAP15	0,00806	3,476	10	35
3512948	C13orf18	0,00519	3,358	23	79
2841460		0,04175	3,270	3	9
3928590	KRTAP21-1	0,00930	3,239	7	24
2542972	NDUFAF2	0,03478	3,193	2	7
2674436		0,01686	3,166	4	14
3677187		0,02353	3,098	5	14
3335822		0,00324	3,022	9	28
3958307		0,03095	3,015	13	38
3470354		0,02413	2,976	30	90
3126191	PSD3	0,01657	2,975	29	85
4035833	CD24	0,00600	2,933	30	88
2597867	IKZF2	0,00081	2,815	19	53
3954879	VPREB3	0,00471	2,810	103	288
3524999	LIG4	0,01851	2,801	87	243
2781138	**LEF1**	0,00588	2,774	127	354
3647421	ABAT	0,00761	2,672	10	26
3286792	C10orf25	0,00936	2,637	23	60
3825154		0,01285	2,636	9	23
3275246		0,01946	2,608	8	22
3966225	RABL2B	0,01796	2,599	36	92
2318867		0,04865	2,564	8	20
3677164	TCEB2	0,04247	2,563	23	59
3402315	CD9	0,02108	2,541	93	236
2563785	**IGK@**	0,00108	2,493	135	337
3819749		0,04237	2,489	20	51
2958861	GUSBL2	0,01196	2,475	8	20
2964553	BACH2	0,00094	2,427	63	153
3046556	TARP	0,02016	2,389	32	76
3126368	PSD3	0,01651	2,371	15	35
3205293	PAX5	0,00019	2,363	98	231
3831223		0,03677	2,355	7	17
2875193	P4HA2	0,04007	2,351	46	107
2616596	ARPP21	0,02901	2,349	106	250
3129149	PBK	0,02463	2,346	12	29

2790109	ANXA2P1	0,00043	2,327	9	21
3825153		0,01234	2,321	45	103
3570141		0,00463	2,320	18	41
3855099		0,01681	2,319	7	17
3113894	ZHX2	0,01352	2,304	94	217
2599946		0,01187	2,294	8	18
3766533	CD79B	0,00493	2,262	52	118
2931569	AKAP12	0,03668	2,254	165	373
3226138	AK1	0,00581	2,185	33	72
2832043		0,00830	2,175	16	36
3275586		0,03522	2,171	103	223
3365776	E2F8	0,00522	2,161	34	73
4007899	SYP	0,01582	2,143	11	24
2362282	OR10Z1	0,03211	2,116	8	17
3838385	CD37	0,01555	2,112	27	57
2320088		0,00064	2,108	38	81
3744263	AURKB	0,00005	2,096	60	126
3977886	SSX8	0,02284	2,096	64	135
3809820		0,00415	2,091	14	28
2874739		0,00759	2,081	13	28
3539724	SYNE2	0,01433	2,079	17	36
2459411		0,03659	2,076	10	22
3361040		0,04216	2,067	7	14
2841482		0,01692	2,055	7	15
3666989	CLEC18C	0,03659	2,040	9	18
3850166	S1PR2	0,01240	2,038	8	16
3204648	CD72	0,00486	2,022	45	91
2451200	UBE2T	0,01448	2,013	37	74
3536336	CDKN3	0,02118	2,011	87	175
3819574		0,02847	-2,001	189	95
2564816	ANKRD36B	0,04309	-2,002	100	50
2818517	VCAN	0,03191	-2,006	19	9
2514516	KLHL23	0,00234	-2,011	37	18
3499585	BIVM	0,00718	-2,014	14	7
2320048 2320059	TARDBP	0,03982	-2,017	274	136
3570089		0,02834	-2,019	32	16
2601414	SERPINE2	0,00729	-2,021	20	10
2883661		0,00778	-2,027	34	17
2854327	FYB	0,03404	-2,033	19	9
2671968	LZTFL1	0,00012	-2,037	65	32
2674422		0,04359	-2,038	6	3
2783473	C4orf3	0,03899	-2,042	374	183
3182984	NIPSNAP3B	0,00029	-2,043	42	21
2955282	SUPT3H	0,00000	-2,044	45	22
3392885		0,02952	-2,054	156	76
2842561	HIGD2A	0,04842	-2,054	150	73
2993727	SNX10	0,00319	-2,058	50	24

3714896	FAM27L	0,00148	-2,059	42	21
3096271	C8orf40	0,00463	-2,059	283	137
3315675	IFITM1	0,01013	-2,061	47	23
3096464		0,02937	-2,062	18	9
3676739		0,03542	-2,063	52	25
3630195		0,00522	-2,070	628	303
2893109	LOC100129033	0,00254	-2,071	20	10
2840676		0,01251	-2,072	59	29
3714177	CYTSB	0,00056	-2,078	70	33
2902444	AIF1	0,00031	-2,084	158	76
3676709		0,03764	-2,085	177	85
3188050	MRRF	0,00027	-2,086	52	25
2356425	PDZK1	0,04124	-2,094	25	12
3217077	HEMGN	0,01519	-2,098	12	6
2971801	MAN1A1	0,00057	-2,103	260	124
2496536	RPL31	0,03239	-2,106	79	38
2565149		0,03512	-2,108	139	66
36997573699773	KARS	0,02237	-2,109	34	16
4011844	IL2RG	0,00032	-2,119	136	64
2734784	AFF1	0,00001	-2,122	478	225
2421121	ODF2L	0,00003	-2,125	38	18
3136178	PLAG1	0,02654	-2,129	21	10
2975741	MAP7	0,00079	-2,132	40	19
3630183		0,02826	-2,141	335	157
2527196	RPL37A	0,00107	-2,144	58	27
2949109		0,04057	-2,147	310	144
2830861	EGR1	0,00058	-2,149	108	50
2318795		0,00505	-2,152	23	11
2908488		0,00448	-2,154	341	158
39596313959653	EIF3D	0,00228	-2,157	481	223
3041816	DFNA5	0,00092	-2,161	17	8
2536380		0,04973	-2,170	23	10
2643217	TF	0,01272	-2,175	33	15
2880361	JAKMIP2	0,00018	-2,197	29	13
2518272	ITGA4	0,00016	-2,201	238	108
3420442	IRAK3	0,00006	-2,203	44	20
2593159	STK17B	0,00026	-2,205	164	75
23200482320077	TARDBP	0,04024	-2,206	92	42
2604390	ARL4C	0,00027	-2,209	57	26
3275166		0,04225	-2,210	92	42
2822215	PAM	0,00001	-2,220	19	8
3959742		0,01541	-2,224	22	10
3746675	CDRT4	0,00624	-2,225	10	4
2993690		0,00683	-2,225	94	42
3136888	TOX	0,00152	-2,226	94	42
2649723	MFSD1	0,00041	-2,226	169	76
3556288		0,00819	-2,232	96	43

2768654	OCIAD2	0,00169	-2,242	84	37
2908500		0,00281	-2,248	259	115
2461473	TARBP1	0,00316	-2,252	144	64
3474541		0,03502	-2,258	95	42
3464912	POC1B	0,00011	-2,261	35	15
2746024	ABCE1	0,00507	-2,268	114	50
2701109	IGSF10	0,00651	-2,270	29	13
3251393	DDIT4	0,00148	-2,273	66	29
3326635	CD44	0,00289	-2,290	103	45
2705266	TNIK	0,00035	-2,292	31	13
2588889	LOC100130691	0,00025	-2,293	25	11
2526806	FN1	0,01380	-2,297	44	19
2348992	VCAM1	0,01441	-2,297	13	6
2924619	TRMT11	0,00110	-2,307	36	16
3617574	GOLGA8B	0,00244	-2,310	97	42
2765935	GAFA3	0,00192	-2,317	217	94
2406311		0,03492	-2,325	11	5
2421000	COL24A1	0,00480	-2,332	37	16
2517408	AGPS	0,00285	-2,344	333	142
3256560	MINPP1	0,00320	-2,355	20	9
2993686		0,03652	-2,356	35	15
3725856		0,00743	-2,358	32	14
2928461	GPR126	0,00065	-2,363	14	6
2536442		0,04742	-2,366	5	2
2536307		0,02639	-2,386	18	8
3275574		0,04570	-2,394	11	5
3527662	RNASE6	0,00770	-2,406	37	15
2713950	ZNF141	0,00391	-2,408	97	40
2340433	LEPR	0,00006	-2,416	19	8
3630217		0,01143	-2,417	30	12
2742224	SPRY1	0,00045	-2,426	364	150
3740644		0,04297	-2,428	18	7
2938972	SERPINB1	0,00002	-2,430	115	47
2820925	RHOBTB3	0,00110	-2,430	35	15
3725850		0,01664	-2,433	83	34
2599901[illegible]599903	C2orf24	0,03832	-2,439	71	29
2436196		0,01028	-2,442	85	35
3551855		0,02628	-2,442	170	69
3577078	LGMN	0,01023	-2,443	166	68
3306571		0,02074	-2,444	16	7
2840782		0,04368	-2,449	18	7
3351766		0,04291	-2,454	49	20
2993206	MPP6	0,00079	-2,459	100	41
2328465[illegible]328501	KHDRBS1	0,02455	-2,474	97	39
2320064		0,03822	-2,475	238	96
2840680		0,02962	-2,476	124	50
2949113		0,00973	-2,477	22	9

2923661	GJA1	0,00228	-2,483	39	16
2991233	AHR	0,00000	-2,483	61	25
3811339	BCL2	0,00071	-2,484	151	61
3020192	TES	0,00054	-2,487	113	45
3076753	KIAA1147	0,00189	-2,488	25	10
2406395		0,00218	-2,488	24	10
3916290	FLJ42200	0,00107	-2,513	18	7
3819761		0,00838	-2,516	146	58
2700244	CP	0,00589	-2,521	10	4
3255506		0,03016	-2,527	28	11
3442854	SLC2A3	0,00059	-2,528	56	22
2665199	SATB1	0,00016	-2,532	357	141
3959684		0,00008	-2,540	490	193
3561110	RALGAPA1	0,00483	-2,547	15	6
3177880	DAPK1	0,00211	-2,547	46	18
2636125	CD200	0,00001	-2,583	84	33
3740580		0,01530	-2,586	47	18
2840656		0,04433	-2,588	78	30
2908487		0,03699	-2,592	83	32
3980926		0,03871	-2,606	43	16
3819669		0,04258	-2,607	84	32
3233049	AKR1C3	0,00103	-2,647	43	16
2841470		0,04306	-2,662	10	4
3980922		0,03544	-2,670	14	5
2774971	ANTXR2	0,00013	-2,671	173	65
2890413	RNF130	0,00029	-2,688	87	32
2686775		0,04609	-2,703	143	53
2325214		0,00898	-2,706	49	18
3475102		0,02913	-2,716	68	25
3699808		0,01474	-2,729	50	18
3740638		0,02710	-2,729	193	71
3556228		0,00797	-2,735	111	41
2557659		0,02548	-2,741	22	8
2727587	KIT	0,00000	-2,748	36	13
2701033	P2RY14	0,00079	-2,750	277	101
3699757 3699771	KARS	0,00521	-2,755	48	17
2993672		0,00888	-2,779	39	14
2343511	IFI44	0,01907	-2,781	78	28
2591421	TFPI	0,00007	-2,809	15	5
3630197		0,03793	-2,818	9	3
2949059		0,01249	-2,840	59	21
2349402	AMY2B	0,00695	-2,872	113	39
3556266		0,01558	-2,876	68	24
3959651		0,00145	-2,882	134	46
3630225		0,00647	-2,885	84	29
3676745		0,02974	-2,919	116	40
3306557		0,02045	-2,941	13	5

2565211		0,03145	-2,942	35	12
3630219		0,00530	-2,973	33	11
3319347		0,02994	-2,986	9	3
3401704	CCND2	0,00112	-2,998	74	25
3740640		0,04961	-3,006	46	15
2840674		0,02176	-3,022	42	14
2523632		0,01284	-3,027	96	32
3748449	CCDC144A	0,00097	-3,042	85	28
2444283	TNFSF4	0,00004	-3,068	116	38
2638988	PARP15	0,00033	-3,068	39	13
2791197	PDGFC	0,00011	-3,078	39	13
3275272		0,00018	-3,108	128	41
3298738	WAPAL	0,00454	-3,116	94	30
2816506	S100Z	0,00078	-3,127	46	15
3269939	DOCK1	0,00020	-3,130	31	10
2383811		0,04685	-3,139	39	13
3749588		0,02970	-3,164	94	30
3475016		0,03358	-3,174	55	17
2908762	RUNX2	0,00016	-3,196	119	37
3958341		0,00792	-3,214	26	8
3275202		0,01457	-3,243	17	5
2949107		0,01163	-3,262	37	11
2486178	MEIS1	0,00002	-3,283	82	25
3041875	OSBPL3	0,00001	-3,295	99	30
3275274		0,00003	-3,332	142	43
2873785	ALDH7A1	0,01831	-3,398	64	19
2840666		0,01054	-3,439	60	17
3020222	LOC100128868	0,00283	-3,471	19	5
2841342		0,00199	-3,509	49	14
2406245□406261	PSMB2	0,03038	-3,525	45	13
2320112		0,01288	-3,591	65	18
2557657		0,00250	-3,614	47	13
3182930	OR13D1	0,00447	-3,620	17	5
3286602	CXCL12	0,00221	-3,632	50	14
2405104	ZBTB8OS	0,00664	-3,647	22	6
3831168□831178	CAPNS1	0,00134	-3,670	37	10
3699892		0,00563	-3,678	59	16
3507199	FLT3	0,00130	-3,690	257	70
3364755		0,00031	-3,705	157	42
3477967	MGC16384	0,01962	-3,751	42	11
3556268		0,02993	-3,761	42	11
3725966		0,01175	-3,850	32	8
2372781	RGS1	0,00159	-3,857	25	6
3203382	SMU1	0,00335	-3,877	47	12
3959744		0,00588	-3,993	14	4
3096494		0,02858	-4,021	23	6
2584957	SCN3A	0,00198	-4,047	135	33

3959738		0,02417	-4,070	11	3
3386217	CHORDC1	0,00539	-4,079	45	11
2436198		0,00016	-4,138	33	8
3253452		0,01714	-4,176	19	5
3980930		0,01536	-4,236	12	3
3844035		0,00027	-4,292	36	8
3090209	ADAM28	0,00097	-4,319	42	10
2418078	NEGR1	0,00004	-4,394	88	20
2806468	IL7R	0,00343	-4,405	124	28
3021158	C7orf58	0,00115	-4,454	35	8
3433466	NCRNA00173	0,00143	-4,522	58	13
3740642		0,00295	-4,538	37	8
2925590	TMEM200A	0,00063	-4,551	40	9
2730673	MOBKL1A	0,00000	-4,667	102	22
2443450	SELL	0,00009	-4,706	664	141
3470253 3470282	SART3	0,03970	-5,132	13	2
3214451	NFIL3	0,00001	-5,267	93	18
2325212		0,00062	-5,734	47	8
3174816	ANXA1	0,00002	-5,785	79	14
3110217	BAALC	0,00014	-6,856	155	23
2761842	PROM1	0,00012	-7,206	68	9
2772566	IGJ	0,00000	-7,363	449	61

Adult PreBII L vs PreBI

				Mean signal	
Transcript Cluster	Gene Symbol	p-value	fold change	PreBI	PreBII L
3755862	IKZF3	0,00000	11,525	13	149
3904691	SAMHD1	0,00000	10,343	11	113
3578152	**TCL1A**	0,00000	7,797	41	320
2823880	CAMK4	0,00000	7,753	9	73
2782545	CAMK2D	0,00000	6,273	28	177
3648391	TNFRSF17	0,00002	6,134	6	37
2772566	IGJ	0,00002	6,101	61	372
3844035		0,00002	5,817	8	48
2766192	TLR10	0,00001	5,774	21	122
3098977	LYN	0,00000	5,713	24	138
2638988	PARP15	0,00000	5,535	13	71
2860178	CD180	0,00000	5,334	15	82
3298738	WAPAL	0,00010	5,282	30	159
3275166		0,00012	5,233	42	218
4016193	TMSB15A	0,01308	5,195	13	69
2524653	ADAM23	0,00000	5,189	14	75
2599901 2599903	C2orf24	0,00043	5,125	29	149
2597867	IKZF2	0,00000	4,939	53	261

3556556 3556580	DAD1	0,00852	4,871	10	47
2688717	BTLA	0,00002	4,854	8	38
3590341	CHP	0,00000	4,850	79	381
2366884	C1orf129	0,00008	4,774	4	21
3669552	VAT1L	0,00000	4,754	21	98
3945545	APOBEC3B	0,00003	4,568	11	49
2372858	RGS2	0,00179	4,513	212	959
3386217	CHORDC1	0,00377	4,360	11	48
3577256	C14orf142	0,00063	4,302	16	69
3980930		0,01563	4,219	3	11
2388085	KMO	0,00000	4,185	12	49
2621570		0,04844	4,181	16	67
3593575	SLC27A2	0,00000	4,173	7	28
2363618	SDHC	0,00231	4,157	6	26
3555887		0,00390	4,142	17	72
2908487		0,00284	4,140	32	133
3470470		0,00728	4,081	7	28
3470549	CORO1C	0,00017	4,055	40	161
2328565		0,00525	4,008	6	23
3203382	SMU1	0,00275	4,004	12	48
3630197		0,00722	3,961	3	12
3173673	PIP5K1B	0,00000	3,946	10	41
3291435	RTKN2	0,00002	3,910	14	54
2806468	IL7R	0,00663	3,898	28	109
3288013	BMS1P1	0,01451	3,840	3	11
3844039		0,03551	3,784	5	21
3253459		0,00291	3,780	3	12
3475026		0,01789	3,772	7	27
2320134		0,01161	3,735	4	15
2891341	IRF4	0,00001	3,729	55	204
2747893	ARFIP1	0,00006	3,725	9	34
2698565	TFDP2	0,00000	3,719	114	423
3175971	PSAT1	0,00009	3,603	8	29
2993658		0,00178	3,577	9	34
3275574		0,00493	3,570	5	17
3362719 3362765	EIF4G2	0,00304	3,499	6	23
2523632		0,00562	3,489	32	110
3197955	GLDC	0,00005	3,471	44	153
2844293		0,03511	3,441	5	16
3699892		0,00821	3,441	16	55
3464983	ATP2B1	0,00000	3,406	35	118
3815399	CNN2	0,00059	3,398	9	32
3924041	ADARB1	0,00001	3,393	23	78
2328465 2328501	KHDRBS1	0,00339	3,383	39	133
2565149		0,00122	3,352	66	221
3129149	PBK	0,00222	3,343	29	97
3699757 3699771	KARS	0,00118	3,342	17	58

2406245 2406261	PSMB2	0,03762	3,340	13	42
2686775		0,01808	3,305	53	175
2467194		0,03569	3,287	3	11
2908500		0,00004	3,287	115	379
2319340	SLC25A33	0,01395	3,268	32	103
3203855	DCAF12	0,00001	3,265	36	118
2638676	EAF2	0,00000	3,258	81	263
3708826	EIF4A1	0,00269	3,255	39	128
2325212		0,01485	3,252	8	27
3651478	ACSM3	0,00031	3,251	16	51
3629103	KIAA0101	0,00143	3,250	92	298
2680819	SUCLG2	0,00123	3,241	20	66
2389062	FAM36A	0,02992	3,237	27	88
3973839	CYBB	0,00260	3,218	17	54
2724671	RHOH	0,00001	3,209	57	182
2500275	BCL2L11	0,00007	3,192	30	97
3538213	DAAM1	0,00003	3,188	20	63
2565211		0,02206	3,172	12	38
2565200		0,01955	3,164	10	31
3556290		0,00587	3,158	11	34
3699808		0,00599	3,152	18	58
2468622	ID2	0,01015	3,148	43	137
3329983	PTPRJ	0,00000	3,147	12	38
3630225		0,00357	3,143	29	92
3474952		0,02154	3,126	10	33
3239584	MYO3A	0,00000	3,106	7	21
2768654	OCIAD2	0,00004	3,092	37	115
3470398		0,01610	3,083	7	23
3301218	PDLIM1	0,00000	3,081	111	341
3959744		0,02244	3,074	4	11
3551725		0,03536	3,055	10	31
2775259	RASGEF1B	0,00003	3,050	46	141
3807487 3807501	RPL17	0,04227	3,047	29	88
3902743	C20orf112	0,00123	3,044	19	58
2828135	LYRM7	0,00502	3,035	26	79
3003143	MRPS17	0,00661	3,027	62	188
2417528	DEPDC1	0,00249	3,002	16	49
3203162	NDUFB6	0,00100	3,002	48	146
2688605	GCET2	0,00004	2,995	46	139
2689286	KIAA1407	0,00000	2,983	13	40
3556058		0,00363	2,969	4	12
2373842	PTPRC	0,00000	2,962	93	276
3096271	C8orf40	0,00008	2,957	137	406
2557659		0,01730	2,948	8	24
2837479	THG1L	0,00005	2,934	39	115
2674472		0,04294	2,931	7	21
2898597	GMNN	0,00013	2,931	47	139

3559690	HEATR5A	0,00182	2,930	8	23
2969289	WASF1	0,01061	2,925	53	154
2320064		0,01550	2,925	96	281
3555521	GAFA1	0,00093	2,920	10	28
3809705		0,02030	2,915	9	25
3595979	CCNB2	0,00543	2,912	53	154
3248289	CDK1	0,00146	2,905	17	50
3346548	BIRC3	0,00089	2,887	9	26
3980926		0,02432	2,860	16	47
3831168 3831178	CAPNS1	0,00774	2,854	10	29
2914777	TTK	0,00064	2,845	19	55
2766492	C4orf34	0,00000	2,845	106	301
3696697	NOB1	0,00015	2,822	14	38
2440354	CD48	0,00019	2,807	90	252
3590086	RAD51	0,00067	2,802	44	124
3837731	EMP3	0,00002	2,798	29	81
3214451	NFIL3	0,00236	2,788	18	49
3625761	MNS1	0,00097	2,775	32	89
3819543 3819609	HNRNPM	0,02767	2,769	60	166
2742985	PLK4	0,00033	2,761	39	107
2855058	OXCT1	0,00001	2,757	42	117
3044753	LSM5	0,00634	2,752	21	58
2893392	LY86	0,00138	2,752	16	44
3384321	RAB30	0,00083	2,739	14	39
2874686 2874696	HINT1	0,00867	2,714	60	164
2553730	MTIF2	0,00001	2,714	29	78
3982560	P2RY10	0,00001	2,711	10	27
3433747	RFC5	0,00001	2,711	50	137
2722377	STIM2	0,00000	2,709	67	181
2416218	ITGB3BP	0,00335	2,701	51	137
2565243		0,01679	2,701	68	184
4052881	FAM72D	0,00112	2,696	41	112
2320112		0,04919	2,692	18	49
3869847	ZNF468	0,02632	2,683	9	24
2835792	GM2A	0,00051	2,683	38	103
3082590	LOC286161	0,00204	2,681	51	138
3776139	NDC80	0,00773	2,660	56	149
2838656	HMMR	0,00713	2,659	61	161
3256560	MINPP1	0,00097	2,659	9	23
3375999	C11orf48	0,00259	2,657	69	185
2908488		0,00050	2,654	158	420
3556266		0,02472	2,648	24	62
2897172	RNF144B	0,00006	2,645	31	82
2709606	RPL39L	0,00073	2,644	157	415
2968375		0,00614	2,642	14	36
2565219		0,01402	2,636	29	76
2920962	FIG4	0,00000	2,633	26	67

3728037	SCPEP1	0,00000	2,623	37	97
2585933	SPC25	0,01911	2,622	106	279
3408505	LRMP	0,00000	2,620	88	230
2652675	ECT2	0,00058	2,619	28	74
2901699		0,01424	2,618	29	77
3145107	CCNE2	0,00103	2,610	49	128
3676739		0,00673	2,607	25	65
3275524		0,00231	2,602	10	25
2450345	KIF14	0,00032	2,597	32	84
2733483	BMP3	0,00025	2,584	25	64
2780172	CENPE	0,00154	2,582	29	75
3428845	C12orf48	0,00008	2,579	9	23
2406722	LSM10	0,00183	2,577	8	21
3275588		0,04703	2,565	17	44
3633794	ETFA	0,00007	2,565	68	173
3212277	C9orf64	0,00003	2,565	8	20
2933522	GTF2H5	0,01438	2,564	15	39
2557657		0,02184	2,562	13	33
2818454	XRCC4	0,00029	2,561	38	97
3328069	HSD17B12	0,00038	2,547	19	48
3279058	ACBD7	0,02488	2,546	12	31
2364438	NUF2	0,00554	2,546	78	197
3699767		0,00021	2,545	88	224
2859667	CENPK	0,00630	2,543	68	173
2783473	C4orf3	0,00854	2,538	183	465
2940920	EEF1E1	0,00220	2,535	10	24
3362719 3362764	EIF4G2	0,00040	2,525	72	183
4015763	GLA	0,00007	2,518	29	74
2363852	FCRLA	0,00014	2,517	17	43
2515369	HAT1	0,00225	2,514	79	199
2413423	TMEM48	0,00028	2,510	33	83
3551778		0,02130	2,506	29	73
3807569	ACAA2	0,00003	2,505	84	210
2339872	ROR1	0,00005	2,504	18	45
4013828	HMGN5	0,00026	2,504	23	58
3508330	HSPH1	0,00016	2,500	73	182
3556024		0,04054	2,497	6	15
3013952	ACN9	0,00193	2,493	23	57
3809685		0,02123	2,491	41	102
3096214	VDAC3	0,00006	2,487	155	386
2840782		0,04059	2,485	7	18
3658925	ORC6L	0,00007	2,480	69	171
3512948	C13orf18	0,03140	2,475	79	195
3141346	PEX2	0,00043	2,474	14	36
3551786		0,01147	2,474	33	81
3020192	TES	0,00059	2,470	45	112
3020222	LOC100128868	0,02468	2,469	5	13

2589868	CCDC141	0,00007	2,463	14	34
3066436	PUS7	0,00018	2,462	7	17
3389450	CASP1	0,02974	2,462	48	118
2565147		0,00019	2,461	93	230
3773312	EIF4A3	0,00001	2,458	82	201
2318736 2318743	PARK7	0,00044	2,452	102	250
3318009	RRM1	0,00009	2,451	85	208
3012064	CDK14	0,00909	2,447	47	114
3275154		0,01193	2,447	83	204
3527514	PNP	0,00025	2,446	36	89
2620256	KIF15	0,00454	2,438	75	184
2346399	CDC7	0,00163	2,437	52	127
2406420	CLSPN	0,00708	2,435	86	209
2409904	EIF2B3	0,00096	2,430	33	80
3630219		0,02026	2,430	11	27
3689880	SHCBP1	0,00039	2,429	61	148
2599901 2599907	C2orf24	0,02759	2,426	16	40
3417146	CDK2	0,00006	2,420	67	163
2813414	CCNB1	0,00253	2,417	121	293
2778980	EIF4E	0,00343	2,416	51	123
2877378	CDC25C	0,00188	2,413	18	44
2610241	FANCD2	0,00000	2,409	32	77
3331903	FAM111B	0,00232	2,406	116	280
3403092	PTPN6	0,00000	2,406	57	138
3570089		0,00743	2,402	16	37
2971678	MCM9	0,00062	2,401	30	73
2924619	TRMT11	0,00071	2,396	16	38
3204648	CD72	0,00073	2,393	91	217
3470253 3470259	SART3	0,00541	2,392	23	54
2841342		0,02523	2,392	14	33
2411228	STIL	0,00095	2,391	23	54
3004768	ZNF273	0,00603	2,384	12	28
2401643	FUCA1	0,00008	2,382	13	30
4045780	SFRS13A	0,00116	2,381	89	212
2840640		0,00012	2,380	337	801
3419849	TBK1	0,00000	2,377	32	75
3223687	PHF19	0,00024	2,377	9	20
3257338	KIF20B	0,00079	2,377	38	91
3255506		0,04206	2,375	11	26
2328613		0,00856	2,373	10	23
3448152	ITPR2	0,00000	2,372	62	147
3854218	HAUS8	0,00012	2,372	28	67
2729667	STAP1	0,00330	2,372	32	77
2842561	HIGD2A	0,01957	2,372	73	173
2975655	FAM54A	0,02133	2,371	65	155
3875195	MCM8	0,00025	2,370	56	132
3258168	KIF11	0,01310	2,370	78	185

3958341		0,04395	2,368	8	19
3275132 3275175	GDI2	0,00246	2,362	125	296
3991698	HPRT1	0,00098	2,360	62	147
3354210	SPA17	0,00100	2,357	21	49
3699764		0,00015	2,353	85	201
2318759		0,03259	2,351	7	17
3764002	MRPS23	0,00022	2,346	35	81
3301011	NOC3L	0,00038	2,343	18	42
3105430	LRRCC1	0,00082	2,340	24	57
2946268	HIST1H2BC	0,03107	2,340	109	254
3781980	TTC39C	0,00003	2,335	33	76
3556228		0,02323	2,331	41	95
2379754	SMYD2	0,00003	2,330	32	75
3554851	CRIP1	0,04773	2,325	30	70
2339414	USP1	0,00552	2,323	71	164
3076489	MRPS33	0,00741	2,323	38	89
3258910	HELLS	0,00191	2,321	90	208
3362719 3362739	EIF4G2	0,02339	2,320	54	126
2753952	ING2	0,00125	2,318	36	84
2438482	ISG20L2	0,00003	2,318	38	89
3470342		0,03751	2,317	9	20
3351775	TRAPPC4	0,00161	2,317	28	64
2952679	GLO1	0,00301	2,312	93	215
3367338	KIF18A	0,00128	2,307	27	62
2853275	CAPSL	0,00019	2,307	16	37
3168508	MELK	0,00342	2,306	44	101
2717165	TBC1D14	0,00001	2,305	114	262
2784027	ANXA5	0,03840	2,304	92	212
3539724	SYNE2	0,00592	2,303	36	84
3212300		0,03049	2,303	128	296
2521479	HSPE1	0,01524	2,299	29	66
2709486	RFC4	0,00279	2,298	91	209
3040967	RAPGEF5	0,00013	2,296	27	63
3725779 3725803	MYST2	0,04298	2,295	25	58
2754582	SNX25	0,00099	2,295	26	60
2577896	MCM6	0,01079	2,292	126	289
3275578		0,04091	2,292	36	84
3570109		0,04668	2,290	19	44
2766456	UGDH	0,00283	2,289	36	82
3516639	PCDH9	0,00005	2,289	27	62
2665572	SGOL1	0,00140	2,288	66	151
3364755		0,01493	2,287	42	97
3461341	CPM	0,00044	2,286	12	27
2908501		0,00036	2,286	208	475
3322072		0,02575	2,285	48	110
2991395	HDAC9	0,00000	2,282	24	54
2616932	MLH1	0,00022	2,282	50	113

2768145	COMMD8	0,00280	2,280	8	17
2451200	UBE2T	0,00467	2,280	74	169
3750785	SPAG5	0,00004	2,278	31	71
2374956	TIMM17A	0,00159	2,272	100	228
3565663	DLGAP5	0,00661	2,272	103	235
2881860	CCDC69	0,00009	2,271	80	182
3910785	AURKA	0,00729	2,270	38	87
3011454	DBF4	0,00038	2,269	11	25
3387483	MTMR2	0,00001	2,267	33	75
3335798		0,01061	2,267	38	86
3504617	SKA3	0,00385	2,264	40	91
3472089	RPL6	0,01908	2,264	16	35
3720896	CDC6	0,01081	2,261	94	212
3485863	EXOSC8	0,00162	2,260	43	98
2758686	LYAR	0,00595	2,256	31	70
3309383	PRDX3	0,00001	2,255	42	96
2826343	SNX24	0,00013	2,254	10	23
2840002	CCDC99	0,00398	2,253	87	195
3728964	PRR11	0,00094	2,251	49	111
2485176	MDH1	0,00013	2,249	39	87
3959657		0,01561	2,248	71	160
3513995	DLEU2	0,00358	2,248	13	28
2841392		0,03241	2,246	103	230
2536298 2536329		0,00118	2,245	59	132
2388219	EXO1	0,00554	2,244	62	139
2974635	VNN2	0,00065	2,244	11	24
2425118	SASS6	0,00072	2,243	29	66
2469094	TAF1B	0,00099	2,242	10	22
3471224	GPN3	0,00181	2,240	24	55
3621623	ELL3	0,00002	2,239	21	46
3150715	DSCC1	0,00397	2,238	41	92
2595443	WDR12	0,00707	2,238	51	113
3235789	MCM10	0,00090	2,237	48	108
3758317	BRCA1	0,00029	2,236	35	77
2406245 2406253	PSMB2	0,00475	2,235	106	237
2333658 2333677	ATP6V0B	0,04818	2,234	39	88
2349129	S1PR1	0,00087	2,232	16	35
3340697	UVRAG	0,00010	2,232	52	116
3683806	ERI2	0,00035	2,231	22	50
3515965	DIAPH3	0,00359	2,229	35	79
3958269		0,00044	2,226	43	96
2480992	MSH2	0,00029	2,225	71	158
2911413	PRIM2	0,00129	2,222	15	34
3570049 3570053	ERH	0,00460	2,222	67	149
2496536	RPL31	0,02268	2,221	38	84
3397589	ETS1	0,00000	2,220	80	178
3829687	GPI	0,00026	2,217	43	94

3512874	LCP1	0,00001	2,214	356	788
2871717	CCDC112	0,00037	2,213	15	34
2894689	TMEM14C	0,00046	2,210	20	44
2559696	TPRKB	0,00185	2,209	31	69
2720251	NCAPG	0,00657	2,207	138	305
3236538	RPP38	0,00312	2,206	9	19
2565221		0,02362	2,206	13	28
2866543	CETN3	0,00333	2,206	62	138
2463515	CHML	0,00010	2,203	44	97
3311157	OAT	0,00227	2,199	27	60
2633460	C3orf26	0,00043	2,197	46	102
3362719 3362762	EIF4G2	0,00969	2,195	73	160
2868283	RIOK2	0,00019	2,194	38	83
2687979	KIAA1524	0,00181	2,193	20	44
2993690		0,00780	2,193	42	93
2403470	DNAJC8	0,01540	2,192	80	176
2406766	MRPS15	0,00017	2,190	59	129
3468301	PMCH	0,01297	2,184	8	18
3340269	POLD3	0,00093	2,182	152	331
3474935 3474950	ANAPC5	0,04545	2,182	32	69
3056414	RFC2	0,00050	2,181	50	108
3113456	MTBP	0,00164	2,180	14	31
4011743	SLC7A3	0,00040	2,175	32	70
3505449	MIPEP	0,00000	2,175	24	53
3726934	NME1	0,00003	2,172	100	218
3347615	ACAT1	0,00014	2,172	21	46
2687840	IFT57	0,00053	2,172	25	54
2517013	MTX2	0,00787	2,172	11	25
3458133	PRIM1	0,00106	2,171	52	113
3477917	SLC15A4	0,00016	2,169	35	75
3743524		0,04495	2,168	12	27
3228097	TTF1	0,00033	2,167	19	42
3060245	SLC25A40	0,00855	2,165	21	46
3261923	AS3MT	0,01026	2,163	8	17
2565143 2565145	STARD7	0,00003	2,162	312	675
3494137	LMO7	0,00000	2,162	15	33
4050485	TUBB2C	0,04986	2,161	26	57
2746024	ABCE1	0,00801	2,157	50	109
3672489	IRF8	0,00050	2,157	25	55
3082181	NCAPG2	0,00197	2,156	43	92
3505937	CENPJ	0,00039	2,155	49	105
2783715	MAD2L1	0,03006	2,154	91	197
2974671	C6orf192	0,00094	2,154	14	30
2830638	KIF20A	0,00397	2,153	29	62
3409330	MRPS35	0,00038	2,153	41	88
2858592	DEPDC1B	0,01272	2,151	78	168
3607537	FANCI	0,00110	2,150	81	174

3959651		0,01652	2,150	46	100
3630217		0,02636	2,148	12	27
3301512	ALDH18A1	0,00126	2,148	33	71
3916290	FLJ42200	0,00529	2,148	7	16
3618736	RASGRP1	0,00005	2,145	38	82
2674418		0,01748	2,145	12	26
3352503	ARHGEF12	0,00231	2,143	14	30
3485074	RFC3	0,00086	2,140	127	271
2526759	ATIC	0,00054	2,140	73	156
3945572	APOBEC3C	0,00157	2,138	37	79
2878246	PFDN1	0,00007	2,138	25	54
3391816	USP28	0,00053	2,135	28	61
3429857	C12orf75	0,00173	2,132	110	235
2992963	CCDC126	0,00062	2,129	13	29
3333443	ASRGL1	0,00495	2,128	20	42
3608298	BLM	0,00027	2,126	64	135
2570616	BUB1	0,00155	2,124	65	138
2558595	FAM136A	0,00012	2,120	22	46
3179359	CENPP	0,00182	2,118	29	61
3090697	CDCA2	0,00186	2,116	30	63
3289031	TIMM23	0,00210	2,115	175	370
2565148		0,00003	2,114	269	568
2878446	NDUFA2	0,00990	2,113	128	271
2436141		0,00667	2,113	157	332
3460584	LLPH	0,00327	2,112	43	91
2913277	KCNQ5	0,00018	2,108	33	70
3752258	EVI2B	0,00011	2,108	79	166
2494484	NCAPH	0,00471	2,107	64	135
3707990	TXNDC17	0,00015	2,106	111	234
3145567	UQCRB	0,01363	2,105	9	20
2427720	DRAM2	0,00220	2,104	59	124
3881443	TPX2	0,00656	2,103	125	262
2908486		0,00335	2,102	265	557
3139950	LACTB2	0,00041	2,102	9	19
3570049 3570052	ERH	0,00018	2,100	193	406
3842141 3842156	RPL28	0,01796	2,100	39	83
2967650	RTN4IP1	0,00042	2,099	30	64
2571457	CKAP2L	0,00941	2,098	68	143
2594535	PPIL3	0,00060	2,097	94	198
3980917		0,00051	2,097	105	221
2860666	TAF9	0,00901	2,097	20	43
2612813	PLCL2	0,00011	2,095	78	163
2853768	NUP155	0,00083	2,095	69	144
3556966	HAUS4	0,00001	2,092	62	129
2635263	DZIP3	0,00000	2,091	19	39
3722060	VPS25	0,00919	2,089	56	117
3958253 3958267	C22orf28	0,00641	2,089	81	170

2748198	KIAA0922	0,00050	2,088	139	291
2805581	SUB1	0,00009	2,087	66	138
3468261	NUP37	0,00124	2,087	49	102
3240012	MASTL	0,00547	2,085	59	123
2378937	DTL	0,00712	2,085	207	432
2796510	MLF1IP	0,02985	2,085	84	175
2413685	SSBP3	0,00343	2,084	14	29
2347023	CCDC18	0,00455	2,081	48	99
3224591	STRBP	0,00002	2,079	158	329
2449559	ASPM	0,01597	2,078	80	167
3109201	SPAG1	0,00001	2,077	10	21
3589697	BUB1B	0,00239	2,077	141	292
2711644	ATP13A3	0,00004	2,075	58	120
2604254	HJURP	0,00224	2,074	67	139
2463425	FH	0,00053	2,072	31	65
2417174	SERBP1	0,03368	2,071	207	428
2737596	BANK1	0,00034	2,070	46	95
3306299	XPNPEP1	0,00025	2,070	137	284
2502300	DDX18	0,00279	2,068	112	231
3888133	CSE1L	0,00050	2,067	104	214
3591365	ADAL	0,00248	2,064	21	43
3428088	ACTR6	0,00285	2,062	17	36
2565143?2565160	STARD7	0,00037	2,062	485	1001
3742627	C17orf87	0,03207	2,062	13	27
3728776	RAD51C	0,00059	2,059	72	148
3630195		0,00559	2,057	303	624
3497790	IPO5	0,00008	2,055	109	223
2761151	RAB28	0,00812	2,055	16	34
2428501	SLC16A1	0,00804	2,052	75	155
3354799	CHEK1	0,00900	2,051	110	226
2365958	MPZL1	0,00002	2,051	46	94
2636695	ZDHHC23	0,00020	2,050	22	44
3374934	MS4A6A	0,00465	2,050	13	27
2531233	SP140	0,00208	2,050	7	14
3401804	RAD51AP1	0,00168	2,050	197	403
3628469	RPS27L	0,02888	2,049	47	97
2784113	CCNA2	0,00593	2,049	85	175
2766262	TLR6	0,01045	2,049	6	12
2965674	NDUFAF4	0,02698	2,046	49	100
2691575	POLQ	0,00400	2,046	44	91
3563395	POLE2	0,00421	2,045	13	26
3435362	KNTC1	0,00130	2,045	53	109
3841756	KIR2DL1	0,02233	2,041	10	19
3475020		0,04295	2,041	45	92
3756193	TOP2A	0,01087	2,040	269	549
3968664	HCCS	0,00001	2,039	46	93
3642572	SNRNP25	0,00011	2,038	160	326

3219621	CTNNAL1	0,00415	2,036	43	88
2403557	SNORA44	0,00168	2,033	90	182
3541137	EIF2S1	0,00016	2,032	199	404
3828112	CCNE1	0,00026	2,031	32	66
2343025	AK5	0,00072	2,031	20	40
3180263	HIATL1	0,00023	2,031	48	97
2417737	LRRC40	0,00121	2,030	20	40
3365776	E2F8	0,00960	2,029	73	149
2674245		0,03898	2,028	127	258
3699757?3699779	KARS	0,04494	2,028	55	112
2565143?2565163	STARD7	0,00935	2,028	173	351
2737840	CISD2	0,02088	2,027	52	105
2837810	UBLCP1	0,00030	2,027	59	119
2379863	CENPF	0,03013	2,026	206	418
2351854	C1orf162	0,00202	2,025	44	88
3725856		0,02488	2,024	14	28
3763270	MMD	0,00124	2,023	31	63
3197528	C9orf46	0,00737	2,023	9	19
3984655	CENPI	0,00309	2,020	25	50
3141809	MRPS28	0,00890	2,020	29	59
3176999	RMI1	0,00081	2,019	50	102
2820622	ANKRD32	0,00647	2,019	25	51
3878533	DTD1	0,00746	2,018	65	132
3752424	C17orf79	0,00064	2,016	23	47
3809671?3809680	NARS	0,03975	2,016	54	109
3676692		0,02300	2,014	100	201
3972093	POLA1	0,00042	2,014	114	231
2580635	MMADHC	0,01557	2,013	94	189
3703112	GINS2	0,00061	2,012	83	166
3258444	CEP55	0,00386	2,011	27	54
2654091	USP13	0,00109	2,011	35	71
3565571	WDHD1	0,00607	2,011	99	200
3451670	PUS7L	0,00037	2,010	12	24
3765580	BRIP1	0,00072	2,008	65	130
2700500	COMMD2	0,00093	2,008	106	213
3980898		0,02304	2,007	57	113
3591704	WDR76	0,01470	2,005	122	245
3591281	TMEM62	0,00007	2,005	24	47
3421523	YEATS4	0,00162	2,004	126	253
3096456		0,03288	2,004	62	124
2913694	CD109	0,00004	-2,007	27	14
2329341	ZSCAN20	0,01210	-2,009	52	26
3870733	LILRB2	0,00064	-2,012	45	23
2515627	ITGA6	0,00020	-2,015	27	14
3430086	TCP11L2	0,01400	-2,018	52	26
2692447	MYLK	0,00001	-2,025	85	42
2902427	LST1	0,00246	-2,026	93	46

3917582	KRTAP6-3	0,00058	-2,026	80	39
3974019	TSPAN7	0,00382	-2,028	20	10
3357279	VPS26B	0,00097	-2,029	64	31
3841357	LILRA2	0,00283	-2,029	40	20
3307939	ABLIM1	0,00003	-2,031	51	25
3046681	TARP	0,01946	-2,035	27	13
3749654		0,01958	-2,038	25	12
3902552	FOXS1	0,00064	-2,038	31	15
2360206	ATP8B2	0,00004	-2,044	49	24
2406375		0,00054	-2,047	82	40
3401704	CCND2	0,02514	-2,048	25	12
2650393	PPM1L	0,01080	-2,049	40	19
3551713		0,01276	-2,049	98	48
3407096	PLEKHA5	0,00019	-2,053	54	26
3362789		0,02779	-2,054	63	30
2969406	SLC22A16	0,00000	-2,057	34	16
3335808		0,00689	-2,057	526	256
2352106	CTTNBP2NL	0,00043	-2,073	30	14
3774504		0,00984	-2,077	81	39
2766122	FLJ13197	0,00547	-2,079	20	10
2901753		0,01889	-2,086	331	159
3088048	NSAP11	0,00372	-2,099	22	11
2793198		0,04573	-2,100	10	5
3361040		0,03732	-2,107	14	7
3624145	DMXL2	0,00094	-2,108	26	12
2790109	ANXA2P1	0,00143	-2,114	21	10
2716713	STK32B	0,00032	-2,116	71	33
3351461	MLL	0,00080	-2,117	93	44
2969886	FYN	0,00509	-2,124	77	36
2746119	SMAD1	0,01409	-2,137	147	69
3264744		0,00053	-2,141	31	14
3594031	TMOD2	0,00017	-2,148	27	13
3619185		0,00718	-2,156	132	61
2599946		0,01893	-2,157	18	8
3402736	PTMS	0,00000	-2,160	100	46
3147508	KLF10	0,00239	-2,163	101	47
2832045		0,02139	-2,164	110	51
3610958	IGF1R	0,00104	-2,166	76	35
3174816	ANXA1	0,03347	-2,168	14	6
2406351		0,01643	-2,178	113	52
2374746	NAV1	0,00011	-2,185	70	32
2776372	WDFY3	0,00027	-2,194	14	6
3925639	NRIP1	0,00010	-2,200	415	188
2735759	MMRN1	0,00000	-2,205	25	11
2786732	MAML3	0,00000	-2,217	39	17
3699870		0,01090	-2,228	28	12
2985026	GPR31	0,04184	-2,231	13	6

2318865		0,02915	-2,232	18	8
3840883	ZNF761	0,00096	-2,248	90	40
2362282	OR10Z1	0,02133	-2,249	17	8
3722331		0,02072	-2,251	25	11
3405032	ETV6	0,00000	-2,263	189	83
3484060	ALOX5AP	0,02326	-2,266	137	60
3453506		0,00718	-2,279	32	14
3130872	DUSP26	0,00018	-2,284	51	22
2616596	ARPP21	0,03303	-2,298	250	109
3802602	CDH2	0,00005	-2,302	31	13
2615060	RBMS3	0,00757	-2,306	16	7
2745899	HHIP	0,00225	-2,314	34	15
2982319	SOD2	0,01076	-2,316	317	137
3327143	RAG1	0,00011	-2,321	73	32
3145149	TP53INP1	0,00332	-2,322	368	159
2931569	AKAP12	0,03062	-2,324	373	160
4047185	CCRL2	0,01526	-2,331	22	9
3556709		0,00073	-2,344	31	13
2362537	FCER1A	0,00580	-2,353	36	15
3429676		0,00131	-2,360	120	51
3713539	FAM18B	0,00009	-2,364	32	13
3825153		0,00963	-2,399	103	43
2318815		0,01237	-2,411	99	41
3096575	HGSNAT	0,00968	-2,426	130	53
3831223		0,03105	-2,427	17	7
3548050	PRO1768	0,00057	-2,434	86	35
2872047	SEMA6A	0,00019	-2,445	49	20
2439373	SPTA1	0,00130	-2,446	48	20
3126504	CSGALNACT1	0,00188	-2,472	39	16
3740638		0,04467	-2,472	71	29
3951136	RPL23AP82	0,00338	-2,475	20	8
2745547	GAB1	0,00653	-2,475	306	123
3923850	KRTAP10-7	0,00481	-2,492	104	42
3945006	H1F0	0,00005	-2,496	70	28
3676747		0,02981	-2,498	25	10
3695107	TK2	0,00086	-2,500	41	16
2608469	ITPR1	0,00006	-2,521	122	48
3335822		0,01189	-2,522	28	11
2395564	SLC2A5	0,00000	-2,534	42	16
3426257	SOCS2	0,00000	-2,553	107	42
3932261	C21orf87	0,00033	-2,553	28	11
2340529	PDE4B	0,00579	-2,556	122	48
2849992	FAM134B	0,00001	-2,580	56	22
3852944	OR7C1	0,00114	-2,591	14	5
3435192	MLXIP	0,00001	-2,606	124	48
2665199	SATB1	0,00011	-2,607	141	54
3126191	PSD3	0,03270	-2,616	85	33

3286792	C10orf25	0,00959	-2,628	60	23
3569814	ACTN1	0,00000	-2,681	80	30
2491676	VAMP5	0,00003	-2,714	78	29
2777564	FAM13A	0,00006	-2,722	44	16
3126368	PSD3	0,00613	-2,726	35	13
2886595	LCP2	0,00000	-2,728	81	30
4014387	RPSAP15	0,02907	-2,734	35	13
3956589	XBP1	0,00001	-2,735	221	81
3767169	LRRC37A3	0,00208	-2,736	22	8
2784687	ANKRD50	0,00044	-2,740	264	96
3742415 3742428	SPAG7	0,00206	-2,753	209	76
3825154		0,00959	-2,756	23	8
2444283	TNFSF4	0,00015	-2,766	38	14
3677177		0,01250	-2,810	834	297
2840652		0,00760	-2,850	85	30
2723997	KLF3	0,00012	-2,853	168	59
2701033	P2RY14	0,00051	-2,874	101	35
3383227	GAB2	0,00001	-2,887	193	67
3517251	DACH1	0,00000	-2,890	52	18
3928590	KRTAP21-1	0,01662	-2,922	24	8
2833623	HMHB1	0,00219	-2,960	580	196
2536965	FLJ38379	0,00296	-2,986	159	53
3749606		0,03037	-3,018	42	14
3254609	SH2D4B	0,00005	-3,024	62	20
2742224	SPRY1	0,00002	-3,133	150	48
2633256	ST3GAL6	0,00019	-3,156	35	11
3441849	TNFRSF1A	0,00000	-3,177	85	27
3819749		0,01137	-3,193	51	16
3937967	FLJ26056	0,03637	-3,279	56	17
3230397	LCN6	0,00038	-3,338	92	28
3264621	TCF7L2	0,00000	-3,479	126	36
2542972	NDUFAF2	0,02384	-3,490	7	2
2375706	ATP2B4	0,00000	-3,530	123	35
3811000	RNF152	0,00001	-3,543	65	18
3226138	AK1	0,00004	-3,555	72	20
2678714	FHIT	0,00000	-3,558	219	61
3966225	RABL2B	0,00156	-3,779	92	24
2421995	GBP4	0,00004	-3,969	128	32
3852880	EMR2	0,00000	-4,168	66	16
2571510	IL1B	0,00000	-4,451	74	17
3993360	SPANXB1	0,02237	-4,455	53	12
3046556	TARP	0,00022	-4,458	76	17
2679014	NPCDR1	0,00014	-4,519	108	24
2453307	CD34	0,00000	-4,600	85	18
3427098	ELK3	0,00000	-4,650	221	47
2649824	SCHIP1	0,00002	-4,678	136	29
3031533	GIMAP4	0,00016	-4,978	70	14

3259503	DNTT	0,00215	-5,234	727	139
3766796	PECAM1	0,00000	-5,515	271	49
3624513	MYO5C	0,00000	-5,670	65	11
3012978	GNG11	0,00098	-5,928	20	3
3301782	OPALIN	0,00000	-6,078	78	13
3931765	ERG	0,00001	-6,940	365	53

Adult PreBII s vs PreBII L

Transcript Cluster	Gene Symbol	p-value	fold change	Mean signal<br>PreBII L	 PreBII s
2844381		0,00994	6,121	7	43
2841330		0,01957	4,493	17	77
3825154		0,00080	3,550	8	30
3819749		0,01045	2,970	16	47
3012978	GNG11	0,02272	2,949	3	10
3749652		0,01795	2,751	6	15
3749634		0,02882	2,715	9	24
3353876	OR10G9	0,00006	2,630	23	61
2557641		0,02421	2,594	16	40
3046556	TARP	0,00698	2,591	17	44
3337740		0,00229	2,580	5	12
2678714	FHIT	0,00015	2,424	61	149
3392906		0,01168	2,419	9	21
3699870		0,00335	2,390	12	30
3430086	TCP11L2	0,00210	2,315	26	60
3931765	ERG	0,01685	2,271	53	119
2679014	NPCDR1	0,01588	2,266	24	54
2733483	BMP3	0,00063	2,241	64	144
3928590	KRTAP21-1	0,04848	2,235	8	18
3226138	AK1	0,00315	2,187	20	44
3031533	GIMAP4	0,03098	2,178	14	31
3335842		0,03727	2,126	178	379
2362282	OR10Z1	0,02137	2,117	8	16
2362537	FCER1A	0,00870	2,113	15	32
3766796	PECAM1	0,00643	2,097	49	103
2968456		0,04695	2,068	6	13
3301782	OPALIN	0,00649	2,037	13	26
2571510	IL1B	0,00387	2,035	17	34
3335820		0,04773	2,035	17	35
2844299		0,02104	2,034	10	20
2784687	ANKRD50	0,00608	2,008	96	194
3811000	RNF152	0,00281	2,008	18	37
2557633		0,00371	2,003	153	306
2840682		0,04065	-2,001	82	41

3527514	PNP	0,00150	-2,003	89	44
3475020		0,03264	-2,014	92	46
3556202		0,04323	-2,019	19	9
3168508	MELK	0,00672	-2,031	101	50
2775532		0,01218	-2,031	20	10
3658925	ORC6L	0,00048	-2,035	171	84
3409330	MRPS35	0,00037	-2,038	88	43
2901687 2901714	ABCF1	0,02001	-2,040	38	19
3565663	DLGAP5	0,00975	-2,052	235	115
2908486		0,00229	-2,053	557	271
2828135	LYRM7	0,04199	-2,055	79	38
2968373		0,01059	-2,058	39	19
2758686	LYAR	0,00797	-2,061	70	34
3212300		0,04133	-2,065	296	143
3629103	KIAA0101	0,02560	-2,073	298	144
2521479	HSPE1	0,01997	-2,087	66	32
3577256	C14orf142	0,04547	-2,088	69	33
3844035		0,03107	-2,089	48	23
3556258		0,00554	-2,089	46	22
3513995	DLEU2	0,00405	-2,092	28	14
3464983	ATP2B1	0,00038	-2,095	118	56
3311157	OAT	0,00197	-2,099	60	29
2838656	HMMR	0,02449	-2,101	161	77
3066436	PUS7	0,00063	-2,102	17	8
2914777	TTK	0,00632	-2,105	55	26
2780172	CENPE	0,00599	-2,106	75	35
3428845	C12orf48	0,00051	-2,108	23	11
3672489	IRF8	0,00029	-2,115	55	26
2674355		0,04603	-2,116	40	19
3175971	PSAT1	0,00751	-2,120	29	13
2823880	CAMK4	0,01058	-2,126	73	34
3819602		0,03323	-2,129	253	119
2772566	IGJ	0,02978	-2,130	372	175
3827218	RPSAP58	0,04117	-2,133	32	15
3556292		0,02530	-2,134	76	36
2450345	KIF14	0,00145	-2,145	84	39
2968416		0,03765	-2,150	22	10
3555521	GAFA1	0,00800	-2,153	28	13
3699757 3699779	KARS	0,02005	-2,157	112	52
3515965	DIAPH3	0,00266	-2,158	79	36
2840712		0,03844	-2,160	61	28
3556194		0,04627	-2,169	47	22
3676692		0,00763	-2,169	201	93
3593575	SLC27A2	0,00030	-2,184	28	13
3743512		0,03551	-2,193	49	22
2417528	DEPDC1	0,01552	-2,204	49	22
3556058		0,01889	-2,207	12	5

3556080		0,01968	-2,208	8	3
3419807	XPOT	0,00116	-2,212	39	18
4052881	FAM72D	0,00382	-2,222	112	50
3260586	SCD	0,00239	-2,224	18	8
3013952	ACN9	0,00293	-2,239	57	26
2844409		0,03651	-2,245	15	7
3291435	RTKN2	0,00225	-2,264	54	24
2831968		0,01340	-2,265	529	234
3362719▯3362739	EIF4G2	0,01653	-2,290	126	55
2840660		0,00916	-2,290	127	56
3335774▯3335789	SART1	0,00233	-2,295	47	20
3625761	MNS1	0,00306	-2,297	89	39
3389450	CASP1	0,02792	-2,327	118	51
3434393	DYNLL1	0,02481	-2,334	396	170
3669552	VAT1L	0,00017	-2,336	98	42
3505319	SACS	0,00062	-2,345	47	20
3749622		0,02679	-2,353	15	7
3840642	ZNF818P	0,01140	-2,358	20	8
3855100		0,00082	-2,359	26	11
3248289	CDK1	0,00464	-2,367	50	21
2766192	TLR10	0,00882	-2,367	122	52
2557567		0,00494	-2,372	114	48
3819543▯3819609	HNRNPM	0,04201	-2,376	166	70
3556036		0,01889	-2,381	37	16
3556668		0,03872	-2,383	16	7
3973839	CYBB	0,01285	-2,389	54	22
3725868		0,04492	-2,401	13	5
2729667	STAP1	0,00149	-2,402	77	32
3676682		0,01559	-2,406	46	19
2874686▯2874696	HINT1	0,01206	-2,409	164	68
2363618	SDHC	0,03195	-2,435	26	11
3556162		0,00393	-2,442	13	5
3298738	WAPAL	0,01383	-2,451	159	65
2737596	BANK1	0,00001	-2,468	95	39
3253461		0,04587	-2,492	18	7
3275588		0,03802	-2,494	44	18
3630197		0,04661	-2,501	12	5
3708826	EIF4A1	0,00975	-2,512	128	51
4013828	HMGN5	0,00010	-2,514	58	23
2514563	KLHL23	0,01220	-2,515	52	21
2320064		0,02292	-2,530	281	111
2686775		0,04398	-2,535	175	69
2496536	RPL31	0,00508	-2,536	84	33
3556100		0,02546	-2,590	20	8
3551784		0,03009	-2,594	36	14
3556138		0,01145	-2,602	76	29
3129149	PBK	0,00746	-2,606	97	37

3809671 3809680	NARS	0,00349	-2,607	109	42
3197955	GLDC	0,00042	-2,643	153	58
3551868		0,04942	-2,696	26	10
2523632		0,01575	-2,696	110	41
2901699		0,00664	-2,715	77	28
3980922		0,02170	-2,719	10	4
3427847		0,03309	-2,728	65	24
2840674		0,02318	-2,752	24	9
3288013	BMS1P1	0,04136	-2,781	11	4
3809671 3809692	NARS	0,01075	-2,792	23	8
3279058	ACBD7	0,00882	-2,792	31	11
3742627	C17orf87	0,00172	-2,794	27	10
2523540 2523572	NBEAL1	0,01711	-2,816	13	4
2328575		0,02500	-2,847	43	15
3135567	LYPLA1	0,03078	-2,873	38	13
2674361		0,01987	-2,904	40	14
3275166		0,00441	-2,907	218	75
2468622	ID2	0,00801	-3,003	137	46
3958307		0,02092	-3,005	73	24
2565221		0,00114	-3,024	28	9
2908487		0,01017	-3,031	133	44
2328555		0,00823	-3,061	83	27
2840688		0,01258	-3,073	96	31
2674494		0,03373	-3,088	24	8
3472089	RPL6	0,00089	-3,090	35	11
2328565		0,01245	-3,111	23	7
3470322		0,02458	-3,115	56	18
2844226		0,01440	-3,139	37	12
2993658		0,00206	-3,193	34	11
3470398		0,00719	-3,252	23	7
3362719 3362765	EIF4G2	0,00202	-3,375	23	7
3980934		0,01534	-3,552	33	9
2674456		0,00103	-3,582	21	6
2565211		0,00613	-3,692	38	10
3253459		0,00173	-3,694	12	3
2383896		0,02272	-3,780	17	5
2844293		0,01535	-3,794	16	4
3904691	SAMHD1	0,00005	-3,870	113	29
2968289		0,03344	-3,963	12	3
3556290		0,00056	-4,006	34	9
3275574		0,00074	-4,304	17	4
3470394		0,00894	-4,356	55	13
3306591		0,00192	-4,619	27	6
3335844		0,01091	-4,916	35	7
3980910		0,00345	-4,989	22	4
3980926		0,00043	-5,083	47	9

Adult Immature B vs PreBII s

Transcript Cluster	Gene Symbol	p-value	fold change	Mean signal PreBII s	Mean signal Immature B
2531233	SP140	0,00000	16,997	8	142
3973839	CYBB	0,00000	10,506	22	236
2445982	ANGPTL1	0,00000	7,374	18	131
3346548	BIRC3	0,00000	7,344	26	189
3384321	RAB30	0,00000	7,062	46	327
2369339	RALGPS2	0,00000	7,061	38	265
2688717	BTLA	0,00000	6,957	28	193
2968289		0,00412	6,827	3	21
2968363		0,00129	6,251	5	33
3332403	MS4A1	0,00000	6,194	77	478
3815399	CNN2	0,00000	5,938	20	119
2439052	FCRL2	0,00000	5,866	16	92
3672489	IRF8	0,00000	5,855	26	152
2968438		0,00158	5,815	3	19
2792166		0,00000	5,548	13	74
2439101	FCRL1	0,00000	5,538	62	345
2895945		0,00000	5,281	11	58
3442854	SLC2A3	0,00000	5,239	23	120
3329983	PTPRJ	0,00000	5,236	39	206
2406291		0,01130	5,191	6	33
3335844		0,00964	5,066	7	36
2950145	HLA-DOB	0,00000	5,061	32	161
2766192	TLR10	0,00002	4,904	52	253
2377283	CR2	0,00000	4,742	6	26
3013255	PEG10	0,00000	4,660	25	116
2366884	C1orf129	0,00004	4,631	14	63
2737596	BANK1	0,00000	4,629	39	179
2968444		0,00002	4,597	16	73
2968418		0,03669	4,511	19	87
3268274	PLEKHA1	0,00000	4,391	18	78
3831229		0,00845	4,355	5	22
2764192	SEL1L3	0,00000	4,290	11	46
2968285		0,00188	4,241	26	108
2353988	FAM46C	0,00000	4,170	25	105
2638988	PARP15	0,00001	4,062	49	197
2968357		0,00273	4,057	9	38
3926271	TMPRSS15	0,00000	4,050	4	14
3253452		0,01246	4,032	5	20
3742627	C17orf87	0,00006	4,011	10	39
2968448		0,04828	3,964	11	45
3291601	EGR2	0,00001	3,949	28	112

2968287		0,00883	3,929	42	164
2557651		0,00568	3,895	6	22
3958341		0,00132	3,847	10	39
2968416		0,00067	3,817	10	40
3718930	CCL4	0,00020	3,809	6	25
2734421	ARHGAP24	0,00000	3,746	14	52
2968281		0,00004	3,693	32	117
3151970	MTSS1	0,00000	3,611	58	211
2968295		0,00068	3,547	180	638
3374934	MS4A6A	0,00000	3,539	31	110
3464983	ATP2B1	0,00000	3,505	56	197
2438892	FCRL5	0,00000	3,456	7	25
3335812		0,01276	3,445	68	234
3470322		0,01519	3,443	18	62
3982560	P2RY10	0,00000	3,418	19	67
2968343		0,00919	3,404	11	36
4013460	CYSLTR1	0,00000	3,359	24	80
2582124	NR4A2	0,00032	3,325	14	47
2674309		0,00717	3,315	3	11
3392902		0,00705	3,295	9	31
3090209	ADAM28	0,00318	3,275	10	32
2508520	KYNU	0,00009	3,267	5	15
2362201	CD1C	0,00000	3,262	12	38
2860178	CD180	0,00002	3,222	52	167
2536478		0,00357	3,217	70	224
3173673	PIP5K1B	0,00000	3,217	31	100
2523540?523572	NBEAL1	0,00869	3,163	4	14
3250650		0,02111	3,147	6	18
3824874	IFI30	0,00000	3,132	14	43
2766289	TMEM156	0,00027	3,117	19	60
3244622	ALOX5	0,00000	3,116	31	97
3527662	RNASE6	0,00042	3,098	17	54
3392900		0,00599	3,080	18	56
2968373		0,00020	3,077	19	58
2844339		0,00127	3,070	11	33
3740618		0,00334	3,066	6	18
3840642	ZNF818P	0,00144	3,060	8	26
3556668		0,01029	3,006	7	21
2664209	SH3BP5	0,00000	3,001	28	85
2968377		0,00004	2,980	133	398
2565211		0,02012	2,964	10	30
2901751		0,01202	2,963	5	16
2388085	KMO	0,00002	2,961	50	147
3444503	TAS2R31	0,02239	2,950	14	40
3430125		0,00287	2,946	6	17
4017212	MORC4	0,00337	2,944	10	29
3208355	CBWD3	0,02236	2,944	8	22

2830861	EGR1	0,00000	2,930	47	138
2840654		0,01984	2,927	21	62
3306591		0,02435	2,902	6	17
2536422		0,00738	2,894	8	22
3319997	SWAP70	0,00000	2,863	100	285
3275200		0,02160	2,860	6	16
2949091		0,04298	2,852	9	27
3098977	LYN	0,00035	2,840	126	357
3556290		0,00689	2,833	9	24
4044363	CNR2	0,00005	2,829	31	87
3959720		0,02602	2,796	7	19
2600689	EPHA4	0,00002	2,793	13	36
3662387	HERPUD1	0,00000	2,758	99	273
2729667	STAP1	0,00035	2,749	32	88
3403595	CLEC4A	0,00000	2,734	23	62
2531310	SP140L	0,00000	2,721	61	166
2766262	TLR6	0,00030	2,708	8	21
2868265	LIX1	0,00005	2,706	7	19
3725916		0,02786	2,702	26	71
2523540 2523575	NBEAL1	0,03857	2,698	7	20
3061805	SGCE	0,00000	2,690	11	28
3375648	FTH1	0,00158	2,677	33	90
3725854		0,03678	2,659	14	37
3819701		0,00167	2,658	41	109
4010152	LOC442454	0,04277	2,647	25	66
2790062	TMEM154	0,00010	2,640	8	20
3755903	GSDMB	0,00022	2,593	43	111
2674456		0,01105	2,583	6	15
3699884		0,03184	2,575	4	11
2968293		0,02859	2,568	10	26
3451375	PRICKLE1	0,00021	2,537	21	54
2383781		0,00577	2,534	6	16
3944690	CYTH4	0,00000	2,532	25	64
2518583	DNAJC10	0,00001	2,529	65	165
2792459	GK3P	0,02499	2,517	8	21
2423017	EVI5	0,00206	2,511	23	58
3556138		0,01476	2,506	29	73
3060117	ABCB4	0,00000	2,500	23	58
2373842	PTPRC	0,00000	2,486	215	534
3837731	EMP3	0,00004	2,484	56	138
3470398		0,03384	2,481	7	18
2531377	SP100	0,00000	2,480	73	180
3154263	SLA	0,00004	2,475	24	60
3838385	CD37	0,00217	2,474	104	257
2476671	RASGRP3	0,00000	2,459	8	20
3484060	ALOX5AP	0,00806	2,458	82	202
2974413	MOXD1	0,00000	2,458	7	16

2712147	PPP1R2	0,00541	2,445	51	126
3275574		0,02862	2,439	4	9
3474553		0,02248	2,435	57	139
2984884	RNASET2	0,00014	2,433	66	160
3443891	CLEC2B	0,00016	2,427	47	114
2601287	AP1S3	0,00002	2,422	11	26
3340697	UVRAG	0,00001	2,422	82	199
2968257		0,01471	2,417	21	51
2363689	FCGR2A	0,00008	2,416	7	17
3969081	TLR7	0,00778	2,406	19	45
3822849	CLEC17A	0,00000	2,389	27	65
3222144	TNFSF8	0,00043	2,385	24	58
2482944		0,02152	2,374	9	21
3418086		0,00325	2,372	34	81
2557659		0,03794	2,368	12	29
2841699	CPEB4	0,00000	2,368	42	100
2406289		0,02096	2,363	9	22
2974671	C6orf192	0,00012	2,358	31	74
3748400	USP6	0,00941	2,351	131	309
3940631	ADRBK2	0,00001	2,349	62	146
3141857	TPD52	0,00000	2,342	56	131
2731542	AREG	0,00409	2,336	11	27
3755862	IKZF3	0,00009	2,335	112	261
3181976	NR4A3	0,00001	2,333	13	30
2351854	C1orf162	0,00014	2,325	62	144
2973376	PTPRK	0,00000	2,324	12	28
2593464	ANKRD44	0,00000	2,316	56	130
3474555		0,01591	2,314	19	44
2737318	DAPP1	0,00003	2,308	26	60
3403092	PTPN6	0,00000	2,301	113	259
3061456	SAMD9L	0,00035	2,299	31	72
2968283		0,03341	2,297	21	47
3699854		0,00418	2,289	13	31
2982076	TAGAP	0,00000	2,288	43	99
3333595	GNG3	0,00002	2,285	22	50
2377229	CD55	0,00001	2,274	216	491
2434575	CTSS	0,00004	2,270	55	126
2968379		0,01227	2,267	65	147
3449760	DENND5B	0,00000	2,247	9	21
2774971	ANTXR2	0,00050	2,244	53	118
3212360		0,00135	2,244	61	137
2991395	HDAC9	0,00000	2,234	49	109
2486811	PLEK	0,00054	2,231	8	17
2363808	FCGR2B	0,00001	2,228	26	59
3629811	DENND4A	0,00000	2,228	56	124
3350775	SIDT2	0,00000	2,221	51	114
2968232?2968247	SNX3	0,00375	2,211	88	194

3699864		0,00189	2,202	22	48
2565343		0,03370	2,199	6	14
3335808		0,00182	2,198	254	558
3556258		0,00336	2,194	22	48
2912649	COL19A1	0,00000	2,189	8	18
3410384	C12orf35	0,00003	2,180	158	345
2968393		0,00101	2,179	83	180
3741547	P2RX5	0,00161	2,174	36	78
3286895	OR13A1	0,00747	2,171	12	26
2563785	IGK@	0,00241	2,169	494	1071
2326463	CD52	0,00000	2,166	550	1191
2939014	MGC39372	0,00021	2,162	40	87
3959688		0,02006	2,159	20	42
3556288		0,00655	2,154	37	79
3626826	MYO1E	0,00019	2,151	9	19
2895841	CD83	0,00001	2,143	50	108
3362795	RNF141	0,00000	2,143	70	151
2536424		0,00106	2,141	53	113
3749622		0,04733	2,138	7	14
3449910	AMN1	0,00027	2,137	51	109
2589868	CCDC141	0,00021	2,136	31	66
2968420		0,00010	2,133	52	111
2574855		0,00818	2,132	45	96
3522662	GPR183	0,00021	2,125	33	71
2603051	SP110	0,00000	2,125	168	358
3375307	CYBASC3	0,00000	2,124	35	75
2452977	FAIM3	0,00068	2,121	184	390
3740624		0,03472	2,115	13	27
2613293	KCNH8	0,00000	2,114	8	16
2423175	FAM69A	0,00011	2,110	28	59
3742415 3742426	SPAG7	0,03421	2,101	23	48
2574798	MAP3K2	0,00017	2,098	42	89
3699860		0,01135	2,096	77	161
2536380		0,04357	2,094	13	27
2667809	OSBPL10	0,00000	2,086	25	52
2320048 2320077	TARDBP	0,03992	2,083	39	82
3922100	MX1	0,01938	2,078	38	78
2903219		0,02472	2,077	47	97
3740201	MYO1C	0,00000	2,075	33	68
2325214		0,03545	2,067	22	45
3401828	DYRK4	0,00001	2,065	19	40
2968269		0,02973	2,058	6	12
3917305	BACH1	0,00003	2,050	56	114
2428855	AP4B1	0,00035	2,047	46	94
2841342		0,04544	2,045	28	57
3212356		0,01355	2,044	50	102
2883440	ADAM19	0,00000	2,042	52	106

2766492	C4orf34	0,00002	2,039	192	392
3839360	MYBPC2	0,00001	2,036	18	37
2536462		0,00003	2,035	204	416
3722321		0,04412	2,031	30	60
2949971	C6orf10	0,00081	2,029	9	18
2689286	KIAA1407	0,00010	2,029	64	130
3752258	EVI2B	0,00008	2,028	96	194
2355591		0,01382	2,026	16	33
2523354	FAM117B	0,00001	2,023	60	122
3471327	HVCN1	0,00003	2,023	29	59
3648412	RUNDC2A	0,00098	2,022	26	53
2378662	TRAF5	0,00003	2,021	25	50
2810764	GAPT	0,00019	2,020	58	117
2536518		0,00350	2,014	37	75
2536428		0,00730	2,011	45	90
2478748	EML4	0,00001	2,007	55	111
2598099	BARD1	0,00003	-2,001	137	68
3474951		0,03457	-2,003	20	10
3625539	NEDD4	0,00209	-2,004	20	10
3699044	RFWD3	0,00004	-2,004	70	35
2974330	CTGF	0,01608	-2,004	67	34
3689880	SHCBP1	0,00200	-2,006	80	40
2878446 2878455	NDUFA2	0,00003	-2,007	717	358
2838656	HMMR	0,03365	-2,009	77	38
3651018	CP110	0,00079	-2,012	76	38
3736290	BIRC5	0,00003	-2,013	112	55
3773312	EIF4A3	0,00013	-2,014	165	82
3770563	ATP5H	0,01568	-2,016	30	15
3617403	NOP10	0,00004	-2,016	243	121
3980917		0,00039	-2,019	181	90
3804358	C18orf10	0,00011	-2,021	128	63
2332144	CTPS	0,00025	-2,022	145	72
2796510	MLF1IP	0,02487	-2,023	107	53
3096448		0,00840	-2,024	204	101
2775463 2775500	HNRNPD	0,00000	-2,025	622	307
3150715	DSCC1	0,00605	-2,026	46	23
2742985	PLK4	0,00473	-2,026	54	27
3427820 3427838	SLC25A3	0,00002	-2,028	589	291
3203855	DCAF12	0,00174	-2,032	121	60
3955815	HPS4	0,00006	-2,033	101	49
2763278	GPR125	0,00142	-2,033	62	30
3651478	ACSM3	0,01238	-2,034	92	45
3289031	TIMM23	0,00169	-2,035	280	138
2436145		0,00018	-2,039	287	141
3975893	PHF16	0,00002	-2,040	49	24
3373845	SLC43A3	0,00001	-2,041	69	34
4052881	FAM72D	0,00852	-2,047	50	25

3427098	ELK3	0,00825	-2,047	91	44
2901913	TUBB	0,00057	-2,049	1208	590
3151534	ATAD2	0,00179	-2,050	79	39
3371719	CKAP5	0,00005	-2,050	162	79
3168508	MELK	0,00609	-2,051	50	24
3335785		0,00988	-2,051	124	60
36997573699776	KARS	0,02670	-2,053	13	7
3417309	PA2G4	0,00001	-2,054	2497	1216
2521479	HSPE1	0,02254	-2,054	32	15
3803120	B4GALT6	0,00402	-2,054	41	20
3434562	UNC119B	0,00223	-2,061	154	75
24361322436147	ILF2	0,00001	-2,062	707	343
3290746	SLC16A9	0,00945	-2,063	45	22
2565283		0,01026	-2,064	28	14
3985523	WBP5	0,00310	-2,064	36	17
2436157		0,00019	-2,066	666	322
3821908	RNASEH2A	0,00000	-2,067	115	56
3258444	CEP55	0,00136	-2,074	30	14
2511820	PKP4	0,00001	-2,079	62	30
3236448	SUV39H2	0,00077	-2,079	51	25
2491661	VAMP8	0,00000	-2,080	924	444
3719962	PSMB3	0,00434	-2,081	107	51
2914777	TTK	0,00692	-2,086	26	13
3046556	TARP	0,03264	-2,087	44	21
2827185	LMNB1	0,00018	-2,089	277	132
3943207	YWHAH	0,00005	-2,089	367	176
3367036	CCDC34	0,00031	-2,090	67	32
3145107	CCNE2	0,00505	-2,093	68	32
2364438	NUF2	0,01584	-2,096	100	48
3264621	TCF7L2	0,00059	-2,096	54	26
3060245	SLC25A40	0,00649	-2,104	35	17
3335798		0,01186	-2,106	80	38
2735221	PKD2	0,00013	-2,108	109	51
3563395	POLE2	0,00149	-2,113	15	7
2444790	MRPS14	0,00368	-2,115	94	44
24361322436156	ILF2	0,00021	-2,120	413	195
3896200	PCNA	0,00052	-2,121	189	89
3253683	ZMIZ1	0,00000	-2,125	69	32
3318009	RRM1	0,00027	-2,132	120	56
3765580	BRIP1	0,00012	-2,134	76	36
3590388	NUSAP1	0,00270	-2,134	418	196
2946268	HIST1H2BC	0,03715	-2,135	225	105
2439944	PIGM	0,00051	-2,139	32	15
3078348	EZH2	0,00022	-2,140	194	90
23838592383890	GUK1	0,00347	-2,140	112	52
3275218		0,01440	-2,140	197	92
3303255	ERLIN1	0,00769	-2,142	72	34

2838598	CCNG1	0,00001	-2,142	90	42
2946353	HIST1H1D	0,00165	-2,143	115	54
3875642	PLCB1	0,00005	-2,143	30	14
2353773	TTF2	0,00016	-2,144	62	29
3015769	POP7	0,00024	-2,144	66	31
3984655	CENPI	0,00071	-2,146	27	13
3980907		0,00000	-2,146	784	365
2784113	CCNA2	0,00189	-2,148	108	50
3340269	POLD3	0,00052	-2,148	187	87
3703112	GINS2	0,00009	-2,152	116	54
2867836	GLRX	0,00246	-2,153	68	32
2406420	CLSPN	0,01130	-2,158	119	55
2714955	TACC3	0,00021	-2,160	106	49
3126368	PSD3	0,02037	-2,161	17	8
2954022	TRERF1	0,00346	-2,163	18	8
3286792	C10orf25	0,02293	-2,170	40	18
4008427	NUDT11	0,01040	-2,170	59	27
3570049 3570052	ERH	0,00004	-2,171	318	147
3759335	GJC1	0,00739	-2,176	51	23
3554592	BTBD6	0,00058	-2,176	102	47
2901333	ZNRD1	0,00134	-2,176	105	48
2796066	RWDD4A	0,03071	-2,176	62	28
3565571	WDHD1	0,00129	-2,178	118	54
2436194		0,04905	-2,178	43	20
2521278	CCDC150	0,00000	-2,181	21	10
3080283	XRCC2	0,00135	-2,189	44	20
3744263	AURKB	0,00001	-2,191	122	56
2620256	KIF15	0,00654	-2,195	98	45
2965739	C6orf167	0,00076	-2,198	37	17
3390542	RDX	0,00049	-2,199	69	31
3599811	KIF23	0,00081	-2,200	77	35
3258910	HELLS	0,00172	-2,201	111	50
3902489	BCL2L1	0,00006	-2,201	133	61
3587457	ARHGAP11A	0,00546	-2,201	68	31
3301782	OPALIN	0,00287	-2,202	26	12
3740479 3740494	PRPF8	0,00001	-2,207	383	174
3677176		0,00004	-2,209	418	189
2748830	GUCY1A3	0,00003	-2,211	39	18
3427014	SNRPF	0,00000	-2,211	460	208
2941721	ELOVL2	0,00524	-2,216	23	10
2735598	TIGD2	0,00001	-2,220	29	13
3468301	PMCH	0,00669	-2,221	10	4
3507199	FLT3	0,02525	-2,227	42	19
3722060	VPS25	0,00273	-2,227	124	56
2798538	SDHA	0,00006	-2,227	218	98
3980889		0,00244	-2,230	392	176
2899090	HIST1H3A	0,01210	-2,238	35	16

2946208	HIST1H4B	0,00148	-2,241	1233	550
3382861	PAK1	0,00011	-2,241	89	40
2652675	ECT2	0,00152	-2,243	41	18
3980893		0,00871	-2,245	104	46
3419471 3419474	RPL14	0,00198	-2,247	173	77
2859667	CENPK	0,00973	-2,252	96	43
3091077	DPYSL2	0,00001	-2,253	124	55
2516023	CDCA7	0,00006	-2,253	59	26
3258168	KIF11	0,01120	-2,267	93	41
3607510	FANCI	0,00066	-2,269	179	79
2411228	STIL	0,00082	-2,270	36	16
3589697	BUB1B	0,00037	-2,271	166	73
3753568	SLFN13	0,00005	-2,276	132	58
3504617	SKA3	0,00188	-2,279	54	24
2358693	MLLT11	0,00180	-2,282	105	46
3182957	NIPSNAP3A	0,00020	-2,297	26	11
2682088	EIF4E3	0,00002	-2,301	68	30
2395490	ENO1	0,00003	-2,303	310	135
3756193	TOP2A	0,00174	-2,308	323	140
3706753	GSG2	0,00005	-2,310	47	21
3407096	PLEKHA5	0,00001	-2,313	31	13
3458133	PRIM1	0,00020	-2,314	70	30
3928590	KRTAP21-1	0,03997	-2,316	18	8
3183111	SLC44A1	0,00079	-2,317	73	31
3744965	GAS7	0,00000	-2,320	251	108
2709486	RFC4	0,00121	-2,330	123	53
2784687	ANKRD50	0,00119	-2,331	194	83
2345929	LRRC8C	0,00000	-2,331	209	90
2783715	MAD2L1	0,01074	-2,338	114	49
2659560	PIGX	0,00271	-2,343	45	19
3178583	CKS2	0,00026	-2,347	216	92
3565663	DLGAP5	0,00263	-2,350	115	49
2469252	RRM2	0,00239	-2,354	1311	557
3920003	CHAF1B	0,00009	-2,355	65	28
3980903		0,02580	-2,359	44	19
2900116	HIST1H2BO	0,00618	-2,360	283	120
3454223	RACGAP1	0,00000	-2,360	264	112
2957126	MCM3	0,00000	-2,361	161	68
2468622	ID2	0,03367	-2,365	46	19
2449559	ASPM	0,00285	-2,369	102	43
3235789	MCM10	0,00018	-2,375	56	23
3720896	CDC6	0,00403	-2,377	126	53
3365776	E2F8	0,00098	-2,378	91	38
3538893	PRKCH	0,00000	-2,394	60	25
3401804	RAD51AP1	0,00009	-2,394	255	106
2678714	FHIT	0,00018	-2,395	149	62
3463112	E2F7	0,00002	-2,405	69	29

2379863	CENPF	0,00470	-2,406	258	107
3624513	MYO5C	0,00042	-2,411	20	8
3728964	PRR11	0,00017	-2,414	70	29
3354799	CHEK1	0,00086	-2,419	124	51
3275334		0,03531	-2,420	11	4
3031533	GIMAP4	0,01533	-2,421	31	13
2520225	NAB1	0,00002	-2,422	37	15
2748163	MND1	0,01012	-2,433	174	71
2946319	HIST1H4D	0,01280	-2,434	53	22
2570616	BUB1	0,00011	-2,443	89	37
2679014	NPCDR1	0,00906	-2,443	54	22
3932131	PSMG1	0,00034	-2,446	110	45
2911372	BAG2	0,00050	-2,460	30	12
2947081	HIST1H4L	0,00518	-2,469	547	222
2319802	PGD	0,00001	-2,474	113	46
3327143	RAG1	0,00001	-2,475	55	22
3312490	MKI67	0,00050	-2,480	387	156
2565262?565284	SNRNP200	0,00162	-2,485	138	56
2494484	NCAPH	0,00036	-2,486	90	36
3368707	CD59	0,00000	-2,488	78	31
3683018	RPS15A	0,00015	-2,492	305	122
3515965	DIAPH3	0,00051	-2,496	36	15
2428501	SLC16A1	0,00050	-2,501	93	37
3772187	EPR1	0,00010	-2,502	891	356
2946219	HIST1H2AB	0,02742	-2,505	67	27
2947100	HIST1H2AM	0,00616	-2,505	43	17
2388219	EXO1	0,00094	-2,508	79	32
2720251	NCAPG	0,00095	-2,512	183	73
2946369	HIST1H3G	0,00113	-2,519	137	54
3881443	TPX2	0,00048	-2,520	162	64
2396781	MAD2L2	0,00007	-2,521	163	65
2571457	CKAP2L	0,00076	-2,530	95	37
3921391	WRB	0,00001	-2,530	46	18
3817698	UHRF1	0,00002	-2,532	51	20
2830638	KIF20A	0,00030	-2,541	37	14
2383805		0,01822	-2,554	43	17
3590086	RAD51	0,00079	-2,555	97	38
3938792	VPREB1	0,00000	-2,565	387	151
2899768	HIST1H4I	0,00011	-2,568	59	23
2997376	ANLN	0,00071	-2,571	65	25
3715489	TMEM97	0,00047	-2,584	65	25
3428268	GAS2L3	0,00004	-2,587	50	19
3198346	PTPRD	0,00074	-2,589	44	17
3740479?740506	PRPF8	0,00026	-2,593	63	24
3607537	FANCI	0,00004	-2,594	100	39
3219621	CTNNAL1	0,00009	-2,610	51	20
2389130	EFCAB2	0,00415	-2,614	82	31

3980560	KIF4A	0,00002	-2,615	169	65
3829150		0,00014	-2,630	17	6
3749554		0,02694	-2,639	53	20
3775842	TYMS	0,00001	-2,644	696	263
3776139	NDC80	0,00451	-2,650	75	28
2333136	CDC20	0,03055	-2,653	58	22
3811000	RNF152	0,00008	-2,666	37	14
2389789	SCCPDH	0,00000	-2,680	103	38
3595979	CCNB2	0,00555	-2,682	88	33
2417528	DEPDC1	0,00322	-2,684	22	8
2900091	HIST1H2AL	0,00059	-2,701	338	125
2813414	CCNB1	0,00037	-2,704	169	62
3852565	ASF1B	0,00000	-2,712	60	22
3097152	MCM4	0,00002	-2,715	113	42
2454444	NEK2	0,00002	-2,734	93	34
2947073	HIST1H1B	0,00031	-2,735	41	15
3648391	TNFRSF17	0,00478	-2,756	32	12
2899772	HIST1H2AH	0,01304	-2,779	35	12
3960685	DMC1	0,00002	-2,782	15	5
2330773	CDCA8	0,00017	-2,792	60	21
3233049	AKR1C3	0,00025	-2,799	18	6
2975655	FAM54A	0,00374	-2,816	93	33
3003143	MRPS17	0,00604	-2,824	112	40
3936913	CDC45	0,00002	-2,831	143	51
2451200	UBE2T	0,00023	-2,847	107	38
3921068	ETS2	0,00000	-2,853	113	40
3629103	KIAA0101	0,00205	-2,854	144	50
3137875	GGH	0,00027	-2,857	45	16
3764738	SKA2	0,00000	-2,858	251	88
2585933	SPC25	0,00667	-2,860	151	53
2858592	DEPDC1B	0,00044	-2,886	107	37
2900059	HIST1H2BM	0,00106	-2,897	780	269
2838201	PTTG1	0,00002	-2,906	963	331
3707199	PSMB6	0,00134	-2,943	56	19
2947077	HIST1H3I	0,00178	-2,947	737	250
2378937	DTL	0,00007	-2,981	297	100
2709606	RPL39L	0,00008	-2,984	345	116
2604254	HJURP	0,00001	-3,017	99	33
4027585	MPP1	0,00000	-3,033	213	70
2686784		0,04039	-3,051	9	3
2931569	AKAP12	0,00286	-3,061	240	78
3591704	WDR76	0,00010	-3,072	153	50
2946364	HIST1H3F	0,00097	-3,075	474	154
3880827	GINS1	0,00030	-3,142	145	46
2616596	ARPP21	0,00231	-3,158	169	54
2744734	MGST2	0,00000	-3,167	131	41
3536336	CDKN3	0,00013	-3,220	204	63

3306516 3306527	SMNDC1	0,00255	-3,237	44	13
3931765	ERG	0,00096	-3,280	119	36
2577896	MCM6	0,00022	-3,284	196	60
3129149	PBK	0,00116	-3,321	37	11
2833623	HMHB1	0,00033	-3,373	345	102
3369931	RAG2	0,00019	-3,377	115	34
3046708	TRGV3	0,00634	-3,421	18	5
3210808	GNAQ	0,00002	-3,428	164	48
2946215	HIST1H3B	0,00369	-3,476	320	92
2875193	P4HA2	0,00185	-3,531	142	40
2899102	HIST1H3C	0,00180	-3,675	225	61
4016193	TMSB15A	0,02780	-3,805	51	13
2421995	GBP4	0,00002	-3,908	64	16
3259503	DNTT	0,00161	-4,869	325	67

CHILDREN

Child PreBI vs ProB

Transcript Cluster	Gene Symbol	p-value	fold change	Mean signal ProB	Mean signal PreBI
2947040	HIST1H2AJ	0,01349	3,786	4	16
2328527		0,00187	3,542	3	9
3578152	**TCL1A**	0,00007	3,463	15	51
2325224		0,01987	3,397	11	38
4016193	TMSB15A	0,04705	3,309	8	25
2363074	SUMO1P3	0,02287	3,245	7	21
2597867	IKZF2	0,00010	3,158	37	118
3844051		0,01425	3,085	8	24
3755862	IKZF3	0,00000	3,036	9	27
3904691	SAMHD1	0,00060	2,970	8	25
3197955	GLDC	0,00019	2,835	29	82
3129149	PBK	0,00427	2,808	23	64
2680819	SUCLG2	0,00316	2,661	9	23
3536336	CDKN3	0,00094	2,657	95	253
3565663	DLGAP5	0,00078	2,649	70	185
2782545	CAMK2D	0,00103	2,495	20	50
3428268	GAS2L3	0,00008	2,474	21	52
2899772	HIST1H2AH	0,02615	2,473	9	22
3599811	KIF23	0,00019	2,465	45	110
3743515		0,01028	2,434	6	15
2838656	HMMR	0,00846	2,420	56	134
4035833	CD24	0,01402	2,408	69	165
3512948	C13orf18	0,02482	2,406	38	92
3910785	AURKA	0,00243	2,395	27	64

3248289	CDK1	0,00431	2,387	15	35
3881443	TPX2	0,00107	2,350	82	193
2830638	KIF20A	0,00084	2,330	22	52
2954022	TRERF1	0,00167	2,316	16	38
3301218	PDLIM1	0,00004	2,296	68	156
3258168	KIF11	0,01025	2,293	59	136
2858592	DEPDC1B	0,00480	2,261	50	114
2781138	**LEF1**	0,01583	2,258	242	546
3595979	CCNB2	0,01980	2,253	50	112
2449559	ASPM	0,00483	2,243	61	137
2901687?2901723	ABCF1	0,04467	2,223	25	55
3756193	TOP2A	0,00263	2,221	194	430
2450345	KIF14	0,00098	2,215	27	59
3699870		0,00697	2,207	16	34
2813414	CCNB1	0,00350	2,193	115	252
2524653	ADAM23	0,00017	2,182	8	18
2434129	HIST2H2AB	0,00028	2,181	436	952
2784113	CCNA2	0,00165	2,173	62	135
3551778		0,03445	2,171	33	72
2330773	CDCA8	0,00294	2,164	28	60
2620256	KIF15	0,00767	2,157	59	128
3774283	ARHGDIA	0,01615	2,156	20	43
2563785	**IGK@**	0,00273	2,144	38	81
2585933	SPC25	0,04345	2,137	82	175
2946364	HIST1H3F	0,01925	2,135	178	380
3365776	E2F8	0,00323	2,135	41	88
2914777	TTK	0,00557	2,132	18	38
2766192	TLR10	0,02074	2,118	33	70
2343025	AK5	0,00016	2,117	19	41
3980560	KIF4A	0,00042	2,112	89	189
2417528	DEPDC1	0,02182	2,106	16	35
2364438	NUF2	0,01527	2,105	64	134
3312490	MKI67	0,00342	2,095	189	395
2878461	DND1	0,03999	2,093	31	64
3587457	ARHGAP11A	0,00876	2,093	46	97
2388085	KMO	0,00201	2,087	9	18
3474541		0,04000	2,081	41	85
3589697	BUB1B	0,00120	2,076	103	214
2722377	STIM2	0,00009	2,057	61	125
3776139	NDC80	0,03048	2,054	47	96
2604254	HJURP	0,00130	2,050	46	95
3258444	CEP55	0,00164	2,044	19	40
2997376	ANLN	0,00770	2,042	46	93
3699757?3699771	KARS	0,02936	2,037	33	66
2780172	CENPE	0,00958	2,007	29	58
2730673	MOBKL1A	0,00349	-2,005	60	30
3725982		0,00983	-2,024	29	14

3422855	GLIPR1	0,00532	-2,040	247	121
2591421	TFPI	0,00172	-2,047	11	6
2358117	C1orf54	0,00108	-2,055	69	33
3556080		0,03150	-2,065	13	6
2391532	CCNL2	0,00173	-2,071	107	52
3819659		0,01611	-2,075	166	80
2665199	SATB1	0,00091	-2,082	290	139
3233049	AKR1C3	0,00584	-2,085	27	13
2701033	P2RY14	0,00583	-2,103	156	74
3577078	LGMN	0,01925	-2,109	115	55
3326635	CD44	0,00325	-2,131	74	35
2735759	MMRN1	0,00000	-2,153	38	18
2968375		0,01739	-2,155	32	15
3214451	NFIL3	0,01113	-2,165	47	22
3018484	GPR22	0,00370	-2,167	32	15
2514497	PHOSPHO2	0,00807	-2,185	24	11
2816506	S100Z	0,00880	-2,204	29	13
3740618		0,03105	-2,213	13	6
3748449	CCDC144A	0,00776	-2,232	59	26
3269939	DOCK1	0,00291	-2,241	23	10
2742224	SPRY1	0,00052	-2,247	176	78
2565347		0,00319	-2,254	13	6
2854327	FYB	0,00847	-2,303	22	9
2908762	RUNX2	0,00194	-2,327	56	24
2701018	GPR171	0,00166	-2,331	29	12
2406311		0,02278	-2,337	11	5
2674496		0,04281	-2,349	26	11
2635349	TRAT1	0,00052	-2,351	19	8
2486178	MEIS1	0,00040	-2,360	49	21
3474553		0,02565	-2,384	122	51
3401704	CCND2	0,00402	-2,407	54	22
2727587	KIT	0,00000	-2,417	31	13
3551843		0,01720	-2,448	36	15
2901751		0,03508	-2,451	9	4
3507199	FLT3	0,01283	-2,460	173	70
2421000	COL24A1	0,00143	-2,471	31	12
3982612	GPR174	0,03040	-2,491	82	33
2791197	PDGFC	0,00039	-2,541	27	11
3090209	ADAM28	0,01498	-2,596	20	8
2774971	ANTXR2	0,00007	-2,598	139	53
3475112		0,01197	-2,628	42	16
2444283	TNFSF4	0,00010	-2,649	118	45
3177880	DAPK1	0,00052	-2,722	38	14
3092561		0,00389	-2,735	6	2
3475062		0,03036	-2,736	9	3
3973839	CYBB	0,00395	-2,796	40	14
3974019	TSPAN7	0,00003	-2,814	61	22

2557667		0,00464	-2,888	7	3
3174816	ANXA1	0,00253	-2,889	16	6
3475114		0,02454	-2,970	31	10
3740642		0,01730	-2,972	14	5
3749652		0,01094	-2,995	17	6
3217077	HEMGN	0,00022	-3,069	15	5
2925590	TMEM200A	0,00427	-3,112	25	8
2968293		0,00874	-3,162	28	9
2832005		0,04055	-3,192	19	6
2318869		0,00765	-3,222	17	5
3556771		0,01420	-3,257	9	3
3028744	TRY6	0,04738	-3,299	8	2
2565935	ANKRD36B	0,04193	-3,306	9	3
2418078	NEGR1	0,00015	-3,424	68	20
2584957	SCN3A	0,00245	-3,537	101	28
3556066		0,00526	-3,571	39	11
2443450	SELL	0,00009	-4,213	509	121
2832007		0,00454	-4,287	26	6
3110217	BAALC	0,00092	-4,475	76	17
2761842	PROM1	0,00055	-4,956	40	8
3433466	NCRNA00173	0,00029	-5,118	38	7
2968295		0,00001	-6,114	322	53
2772566	IGJ	0,00001	-6,120	354	58

Child PreBII L vs PreBI

Transcript Cluster	Gene Symbol	p-value	fold change	Mean signal PreBI	Mean signal PreBII L
3648391	TNFRSF17	0,00000	9,034	7	65
3578152	**TCL1A**	0,00000	7,187	51	369
2524653	ADAM23	0,00000	7,180	18	129
3755862	IKZF3	0,00000	7,029	27	189
3876084	C20orf103	0,00000	6,568	14	89
2772566	IGJ	0,00001	5,962	58	345
2563785	**IGK@**	0,00000	5,794	81	469
2688717	BTLA	0,00000	5,451	9	49
3098977	LYN	0,00000	5,032	33	165
2388085	KMO	0,00000	4,414	18	80
3973839	CYBB	0,00010	4,372	14	63
3924041	ADARB1	0,00000	4,272	21	90
2823880	CAMK4	0,00001	4,270	14	60
2891341	IRF4	0,00000	3,984	63	250
3374934	MS4A6A	0,00000	3,813	11	41

2349129	S1PR1	0,00000	3,795	12	47
2832007		0,00879	3,778	6	23
2372858	RGS2	0,00276	3,769	131	496
3982560	P2RY10	0,00000	3,765	8	28
2782545	CAMK2D	0,00001	3,762	50	190
2832005		0,02089	3,753	6	23
3384321	RAB30	0,00001	3,726	18	67
3239584	MYO3A	0,00000	3,696	7	24
2860178	CD180	0,00000	3,617	30	108
2363852	FCRLA	0,00000	3,549	15	53
2366884	C1orf129	0,00046	3,492	4	13
3651478	ACSM3	0,00006	3,466	20	70
2733483	BMP3	0,00000	3,415	33	113
3470549	CORO1C	0,00040	3,319	48	159
2689286	KIAA1407	0,00000	3,227	18	59
2589868	CCDC141	0,00000	3,163	17	54
3173673	PIP5K1B	0,00000	3,108	14	42
3512948	C13orf18	0,00542	3,049	92	282
3904691	SAMHD1	0,00063	2,955	25	74
3470388		0,00405	2,923	8	23
2339872	ROR1	0,00000	2,897	18	51
3837731	EMP3	0,00000	2,885	23	65
3516639	PCDH9	0,00000	2,859	23	66
2897172	RNF144B	0,00001	2,830	39	112
3040967	RAPGEF5	0,00000	2,800	28	79
2766492	C4orf34	0,00000	2,791	118	330
2747893	ARFIP1	0,00049	2,781	11	30
2638988	PARP15	0,00038	2,780	29	81
3556755		0,02117	2,777	12	33
3291435	RTKN2	0,00026	2,752	19	52
3204648	CD72	0,00006	2,735	93	253
2901751		0,01993	2,715	4	10
3346548	BIRC3	0,00080	2,696	9	23
3329983	PTPRJ	0,00000	2,679	15	41
2597867	IKZF2	0,00067	2,655	118	314
2968295		0,00664	2,646	53	139
3815399	CNN2	0,00328	2,563	12	32
3590341	CHP	0,00003	2,549	142	362
3013255	PEG10	0,00004	2,538	16	40
3203855	DCAF12	0,00009	2,534	54	136
3475062		0,04446	2,531	3	8
3151970	MTSS1	0,00001	2,530	22	55
3945545	APOBEC3B	0,00297	2,528	16	42
3379452	C11orf24	0,00000	2,445	23	55
3669552	VAT1L	0,00009	2,433	31	75
2900116	HIST1H2BO	0,00485	2,427	169	411
2638676	EAF2	0,00004	2,421	116	280

3994846	MTMR1	0,00000	2,409	71	171
3461496	BEST3	0,04656	2,369	23	55
3290746	SLC16A9	0,00266	2,354	19	46
3020192	TES	0,00052	2,334	54	125
3744965	GAS7	0,00000	2,325	72	168
3725998		0,04026	2,318	12	27
2439101	FCRL1	0,00887	2,314	28	64
3604147	KIAA1199	0,00001	2,304	15	34
2566848	AFF3	0,00000	2,267	50	112
2688605	GCET2	0,00062	2,246	82	185
3902743	C20orf112	0,00878	2,245	26	59
3881651	HCK	0,00000	2,244	21	47
2344450	NEDD8	0,03716	2,228	11	25
2698565	TFDP2	0,00006	2,226	201	448
2565347		0,00391	2,209	6	13
2435383	S100A10	0,02802	2,189	45	99
2320094		0,00222	2,188	8	18
3759335	GJC1	0,00730	2,178	35	75
3301218	PDLIM1	0,00009	2,165	156	338
2900372	ZNF193	0,00000	2,159	11	24
2724671	RHOH	0,00056	2,158	91	195
3203962	KIF24	0,00055	2,152	12	26
3803418	KLHL14	0,00001	2,146	16	35
3356115	APLP2	0,00002	2,146	76	164
2900051	HIST1H3H	0,00982	2,132	11	24
3403092	PTPN6	0,00000	2,130	65	138
3958399		0,00004	2,122	482	1023
2991395	HDAC9	0,00000	2,119	27	58
2881860	CCDC69	0,00011	2,117	88	186
3397589	ETS1	0,00000	2,117	89	189
2939886	LYRM4	0,04609	2,113	5	10
3154263	SLA	0,00039	2,112	13	28
3690154	NETO2	0,00000	2,111	10	22
3377474	SYVN1	0,00002	2,110	143	301
3060117	ABCB4	0,00003	2,094	11	23
2883440	ADAM19	0,00000	2,093	25	52
3082590	LOC286161	0,01041	2,092	65	137
3082531	FBXO25	0,00000	2,066	30	62
2893392	LY86	0,01049	2,065	21	43
2439052	FCRL2	0,00023	2,054	9	19
2461999	LYST	0,00007	2,051	20	41
3340697	UVRAG	0,00015	2,047	59	121
3377895		0,00013	2,023	78	159
2960399	C6orf155	0,00262	2,018	14	28
2440354	CD48	0,00389	2,017	136	275
3207241	FLJ20444	0,04169	2,006	5	9
2775259	RASGEF1B	0,00234	2,006	42	84

3724505	MYL4	0,00002	2,002	19	38
3333443	ASRGL1	0,00528	2,000	28	57
3941010	SRRD	0,00434	-2,001	44	22
3326938		0,01505	-2,004	49	24
2944491	MBOAT1	0,00006	-2,008	44	22
3307939	ABLIM1	0,00001	-2,010	45	22
3182957	NIPSNAP3A	0,00123	-2,012	49	24
4016001	ZMAT1	0,00065	-2,012	99	49
23200482320077	TARDBP	0,04926	-2,014	92	46
2331505	MACF1	0,00183	-2,019	49	24
2777564	FAM13A	0,00133	-2,023	32	16
3767169	LRRC37A3	0,01605	-2,023	24	12
3403841	RIMKLB	0,00002	-2,024	371	183
2968054	SEC63	0,00008	-2,027	464	229
2884647	C5orf54	0,00236	-2,029	13	6
2343289	DNAJB4	0,00111	-2,032	61	30
3453388		0,01258	-2,035	12	6
2840660		0,02313	-2,037	143	70
2969406	SLC22A16	0,00000	-2,042	28	14
2793198		0,03749	-2,049	9	4
3625539	NEDD4	0,00149	-2,057	36	17
2864449	SERINC5	0,00024	-2,058	85	41
3327143	RAG1	0,00027	-2,060	78	38
3740580		0,04156	-2,070	35	17
3147508	KLF10	0,00184	-2,086	77	37
2443450	SELL	0,02699	-2,088	121	58
2853388	C5orf33	0,00041	-2,098	176	84
2603897	TIGD1	0,02969	-2,102	30	14
2955282	SUPT3H	0,00000	-2,103	26	12
2984884	RNASET2	0,00097	-2,105	94	45
2554975	BCL11A	0,00000	-2,110	180	85
3783723	RNF125	0,00144	-2,113	40	19
3894601	FKBP1A	0,00768	-2,114	123	58
2844339		0,02384	-2,116	40	19
2517737	PLEKHA3	0,00659	-2,116	119	56
3676709		0,02298	-2,117	185	87
3249043	REEP3	0,02973	-2,141	64	30
2982319	SOD2	0,01231	-2,141	242	113
2462329	ERO1LB	0,00115	-2,155	102	47
3198346	PTPRD	0,00486	-2,157	57	27
3331487	CTNND1	0,00000	-2,158	101	47
2748830	GUCY1A3	0,00004	-2,183	61	28
3507199	FLT3	0,02819	-2,190	70	32
3421300	MDM2	0,00049	-2,216	393	178
2565353		0,02631	-2,219	88	40
3852880	EMR2	0,00020	-2,222	35	16
3352948	SORL1	0,00001	-2,223	60	27

2775909	PLAC8	0,00012	-2,223	171	77
3630225		0,02354	-2,224	92	41
2340529	PDE4B	0,01015	-2,230	68	30
3898796	KIF16B	0,00004	-2,233	40	18
3624145	DMXL2	0,00018	-2,233	27	12
2840664		0,00432	-2,239	359	160
3126368	PSD3	0,01559	-2,241	27	12
2380785	LYPLAL1	0,00478	-2,242	34	15
3619183		0,04063	-2,249	57	25
2557657		0,03128	-2,256	52	23
3275574		0,04297	-2,269	13	6
3075550	ZC3HAV1L	0,00510	-2,272	22	10
2840662		0,00794	-2,276	200	88
3145149	TP53INP1	0,00209	-2,279	355	156
2345617	PKN2	0,00006	-2,281	245	107
3204721	TPM2	0,00052	-2,285	107	47
2745547	GAB1	0,00725	-2,287	369	162
3183111	SLC44A1	0,00086	-2,300	123	54
2482944		0,02630	-2,301	21	9
2816459	F2R	0,00000	-2,322	28	12
2549260	MAP4K3	0,00005	-2,326	51	22
3740624		0,01814	-2,332	30	13
3610958	IGF1R	0,00016	-2,338	116	50
3619116	GPR176	0,00005	-2,345	31	13
3742415?742428	SPAG7	0,00450	-2,348	135	57
2840700		0,00106	-2,350	76	33
3210808	GNAQ	0,00116	-2,357	265	112
3699884		0,04855	-2,372	7	3
2651165	SERPINI1	0,01636	-2,378	26	11
3955815	HPS4	0,00000	-2,380	174	73
2840772		0,01187	-2,381	281	118
2840702		0,00165	-2,387	214	90
3420442	IRAK3	0,00000	-2,404	18	7
3551853		0,02295	-2,407	177	74
2436184		0,01740	-2,418	7	3
3238491	BMI1	0,00065	-2,427	43	18
3517251	DACH1	0,00001	-2,432	42	17
3426257	SOCS2	0,00000	-2,435	92	38
2867836	GLRX	0,00057	-2,448	126	51
2902444	AIF1	0,00001	-2,448	95	39
3594031	TMOD2	0,00001	-2,454	33	13
3556320		0,00910	-2,457	79	32
3285926	ZNF37B	0,00011	-2,469	31	12
3725868		0,03820	-2,478	8	3
2974330	CTGF	0,00220	-2,494	108	43
2742224	SPRY1	0,00012	-2,517	78	31
2515627	ITGA6	0,00000	-2,519	33	13

2723997	KLF3	0,00020	-2,540	92	36
4008427	NUDT11	0,00257	-2,543	97	38
3509719	SPG20	0,00000	-2,558	31	12
2913694	CD109	0,00000	-2,567	27	10
3548050	PRO1768	0,00012	-2,569	81	31
4001369	SCML2	0,00001	-2,576	50	19
2701033	P2RY14	0,00070	-2,578	74	29
3407096	PLEKHA5	0,00000	-2,641	48	18
2325214		0,00603	-2,651	32	12
3126191	PSD3	0,01944	-2,670	76	29
2949107		0,02218	-2,670	35	13
2375706	ATP2B4	0,00000	-2,678	80	30
2886595	LCP2	0,00000	-2,678	59	22
3031517	GIMAP7	0,00077	-2,695	30	11
3046556	TARP	0,00506	-2,704	65	24
2954022	TRERF1	0,00026	-2,743	38	14
3538893	PRKCH	0,00000	-2,748	125	45
2349402	AMY2B	0,00538	-2,750	55	20
3441849	TNFRSF1A	0,00000	-2,766	68	25
3564620	NID2	0,00385	-2,772	63	23
3286792	C10orf25	0,00350	-2,788	60	22
2421883	GBP1	0,00008	-2,848	28	10
3126504	CSGALNACT1	0,00019	-2,853	38	13
3046708	TRGV3	0,01739	-2,870	38	13
2608469	ITPR1	0,00000	-2,901	114	39
3956589	XBP1	0,00000	-2,901	207	71
2866225	MEF2C	0,00000	-2,911	359	123
2840688		0,01696	-2,913	138	47
2343511	IFI44	0,00819	-2,953	41	14
3031556	GIMAP2	0,00047	-2,956	100	34
2833623	HMHB1	0,00109	-2,958	440	149
3807487 3807501	RPL17	0,03333	-2,959	125	42
3212344		0,01526	-2,980	87	29
2686806		0,04601	-2,997	68	23
2665199	SATB1	0,00001	-3,001	139	46
2363074	SUMO1P3	0,03102	-3,036	21	7
3556158		0,00287	-3,056	88	29
3255506		0,00547	-3,096	48	15
3980922		0,01008	-3,110	11	4
2872047	SEMA6A	0,00000	-3,153	54	17
2858023	PLK2	0,00006	-3,163	50	16
3383227	GAB2	0,00000	-3,230	146	45
2686808		0,00310	-3,267	79	24
3264621	TCF7L2	0,00000	-3,366	88	26
3749696		0,00290	-3,413	74	22
2849992	FAM134B	0,00000	-3,430	54	16
3809671 3809690	NARS	0,03258	-3,443	25	7

3925639	NRIP1	0,00000	-3,485	466	134
2969886	FYN	0,00001	-3,495	91	26
3182984	NIPSNAP3B	0,00000	-3,505	33	9
2674440		0,04782	-3,551	47	13
3811000	RNF152	0,00000	-3,595	55	15
3306565		0,00349	-3,717	16	4
3031533	GIMAP4	0,00060	-3,748	41	11
2633256	ST3GAL6	0,00001	-3,798	37	10
2536965	FLJ38379	0,00018	-3,810	66	17
2557659		0,00175	-3,922	26	7
3937967	FLJ26056	0,00906	-4,061	42	10
2902707	HSPA1A	0,00104	-4,136	284	69
2840666		0,00161	-4,285	70	16
2679014	NPCDR1	0,00004	-4,688	135	29
2444283	TNFSF4	0,00000	-4,982	45	9
3624513	MYO5C	0,00000	-4,986	39	8
3301782	OPALIN	0,00000	-5,102	63	12
3012978	GNG11	0,00107	-5,123	35	7
3427098	ELK3	0,00000	-5,185	162	31
3993360	SPANXB1	0,00610	-5,451	55	10
3096575	HGSNAT	0,00000	-5,493	186	34
2649824	SCHIP1	0,00000	-5,497	145	26
2542972	NDUFAF2	0,00086	-6,064	15	2
3766796	PECAM1	0,00000	-6,102	238	39
2421995	GBP4	0,00000	-6,376	150	24
2453307	CD34	0,00000	-7,047	127	18
3259503	DNTT	0,00004	-8,862	701	79
3931765	ERG	0,00000	-8,946	286	32

Child PreBII s vs PreBII L

Transcript Cluster	Gene Symbol	p-value	fold change	Mean signal	
				PreBII L	PreBII s
3862873	CYP2A6	0,02309	3,769	3	10
3470476		0,02668	3,388	8	26
2320134		0,02361	2,950	3	10
3959724		0,03710	2,821	22	63
3551851		0,04581	2,738	10	28
3725910		0,00802	2,639	25	67
2841416		0,00109	2,599	3	8
3335816		0,00234	2,582	18	46
2406293		0,03199	2,543	6	15
3725868		0,03425	2,531	3	8
2599944		0,03879	2,389	8	19
3595909	RNF111	0,01838	2,339	11	25

3699806		0,02815	2,252	31	70
3749696		0,04013	2,250	22	49
2773434	CXCL2	0,01454	2,219	10	22
2318825		0,04569	2,199	6	14
3819643		0,01777	2,172	80	173
2841478		0,01116	2,157	8	16
2841462		0,04429	2,150	100	216
2679014	NPCDR1	0,03077	2,065	29	60
2325214		0,03570	2,064	12	25
2841482		0,01066	2,051	8	16
2328571		0,01799	2,005	14	28
2390322	OR2M5	0,00797	2,002	8	16
3096512		0,02549	-2,007	54	27
4007899	SYP	0,01608	-2,021	18	9
3470388		0,04534	-2,052	23	11
2766262	TLR6	0,00523	-2,080	12	6
3392928		0,02514	-2,388	33	14
3904691	SAMHD1	0,00447	-2,390	74	31
2318867		0,01378	-3,036	19	6

Child Immature B vs PreBII s

Transcript Cluster	Gene Symbol	p-value	fold change	Mean signal PreBII s	Mean signal Immature B
2531233	SP140	0,00000	22,396	8	177
2688717	BTLA	0,00000	13,031	27	348
3973839	CYBB	0,00000	9,782	39	381
3332403	MS4A1	0,00000	9,666	68	656
3742627	C17orf87	0,00000	8,470	10	82
2369339	RALGPS2	0,00000	8,253	38	315
2445982	ANGPTL1	0,00000	8,221	16	131
2766192	TLR10	0,00000	8,033	59	470
2792166	01.mar	0,00000	7,760	13	101
2362201	CD1C	0,00000	7,505	12	94
2950145	HLA-DOB	0,00000	7,473	27	204
3442854	SLC2A3	0,00000	7,284	20	145
3672489	IRF8	0,00000	6,985	23	164
3815399	CNN2	0,00000	6,931	26	181
3451375	PRICKLE1	0,00000	6,848	18	122
3484060	ALOX5AP	0,00000	6,299	59	370
2486811	PLEK	0,00000	6,165	6	37
2737596	BANK1	0,00000	6,107	33	204
3926271	TMPRSS15	0,00000	6,046	4	23
2968401		0,00131	5,946	4	22
2353988	FAM46C	0,00000	5,894	18	103

3268274	PLEKHA1	0,00000	5,852	17	98
2766289	TMEM156	0,00000	5,792	19	109
2377283	CR2	0,00000	5,672	5	30
2439052	FCRL2	0,00000	5,647	23	127
3384321	RAB30	0,00000	5,376	58	312
4013460	CYSLTR1	0,00000	5,358	26	138
3329983	PTPRJ	0,00000	5,328	40	215
3346548	BIRC3	0,00000	5,318	34	182
2439101	FCRL1	0,00001	5,259	72	379
2968436		0,00166	5,168	9	46
2790062	TMEM154	0,00000	5,164	8	41
2438892	FCRL5	0,00000	5,086	7	38
2968285		0,00062	5,062	23	117
3740634		0,01484	5,005	14	70
2600689	EPHA4	0,00000	5,005	14	68
2868265	LIX1	0,00000	4,942	7	37
2764192	SEL1L3	0,00000	4,751	10	48
2936657	CCR6	0,00000	4,684	6	30
3982560	P2RY10	0,00000	4,645	20	94
2351854	C1orf162	0,00000	4,644	47	220
2734421	ARHGAP24	0,00000	4,599	13	59
2766262	TLR6	0,00000	4,574	6	27
2476671	RASGRP3	0,00000	4,456	7	31
3013255	PEG10	0,00000	4,387	34	148
2599371	TMBIM1	0,00000	4,164	27	113
3275374		0,00621	4,036	3	13
3374934	MS4A6A	0,00000	3,885	43	167
3403595	CLEC4A	0,00000	3,795	20	76
2968313		0,01573	3,761	6	21
2667809	OSBPL10	0,00000	3,757	24	88
2378662	TRAF5	0,00000	3,705	18	67
2664209	SH3BP5	0,00000	3,680	29	109
3958285		0,03901	3,680	7	27
3389450	CASP1	0,00146	3,609	41	148
3837731	EMP3	0,00000	3,580	52	187
3154263	SLA	0,00000	3,556	20	73
2508520	KYNU	0,00005	3,483	4	15
2973376	PTPRK	0,00000	3,478	10	35
2638988	PARP15	0,00003	3,474	61	212
3244622	ALOX5	0,00000	3,469	31	109
3151970	MTSS1	0,00000	3,461	63	218
2860178	CD180	0,00001	3,449	87	300
3824874	IFI30	0,00000	3,447	13	45
3333595	GNG3	0,00000	3,432	20	68
3291601	EGR2	0,00003	3,413	21	70
2984884	RNASET2	0,00000	3,411	59	202
2723997	KLF3	0,00001	3,379	35	118

2363808	FCGR2B	0,00000	3,378	23	78
3375307	CYBASC3	0,00000	3,361	29	97
3350775	SIDT2	0,00000	3,353	48	162
3131881	PPAPDC1B	0,00000	3,306	42	140
3944690	CYTH4	0,00000	3,248	24	76
3471327	HVCN1	0,00000	3,198	27	87
3096575	HGSNAT	0,00054	3,164	37	118
3982612	GPR174	0,00791	3,140	36	112
3362795	RNF141	0,00000	3,134	63	198
2968383		0,00693	3,132	27	86
3654175	IL4R	0,00000	3,125	26	80
3090209	ADAM28	0,00444	3,120	8	25
2366884	C1orf129	0,00123	3,111	7	22
2922631	DSE	0,00001	3,045	16	49
2968279		0,03046	3,042	9	27
3449760	DENND5B	0,00000	3,033	9	27
3286895	OR13A1	0,00030	3,024	9	26
3718930	CCL4	0,00153	3,010	6	17
2903219		0,00128	3,004	43	128
4011844	IL2RG	0,00000	3,002	46	137
3527662	RNASE6	0,00061	2,975	23	69
3319997	SWAP70	0,00000	2,942	115	338
2968375		0,00150	2,907	21	62
2452977	FAIM3	0,00001	2,900	168	489
3403092	PTPN6	0,00000	2,898	104	300
2766492	C4orf34	0,00000	2,872	199	573
2841699	CPEB4	0,00000	2,870	38	109
3969081	TLR7	0,00202	2,834	21	60
2895945		0,00018	2,833	12	34
2968387		0,00719	2,824	22	61
3839360	MYBPC2	0,00000	2,818	15	43
3511698	EPSTI1	0,02376	2,804	25	69
3229797	QSOX2	0,00000	2,804	19	54
2968432		0,00936	2,791	24	66
3951136	RPL23AP82	0,00052	2,788	9	26
3442785	CLEC4C	0,00000	2,766	12	32
3275202		0,02186	2,762	9	26
3958375		0,02396	2,746	6	16
3061997	PON2	0,00000	2,720	32	86
4044363	CNR2	0,00009	2,682	27	72
3275139		0,00305	2,673	14	36
2774971	ANTXR2	0,00005	2,668	59	158
2368180	GPR52	0,00001	2,666	20	53
2326463	CD52	0,00000	2,664	488	1301
3838385	CD37	0,00109	2,654	111	295
3464983	ATP2B1	0,00001	2,648	75	197
3375648	FTH1	0,00179	2,642	33	87

3940631	ADRBK2	0,00000	2,627	67	177
3156307	PTK2	0,00003	2,612	8	22
2775909	PLAC8	0,00001	2,605	86	223
2536332		0,00957	2,602	9	23
2968281		0,00147	2,602	45	116
2582124	NR4A2	0,00299	2,596	12	31
3061805	SGCE	0,00000	2,585	14	37
2866225	MEF2C	0,00000	2,583	133	344
3275264		0,03631	2,571	8	21
3307939	ABLIM1	0,00000	2,565	23	60
3662387	HERPUD1	0,00000	2,558	104	265
3626826	MYO1E	0,00001	2,544	9	22
2608725	BHLHE40	0,00000	2,531	18	46
3904691	SAMHD1	0,00271	2,526	31	78
2431112	NOTCH2	0,00000	2,515	31	77
2968295		0,00970	2,512	138	346
2968283		0,01966	2,508	15	37
2692060	PARP9	0,00324	2,507	102	255
3306565		0,03482	2,494	5	13
2810764	GAPT	0,00001	2,494	67	166
3766796	PECAM1	0,00118	2,477	55	136
2377035	IL24	0,00003	2,473	25	63
2434575	CTSS	0,00001	2,466	59	146
2968416		0,01575	2,463	25	62
2840656		0,03979	2,463	18	45
3275332		0,04304	2,451	6	14
2968377		0,00048	2,450	176	431
2563785	IGK@	0,00065	2,435	438	1067
2830861	EGR1	0,00004	2,431	41	99
3726036		0,04933	2,426	3	8
3173673	PIP5K1B	0,00001	2,420	41	100
2841410		0,03828	2,411	5	13
3374402	LPXN	0,00000	2,403	25	60
2518583	DNAJC10	0,00001	2,402	80	192
2968293		0,04141	2,396	12	28
2993727	SNX10	0,00023	2,392	24	58
3222144	TNFSF8	0,00041	2,392	23	56
3379452	C11orf24	0,00000	2,383	48	115
2613293	KCNH8	0,00000	2,378	9	22
3843662	ZNF587	0,03432	2,371	38	90
3061456	SAMD9L	0,00023	2,371	31	73
3608220	CRTC3	0,00000	2,363	25	59
2608469	ITPR1	0,00006	2,357	53	126
2601287	AP1S3	0,00003	2,342	8	20
2557657		0,02500	2,339	23	54
2982076	TAGAP	0,00000	2,337	38	89
3834502	CD79A	0,00006	2,334	105	246

2459411		0,01033	2,331	9	21
2536502		0,00132	2,330	36	84
2835792	GM2A	0,00118	2,319	62	143
3145240	C8orf37	0,00029	2,318	7	15
2443450	SELL	0,01257	2,318	58	134
2903435	HLA-DPB2	0,00001	2,318	15	34
2957596	ELOVL5	0,00002	2,317	152	353
2991395	HDAC9	0,00000	2,305	53	123
2585129	GALNT3	0,00001	2,300	8	18
2604390	ARL4C	0,00006	2,299	30	69
2912649	COL19A1	0,00000	2,294	9	20
3755862	IKZF3	0,00012	2,282	148	338
3281068	PIP4K2A	0,00006	2,282	37	84
3303392	BLOC1S2	0,00000	2,280	44	101
2832029		0,03161	2,277	7	15
2522693	CASP10	0,00001	2,276	14	33
2377229	CD55	0,00001	2,274	214	488
3145957		0,02863	2,268	5	12
2363852	FCRLA	0,00023	2,268	48	110
2959039	KHDRBS2	0,00001	2,265	18	40
2991150	TSPAN13	0,00001	2,263	109	246
2470165	TRIB2	0,00013	2,257	55	124
2531310	SP140L	0,00004	2,254	71	160
2423175	FAM69A	0,00004	2,235	34	77
3449910	AMN1	0,00014	2,235	58	129
2536965	FLJ38379	0,01557	2,231	25	56
2737318	DAPP1	0,00006	2,228	33	74
3922100	MX1	0,01127	2,224	30	68
2949118	LTB	0,00000	2,224	57	127
3202316	MOBKL2B	0,00000	2,222	21	47
3958397		0,00883	2,218	7	15
3850261	ICAM3	0,00001	2,218	23	50
2974413	MOXD1	0,00001	2,218	7	15
3401828	DYRK4	0,00000	2,212	23	51
2478748	EML4	0,00000	2,210	57	125
3060117	ABCB4	0,00001	2,207	34	75
2363689	FCGR2A	0,00030	2,202	8	17
2939014	MGC39372	0,00016	2,199	41	90
2444842	KIAA0040	0,00019	2,188	97	211
3470472		0,01061	2,187	17	37
3946095	GRAP2	0,00012	2,182	12	27
2531377	SP100	0,00001	2,175	78	169
3204680	SIT1	0,00027	2,175	17	37
2968257		0,03027	2,170	24	52
2318795		0,00251	2,168	13	29
2427720	DRAM2	0,00075	2,163	130	281
3451988	PLEKHA9	0,00000	2,154	47	101

2369325	C1orf220	0,00001	2,152	12	25
2593464	ANKRD44	0,00000	2,142	68	145
3918635	IFNGR2	0,00000	2,137	69	147
3740588		0,02942	2,135	44	95
2726072	ATP10D	0,00003	2,133	52	110
3551816		0,00028	2,131	44	94
2553282	PSME4	0,00001	2,126	70	149
2373842	PTPRC	0,00004	2,126	267	568
3475112		0,04563	2,123	16	35
2437554		0,04164	2,113	18	37
2440354	CD48	0,00231	2,111	207	436
3444493	TAS2R19	0,02009	2,108	23	48
3275222		0,04443	2,107	7	14
2388085	KMO	0,00184	2,102	89	187
2423017	EVI5	0,01069	2,100	25	52
2968395		0,00635	2,096	9	19
3556816	SLC7A7	0,00000	2,096	15	32
3978943	KLF8	0,00003	2,096	28	59
3007438	POM121	0,02746	2,095	12	24
3748400	USP6	0,02299	2,092	154	323
2974671	C6orf192	0,00065	2,089	38	79
2881747	ANXA6	0,00001	2,089	44	92
2950329	HLA-DPA1	0,00014	2,088	250	522
2968232[illegible]968246	SNX3	0,00024	2,084	140	292
2950263	HLA-DMB	0,00119	2,076	136	282
2949971	C6orf10	0,00059	2,075	9	18
2883440	ADAM19	0,00000	2,067	61	126
3023318	TSPAN33	0,00003	2,067	18	38
2715634	ADD1	0,00000	2,064	75	154
2656837	ST6GAL1	0,00001	2,064	50	103
3725856		0,01386	2,061	20	40
3551841		0,00014	2,059	45	93
3275188		0,03892	2,055	10	20
3361040		0,03072	2,051	14	28
2523620		0,00000	2,050	30	61
2926969	PDE7B	0,00000	2,048	11	22
2349129	S1PR1	0,00126	2,046	48	97
3035990	CARD11	0,00000	2,045	24	49
3432438	OAS1	0,00049	2,042	43	89
3548050	PRO1768	0,00222	2,042	33	68
2371065	LAMC1	0,00000	2,040	17	35
3258221	HHEX	0,00000	2,039	63	129
2475678	LBH	0,00000	2,037	62	127
2636483	SIDT1	0,00003	2,036	41	83
2362180	CD1A	0,00004	2,035	12	24
2954527	ZNF318	0,00000	2,026	33	67
3107828	PLEKHF2	0,00719	2,020	95	192

3634071	TSPAN3	0,00000	2,019	81	163
3299585	LIPA	0,00001	2,017	16	33
3275194		0,04998	2,015	68	138
2584787	COBLL1	0,00097	2,009	13	27
3414739	METTL7A	0,00000	2,004	22	44
2374700	RPS10P7	0,01557	2,002	19	38
3302177	ARHGAP19	0,00523	-2,002	160	80
3875642	PLCB1	0,00014	-2,007	24	12
2952679	GLO1	0,00666	-2,015	193	96
2867836	GLRX	0,00505	-2,017	65	32
3268669	BUB3	0,00002	-2,018	337	167
3921391	WRB	0,00037	-2,019	42	21
2645764	ATP1B3	0,00472	-2,030	112	55
2402416	C1orf135	0,00071	-2,030	41	20
2798538	SDHA	0,00027	-2,032	190	94
2597867	IKZF2	0,00972	-2,033	320	158
3781082	SNRPD1	0,00032	-2,034	156	77
2964231	RRAGD	0,00058	-2,035	33	16
3959918	TST	0,00004	-2,037	61	30
2328571		0,01567	-2,038	28	14
2715076	WHSC1	0,00000	-2,039	89	44
2980241	FBXO5	0,00591	-2,042	113	55
3216319	ZNF367	0,00000	-2,047	58	28
2366581	SCYL3	0,00099	-2,048	36	17
3236448	SUV39H2	0,00095	-2,048	51	25
2461891	B3GALNT2	0,00003	-2,048	120	59
3593575	SLC27A2	0,00075	-2,048	15	7
24361322436139	ILF2	0,00013	-2,049	429	209
2735221	PKD2	0,00020	-2,055	104	51
2520225	NAB1	0,00030	-2,056	37	18
2565219		0,04423	-2,058	86	42
3456630	CBX5	0,00000	-2,060	874	424
3819773		0,00652	-2,060	22	10
3921992	FAM3B	0,00043	-2,061	17	8
2864849	SSBP2	0,00033	-2,062	327	159
2746119	SMAD1	0,01163	-2,064	129	63
24062452406253	PSMB2	0,00571	-2,068	146	71
2568968	UXS1	0,00041	-2,070	106	51
3311157	OAT	0,00231	-2,071	28	14
2481142	MSH6	0,00008	-2,074	266	128
3699044	RFWD3	0,00002	-2,075	69	33
2982381	TCP1	0,00027	-2,078	530	255
3065546	DPY19L2P2	0,03276	-2,079	158	76
2335922	CDKN2C	0,00004	-2,080	36	17
4011743	SLC7A3	0,00033	-2,080	62	30
2442858	BRP44	0,00324	-2,081	88	42
3563395	POLE2	0,00179	-2,081	15	7

3334125	COX8A	0,00015	-2,083	148	71
3570057		0,00017	-2,088	466	223
3929775	DONSON	0,00005	-2,090	38	18
3490655	CKAP2	0,00023	-2,095	151	72
2328567		0,00968	-2,096	18	8
3427014	SNRPF	0,00001	-2,096	378	180
2408499	SCMH1	0,00000	-2,097	121	58
3063685	MCM7	0,00001	-2,098	148	70
2946345	HIST1H2BG	0,00320	-2,098	135	64
3932261	C21orf87	0,00159	-2,102	19	9
2763278	GPR125	0,00091	-2,102	70	33
3277751	NUDT5	0,00068	-2,103	38	18
3233049	AKR1C3	0,00528	-2,106	12	6
3431426	IFT81	0,00009	-2,106	29	14
3091077	DPYSL2	0,00002	-2,111	104	49
3421523	YEATS4	0,00036	-2,112	144	68
2665572	SGOL1	0,00175	-2,113	98	47
2521479	HSPE1	0,01805	-2,115	34	16
3747717	COPS3	0,00001	-2,115	180	85
2728189	PAICS	0,00090	-2,116	48	22
3577256	C14orf142	0,04148	-2,120	41	19
2436145		0,00009	-2,121	309	146
2436141		0,00353	-2,122	213	100
3410908	ALG10	0,00384	-2,124	27	13
3573994	C14orf145	0,00022	-2,124	220	104
2842101	SFXN1	0,00003	-2,126	83	39
2900051	HIST1H3H	0,00992	-2,129	18	8
2383829		0,02190	-2,132	33	16
2328465 2328494	KHDRBS1	0,00575	-2,132	141	66
3422144	LGR5	0,00008	-2,136	16	8
2406245 2406255	PSMB2	0,00000	-2,136	402	188
2395490	ENO1	0,00011	-2,141	214	100
2339872	ROR1	0,00018	-2,141	67	31
3570055		0,01113	-2,144	194	91
2735409	HERC5	0,00215	-2,145	59	27
3690154	NETO2	0,00000	-2,146	28	13
2841388		0,01749	-2,149	19	9
2353477	ATP1A1	0,00009	-2,149	137	64
3316208	TALDO1	0,00001	-2,150	136	63
3804358	C18orf10	0,00004	-2,158	114	53
2899206	HIST1H2BF	0,00001	-2,160	1604	743
2353773	TTF2	0,00014	-2,160	58	27
2489172	MTHFD2	0,00132	-2,161	77	36
2406245 2406256	PSMB2	0,00001	-2,162	342	158
3303255	ERLIN1	0,00700	-2,163	85	39
2485257	UGP2	0,00070	-2,164	209	97
2678714	FHIT	0,00068	-2,168	132	61

3556966	HAUS4	0,00000	-2,170	71	33
3453732	TUBA1B	0,00758	-2,170	702	324
2774049	SCARB2	0,00018	-2,173	105	48
3440598	FOXM1	0,00000	-2,173	63	29
2412799	ORC1L	0,00002	-2,175	52	24
2465395	ZNF695	0,00163	-2,176	44	20
3850501	LOC147727	0,00239	-2,176	216	99
3738280	ANAPC11	0,00481	-2,182	94	43
3719702	MRPL45	0,00879	-2,184	70	32
3290210	ZWINT	0,00000	-2,188	33	15
3636522	HDGFRP3	0,00011	-2,189	82	37
4013828	HMGN5	0,00062	-2,192	36	17
3270270	PTPRE	0,00054	-2,195	105	48
2339414	USP1	0,00522	-2,195	105	48
2366422	ATP1B1	0,00009	-2,197	32	14
2832045		0,01194	-2,199	83	38
2910364	TMEM14A	0,00075	-2,200	52	24
2620150	ZNF660	0,00251	-2,202	57	26
3669552	VAT1L	0,00037	-2,204	47	21
3373845	SLC43A3	0,00000	-2,204	55	25
2947063	HIST1H2AK	0,01159	-2,205	230	104
2328517		0,03589	-2,207	72	33
2831975		0,01748	-2,207	16	7
3535674	C14orf166	0,00001	-2,210	575	260
3628469	RPS27L	0,01026	-2,211	70	32
2328511		0,01211	-2,212	161	73
3819543 3819584	HNRNPM	0,04114	-2,212	122	55
3399545	NCAPD3	0,00000	-2,215	72	32
2635906	PHLDB2	0,00025	-2,217	52	23
2416218	ITGB3BP	0,00974	-2,218	107	48
2644702	FAIM	0,00052	-2,219	46	21
3781429	RBBP8	0,00014	-2,221	95	43
3807569	ACAA2	0,00007	-2,221	137	62
3417485	OBFC2B	0,00013	-2,226	204	92
2435383	S100A10	0,02492	-2,228	80	36
3980898		0,00568	-2,231	101	45
2402459	STMN1	0,00000	-2,233	791	354
3803120	B4GALT6	0,00151	-2,242	34	15
2993672		0,02319	-2,245	26	11
3980896		0,00008	-2,247	173	77
2465493	ZNF670	0,00040	-2,259	36	16
2782230	TIFA	0,00277	-2,261	168	74
3821908	RNASEH2A	0,00000	-2,266	84	37
3446868	LDHB	0,00000	-2,267	713	314
3368707	CD59	0,00002	-2,269	65	29
2784687	ANKRD50	0,00151	-2,282	159	70
3458133	PRIM1	0,00023	-2,289	66	29

3183111	SLC44A1	0,00090	-2,291	62	27
3428268	GAS2L3	0,00023	-2,295	46	20
2549565	SLC8A1	0,00023	-2,295	21	9
3485074	RFC3	0,00014	-2,298	178	78
2757319	SLBP	0,00021	-2,299	77	34
3331903	FAM111B	0,00187	-2,300	185	81
3105430	LRRCC1	0,00045	-2,302	32	14
3286792	C10orf25	0,01492	-2,304	32	14
3502710	TFDP1	0,00000	-2,305	178	77
2500919	SLC20A1	0,00002	-2,308	89	38
3341440	RN28S1	0,00001	-2,309	64	28
3821301	ZNF627	0,00009	-2,310	33	14
2434319	ANP32E	0,00019	-2,321	542	233
3439178	PXMP2	0,00000	-2,322	74	32
3759335	GJC1	0,00395	-2,328	43	19
2901687?901715	ABCF1	0,02313	-2,329	56	24
4013434	TAF9B	0,00008	-2,334	73	31
2732273	11.sep	0,00039	-2,338	141	60
2636062	C3orf52	0,00232	-2,338	73	31
3876645	BTBD3	0,00045	-2,342	116	49
3902743	C20orf112	0,00604	-2,345	73	31
3758317	BRCA1	0,00006	-2,346	40	17
2840002	CCDC99	0,00133	-2,349	118	50
3955815	HPS4	0,00000	-2,350	83	35
2744734	MGST2	0,00000	-2,352	116	49
3902489	BCL2L1	0,00002	-2,352	129	55
3507199	FLT3	0,01728	-2,356	25	11
3468301	PMCH	0,00387	-2,358	10	4
3513549	RCBTB2	0,00000	-2,359	48	20
3975893	PHF16	0,00000	-2,360	43	18
2396781	MAD2L2	0,00016	-2,369	122	51
2779486	H2AFZ	0,00005	-2,372	2268	956
3427767	TMPO	0,00004	-2,374	190	80
3593147	DUT	0,00001	-2,375	96	41
3985534	NGFRAP1	0,00000	-2,376	39	17
3197955	GLDC	0,00129	-2,384	64	27
3082181	NCAPG2	0,00026	-2,387	48	20
3534785	PPIL5	0,00022	-2,387	68	29
3651018	CP110	0,00006	-2,393	77	32
3980892		0,00000	-2,394	974	407
3751830	BLMH	0,00000	-2,394	61	25
2947073	HIST1H1B	0,00125	-2,406	40	16
3757970	PSMC3IP	0,00007	-2,408	83	34
3371719	CKAP5	0,00000	-2,413	170	71
2319802	PGD	0,00001	-2,416	87	36
3817501	CHAF1A	0,00000	-2,421	93	38
3319937	WEE1	0,00000	-2,422	82	34

3136888	TOX	0,00024	-2,425	43	18
3980889		0,00096	-2,428	501	206
2413519	HSPB11	0,00051	-2,431	192	79
3658925	ORC6L	0,00003	-2,433	86	35
3254488	C10orf58	0,00012	-2,439	60	24
3744263	AURKB	0,00000	-2,440	108	44
3828112	CCNE1	0,00000	-2,443	42	17
3090697	CDCA2	0,00013	-2,445	39	16
3945545	APOBEC3B	0,00388	-2,454	30	12
2899171	HIST1H1E	0,00038	-2,455	160	65
2965739	C6orf167	0,00018	-2,459	40	16
3683018	RPS15A	0,00018	-2,461	391	159
2345929	LRRC8C	0,00000	-2,468	184	75
2417528	DEPDC1	0,00636	-2,469	21	9
2704143	WDR49	0,00000	-2,472	69	28
2522212	SGOL2	0,00003	-2,477	41	17
2332144	CTPS	0,00001	-2,481	158	64
2830638	KIF20A	0,00039	-2,487	39	16
2330773	CDCA8	0,00062	-2,492	60	24
2389130	EFCAB2	0,00613	-2,492	63	25
3505937	CENPJ	0,00002	-2,495	73	29
2709486	RFC4	0,00055	-2,496	141	56
3570056		0,00155	-2,498	169	67
3716893	ATAD5	0,00006	-2,503	59	24
3402571	NCAPD2	0,00000	-2,505	97	39
2901913	TUBB	0,00003	-2,513	1208	481
3429857	C12orf75	0,00009	-2,514	164	65
3176999	RMI1	0,00001	-2,522	75	30
3240012	MASTL	0,00032	-2,523	70	28
3291435	RTKN2	0,00068	-2,527	33	13
3470310		0,02625	-2,527	22	9
3604147	KIAA1199	0,00000	-2,534	47	19
3984655	CENPI	0,00007	-2,546	28	11
3198346	PTPRD	0,00087	-2,549	33	13
2902178	TCF19	0,00000	-2,560	84	33
2521278	CCDC150	0,00000	-2,561	28	11
2957126	MCM3	0,00000	-2,563	151	59
2971801	MAN1A1	0,00001	-2,565	149	58
2946369	HIST1H3G	0,00091	-2,573	136	53
3430926	UNG	0,00003	-2,581	71	27
2951567	FKBP5	0,00022	-2,593	92	35
2899756	HIST1H2AG	0,00000	-2,598	47	18
3750785	SPAG5	0,00000	-2,601	45	17
2840682		0,00609	-2,606	56	22
2902707	HSPA1A	0,01983	-2,612	54	21
2514563	KLHL23	0,00888	-2,632	36	14
3876084	C20orf103	0,00115	-2,635	128	49

2946219	HIST1H2AB	0,02030	-2,641	64	24
3484641	BRCA2	0,00002	-2,641	25	9
3367338	KIF18A	0,00011	-2,641	32	12
2714955	TACC3	0,00001	-2,654	90	34
2709606	RPL39L	0,00031	-2,654	302	114
2946232	HIST1H1C	0,00039	-2,669	665	249
2823880	CAMK4	0,00129	-2,672	48	18
3817698	UHRF1	0,00001	-2,682	34	13
3706753	GSG2	0,00000	-2,686	46	17
3003143	MRPS17	0,00812	-2,707	90	33
3630099	TIPIN	0,00021	-2,711	193	71
2946353	HIST1H1D	0,00009	-2,713	121	44
2891341	IRF4	0,00019	-2,716	304	112
2900091	HIST1H2AL	0,00054	-2,725	271	99
2697490	CEP70	0,00004	-2,736	62	22
2993690		0,00044	-2,742	74	27
3163728	CNTLN	0,00002	-2,746	18	6
2900116	HIST1H2BO	0,00160	-2,751	268	97
3080283	XRCC2	0,00008	-2,761	50	18
3010439	GNAI1	0,00002	-2,764	81	29
2899090	HIST1H3A	0,00207	-2,769	40	14
2947100	HIST1H2AM	0,00270	-2,773	43	16
2516023	CDCA7	0,00000	-2,789	52	19
3715489	TMEM97	0,00019	-2,798	62	22
3773312	EIF4A3	0,00000	-2,808	174	62
2844257		0,02033	-2,813	33	12
2328465	KHDRBS1	0,02297	-2,818	42	15
2687979	KIAA1524	0,00003	-2,828	29	10
3653072	PLK1	0,00003	-2,829	60	21
3764738	SKA2	0,00000	-2,831	207	73
3985523	WBP5	0,00007	-2,832	33	12
2421995	GBP4	0,00047	-2,836	33	12
2411228	STIL	0,00005	-2,838	34	12
2650199	SMC4	0,00003	-2,838	253	89
3447694	BCAT1	0,00001	-2,838	55	19
3736290	BIRC5	0,00000	-2,841	97	34
3827218	RPSAP58	0,00619	-2,847	40	14
3248289	CDK1	0,00080	-2,860	26	9
3737874	BAHCC1	0,00000	-2,862	389	136
2698565	TFDP2	0,00000	-2,882	438	152
2648677	MME	0,00000	-2,883	604	210
3367036	CCDC34	0,00000	-2,894	72	25
3390542	RDX	0,00001	-2,905	79	27
2511820	PKP4	0,00000	-2,907	57	20
2454444	NEK2	0,00001	-2,909	86	30
2926802	MYB	0,00001	-2,912	583	200
3932131	PSMG1	0,00004	-2,921	87	30

2346399	CDC7	0,00009	-2,928	78	27
3463112	E2F7	0,00000	-2,928	63	21
3433747	RFC5	0,00000	-2,946	83	28
3551800		0,00523	-2,947	10	3
2674488		0,00648	-2,966	83	28
3551778		0,00413	-2,968	79	27
3749682		0,00923	-2,969	35	12
3417146	CDK2	0,00000	-2,982	108	36
2899243	HIST1H4F	0,00116	-2,995	209	70
2793951	HMGB2	0,00004	-3,002	1207	402
2898597	GMNN	0,00004	-3,009	80	27
3920003	CHAF1B	0,00000	-3,074	50	16
3573933	C14orf145	0,00001	-3,078	51	17
2899223	HIST1H2AE	0,00008	-3,079	759	246
3327143	RAG1	0,00000	-3,087	64	21
3435362	KNTC1	0,00000	-3,090	65	21
3749648		0,00878	-3,096	55	18
3896200	PCNA	0,00000	-3,101	154	50
2604254	HJURP	0,00000	-3,119	94	30
3921068	ETS2	0,00000	-3,133	115	37
3504617	SKA3	0,00005	-3,153	56	18
3454223	RACGAP1	0,00000	-3,157	221	70
3078348	EZH2	0,00000	-3,172	190	60
3160175	VLDLR	0,01693	-3,175	38	12
3340269	POLD3	0,00000	-3,177	212	67
3972093	POLA1	0,00000	-3,198	146	46
3318009	RRM1	0,00000	-3,199	112	35
3203855	DCAF12	0,00000	-3,201	143	45
2914777	TTK	0,00007	-3,212	30	9
2781138	LEF1	0,00091	-3,233	346	107
3703112	GINS2	0,00000	-3,242	109	34
2827185	LMNB1	0,00000	-3,258	277	85
2652675	ECT2	0,00002	-3,273	42	13
3137875	GGH	0,00006	-3,278	34	10
2450345	KIF14	0,00001	-3,285	47	14
2389789	SCCPDH	0,00000	-3,288	89	27
2780172	CENPE	0,00005	-3,294	46	14
2688605	GCET2	0,00000	-3,301	191	58
3322251	NUCB2	0,00000	-3,319	184	55
2946194	HIST1H1A	0,00084	-3,326	115	34
2742985	PLK4	0,00001	-3,329	53	16
3219621	CTNNAL1	0,00000	-3,365	53	16
2752725	NEIL3	0,00001	-3,366	48	14
3689880	SHCBP1	0,00000	-3,378	84	25
3960685	DMC1	0,00000	-3,387	16	5
3728964	PRR11	0,00000	-3,399	70	21
2494484	NCAPH	0,00001	-3,418	73	21

3648391	TNFRSF17	0,00086	-3,433	69	20
2679014	NPCDR1	0,00057	-3,442	60	17
3294280	DNAJC9	0,00001	-3,445	319	93
3910785	AURKA	0,00006	-3,454	63	18
3819667		0,02785	-3,457	16	5
3182781	SMC2	0,00000	-3,470	200	58
3401804	RAD51AP1	0,00000	-3,479	209	60
2783715	MAD2L1	0,00038	-3,498	113	32
2428501	SLC16A1	0,00001	-3,508	92	26
3210808	GNAQ	0,00001	-3,508	109	31
3150715	DSCC1	0,00001	-3,512	48	14
3991889	FAM127A	0,01567	-3,526	17	5
3097152	MCM4	0,00000	-3,532	98	28
3325503	RCN1	0,00562	-3,535	134	38
2899768	HIST1H4I	0,00000	-3,597	72	20
2946319	HIST1H4D	0,00063	-3,615	63	17
3565571	WDHD1	0,00000	-3,623	121	33
2570616	BUB1	0,00000	-3,635	93	25
3151534	ATAD2	0,00000	-3,640	85	23
3625761	MNS1	0,00002	-3,665	50	14
2784113	CCNA2	0,00000	-3,676	108	29
3931765	ERG	0,00037	-3,684	56	15
2691575	POLQ	0,00000	-3,691	59	16
2388219	EXO1	0,00001	-3,725	78	21
3235789	MCM10	0,00000	-3,753	60	16
3744965	GAS7	0,00000	-3,773	252	67
3590014	CASC5	0,00000	-3,789	150	40
3290746	SLC16A9	0,00002	-3,812	72	19
2946268	HIST1H2BC	0,00058	-3,824	196	51
2434129	HIST2H2AB	0,00000	-3,833	1126	294
3145107	CCNE2	0,00001	-3,843	70	18
3607537	FANCI	0,00000	-3,867	100	26
3651478	ACSM3	0,00002	-3,877	132	34
2571457	CKAP2L	0,00001	-3,888	90	23
4041113	KPNA2	0,00656	-3,895	24	6
3936913	CDC45	0,00000	-3,896	111	29
2796510	MLF1IP	0,00008	-3,909	108	28
3938792	VPREB1	0,00000	-3,910	334	85
2997376	ANLN	0,00001	-3,913	80	20
2838201	PTTG1	0,00000	-3,917	887	227
3639031	PRC1	0,00000	-3,919	228	58
3683806	ERI2	0,00000	-3,956	41	10
3258444	CEP55	0,00000	-3,972	30	8
3980560	KIF4A	0,00000	-3,978	166	42
4052881	FAM72D	0,00001	-4,009	59	15
4027585	MPP1	0,00000	-4,019	209	52
3595979	CCNB2	0,00022	-4,020	81	20

3599811	KIF23	0,00000	-4,038	81	20
3765580	BRIP1	0,00000	-4,064	75	18
3590388	NUSAP1	0,00000	-4,096	425	104
2859667	CENPK	0,00004	-4,096	110	27
2813414	CCNB1	0,00000	-4,198	192	46
2875193	P4HA2	0,00049	-4,241	172	41
3515965	DIAPH3	0,00000	-4,247	41	10
3365776	E2F8	0,00000	-4,264	103	24
3129149	PBK	0,00014	-4,309	43	10
3587457	ARHGAP11A	0,00001	-4,317	74	17
3258910	HELLS	0,00000	-4,409	126	29
3880827	GINS1	0,00001	-4,448	133	30
2946208	HIST1H4B	0,00000	-4,452	1335	300
2833623	HMHB1	0,00002	-4,499	282	63
3168508	MELK	0,00000	-4,499	54	12
2931569	AKAP12	0,00013	-4,528	249	55
2449559	ASPM	0,00000	-4,611	105	23
2364438	NUF2	0,00001	-4,619	116	25
3178583	CKS2	0,00000	-4,619	253	55
2858592	DEPDC1B	0,00000	-4,633	112	24
2451200	UBE2T	0,00000	-4,657	115	25
3756193	TOP2A	0,00000	-4,664	351	75
2975655	FAM54A	0,00006	-4,698	75	16
4016193	TMSB15A	0,01184	-4,710	44	9
2406420	CLSPN	0,00001	-4,713	124	26
3461496	BEST3	0,00080	-4,727	90	19
3607510	FANCI	0,00000	-4,777	164	34
3591704	WDR76	0,00000	-4,795	158	33
3720896	CDC6	0,00000	-4,816	106	22
3590086	RAD51	0,00000	-4,849	97	20
2720251	NCAPG	0,00000	-4,870	200	41
3536336	CDKN3	0,00000	-4,882	210	43
2947081	HIST1H4L	0,00001	-4,912	540	110
2838656	HMMR	0,00002	-4,921	106	22
3776139	NDC80	0,00002	-4,935	76	15
2616596	ARPP21	0,00006	-4,998	204	41
2946364	HIST1H3F	0,00001	-5,013	436	87
3258168	KIF11	0,00001	-5,060	98	19
3589697	BUB1B	0,00000	-5,068	183	36
2577896	MCM6	0,00000	-5,082	181	36
2806468	IL7R	0,00068	-5,141	95	19
2378937	DTL	0,00000	-5,159	255	49
2620256	KIF15	0,00000	-5,162	108	21
2469252	RRM2	0,00000	-5,170	1400	271
2899102	HIST1H3C	0,00015	-5,212	198	38
2947077	HIST1H3I	0,00001	-5,239	698	133
3753568	SLFN13	0,00000	-5,326	144	27

3881443	TPX2	0,00000	-5,358	161	30
2379863	CENPF	0,00000	-5,375	313	58
2748163	MND1	0,00001	-5,402	175	32
3775842	TYMS	0,00000	-5,488	654	119
3354799	CHEK1	0,00000	-5,525	132	24
2900059	HIST1H2BM	0,00000	-5,582	732	131
3565663	DLGAP5	0,00000	-5,774	143	25
2946215	HIST1H3B	0,00012	-5,789	405	70
3772187	EPR1	0,00000	-5,871	788	134
2621570		0,00981	-5,912	26	4
2946225	HIST1H2BB	0,00292	-6,043	16	3
3676669 3676671	RNPS1	0,00156	-6,113	32	5
3629103	KIAA0101	0,00000	-6,231	144	23
3312490	MKI67	0,00000	-6,305	383	61
2585933	SPC25	0,00001	-6,552	140	21
3369931	RAG2	0,00000	-7,276	152	21
3259503	DNTT	0,00005	-8,627	179	21
3556735		0,01647	-9,382	75	8
