# Supplementary material for: Transcriptional Profiling of mRNAs and microRNAs in Human Bone Marrow Precursor B Cells Identifies Subset- and Age-Specific Variations
Source: PLoS One. 2013 Jul 30;8(7):e70721. doi: 10.1371/journal.pone.0070721 (PMC3728296; doi:10.1371/journal.pone.0070721)
Supplement: Table S4 — (PDF) [file pone.0070721.s008.pdf]

Mean expression values of B lineage related transcripts during differentiation

	Expression values					
Gene Symbol	ProB	PreBI	PreBII large	Pre-BII small	Immature B	
	9	27	189	148	338	Children
Aiolos (IKZF3)	8	13	149	112	261	Adults
	241	180	85	109	163	Children
BCL11A	231	179	102	131	135	Adults
	488	341	277	292	358	Children
BLNK	459	401	306	311	325	Adults
	78	88	140	170	162	Children
BTK	69	67	121	130	154	Adults
	621	684	471	604	210	Children
CD10	474	520	392	501	259	Adults
	29	36	57	64	71	Children
CD19	28	41	51	52	70	Adults
	50	59	98	68	656	Children
CD20	46	78	125	77	478	Adults
	125	137	175	181	323	Children
CD22	151	143	213	217	352	Adults
	192	127	18	22	21	Children
CD34	143	85	18	25	19	Adults
	73	70	128	105	246	Children
CD79A	91	124	151	155	232	Adults
	57	74	110	136	138	Children
CD79B	52	118	83	164	153	Adults
	551	535	391	453	342	Children
EBF1	595	608	421	522	369	Adults
	37	118	314	320	158	Children
Helios (IKZF2)	19	53	261	198	167	Adults
	964	1256	1422	1207	402	Children
HMGB2	965	1002	1371	1206	736	Adults
	21	34	24	21	11	Children
ID2	51	43	137	46	19	Adults
	38	81	469	438	1067	Children
IGK@	135	337	389	494	1071	Adults
	9	27	189	148	338	Children
Ikaros (IKZF1)	8	13	149	112	261	Adults
	36	63	250	304	112	Children
IRF4	31	55	204	261	134	Adults
	20	29	47	23	164	Children
IRF8	27	25	55	26	152	Adults
	242	546	357	346	107	Children

LEF1	127	354	412	339	172	Adults
	114	207	338	396	401	Children
PAX5	98	231	295	352	384	Adults
	81	78	38	64	21	Children
RAG1	54	73	32	55	22	Adults
	130	144	104	152	21	Children
RAG2	95	102	81	115	34	Adults
	59	59	63	61	56	Children
SOX4	65	62	57	63	60	Adults
	15	51	369	389	420	Children
TCL1A	11	41	320	386	376	Adults
	807	701	79	179	21	Children
TdT	767	727	139	325	67	Adults
	361	374	314	334	85	Children
VPREB1	325	430	383	387	151	Adults
