# Supplementary material for: Transcriptional Profiling of mRNAs and microRNAs in Human Bone Marrow Precursor B Cells Identifies Subset- and Age-Specific Variations
Source: PLoS One. 2013 Jul 30;8(7):e70721. doi: 10.1371/journal.pone.0070721 (PMC3728296; doi:10.1371/journal.pone.0070721)
Supplement: Table S5 — (PDF) [file pone.0070721.s009.pdf]

Analysis Name: Child_PreBI vs ProB_mRNA
Analysis Creation Date: 2013-06-09
Build version: 220217
Content version: 16542223 (Release Date: 2013-05-13)

Analysis settings

View

Reference set: Ingenuity Knowledge Base (Genes + Endogenous Chemicals)

Relationship to include: Direct and Indirect

Includes Endogenous Chemicals

Optional Analyses: My Pathways My List

Filter Summary:

Consider only molecules and/or relationships where

(species = Rat OR Human OR Mouse) AND

(confidence = Experimentally Observed OR High (predicted))

Cutoff:

Top Networks

ID	Associated Network Functions	Score
1	Cell Cycle, Cancer, Reproductive System Disease	51
2	Cell Cycle, Cell Morphology, Cellular Compromise	40

(c) 2000-2013 Ingenuity Systems, Inc. All rights reserved.

3	Hematological System Development and Function, Tissue Morphology, Cellular Development	35
4	Cellular Development, Hematological System Development and Function, Hematopoiesis	23
5	Embryonic Development, Nervous System Development and Function, Organ Development	23

(c) 2000-2013 Ingenuity Systems, Inc. All rights reserved.

Top Bio Functions

Diseases and Disorders

Name	p-value	# Molecules
Cancer	4,19E-09 - 1,00E-02	69
Hematological Disease	1,29E-08 - 1,00E-02	24
Gastrointestinal Disease	9,10E-08 - 1,00E-02	33
Reproductive System Disease	9,63E-08 - 1,00E-02	31
Respiratory Disease	3,79E-05 - 5,02E-03	40

Molecular and Cellular Functions

Name	p-value	# Molecules
Cell Cycle	6,66E-12 - 1,00E-02	38
Cellular Assembly and Organization	5,46E-09 - 1,00E-02	26
DNA Replication, Recombination, and Repair	5,46E-09 - 1,00E-02	21
Cellular Function and Maintenance	1,33E-08 - 9,20E-03	28
Cellular Growth and Proliferation	3,19E-08 - 1,00E-02	47

Physiological System Development and Function

Name	p-value	# Molecules
Tissue Development	2,17E-06 - 1,00E-02	25
Hematological System Development and Function	3,66E-06 - 1,00E-02	27
Organismal Survival	5,64E-06 - 5,64E-06	15
Lymphoid Tissue Structure and Development	7,87E-06 - 7,02E-03	19
Tumor Morphology	1,00E-05 - 1,00E-02	11

(c) 2000-2013 Ingenuity Systems, Inc. All rights reserved.

INGENUITY® SYSTEMS

Top Canonical Pathways

Name	p-value	Ratio
Mitotic Roles of Polo-Like Kinase	2,14E-05	5/70 (0,071)
Cyclins and Cell Cycle Regulation	5,05E-05	5/90 (0,056)
Cell Cycle: G2/M DNA Damage Checkpoint Regulation	6,87E-05	4/49 (0,082)
DNA damage-induced 14-3-3σ Signaling	1,11E-04	3/21 (0,143)
GADD45 Signaling	1,31E-04	3/23 (0,13)

Top Molecules

Fold Change up-regulated

Molecules	Exp. Value	Exp. Chart
TMSB15A	↑3,363	
TCL1A	↑3,319	
IKZF3	↑3,042	
IKZF2	↑2,965	
PBK	↑2,833	
SUCLG2	↑2,826	
SAMHD1	↑2,780	
GLDC	↑2,597	
DLGAP5	↑2,596	
CDKN3	↑2,491	

Fold Change down-regulated

Molecules	Exp. Value	Exp. Chart
IGJ	↓-6,199	
PROM1	↓-5,031	
SELL	↓-4,373	

(c) 2000-2013 Ingenuity Systems, Inc. All rights reserved.

INGENUITY® SYSTEMS

BAALC	↓-4,209
SCN3A	↓-3,556
NEGR1	↓-3,220
ANKRD36B	↓-3,054
RBMS2	↓-3,046
ANXA1	↓-2,977
TMEM200A	↓-2,948

Top Upstream Regulators

Upstream Regulator	p-value of overlap	Predicted Activation State
CDKN1A	1,22E-13	
CCND1	1,82E-13	
TP53	2,17E-13	Inhibited
KDM5B	8,38E-12	Inhibited
TCF3	4,02E-11	Inhibited

(c) 2000-2013 Ingenuity Systems, Inc. All rights reserved.

Top My Lists

Name	p-value	Ratio
PreBI vs PreBIIL-miR target filter_cell cycle_adul	8,25E-18	15/111 (0,135)
PreBI vs PreBIIL_miR_and mRNA_adults	2,42E-02	3/63 (0,048)
PreBI vs PreBIIL_miR_and mRNA_utvidet_adults	2,91E-02	3/60 (0,05)
PreBI vs PreBII_miR and mRNA_network2_adults	1,14E-01	2/28 (0,071)
PreBI vs PreBII_miR and mRNA_network1_adults	1,49E-01	1/34 (0,029)

Top My Pathways

Name	p-value	Ratio
PreBI vs PreBIIL_miRs and mRNA_voksne	2,91E-02	3/60 (0,05)

Top Tox Lists

Name	p-value	Ratio
Cell Cycle: G2/M DNA Damage Checkpoint Regulation	9,7E-05	4/48 (0,083)
Acute Renal Failure Panel (Rat)	3,87E-02	2/62 (0,032)
Long-term Renal Injury Pro-oxidative Response Panel (Rat)	6,33E-02	1/13 (0,077)
Increases Liver Damage	8,38E-02	2/96 (0,021)
Increases Renal Proliferation	1,2E-01	2/119 (0,017)

(c) 2000-2013 Ingenuity Systems, Inc. All rights reserved.

Top Tox Functions

Assays: Clinical Chemistry and Hematology

Name	p-value	# Molecules
Increased Levels of Blood Urea Nitrogen	6,80E-02 - 6,80E-02	1
Increased Levels of Creatinine	6,80E-02 - 1,62E-01	2
Increased Levels of Alkaline Phosphatase	2,94E-01 - 2,94E-01	1
Increased Levels of Red Blood Cells	3,84E-01 - 3,84E-01	1

Cardiotoxicity

Name	p-value	# Molecules
Cardiac Regeneration	1,00E-02 - 1,00E-02	1
Cardiac Dysfunction	1,50E-02 - 2,48E-02	1
Cardiac Hyperplasia/Hyperproliferation	1,50E-02 - 1,50E-02	1
Cardiac Inflammation	1,50E-02 - 1,50E-02	1
Cardiac Dilation	2,51E-02 - 1,91E-01	2

Hepatotoxicity

Name	p-value	# Molecules
Liver Hyperplasia/Hyperproliferation	1,23E-04 - 2,30E-01	12
Hepatocellular Carcinoma	1,37E-04 - 2,30E-01	10
Liver Inflammation/Hepatitis	3,94E-02 - 3,94E-02	1
Liver Failure	6,80E-02 - 6,80E-02	1
Liver Fibrosis	9,12E-02 - 4,32E-01	2

Nephrotoxicity

Name	p-value	# Molecules
Renal Degeneration	5,02E-03 - 5,02E-03	1

(c) 2000-2013 Ingenuity Systems, Inc. All rights reserved.

Renal Proliferation	9,93E-03 - 9,93E-03	4
Renal Inflammation	1,00E-02 - 3,03E-01	4
Renal Nephritis	1,00E-02 - 3,03E-01	4
Renal Dilation	2,97E-02 - 2,97E-02	1

(c) 2000-2013 Ingenuity Systems, Inc. All rights reserved.

Analysis Name: Child_PreBII L vs PreBI_mRNA
Analysis Creation Date: 2013-06-09
Build version: 220217
Content version: 16542223 (Release Date: 2013-05-13)

Analysis settings

View
Reference set: Ingenuity Knowledge Base (Genes + Endogenous Chemicals)
Relationship to include: Direct and Indirect
Includes Endogenous Chemicals
Optional Analyses: My Pathways My List

Filter Summary:
Consider only molecules and/or relationships where
(species = Rat OR Human OR Mouse) AND
(confidence = Experimentally Observed OR High (predicted))
Cutoff:

Top Networks

ID	Associated Network Functions	Score
1	Cell-To-Cell Signaling and Interaction, Hematological System Development and Function, Immune Cell Trafficking	37
2	Organ Morphology, Skeletal and Muscular System Development and Function, Cellular Assembly and Organization	30

(c) 2000-2013 Ingenuity Systems, Inc. All rights reserved.

3	Cellular Development, Cellular Growth and Proliferation, Hematological System Development and Function	30
4	Post-Translational Modification, Hereditary Disorder, Respiratory Disease	26
5	Cellular Development, Cellular Growth and Proliferation, Hematological System Development and Function	24

(c) 2000-2013 Ingenuity Systems, Inc. All rights reserved.

Top Bio Functions

Diseases and Disorders

Name	p-value	# Molecules
Inflammatory Response	1,34E-07 - 8,88E-03	67
Cancer	1,95E-07 - 8,88E-03	132
Developmental Disorder	2,44E-06 - 6,95E-03	29
Immunological Disease	2,44E-06 - 8,88E-03	45
Hematological Disease	1,09E-05 - 8,88E-03	29

Molecular and Cellular Functions

Name	p-value	# Molecules
Cellular Development	2,20E-13 - 8,88E-03	82
Cellular Growth and Proliferation	2,20E-13 - 8,85E-03	84
Cell Death and Survival	2,82E-09 - 8,69E-03	82
Cellular Movement	8,17E-09 - 7,57E-03	64
Cell-To-Cell Signaling and Interaction	3,72E-08 - 8,88E-03	66

Physiological System Development and Function

Name	p-value	# Molecules
Hematological System Development and Function	9,57E-14 - 8,88E-03	74
Tissue Morphology	9,57E-14 - 8,25E-03	63
Humoral Immune Response	9,14E-11 - 8,12E-03	34
Hematopoiesis	4,91E-10 - 8,45E-03	44
Immune Cell Trafficking	1,34E-07 - 8,88E-03	49

(c) 2000-2013 Ingenuity Systems, Inc. All rights reserved.

INGENUITY SYSTEMS

Top Canonical Pathways

Name	p-value	Ratio
Phospholipase C Signaling	1,27E-05	12/251 (0,048)
Role of NFAT in Cardiac Hypertrophy	2,07E-04	9/197 (0,046)
Thrombin Signaling	2,96E-04	9/201 (0,045)
PI3K Signaling in B Lymphocytes	5,8E-04	7/133 (0,053)
Fcγ Receptor-mediated Phagocytosis in Macrophages and Monocytes	6,04E-04	6/95 (0,063)

Top Molecules

Fold Change up-regulated

Molecules	Exp. Value	Exp. Chart
TNFRSF17	↑9,034	
TCL1A	↑7,187	
ADAM23	↑7,180	
IKZF3	↑7,029	
LAMP5	↑6,568	
IGJ	↑5,962	
IGK	↑5,794	
BTLA	↑5,451	
LYN	↑5,032	
KMO	↑4,414	

Fold Change down-regulated

Molecules	Exp. Value	Exp. Chart
RPS4Y2	↓-35,799	
ERG	↓-8,946	

(c) 2000-2013 Ingenuity Systems, Inc. All rights reserved.

DNTT	↓-8,862
CD34	↓-7,047
GBP4	↓-6,376
PECAM1	↓-6,102
NDUFAF2	↓-6,064
SCHIP1	↓-5,497
HGSNAT	↓-5,493
SPANXC (includes others)	↓-5,451

Top Upstream Regulators

Upstream Regulator	p-value of overlap	Predicted Activation State
IL2	2,97E-07	
miR-96-5p (and other miRNAs w/seed UUGGCAC)	4,29E-07	
TGFB1	6,56E-07	
IL15	1,32E-06	
SPIB	1,60E-06	

(c) 2000-2013 Ingenuity Systems, Inc. All rights reserved.

INGENUITY® SYSTEMS

Top My Lists

Name	p-value	Ratio
PreBI vs PreBIIL-miR target filter_cell cycle_adul	9,68E-14	16/111 (0,144)
PreBI vs PreBII_miR target filter_cell cycle_ID2_c	1,31E-10	10/51 (0,196)
miR-126 PreBI/PreBII large children	1,95E-07	5/15 (0,333)
PreBI vs PreBIIL_miR_and mRNA_utvidet_adults	1,91E-06	7/60 (0,117)
PreBI vs PreBIIL_miR_and mRNA_adults	1,47E-05	6/63 (0,095)

Top My Pathways

Name	p-value	Ratio
mir-126 PreBI/PreBII large children	1,95E-07	5/15 (0,333)
PreBI vs PreBIIL_miRs and mRNA_voksne	1,91E-06	7/60 (0,117)
PreBI vs PreBII L_miR og mRNA_core TF_barn	1,56E-03	4/26 (0,154)

Top Tox Lists

Name	p-value	Ratio
Renal Necrosis/Cell Death	6,08E-05	16/461 (0,035)
Cardiac Hypertrophy	1,32E-04	13/344 (0,038)
Increases Cardiac Dilation	3,27E-03	3/27 (0,111)
Increases Liver Damage	4,39E-03	5/96 (0,052)
Increases Cardiac Dysfunction	6,32E-03	3/34 (0,088)

(c) 2000-2013 Ingenuity Systems, Inc. All rights reserved.

INGENUITY® SYSTEMS

Top Tox Functions

Assays: Clinical Chemistry and Hematology

Name	p-value	# Molecules
Increased Levels of Alkaline Phosphatase	4,17E-02 - 4,17E-02	3
Increased Levels of Creatinine	3,25E-01 - 3,25E-01	1

Cardiotoxicity

Name	p-value	# Molecules
Cardiac Dilation	2,09E-04 - 3,13E-02	6
Heart Failure	1,79E-03 - 1,10E-01	9
Cardiac Infarction	3,73E-03 - 3,73E-03	7
Cardiac Hypertrophy	6,95E-03 - 4,96E-01	13
Cardiac Dysfunction	1,68E-02 - 7,63E-02	4

Hepatotoxicity

Name	p-value	# Molecules
Liver Inflammation/Hepatitis	5,58E-05 - 9,30E-02	12
Liver Cirrhosis	6,39E-04 - 1,11E-02	7
Liver Steatosis	9,94E-03 - 3,32E-01	4
Liver Cholestasis	1,11E-02 - 7,54E-02	1
Liver Adhesion	2,22E-02 - 2,22E-02	1

Nephrotoxicity

Name	p-value	# Molecules
Renal Necrosis/Cell Death	9,61E-06 - 2,27E-01	16
Renal Inflammation	3,30E-03 - 1,55E-01	9
Renal Nephritis	3,30E-03 - 1,55E-01	9

(c) 2000-2013 Ingenuity Systems, Inc. All rights reserved.

Kidney Failure	5,96E-03 - 4,48E-01	7
Nephrosis	1,03E-02 - 2,45E-02	3

(c) 2000-2013 Ingenuity Systems, Inc. All rights reserved.

Analysis Name: Child_PreBII s vs PreBII L_mRNA
Analysis Creation Date: 2013-06-09
Build version: 220217
Content version: 16542223 (Release Date: 2013-05-13)

Analysis settings

View
Reference set: Ingenuity Knowledge Base (Genes + Endogenous Chemicals)
Relationship to include: Direct and Indirect
Includes Endogenous Chemicals
Optional Analyses: My Pathways My List

Filter Summary:
Consider only molecules and/or relationships where
(species = Rat OR Human OR Mouse) AND
(confidence = Experimentally Observed OR High (predicted))
Cutoff:

Top Networks

ID	Associated Network Functions	Score
1	Inflammatory Response, Cell-To-Cell Signaling and Interaction, Cellular Movement	20

(c) 2000-2013 Ingenuity Systems, Inc. All rights reserved.

(c) 2000-2013 Ingenuity Systems, Inc. All rights reserved.

Top Bio Functions

Diseases and Disorders

Name	p-value	# Molecules
Cancer	5,25E-04 - 3,30E-02	8
Connective Tissue Disorders	5,25E-04 - 5,25E-04	1
Dermatological Diseases and Conditions	5,25E-04 - 2,49E-02	3
Developmental Disorder	5,25E-04 - 5,25E-04	1
Hereditary Disorder	5,25E-04 - 5,25E-04	2

Molecular and Cellular Functions

Name	p-value	# Molecules
Cellular Movement	1,05E-03 - 3,21E-02	1
Drug Metabolism	1,05E-03 - 4,19E-03	1
Small Molecule Biochemistry	1,05E-03 - 7,33E-03	3
Cell-To-Cell Signaling and Interaction	1,57E-03 - 3,46E-02	3
Lipid Metabolism	1,57E-03 - 5,76E-03	2

Physiological System Development and Function

Name	p-value	# Molecules
Hematological System Development and Function	1,05E-03 - 3,46E-02	2
Immune Cell Trafficking	1,05E-03 - 3,46E-02	1
Tissue Development	1,05E-03 - 3,46E-02	2
Behavior	1,57E-03 - 1,57E-03	1
Cardiovascular System Development and Function	1,57E-03 - 2,24E-02	2

(c) 2000-2013 Ingenuity Systems, Inc. All rights reserved.

Top Canonical Pathways

Name	p-value	Ratio
Bupropion Degradation	1,36E-02	1/26 (0,038)
Acetone Degradation I (to Methylglyoxal)	1,41E-02	1/27 (0,037)
B Cell Development	1,67E-02	1/33 (0,03)
Estrogen Biosynthesis	1,98E-02	1/38 (0,026)
Nicotine Degradation III	2,7E-02	1/52 (0,019)

Top Molecules

Fold Change up-regulated

Molecules	Exp. Value	Exp. Chart
CYP2A6	↑3,769	
RNF111	↑2,339	
DNTT	↑2,263	
CXCL2	↑2,219	
OR2L3	↑2,034	
OR2M5	↑2,002	

Fold Change down-regulated

Molecules	Exp. Value	Exp. Chart
SAMHD1	↓-2,390	
TLR6	↓-2,080	
SYP	↓-2,021	

Top Upstream Regulators

(c) 2000-2013 Ingenuity Systems, Inc. All rights reserved.

INGENUITY® SYSTEMS

Upstream Regulator	p-value of overlap	Predicted Activation State
SERPINE1	4,26E-05	
VIP	5,35E-04	
Rsk	7,02E-04	
LDL-cholesterol	7,02E-04	
TICAM1	9,33E-04	

(c) 2000-2013 Ingenuity Systems, Inc. All rights reserved.

Top My Lists

Name	p-value	Ratio
PreBI vs PreBII_miR and mRNA_network1_adults	1,67E-02	1/34 (0,029)
PreBI vs PreBIIL_miR_and mRNA_adults	2,49E-02	1/63 (0,016)
PreBI vs PreBIIL_miR_and mRNA_utvidet_adults	2,75E-02	1/60 (0,017)

Top My Pathways

Name	p-value	Ratio
PreBI vs PreBIIL_miRs and mRNA_voksne	2,75E-02	1/60 (0,017)

Top Tox Lists

Name	p-value	Ratio
Cytochrome P450 Panel - Substrate is a Xenobiotic (Human)	1,05E-02	1/20 (0,05)
Persistent Renal Ischemia-Reperfusion Injury (Mouse)	1,56E-02	1/30 (0,033)
Acute Renal Failure Panel (Rat)	3,21E-02	1/62 (0,016)
PXR/RXR Activation	3,46E-02	1/67 (0,015)
Hepatic Fibrosis	4,93E-02	1/96 (0,01)

(c) 2000-2013 Ingenuity Systems, Inc. All rights reserved.

Top Tox Functions

Hepatotoxicity

Name	p-value	# Molecules
Liver Inflammation/Hepatitis	1,67E-02 - 1,67E-02	1

(c) 2000-2013 Ingenuity Systems, Inc. All rights reserved.

Analysis Name: Child_Immature B vs PreBII s_mRNA
Analysis Creation Date: 2013-06-09
Build version: 220217
Content version: 16542223 (Release Date: 2013-05-13)

Analysis settings

View
Reference set: Ingenuity Knowledge Base (Genes + Endogenous Chemicals)
Relationship to include: Direct and Indirect
Includes Endogenous Chemicals
Optional Analyses: My Pathways My List

Filter Summary:
Consider only molecules and/or relationships where
(species = Rat OR Human OR Mouse) AND
(confidence = Experimentally Observed OR High (predicted))
Cutoff:

Top Networks

ID	Associated Network Functions	Score
1	DNA Replication, Recombination, and Repair, Gene Expression, Cell Cycle	51
2	DNA Replication, Recombination, and Repair, Protein Synthesis, Cell Cycle	51

(c) 2000-2013 Ingenuity Systems, Inc. All rights reserved.

3	Cellular Assembly and Organization, Cell Cycle, DNA Replication, Recombination, and Repair	43
4	DNA Replication, Recombination, and Repair, Cell Cycle, Cellular Assembly and Organization	41
5	Cell Cycle, Cellular Assembly and Organization, DNA Replication, Recombination, and Repair	39

(c) 2000-2013 Ingenuity Systems, Inc. All rights reserved.

Top Bio Functions

Diseases and Disorders

Name	p-value	# Molecules
Cancer	3,46E-17 - 1,01E-03	369
Hematological Disease	3,46E-17 - 1,01E-03	105
Gastrointestinal Disease	8,58E-16 - 9,39E-04	146
Reproductive System Disease	1,24E-10 - 1,71E-04	79
Immunological Disease	1,10E-09 - 1,01E-03	114

Molecular and Cellular Functions

Name	p-value	# Molecules
Cell Cycle	1,11E-28 - 1,01E-03	182
Cellular Assembly and Organization	1,11E-28 - 1,01E-03	85
DNA Replication, Recombination, and Repair	1,11E-28 - 1,01E-03	157
Cellular Growth and Proliferation	3,98E-16 - 1,01E-03	227
Cell Death and Survival	9,08E-13 - 1,01E-03	219

Physiological System Development and Function

Name	p-value	# Molecules
Hematological System Development and Function	2,70E-10 - 1,01E-03	134
Humoral Immune Response	2,70E-10 - 1,01E-03	68
Tissue Morphology	3,73E-10 - 9,99E-04	139
Hematopoiesis	6,70E-09 - 1,01E-03	84
Lymphoid Tissue Structure and Development	8,93E-08 - 1,01E-03	85

(c) 2000-2013 Ingenuity Systems, Inc. All rights reserved.

INGENUITY® SYSTEMS

Top Canonical Pathways

Name	p-value	Ratio
Cell Cycle Control of Chromosomal Replication	7,61E-09	10/31 (0,323)
Mitotic Roles of Polo-Like Kinase	1,89E-08	14/70 (0,2)
GADD45 Signaling	8,91E-08	8/23 (0,348)
Mismatch Repair in Eukaryotes	2,81E-07	7/20 (0,35)
Role of BRCA1 in DNA Damage Response	3,29E-07	12/63 (0,19)

Top Molecules

Fold Change up-regulated

Molecules	Exp. Value	Exp. Chart
SP140	↑23,386	
BTLA	↑13,952	
CYBB	↑10,574	
MS4A1	↑9,862	
ANGPTL1	↑8,347	
RALGPS2	↑8,193	
CD1C	↑7,511	
SLC2A3	↑7,456	
HLA-DOB	↑7,070	
TLR10	↑7,065	

Fold Change down-regulated

Molecules	Exp. Value	Exp. Chart
DNTT	↓-8,148	
RAG2	↓-7,824	
SPC25	↓-7,160	

(c) 2000-2013 Ingenuity Systems, Inc. All rights reserved.

INGENUITY® SYSTEMS

MKI67	↓-7,018
KIAA0101	↓-6,733
HIST1H2BB	↓-6,729
DLGAP5	↓-6,463
HIST1H3A (includes others)*	↓-6,304
HIST1H2BM	↓-5,975
CENPF	↓-5,896

Top Upstream Regulators

Upstream Regulator	p-value of overlap	Predicted Activation State
CDKN1A	7,56E-49	Activated
E2F4	5,71E-44	
TP53	9,45E-35	Activated
CSF2	1,63E-32	Inhibited
CCND1	2,55E-31	Inhibited

(c) 2000-2013 Ingenuity Systems, Inc. All rights reserved.

INGENUITY® SYSTEMS

Top My Lists

Name	p-value	Ratio
PreBI vs PreBIIL-miR target filter_cell cycle_adul	3,01E-29	38/111 (0,342)
PreBI vs PreBII_miR target filter_cell cycle_ID2_c	2,61E-06	10/51 (0,196)
PreBI vs PreBII L barn_miR_utvidet mRNA_Core TFs	4,36E-03	3/16 (0,188)
PreBI vs PreBII_miR and mRNA_network1_adults	1,79E-02	4/34 (0,118)
PreBI vs PreBIIL_miR_and mRNA_utvidet_adults	2,61E-02	6/60 (0,1)

Top My Pathways

Name	p-value	Ratio
PreBI vs PreBII L_miR og mRNA_core TF_barn	3,93E-03	4/26 (0,154)
PreBI vs PreBIIL_miRs and mRNA_voksne	2,61E-02	6/60 (0,1)
mir-126 PreBI/PreBII large children	6,24E-02	2/15 (0,133)

Top Tox Lists

Name	p-value	Ratio
Cell Cycle: G2/M DNA Damage Checkpoint Regulation	1,72E-05	9/48 (0,188)
Increases Liver Hyperplasia/Hyperproliferation	9,46E-05	9/59 (0,153)
Cell Cycle: G1/S Checkpoint Regulation	4,46E-03	7/65 (0,108)
p53 Signaling	1,07E-02	8/95 (0,084)
Renal Necrosis/Cell Death	1,27E-02	24/461 (0,052)

(c) 2000-2013 Ingenuity Systems, Inc. All rights reserved.

INGENUITY® SYSTEMS

Top Tox Functions

Assays: Clinical Chemistry and Hematology

Name	p-value	# Molecules
Increased Levels of Bilirubin	6,26E-02 - 6,26E-02	1
Increased Levels of Alkaline Phosphatase	6,83E-02 - 6,83E-02	5
Increased Levels of Red Blood Cells	8,54E-02 - 8,54E-02	6
Increased Levels of Hematocrit	1,96E-01 - 1,96E-01	5
Increased Levels of AST	3,21E-01 - 3,21E-01	1

Cardiotoxicity

Name	p-value	# Molecules
Congenital Heart Anomaly	2,12E-02 - 5,15E-01	5
Cardiac Hypertrophy	2,78E-02 - 1,00E00	12
Cardiac Hypoplasia	3,14E-02 - 2,76E-01	4
Cardiac Necrosis/Cell Death	4,77E-02 - 9,24E-02	12
Cardiac Regeneration	6,26E-02 - 6,26E-02	1

Hepatotoxicity

Name	p-value	# Molecules
Hepatocellular Carcinoma	8,42E-09 - 1,12E-01	41
Liver Hyperplasia/Hyperproliferation	8,42E-09 - 3,84E-01	47
Liver Inflammation/Hepatitis	2,80E-03 - 6,21E-01	18
Biliary Hyperplasia	9,46E-03 - 9,46E-03	2
Liver Cirrhosis	1,01E-02 - 3,18E-02	11

Nephrotoxicity

(c) 2000-2013 Ingenuity Systems, Inc. All rights reserved.

Name	p-value	# Molecules
Kidney Failure	6,50E-03 - 1,00E00	13
Renal Necrosis/Cell Death	8,28E-03 - 5,25E-01	24
Glomerular Injury	3,18E-02 - 4,19E-01	4
Renal Destruction	3,18E-02 - 3,18E-02	1
Renal Fibrosis	6,26E-02 - 4,19E-01	3

(c) 2000-2013 Ingenuity Systems, Inc. All rights reserved.
