# Supplementary material for: Transcriptional Profiling of mRNAs and microRNAs in Human Bone Marrow Precursor B Cells Identifies Subset- and Age-Specific Variations
Source: PLoS One. 2013 Jul 30;8(7):e70721. doi: 10.1371/journal.pone.0070721 (PMC3728296; doi:10.1371/journal.pone.0070721)
Supplement: Table S6 — (PDF) [file pone.0070721.s010.pdf]

Analysis Name: Adult_PreBI vs ProB_mRNA
Analysis Creation Date: 2013-06-09
Build version: 220217
Content version: 16542223 (Release Date: 2013-05-13)

Analysis settings

View

Reference set: Ingenuity Knowledge Base (Genes Only)

Relationship to include: Direct and Indirect

Includes Endogenous Chemicals

Optional Analyses: My Pathways My List

Filter Summary:

Consider only molecules and/or relationships where

(species = Rat OR Human OR Mouse) AND

(confidence = Experimentally Observed OR High (predicted))

Cutoff:

Top Networks

ID	Associated Network Functions	Score
1	Hematological System Development and Function, Tissue Morphology, Cellular Development	40
2	Hematological System Development and Function, Tissue Morphology, Cell Cycle	33

(c) 2000-2013 Ingenuity Systems, Inc. All rights reserved.

3	Cancer, Cell Death and Survival, Tumor Morphology	28
4	Cell Morphology, Hair and Skin Development and Function, Lymphoid Tissue Structure and Development	24
5	Organismal Development, Infectious Disease, Digestive System Development and Function	24

(c) 2000-2013 Ingenuity Systems, Inc. All rights reserved.

Top Bio Functions

Diseases and Disorders

Name	p-value	# Molecules
Cancer	2,70E-09 - 2,63E-03	100
Inflammatory Response	4,17E-08 - 2,43E-03	46
Infectious Disease	6,53E-08 - 2,07E-03	38
Respiratory Disease	6,53E-08 - 2,25E-03	18
Hematological Disease	1,89E-07 - 2,34E-03	31

Molecular and Cellular Functions

Name	p-value	# Molecules
Cell Death and Survival	8,29E-10 - 2,31E-03	67
Cellular Development	2,18E-09 - 2,54E-03	63
Cellular Growth and Proliferation	2,18E-09 - 2,22E-03	68
Cell-To-Cell Signaling and Interaction	7,66E-09 - 2,43E-03	39
Cellular Compromise	7,66E-09 - 1,80E-03	12

Physiological System Development and Function

Name	p-value	# Molecules
Hematological System Development and Function	5,98E-12 - 2,63E-03	53
Tissue Morphology	5,98E-12 - 2,65E-03	49
Humoral Immune Response	3,64E-10 - 2,03E-03	31
Tissue Development	7,66E-09 - 2,68E-03	45
Tumor Morphology	7,66E-09 - 1,49E-03	17

(c) 2000-2013 Ingenuity Systems, Inc. All rights reserved.

Top Canonical Pathways

Name	p-value	Ratio
Ovarian Cancer Signaling	4,71E-03	5/140 (0,036)
Hepatic Fibrosis / Hepatic Stellate Cell Activation	5,67E-03	5/142 (0,035)
β-alanine Degradation I	1,63E-02	1/2 (0,5)
Choline Degradation I	1,63E-02	1/2 (0,5)
L-glutamine Biosynthesis II (tRNA-dependent)	1,63E-02	1/2 (0,5)

Top Molecules

Fold Change up-regulated

Molecules	Exp. Value	Exp. Chart
TCL1A	↑3,802	
KIAA0226L	↑3,276	
LIG4	↑3,250	
CD24	↑3,187	
NDUFAF2	↑3,118	
PSD3*	↑3,025	
VPREB3	↑2,951	
ABAT	↑2,808	
LEF1	↑2,796	
IKZF2	↑2,790	

Fold Change down-regulated

Molecules	Exp. Value	Exp. Chart
PROM1	↓-7,719	
IGJ	↓-7,622	
BAALC	↓-7,042	

(c) 2000-2013 Ingenuity Systems, Inc. All rights reserved.

INGENUITY® SYSTEMS

ANXA1	↓-6,246
NFIL3	↓-5,028
CPED1	↓-4,962
MOB1B	↓-4,613
TMEM200A	↓-4,568
IL7R	↓-4,519
ADAM28	↓-4,489

Top Upstream Regulators

Upstream Regulator	p-value of overlap	Predicted Activation State
WNT3A	5,56E-09	
PTGS2	1,68E-07	Inhibited
IL2	2,79E-07	
FOXP3	4,16E-07	
CSF3	5,99E-07	

(c) 2000-2013 Ingenuity Systems, Inc. All rights reserved.

INGENUITY® SYSTEMS

Top My Lists

Name	p-value	Ratio
PreBI vs PreBIIL-miR target filter_cell cycle_adul	2,99E-04	6/111 (0,054)
PreBI vs PreBIIL_miR_and mRNA_utvidet_adults	9,27E-03	3/60 (0,05)
PreBI vs PreBII_miR and mRNA_network2_adults	1,62E-02	2/28 (0,071)
PreBI vs PreBIIL_miR_and mRNA_adults	5,85E-02	2/63 (0,032)
PreBI vs PreBII_miR target filter_cell cycle_ID2_c	6,07E-02	2/51 (0,039)

Top My Pathways

Name	p-value	Ratio
PreBI vs PreBIIL_miRs and mRNA_voksne	9,27E-03	3/60 (0,05)
PreBI vs PreBII L_miR og mRNA_core TF_barn	1,58E-01	1/26 (0,038)

Top Tox Lists

Name	p-value	Ratio
Acute Renal Failure Panel (Rat)	1,51E-04	5/62 (0,081)
Genes associated with Chronic Allograft Nephropathy (Human)	1,26E-02	2/21 (0,095)
Increases Renal Nephritis	4,03E-02	2/39 (0,051)
p53 Signaling	4,28E-02	3/95 (0,032)
Increases Liver Damage	4,4E-02	3/96 (0,031)

(c) 2000-2013 Ingenuity Systems, Inc. All rights reserved.

Top Tox Functions

Assays: Clinical Chemistry and Hematology

Name	p-value	# Molecules
Increased Levels of Albumin	8,17E-03 - 8,17E-03	1
Increased Levels of Red Blood Cells	4,40E-02 - 4,40E-02	3
Increased Levels of Blood Urea Nitrogen	1,08E-01 - 1,08E-01	1
Increased Levels of Hematocrit	1,88E-01 - 1,88E-01	2
Increased Levels of Creatinine	2,50E-01 - 2,50E-01	1

Cardiotoxicity

Name	p-value	# Molecules
Cardiac Proliferation	8,17E-03 - 1,20E-01	2
Cardiac Fibrosis	1,47E-02 - 1,51E-01	4
Cardiac Infarction	1,51E-02 - 3,79E-01	5
Cardiac Inflammation	2,43E-02 - 2,80E-01	2
Cardiac Hemorrhaging	4,80E-02 - 4,80E-02	1

Hepatotoxicity

Name	p-value	# Molecules
Liver Hyperplasia/Hyperproliferation	1,16E-03 - 3,79E-01	14
Liver Fibrosis	5,89E-03 - 2,33E-01	6
Hepatocellular Carcinoma	5,90E-03 - 3,79E-01	10
Liver Regeneration	1,48E-02 - 1,48E-02	3
Liver Cholestasis	3,23E-02 - 3,84E-01	2

Nephrotoxicity

(c) 2000-2013 Ingenuity Systems, Inc. All rights reserved.

Name	p-value	# Molecules
Glomerular Injury	8,17E-03 - 3,09E-01	4
Renal Destruction	8,17E-03 - 8,17E-03	1
Renal Hypertrophy	1,50E-02 - 7,87E-02	3
Nephrosis	7,66E-02 - 7,66E-02	2
Renal Necrosis/Cell Death	7,87E-02 - 4,14E-01	5

(c) 2000-2013 Ingenuity Systems, Inc. All rights reserved.

Analysis Name: Adult_PreBII L vs PreBI_mRNA
Analysis Creation Date: 2013-06-09
Build version: 220217
Content version: 16542223 (Release Date: 2013-05-13)

Analysis settings

View
Reference set: Ingenuity Knowledge Base (Genes Only)
Relationship to include: Direct and Indirect
Includes Endogenous Chemicals
Optional Analyses: My Pathways My List

Filter Summary:
Consider only molecules and/or relationships where
(species = Rat OR Human OR Mouse) AND
(confidence = Experimentally Observed OR High (predicted))
Cutoff:

Top Networks

ID	Associated Network Functions	Score
1	Cell Cycle, Cancer, Endocrine System Disorders	43
2	Cellular Development, Embryonic Development, Organismal Development	39

(c) 2000-2013 Ingenuity Systems, Inc. All rights reserved.

3	Molecular Transport, Nucleic Acid Metabolism, Small Molecule Biochemistry	37
4	Cell Cycle, Cellular Assembly and Organization, DNA Replication, Recombination, and Repair	35
5	DNA Replication, Recombination, and Repair, Cell Cycle, Connective Tissue Disorders	35

(c) 2000-2013 Ingenuity Systems, Inc. All rights reserved.

Top Bio Functions

Diseases and Disorders

Name	p-value	# Molecules
Cancer	3,63E-13 - 4,32E-03	340
Hematological Disease	3,63E-13 - 3,14E-03	91
Gastrointestinal Disease	5,70E-08 - 4,05E-03	115
Immunological Disease	2,78E-07 - 3,14E-03	77
Connective Tissue Disorders	1,67E-06 - 2,61E-03	15

Molecular and Cellular Functions

Name	p-value	# Molecules
Cell Cycle	2,29E-16 - 3,72E-03	147
Cellular Assembly and Organization	2,68E-15 - 3,72E-03	114
DNA Replication, Recombination, and Repair	2,68E-15 - 3,72E-03	129
Cellular Growth and Proliferation	3,82E-12 - 2,61E-03	203
Cell Death and Survival	1,21E-10 - 4,09E-03	199

Physiological System Development and Function

Name	p-value	# Molecules
Hematological System Development and Function	3,85E-07 - 4,05E-03	109
Hematopoiesis	4,95E-07 - 4,05E-03	76
Humoral Immune Response	5,68E-07 - 3,54E-03	51
Tissue Morphology	9,81E-07 - 3,00E-03	99
Reproductive System Development and Function	1,20E-06 - 2,61E-03	19

(c) 2000-2013 Ingenuity Systems, Inc. All rights reserved.

INGENUITY® SYSTEMS

Top Canonical Pathways

Name	p-value	Ratio
Role of BRCA1 in DNA Damage Response	1,8E-09	14/63 (0,222)
Mismatch Repair in Eukaryotes	6,15E-09	8/20 (0,4)
GADD45 Signaling	5,42E-08	8/23 (0,348)
Role of CHK Proteins in Cell Cycle Checkpoint Control	5,91E-08	12/57 (0,211)
Hereditary Breast Cancer Signaling	5,95E-08	17/124 (0,137)

Top Molecules

Fold Change up-regulated

Molecules	Exp. Value	Exp. Chart
IKZF3	↑11,666	
SAMHD1	↑10,652	
TCL1A	↑9,706	
CAMK4	↑9,016	
TNFRSF17	↑9,008	
IGJ	↑6,728	
WAPAL	↑6,590	
LYN	↑6,323	
CAMK2D	↑6,240	
APOBEC3B	↑6,196	

Fold Change down-regulated

Molecules	Exp. Value	Exp. Chart
ERG	↓-8,177	
GNG11	↓-7,779	

(c) 2000-2013 Ingenuity Systems, Inc. All rights reserved.

INGENUITY® SYSTEMS

PECAM1	↓-7,253
OPALIN	↓-6,713
MYO5C	↓-6,440
GIMAP4	↓-5,659
SCHIP1	↓-5,539
CD34	↓-5,435
DNTT	↓-5,155
ELK3	↓-4,980

Top Upstream Regulators

Upstream Regulator	p-value of overlap	Predicted Activation State
E2F4	3,71E-31	
CCND1	1,13E-25	Activated
CDKN1A	3,05E-25	Inhibited
let-7	7,81E-25	Inhibited
TP53	6,37E-23	Inhibited

(c) 2000-2013 Ingenuity Systems, Inc. All rights reserved.

INGENUITY® SYSTEMS

Top My Lists

Name	p-value	Ratio
PreBI vs PreBIIL-miR target filter_cell cycle_adul	1,04E-46	50/111 (0,45)
PreBI vs PreBIIL_miR_and mRNA_utvidet_adults	3,03E-10	16/60 (0,267)
PreBI vs PreBII_miR target filter_cell cycle_ID2_c	1,24E-09	13/51 (0,255)
PreBI vs PreBIIL_miR_and mRNA_adults	1,23E-07	18/63 (0,286)
PreBI vs PreBII_miR and mRNA_network1_adults	2,63E-07	10/34 (0,294)

Top My Pathways

Name	p-value	Ratio
PreBI vs PreBIIL_miRs and mRNA_voksne	3,03E-10	16/60 (0,267)
mir-126 PreBI/PreBII large children	4,5E-04	4/15 (0,267)
PreBI vs PreBII L_miR og mRNA_core TF_barn	3,12E-03	5/26 (0,192)

Top Tox Lists

Name	p-value	Ratio
Cell Cycle: G2/M DNA Damage Checkpoint Regulation	5,12E-04	7/48 (0,146)
Renal Necrosis/Cell Death	3,05E-03	25/461 (0,054)
p53 Signaling	7,44E-03	8/95 (0,084)
Cell Cycle: G1/S Checkpoint Regulation	1,27E-02	6/65 (0,092)
Increases Transmembrane Potential of Mitochondria and Mitochondrial Membrane	1,62E-02	5/50 (0,1)

(c) 2000-2013 Ingenuity Systems, Inc. All rights reserved.

INGENUITY® SYSTEMS

Top Tox Functions

Assays: Clinical Chemistry and Hematology

Name	p-value	# Molecules
Increased Levels of Albumin	2,98E-02 - 2,98E-02	1
Increased Levels of Alkaline Phosphatase	5,47E-02 - 5,47E-02	5
Decreased Levels of Albumin	1,14E-01 - 1,14E-01	1
Increased Levels of ALT	1,66E-01 - 1,66E-01	1
Increased Levels of Creatinine	2,80E-01 - 2,80E-01	2

Cardiotoxicity

Name	p-value	# Molecules
Cardiac Fibrosis	8,42E-04 - 3,14E-01	5
Cardiac Dilation	9,43E-04 - 1,70E-01	8
Congenital Heart Anomaly	1,79E-02 - 6,21E-01	3
Cardiac Necrosis/Cell Death	2,47E-02 - 1,81E-01	11
Cardiac Proliferation	5,87E-02 - 1,00E00	3

Hepatotoxicity

Name	p-value	# Molecules
Hepatocellular Carcinoma	1,83E-05 - 2,49E-01	32
Liver Hyperplasia/Hyperproliferation	1,83E-05 - 3,65E-01	38
Liver Damage	2,35E-02 - 3,25E-01	5
Liver Cirrhosis	2,98E-02 - 3,76E-01	9
Liver Inflammation/Hepatitis	2,98E-02 - 4,00E-01	14

Nephrotoxicity

(c) 2000-2013 Ingenuity Systems, Inc. All rights reserved.

INGENUITY SYSTEMS

Name	p-value	# Molecules
Renal Necrosis/Cell Death	3,92E-04 - 5,02E-01	25
Renal Degeneration	2,98E-02 - 2,98E-02	1
Renal Inflammation	5,87E-02 - 5,02E-01	12
Renal Nephritis	5,87E-02 - 5,02E-01	12
Kidney Failure	7,28E-02 - 4,21E-01	8

(c) 2000-2013 Ingenuity Systems, Inc. All rights reserved.

IPA Interactive pathway analysis of complex 'omics data

INGENUITY SYSTEMS

Analysis Name: Adult_PreBII s vs PreBII L_mRNA
Analysis Creation Date: 2013-06-09
Build version: 220217
Content version: 16542223 (Release Date: 2013-05-13)

Analysis settings

View

Reference set: Ingenuity Knowledge Base (Genes Only)

Relationship to include: Direct and Indirect

Includes Endogenous Chemicals

Optional Analyses: My Pathways My List

Filter Summary:

Consider only molecules and/or relationships where

(species = Rat OR Human OR Mouse) AND

(confidence = Experimentally Observed OR High (predicted))

Cutoff:

Top Networks

ID	Associated Network Functions	Score
1	DNA Replication, Recombination, and Repair, Cancer, Skeletal and Muscular Disorders	47
2	Cell-To-Cell Signaling and Interaction, Nervous System Development and Function, Cell Death and Survival	34

(c) 2000-2013 Ingenuity Systems, Inc. All rights reserved.

3	Cardiac Dysfunction, Cardiovascular Disease, Lipid Metabolism	31
4	Cell Signaling, Post-Translational Modification, Protein Synthesis	29
5	Cardiovascular Disease, Cell Cycle, Nucleic Acid Metabolism	27

(c) 2000-2013 Ingenuity Systems, Inc. All rights reserved.

Top Bio Functions

Diseases and Disorders

Name	p-value	# Molecules
Cancer	3,32E-05 - 4,78E-02	67
Skeletal and Muscular Disorders	3,32E-05 - 4,79E-02	19
Gastrointestinal Disease	4,03E-04 - 2,15E-02	28
Cardiovascular Disease	1,27E-03 - 3,74E-02	4
Auditory Disease	5,42E-03 - 5,42E-03	1

Molecular and Cellular Functions

Name	p-value	# Molecules
Cellular Assembly and Organization	1,29E-05 - 4,26E-02	18
DNA Replication, Recombination, and Repair	1,29E-05 - 4,14E-02	17
Cell Cycle	2,14E-05 - 4,78E-02	18
Cellular Function and Maintenance	5,97E-04 - 4,78E-02	18
Cell Death and Survival	6,05E-04 - 4,78E-02	30

Physiological System Development and Function

Name	p-value	# Molecules
Tissue Development	5,97E-04 - 4,86E-02	12
Cardiovascular System Development and Function	2,54E-03 - 4,86E-02	6
Hematological System Development and Function	3,75E-03 - 4,26E-02	10
Humoral Immune Response	3,75E-03 - 3,21E-02	5
Tissue Morphology	3,75E-03 - 4,78E-02	7

(c) 2000-2013 Ingenuity Systems, Inc. All rights reserved.

Top Canonical Pathways

Name	p-value	Ratio
Xanthine and Xanthosine Salvage	5,42E-03	1/1 (1)
Tryptophan Degradation III (Eukaryotic)	5,72E-03	2/21 (0,095)
Cell Cycle Control of Chromosomal Replication	1E-02	2/31 (0,065)
Guanine and Guanosine Salvage I	1,08E-02	1/2 (0,5)
Adenine and Adenosine Salvage I	1,08E-02	1/2 (0,5)

Top Molecules

Fold Change up-regulated

Molecules	Exp. Value	Exp. Chart
GNG11	↑3,151	
ERG	↑3,067	
DNTT	↑2,934	
PECAM1	↑2,903	
OR10G9	↑2,804	
FHIT	↑2,762	
GIMAP4	↑2,754	
GBP4	↑2,491	
OPALIN	↑2,452	
FCER1A	↑2,363	

Fold Change down-regulated

Molecules	Exp. Value	Exp. Chart
SAMHD1	↓-3,928	
ID2	↓-3,627	
SCIMP	↓-3,486	

(c) 2000-2013 Ingenuity Systems, Inc. All rights reserved.

RPL31	↓-3,124
RPL6	↓-3,119
HIST1H2BB	↓-2,921
GLDC	↓-2,887
FAM127A	↓-2,810
STAP1	↓-2,805
LYPLA1	↓-2,788

Top Upstream Regulators

Upstream Regulator	p-value of overlap	Predicted Activation State
PBRM1	1,20E-05	
miR-881-3p (and other miRNAs w/seed ACUGUGU)	3,79E-05	Activated
E2F4	1,60E-04	
HGF	2,36E-04	Inhibited
FOXO1	2,41E-04	Inhibited

(c) 2000-2013 Ingenuity Systems, Inc. All rights reserved.

Top My Lists

Name	p-value	Ratio
PreBI vs PreBIIL-miR target filter_cell cycle_adul	3,1E-05	6/111 (0,054)
PreBI vs PreBIIL_miR_and mRNA_utvidet_adults	1,94E-04	4/60 (0,067)
PreBI vs PreBII_miR and mRNA_network1_adults	6,84E-04	3/34 (0,088)
PreBI vs PreBIIL_miR_and mRNA_adults	2,79E-02	3/63 (0,048)
PreBI vs PreBII_miR target filter_cell cycle_ID2_c	2,9E-02	2/51 (0,039)

Top My Pathways

Name	p-value	Ratio
PreBI vs PreBIIL_miRs and mRNA_voksne	1,94E-04	4/60 (0,067)
PreBI vs PreBII L_miR og mRNA_core TF_barn	1,08E-01	1/26 (0,038)

Top Tox Lists

Name	p-value	Ratio
Increases Cardiac Dysfunction	1,46E-02	2/34 (0,059)
Cardiac Necrosis/Cell Death	4,01E-02	4/237 (0,017)
Mitochondrial Dysfunction	5,38E-02	3/157 (0,019)
Long-term Renal Injury Pro-oxidative Response Panel (Rat)	6,83E-02	1/13 (0,077)
Cholesterol Biosynthesis	8,34E-02	1/16 (0,062)

(c) 2000-2013 Ingenuity Systems, Inc. All rights reserved.

INGENUITY® SYSTEMS

Top Tox Functions

Assays: Clinical Chemistry and Hematology

Name	p-value	# Molecules
Increased Levels of Blood Urea Nitrogen	7,34E-02 - 7,34E-02	1
Increased Levels of Creatinine	1,74E-01 - 1,74E-01	1

Cardiotoxicity

Name	p-value	# Molecules
Cardiac Dysfunction	1,27E-03 - 2,00E-01	2
Cardiac Necrosis/Cell Death	2,02E-02 - 8,15E-02	4
Cardiac Transformation	2,15E-02 - 2,15E-02	1
Cardiac Fibrosis	3,74E-02 - 3,74E-02	1
Cardiac Infarction	2,49E-01 - 2,49E-01	2

Hepatotoxicity

Name	p-value	# Molecules
Liver Inflammation/Hepatitis	6,83E-02 - 1,84E-01	3
Hepatocellular Carcinoma	2,61E-01 - 2,71E-01	4
Liver Hyperplasia/Hyperproliferation	2,61E-01 - 2,97E-01	5
Liver Cholestasis	2,75E-01 - 2,75E-01	1
Liver Fibrosis	4,57E-01 - 4,57E-01	1

Nephrotoxicity

Name	p-value	# Molecules
Renal Destruction	5,42E-03 - 5,42E-03	1
Renal Damage	9,07E-02 - 9,07E-02	2
Renal Tubule Injury	9,07E-02 - 9,07E-02	2

(c) 2000-2013 Ingenuity Systems, Inc. All rights reserved.

INGENUITY SYSTEMS

Renal Necrosis/Cell Death	1,62E-01 - 2,09E-01	3
Kidney Failure	2,30E-01 - 2,51E-01	1

(c) 2000-2013 Ingenuity Systems, Inc. All rights reserved.

Analysis Name: Adult_Immature B vs PreBII s_mRNA
Analysis Creation Date: 2013-06-09
Build version: 220217
Content version: 16542223 (Release Date: 2013-05-13)

Analysis settings

View
Reference set: Ingenuity Knowledge Base (Genes Only)
Relationship to include: Direct and Indirect
Includes Endogenous Chemicals
Optional Analyses: My Pathways My List

Filter Summary:
Consider only molecules and/or relationships where
(species = Rat OR Human OR Mouse) AND
(confidence = Experimentally Observed OR High (predicted))
Cutoff:

Top Networks

ID	Associated Network Functions	Score
1	Cell Cycle, Cellular Assembly and Organization, DNA Replication, Recombination, and Repair	55
2	Cell Cycle, Cellular Assembly and Organization, DNA Replication, Recombination, and Repair	52

(c) 2000-2013 Ingenuity Systems, Inc. All rights reserved.

3	Cell Death and Survival, Biliary Hyperplasia, Cardiovascular System Development and Function	42
4	Lipid Metabolism, Small Molecule Biochemistry, Cell Cycle	36
5	Hematological System Development and Function, Humoral Immune Response, Tissue Morphology	33

(c) 2000-2013 Ingenuity Systems, Inc. All rights reserved.

Top Bio Functions

Diseases and Disorders

Name	p-value	# Molecules
Cancer	2,79E-13 - 6,29E-03	229
Gastrointestinal Disease	2,79E-13 - 4,64E-03	104
Hematological Disease	5,17E-09 - 6,06E-03	65
Reproductive System Disease	4,45E-08 - 2,21E-03	52
Immunological Disease	7,93E-08 - 6,19E-03	72

Molecular and Cellular Functions

Name	p-value	# Molecules
Cell Cycle	2,28E-18 - 7,06E-03	102
Cellular Assembly and Organization	2,28E-18 - 6,97E-03	67
DNA Replication, Recombination, and Repair	2,28E-18 - 6,77E-03	74
Cellular Growth and Proliferation	4,05E-12 - 6,54E-03	143
Cell Death and Survival	3,05E-10 - 6,48E-03	141

Physiological System Development and Function

Name	p-value	# Molecules
Hematological System Development and Function	2,97E-10 - 7,06E-03	85
Humoral Immune Response	2,97E-10 - 6,80E-03	48
Tissue Morphology	2,97E-10 - 7,06E-03	86
Reproductive System Development and Function	1,27E-07 - 2,10E-03	11
Hematopoiesis	2,08E-07 - 7,06E-03	58

(c) 2000-2013 Ingenuity Systems, Inc. All rights reserved.

INGENUITY® SYSTEMS

Top Canonical Pathways

Name	p-value	Ratio
Cell Cycle Control of Chromosomal Replication	1,63E-04	5/31 (0,161)
Cell Cycle: G2/M DNA Damage Checkpoint Regulation	1,7E-04	6/49 (0,122)
B Cell Development	3,14E-04	5/33 (0,152)
PI3K Signaling in B Lymphocytes	7,49E-04	9/133 (0,068)
B Cell Receptor Signaling	9,23E-04	10/165 (0,061)

Top Molecules

Fold Change up-regulated

Molecules	Exp. Value	Exp. Chart
SP140	↑17,629	
CYBB	↑10,168	
ANGPTL1	↑7,626	
BIRC3	↑7,528	
RAB30	↑7,498	
RALGPS2	↑7,419	
MS4A1	↑6,312	
BTLA	↑6,006	
CNN2	↑5,910	
IRF8	↑5,740	

Fold Change down-regulated

Molecules	Exp. Value	Exp. Chart
DNTT	↓-4,554	
TMSB15A	↓-3,868	

(c) 2000-2013 Ingenuity Systems, Inc. All rights reserved.

INGENUITY® SYSTEMS

GBP4	↓-3,805
P4HA2	↓-3,539
GNAQ	↓-3,486
PBK	↓-3,350
RAG2	↓-3,302
ERG	↓-3,300
PSMB6	↓-3,269
HIST1H3A (includes others)*	↓-3,261

Top Upstream Regulators

Upstream Regulator	p-value of overlap	Predicted Activation State
CDKN1A	8,07E-33	Activated
TP53	6,41E-25	Activated
E2F4	1,07E-22	
CCND1	2,50E-21	Inhibited
CSF2	6,58E-20	Inhibited

(c) 2000-2013 Ingenuity Systems, Inc. All rights reserved.

Top My Lists

Name	p-value	Ratio
PreBI vs PreBIIL-miR target filter_cell cycle_adul	6,04E-19	24/111 (0,216)
PreBI vs PreBII_miR target filter_cell cycle_ID2_c	3,12E-04	6/51 (0,118)
PreBI vs PreBII_miR and mRNA_network1_adults	3,14E-04	5/34 (0,147)
PreBI vs PreBIIL_miR_and mRNA_utvidet_adults	4,81E-04	7/60 (0,117)
miR-126 PreBI/PreBII large children	2,43E-02	2/15 (0,133)

Top My Pathways

Name	p-value	Ratio
PreBI vs PreBIIL_miRs and mRNA_voksne	4,81E-04	7/60 (0,117)
mir-126 PreBI/PreBII large children	2,43E-02	2/15 (0,133)
PreBI vs PreBII L_miR og mRNA_core TF_barn	5,94E-02	2/26 (0,077)

Top Tox Lists

Name	p-value	Ratio
Cell Cycle: G2/M DNA Damage Checkpoint Regulation	2,78E-04	6/48 (0,125)
Increases Liver Hyperplasia/Hyperproliferation	5,15E-03	5/59 (0,085)
Reversible Glomerulonephritis Biomarker Panel (Rat)	1,41E-02	3/27 (0,111)
Primary Glomerulonephritis Biomarker Panel (Human)	1,76E-02	2/11 (0,182)
Anti-Apoptosis	2,23E-02	3/32 (0,094)

(c) 2000-2013 Ingenuity Systems, Inc. All rights reserved.

INGENUITY® SYSTEMS

Top Tox Functions

Assays: Clinical Chemistry and Hematology

Name	p-value	# Molecules
Increased Levels of Bilirubin	3,76E-02 - 3,76E-02	1
Increased Levels of ALT	9,13E-02 - 9,13E-02	1
Increased Levels of AST	1,09E-01 - 1,09E-01	1
Increased Levels of Alkaline Phosphatase	3,77E-01 - 3,77E-01	2
Increased Levels of Creatinine	4,89E-01 - 4,89E-01	1

Cardiotoxicity

Name	p-value	# Molecules
Cardiac Regeneration	3,76E-02 - 3,76E-02	1
Cardiac Dysfunction	5,58E-02 - 5,44E-01	3
Cardiac Hyperplasia/Hyperproliferation	5,58E-02 - 5,58E-02	1
Cardiac Necrosis/Cell Death	5,58E-02 - 3,92E-01	7
Cardiac Hypertrophy	5,94E-02 - 5,74E-01	8

Hepatotoxicity

Name	p-value	# Molecules
Hepatocellular Carcinoma	1,35E-07 - 9,74E-02	28
Liver Hyperplasia/Hyperproliferation	1,35E-07 - 9,74E-02	31
Liver Inflammation/Hepatitis	2,14E-03 - 5,86E-01	13
Biliary Hyperplasia	3,45E-03 - 3,45E-03	2
Liver Damage	4,64E-03 - 3,56E-01	10

Nephrotoxicity

(c) 2000-2013 Ingenuity Systems, Inc. All rights reserved.

Name	p-value	# Molecules
Renal Inflammation	1,01E-02 - 3,56E-01	12
Renal Nephritis	1,01E-02 - 3,56E-01	12
Renal Necrosis/Cell Death	1,11E-02 - 2,78E-01	15
Glomerular Injury	1,90E-02 - 5,78E-01	3
Renal Destruction	1,90E-02 - 1,90E-02	1

(c) 2000-2013 Ingenuity Systems, Inc. All rights reserved.

INGENUITY SYSTEMS
