# Supplementary material for: Transcriptional Profiling of mRNAs and microRNAs in Human Bone Marrow Precursor B Cells Identifies Subset- and Age-Specific Variations
Source: PLoS One. 2013 Jul 30;8(7):e70721. doi: 10.1371/journal.pone.0070721 (PMC3728296; doi:10.1371/journal.pone.0070721)
Supplement: Table S7 — (PDF) [file pone.0070721.s011.pdf]

IPA® Interactive pathway analysis of complex 'omics data

INGENUITY® SYSTEMS

Analysis Name: Child ProB vs Adult ProB
Analysis Creation Date: 2013-06-09
Build version: 220217
Content version: 16542223 (Release Date: 2013-05-13)

Analysis settings

View

Reference set: Ingenuity Knowledge Base (Genes Only)

Relationship to include: Direct and Indirect

Includes Endogenous Chemicals

Optional Analyses: My Pathways My List

Filter Summary:

Consider only molecules and/or relationships where

(species = Rat OR Human OR Mouse) AND

(confidence = Experimentally Observed OR High (predicted))

Cutoff:

Top Networks

ID	Associated Network Functions	Score
1	Molecular Transport, Hematological Disease, Metabolic Disease	33
2	Cell Cycle, Connective Tissue Disorders, Dental Disease	30

(c) 2000-2013 Ingenuity Systems, Inc. All rights reserved.

3	Cancer, Cellular Movement, Embryonic Development	23
4	Cellular Assembly and Organization, Neurological Disease, Free Radical Scavenging	16
5	Cell-To-Cell Signaling and Interaction, Cellular Movement, Molecular Transport	14

(c) 2000-2013 Ingenuity Systems, Inc. All rights reserved.

Top Bio Functions

Diseases and Disorders

Name	p-value	# Molecules
Gastrointestinal Disease	1,52E-06 - 6,52E-03	22
Immunological Disease	1,52E-06 - 4,27E-03	8
Ophthalmic Disease	1,52E-06 - 6,52E-03	7
Cancer	2,29E-06 - 7,55E-03	41
Dermatological Diseases and Conditions	2,29E-06 - 4,27E-03	17

Molecular and Cellular Functions

Name	p-value	# Molecules
Molecular Transport	3,64E-07 - 7,58E-03	15
Cellular Movement	3,67E-06 - 7,18E-03	20
Cellular Development	8,47E-06 - 7,58E-03	22
Cellular Growth and Proliferation	8,47E-06 - 7,58E-03	28
Cell-To-Cell Signaling and Interaction	9,23E-06 - 7,08E-03	23

Physiological System Development and Function

Name	p-value	# Molecules
Tissue Development	9,23E-06 - 7,58E-03	19
Tumor Morphology	9,45E-06 - 6,52E-03	10
Hematological System Development and Function	1,05E-05 - 7,18E-03	18
Hematopoiesis	1,05E-05 - 6,52E-03	7
Humoral Immune Response	1,05E-05 - 6,52E-03	3

(c) 2000-2013 Ingenuity Systems, Inc. All rights reserved.

Top Canonical Pathways

Name	p-value	Ratio
Acute Phase Response Signaling	2,25E-03	4/174 (0,023)
Agranulocyte Adhesion and Diapedesis	3,05E-03	4/186 (0,022)
Role of Macrophages, Fibroblasts and Endothelial Cells in Rheumatoid Arthritis	3,14E-03	5/329 (0,015)
Leukocyte Extravasation Signaling	3,97E-03	4/201 (0,02)
4-aminobutyrate Degradation I	9,77E-03	1/3 (0,333)

Top Molecules

Fold Change up-regulated

Molecules	Exp. Value	Exp. Chart
IGF2BP3	↑7,400	
NDUFAF2	↑5,043	
FOXD4L3/FOXD4L6	↑3,303	
ANKRD36B	↑2,948	
ZNF417/ZNF587	↑2,769	
ARL17A	↑2,531	
ENPP2	↑2,326	
FXYD6	↑2,306	
RBMS2	↑2,249	
PRSS3	↑2,247	

Fold Change down-regulated

Molecules	Exp. Value	Exp. Chart
HBB	↓-7,552	
CXCL12	↓-4,790	
ANXA1	↓-4,311	

(c) 2000-2013 Ingenuity Systems, Inc. All rights reserved.

RGS1	↓-3,989
IGK	↓-3,843
FAM127A	↓-3,837
ZNF208/ZNF729	↓-3,753
GJA1	↓-3,561
CDH2	↓-3,357
RGS2	↓-3,324

Top Upstream Regulators

Upstream Regulator	p-value of overlap	Predicted Activation State
EDN1	7,36E-08	
TGFB1	1,80E-07	
TAZ	1,08E-06	
TNF	2,02E-06	
STAT3	2,96E-06	

(c) 2000-2013 Ingenuity Systems, Inc. All rights reserved.

INGENUITY SYSTEMS

Top My Lists

Name	p-value	Ratio
PreBI vs PreBIIL-miR target filter_cell cycle_adul	4,72E-04	4/111 (0,036)
PreBI vs PreBII_miR target filter_cell cycle_ID2_c	1,12E-02	2/51 (0,039)
PreBI vs PreBII L barn_miR_utvidet mRNA_Core TFs	3,54E-02	1/16 (0,062)
PreBI vs PreBII_miR and mRNA_network1_adults	9,95E-02	1/34 (0,029)
PreBI vs PreBIIL_miR_and mRNA_utvidet_adults	1,59E-01	1/60 (0,017)

Top My Pathways

Name	p-value	Ratio
PreBI vs PreBII L_miR og mRNA_core TF_barn	6,64E-02	1/26 (0,038)
PreBI vs PreBIIL_miRs and mRNA_voksne	1,59E-01	1/60 (0,017)

Top Tox Lists

Name	p-value	Ratio
Genes associated with Chronic Allograft Nephropathy (Human)	2,11E-03	2/21 (0,095)
Acute Renal Failure Panel (Rat)	1,75E-02	2/62 (0,032)
Renal Inorganic Phosphate Homeostasis (Mouse)	1,94E-02	1/6 (0,167)
Negative Acute Phase Response Proteins	2,58E-02	1/8 (0,125)
Liver Proliferation	2,9E-02	3/204 (0,015)

(c) 2000-2013 Ingenuity Systems, Inc. All rights reserved.

INGENUITY® SYSTEMS

Top Tox Functions

Assays: Clinical Chemistry and Hematology

Name	p-value	# Molecules
Increased Levels of Red Blood Cells	2,70E-01 - 2,70E-01	1
Increased Levels of Hematocrit	2,73E-01 - 2,73E-01	1

Cardiotoxicity

Name	p-value	# Molecules
Cardiac Proliferation	3,27E-03 - 2,13E-01	1
Cardiac Dysfunction	9,77E-03 - 1,62E-02	1
Cardiac Inflammation	9,77E-03 - 9,77E-03	1
Cardiac Fibrosis	2,26E-02 - 3,14E-01	2
Cardiac Stenosis	2,26E-02 - 2,26E-02	1

Hepatotoxicity

Name	p-value	# Molecules
Liver Fibrosis	3,22E-04 - 1,62E-02	4
Liver Proliferation	3,22E-04 - 4,48E-02	3
Hepatocellular Carcinoma	1,25E-02 - 7,05E-02	4
Liver Hyperplasia/Hyperproliferation	1,25E-02 - 7,05E-02	6
Liver Cholestasis	1,30E-02 - 1,94E-02	1

Nephrotoxicity

Name	p-value	# Molecules
Glomerular Injury	3,27E-03 - 7,25E-02	2
Renal Hypertrophy	3,22E-02 - 7,25E-02	2
Renal Damage	3,71E-02 - 8,76E-02	3

(c) 2000-2013 Ingenuity Systems, Inc. All rights reserved.

INGENUITY® SYSTEMS

Renal Tubule Injury	3,71E-02 - 8,76E-02	3
Renal Inflammation	4,79E-02 - 2,70E-01	1

(c) 2000-2013 Ingenuity Systems, Inc. All rights reserved.

Analysis Name: Child PreBI vs Adult PreBI
Analysis Creation Date: 2013-06-09
Build version: 220217
Content version: 16542223 (Release Date: 2013-05-13)

Analysis settings

View

Reference set: Ingenuity Knowledge Base (Genes Only)

Relationship to include: Direct and Indirect

Includes Endogenous Chemicals

Optional Analyses: My Pathways My List

Filter Summary:

Consider only molecules and/or relationships where

(species = Rat OR Human OR Mouse) AND

(confidence = Experimentally Observed OR High (predicted))

Cutoff:

Top Networks

ID	Associated Network Functions	Score
1	Cardiovascular System Development and Function, Cell-To-Cell Signaling and Interaction, Tissue Development	36
2	Cell Death and Survival, Cancer, Endocrine System Disorders	33

(c) 2000-2013 Ingenuity Systems, Inc. All rights reserved.

3	Cellular Function and Maintenance, Post-Translational Modification, Protein Folding	30
4	Cellular Movement, Hematological System Development and Function, Humoral Immune Response	14
5	Cardiovascular Disease, Developmental Disorder, Hereditary Disorder	14

(c) 2000-2013 Ingenuity Systems, Inc. All rights reserved.

Top Bio Functions

Diseases and Disorders

Name	p-value	# Molecules
Infectious Disease	3,28E-04 - 2,40E-02	20
Cancer	4,00E-04 - 2,99E-02	12
Respiratory Disease	4,00E-04 - 2,70E-02	3
Connective Tissue Disorders	5,08E-04 - 2,10E-02	12
Immunological Disease	5,08E-04 - 2,70E-02	13

Molecular and Cellular Functions

Name	p-value	# Molecules
Cell-To-Cell Signaling and Interaction	9,10E-05 - 2,99E-02	11
Cellular Movement	1,34E-04 - 2,99E-02	13
Cellular Development	1,88E-04 - 2,99E-02	9
Cellular Function and Maintenance	1,88E-04 - 2,99E-02	11
Cellular Growth and Proliferation	1,88E-04 - 2,99E-02	9

Physiological System Development and Function

Name	p-value	# Molecules
Cardiovascular System Development and Function	9,10E-05 - 2,99E-02	8
Tissue Development	9,10E-05 - 2,99E-02	12
Connective Tissue Development and Function	1,34E-04 - 2,70E-02	7
Cell-mediated Immune Response	1,88E-04 - 2,70E-02	2
Hematological System Development and Function	1,88E-04 - 2,99E-02	9

(c) 2000-2013 Ingenuity Systems, Inc. All rights reserved.

Top Canonical Pathways

Name	p-value	Ratio
Altered T Cell and B Cell Signaling in Rheumatoid Arthritis	2,26E-03	3/91 (0,033)
Systemic Lupus Erythematosus Signaling	4,63E-03	4/246 (0,016)
Glucocorticoid Receptor Signaling	8,02E-03	4/280 (0,014)
Rapoport-Luebering Glycolytic Shunt	1,21E-02	1/4 (0,25)
TREM1 Signaling	1,34E-02	2/61 (0,033)

Top Molecules

Fold Change up-regulated

Molecules	Exp. Value	Exp. Chart
IGF2BP3	↑7,895	
CHORDC1	↑6,259	
CPED1	↑4,485	
ARL17A	↑3,444	
RPL35	↑3,168	
UTY	↑3,034	
FOXD4L3/FOXD4L6	↑2,904	
HSPA1A/HSPA1B	↑2,800	
HNRNPK	↑2,670	
DYNLL1	↑2,644	

Fold Change down-regulated

Molecules	Exp. Value	Exp. Chart
IGK	↓-4,682	
IFI44L	↓-3,772	
ZNF417/ZNF587	↓-3,608	

(c) 2000-2013 Ingenuity Systems, Inc. All rights reserved.

PPBP	↓-3,568
HBB	↓-3,462
SLC44A5	↓-2,845
CDH2	↓-2,739
IFIT3	↓-2,728
SLFN5	↓-2,719
RBMS2	↓-2,691

Top Upstream Regulators

Upstream Regulator	p-value of overlap	Predicted Activation State
HNRNPU	1,27E-05	
RET	1,48E-05	
miR-3684 (miRNAs w/seed UAGACCU)	2,95E-05	
HSF1	1,24E-04	
RPA1	1,41E-04	

(c) 2000-2013 Ingenuity Systems, Inc. All rights reserved.

Top My Lists

Name	p-value	Ratio
PreBI vs PreBII_miR target filter_cell cycle_ID2_c	4,39E-04	3/51 (0,059)
PreBI vs PreBIIL-miR target filter_cell cycle_adul	4,47E-02	2/111 (0,018)
PreBI vs PreBII_miR and mRNA_network1_adults	9,27E-02	1/34 (0,029)
PreBI vs PreBIIL_miR_and mRNA_adults	1,36E-01	1/63 (0,016)
PreBI vs PreBIIL_miR_and mRNA_utvidet_adults	1,49E-01	1/60 (0,017)

Top My Pathways

Name	p-value	Ratio
PreBI vs PreBIIL_miRs and mRNA_voksne	1,49E-01	1/60 (0,017)

Top Tox Lists

Name	p-value	Ratio
Reversible Glomerulonephritis Biomarker Panel (Rat)	3,02E-03	2/27 (0,074)
Acute Renal Failure Panel (Rat)	1,52E-02	2/62 (0,032)
FXR/RXR Activation	2,8E-02	2/86 (0,023)
Increases Damage of Mitochondria	3,29E-02	1/11 (0,091)
Mechanism of Gene Regulation by Peroxisome Proliferators via PPARα	3,37E-02	2/95 (0,021)

(c) 2000-2013 Ingenuity Systems, Inc. All rights reserved.

INGENUITY® SYSTEMS

Top Tox Functions

Assays: Clinical Chemistry and Hematology

Name	p-value	# Molecules
Increased Levels of Albumin	3,03E-03 - 3,03E-03	1
Decreased Levels of Albumin	1,21E-02 - 1,21E-02	1
Increased Levels of ALT	1,81E-02 - 1,81E-02	1
Increased Levels of LDH	3,87E-02 - 3,87E-02	1
Increased Levels of Alkaline Phosphatase	1,89E-01 - 1,89E-01	1

Cardiotoxicity

Name	p-value	# Molecules
Cardiac Inflammation	9,07E-03 - 1,12E-01	2
Cardiac Fibrosis	2,10E-02 - 2,10E-02	1
Cardiac Damage	1,17E-01 - 1,17E-01	1
Cardiac Arrythmia	2,51E-01 - 2,51E-01	1
Cardiac Hypertrophy	3,13E-01 - 5,23E-01	2

Hepatotoxicity

Name	p-value	# Molecules
Liver Damage	6,18E-02 - 6,18E-02	1
Hepatocellular Carcinoma	1,46E-01 - 1,76E-01	3
Liver Hyperplasia/Hyperproliferation	1,46E-01 - 1,76E-01	3
Liver Cirrhosis	2,49E-01 - 2,49E-01	1
Liver Proliferation	3,71E-01 - 3,71E-01	1

Nephrotoxicity

(c) 2000-2013 Ingenuity Systems, Inc. All rights reserved.

Name	p-value	# Molecules
Renal Necrosis/Cell Death	6,75E-02 - 1,82E-01	3
Nephrosis	1,57E-01 - 1,57E-01	1
Renal Proliferation	1,72E-01 - 1,72E-01	1
Renal Damage	2,47E-01 - 2,47E-01	1
Renal Tubule Injury	2,47E-01 - 2,47E-01	1

(c) 2000-2013 Ingenuity Systems, Inc. All rights reserved.

IPA® Interactive pathway analysis of complex 'omics data

INGENUITY® SYSTEMS

Analysis Name: Child PreBII L vs Adult PreBII L
Analysis Creation Date: 2013-06-09
Build version: 220217
Content version: 16542223 (Release Date: 2013-05-13)

Analysis settings

View

Reference set: Ingenuity Knowledge Base (Genes Only)

Relationship to include: Direct and Indirect

Includes Endogenous Chemicals

Optional Analyses: My Pathways My List

Filter Summary:

Consider only molecules and/or relationships where

(species = Rat OR Human OR Mouse) AND

(confidence = Experimentally Observed OR High (predicted))

Cutoff:

Top Networks

ID	Associated Network Functions	Score
1	Cancer, Hematological System Development and Function, Humoral Immune Response	34
2	Carbohydrate Metabolism, Lipid Metabolism, Post-Translational Modification	29

(c) 2000-2013 Ingenuity Systems, Inc. All rights reserved.

INGENUITY® SYSTEMS

3	Endocrine System Disorders, Reproductive System Disease, Cancer	27
4	Developmental Disorder, Ophthalmic Disease, Protein Synthesis	20
5	Lymphoid Tissue Structure and Development, Tissue Morphology, Cellular Development	17

(c) 2000-2013 Ingenuity Systems, Inc. All rights reserved.

INGENUITY® SYSTEMS

Top Bio Functions

Diseases and Disorders

Name	p-value	# Molecules
Cancer	5,27E-04 - 4,09E-02	19
Reproductive System Disease	5,27E-04 - 2,31E-02	9
Neurological Disease	6,14E-04 - 3,73E-02	9
Inflammatory Response	3,40E-03 - 4,09E-02	4
Cardiovascular Disease	3,79E-03 - 3,36E-02	3

Molecular and Cellular Functions

Name	p-value	# Molecules
Cell Cycle	1,86E-03 - 4,86E-02	6
Cell Death and Survival	1,86E-03 - 4,82E-02	11
Protein Synthesis	2,32E-03 - 3,36E-02	10
Cell-To-Cell Signaling and Interaction	3,40E-03 - 4,46E-02	7
Carbohydrate Metabolism	3,79E-03 - 2,62E-02	1

Physiological System Development and Function

Name	p-value	# Molecules
Hematological System Development and Function	1,86E-03 - 4,82E-02	7
Humoral Immune Response	1,86E-03 - 3,73E-02	5
Tissue Morphology	1,86E-03 - 4,82E-02	6
Lymphoid Tissue Structure and Development	3,23E-03 - 3,73E-02	6
Embryonic Development	3,74E-03 - 4,46E-02	7

(c) 2000-2013 Ingenuity Systems, Inc. All rights reserved.

Top Canonical Pathways

Name	p-value	Ratio
EIF2 Signaling	5,12E-03	4/192 (0,021)
S-methyl-5-thio-α-D-ribose 1-phosphate Degradation	7,57E-03	1/2 (0,5)
Serine Biosynthesis	1,51E-02	1/4 (0,25)
Regulation of eIF4 and p70S6K Signaling	1,83E-02	3/164 (0,018)
Superpathway of Serine and Glycine Biosynthesis I	2,25E-02	1/6 (0,167)

Top Molecules

Fold Change up-regulated

Molecules	Exp. Value	Exp. Chart
IGF2BP3	↑7,349	
RABL2B	↑3,473	
HNRNPK	↑2,918	
FOXD4L3/FOXD4L6	↑2,793	
PCDH11X/PCDH11Y	↑2,771	
AMICA1	↑2,746	
LAMP5	↑2,719	
ZFY	↑2,695	
ENPP2	↑2,521	
LRRC37A3 (includes others)	↑2,388	

Fold Change down-regulated

Molecules	Exp. Value	Exp. Chart
ID2	↓-9,357	
HBB	↓-4,052	
ACBD7	↓-3,262	

(c) 2000-2013 Ingenuity Systems, Inc. All rights reserved.

APIP	↓-2,993
RBMS2	↓-2,853
EIF1AX	↓-2,834
PIGH	↓-2,668
SMU1	↓-2,659
RGS2	↓-2,644
GTSF1	↓-2,503

Top Upstream Regulators

Upstream Regulator	p-value of overlap	Predicted Activation State
miR-881-3p (and other miRNAs w/seed ACUGUGU)	1,40E-04	
TET2	4,39E-04	
ATF4	6,98E-04	
MTA2	1,03E-03	
BRCA1	1,51E-03	

(c) 2000-2013 Ingenuity Systems, Inc. All rights reserved.

Top My Lists

Name	p-value	Ratio
PreBI vs PreBIIL-miR target filter_cell cycle_adul	6,3E-05	5/111 (0,045)
PreBI vs PreBII_miR and mRNA_network1_adults	6,52E-03	2/34 (0,059)
PreBI vs PreBIIL_miR_and mRNA_utvidet_adults	1,72E-02	2/60 (0,033)
PreBI vs PreBII L barn_miR_utvidet mRNA_Core TFs	4,09E-02	1/16 (0,062)
PreBI vs PreBII_miR target filter_cell cycle_ID2_c	1,7E-01	1/51 (0,02)

Top My Pathways

Name	p-value	Ratio
PreBI vs PreBIIL_miRs and mRNA_voksne	1,72E-02	2/60 (0,033)
PreBI vs PreBII L_miR og mRNA_core TF_barn	7,67E-02	1/26 (0,038)

Top Tox Lists

Name	p-value	Ratio
Reversible Glomerulonephritis Biomarker Panel (Rat)	9,75E-02	1/27 (0,037)
Pro-Apoptosis	1,48E-01	1/42 (0,024)
Increases Transmembrane Potential of Mitochondria and Mitochondrial Membrane	1,73E-01	1/50 (0,02)
Cardiac Necrosis/Cell Death	2,27E-01	2/237 (0,008)
Hypoxia-Inducible Factor Signaling	2,34E-01	1/70 (0,014)

(c) 2000-2013 Ingenuity Systems, Inc. All rights reserved.

Top Tox Functions

Assays: Clinical Chemistry and Hematology

Name	p-value	# Molecules
Increased Levels of Red Blood Cells	3,06E-01 - 3,06E-01	1
Increased Levels of Hematocrit	3,09E-01 - 3,09E-01	1

Cardiotoxicity

Name	p-value	# Molecules
Cardiac Hypertrophy	1,15E-01 - 1,15E-01	1
Cardiac Necrosis/Cell Death	1,58E-01 - 1,58E-01	2
Cardiac Arrythmia	3,04E-01 - 3,04E-01	1
Cardiac Arteriopathy	3,08E-01 - 3,08E-01	2

Hepatotoxicity

Name	p-value	# Molecules
Liver Necrosis/Cell Death	1,08E-01 - 1,08E-01	1
Liver Inflammation/Hepatitis	1,61E-01 - 1,61E-01	1
Liver Hyperplasia/Hyperproliferation	2,48E-01 - 5,45E-01	4
Liver Cirrhosis	3,01E-01 - 3,01E-01	1
Hepatocellular Carcinoma	5,45E-01 - 5,45E-01	2

Nephrotoxicity

Name	p-value	# Molecules
Renal Proliferation	1,13E-02 - 1,13E-02	1
Renal Necrosis/Cell Death	3,73E-02 - 6,25E-01	1
Glomerular Injury	7,67E-02 - 7,67E-02	1
Renal Hydronephrosis	2,87E-01 - 2,87E-01	1

(c) 2000-2013 Ingenuity Systems, Inc. All rights reserved.

Kidney Failure	3,77E-01 - 3,77E-01	1

(c) 2000-2013 Ingenuity Systems, Inc. All rights reserved.

Analysis Name: Child PreBII s vs Adult PreBII s
Analysis Creation Date: 2013-06-09
Build version: 220217
Content version: 16542223 (Release Date: 2013-05-13)

Analysis settings

View
Reference set: Ingenuity Knowledge Base (Genes Only)
Relationship to include: Direct and Indirect
Includes Endogenous Chemicals
Optional Analyses: My Pathways My List

Filter Summary:
Consider only molecules and/or relationships where
(species = Rat OR Human OR Mouse) AND
(confidence = Experimentally Observed OR High (predicted))
Cutoff:

Top Networks

ID	Associated Network Functions	Score
1	Cellular Movement, Connective Tissue Development and Function, Cardiovascular Disease	36
2	Cell Signaling, Nucleic Acid Metabolism, Small Molecule Biochemistry	15

(c) 2000-2013 Ingenuity Systems, Inc. All rights reserved.

3 Hereditary Disorder, Neurological Disease, Psychological Disorders 2

(c) 2000-2013 Ingenuity Systems, Inc. All rights reserved.

Top Bio Functions

Diseases and Disorders

Name	p-value	# Molecules
Neurological Disease	2,57E-05 - 9,92E-04	5
Cancer	9,92E-04 - 4,47E-02	7
Cardiovascular Disease	9,92E-04 - 3,13E-02	3
Connective Tissue Disorders	9,92E-04 - 1,77E-02	2
Developmental Disorder	9,92E-04 - 3,13E-02	4

Molecular and Cellular Functions

Name	p-value	# Molecules
RNA Post-Transcriptional Modification	2,97E-04 - 3,51E-02	3
Cellular Development	5,72E-04 - 4,85E-02	6
Antigen Presentation	9,92E-04 - 9,92E-04	1
Cell Cycle	9,92E-04 - 7,91E-03	1
Cell Death and Survival	9,92E-04 - 4,76E-02	4

Physiological System Development and Function

Name	p-value	# Molecules
Cardiovascular System Development and Function	9,92E-04 - 4,85E-02	4
Cell-mediated Immune Response	9,92E-04 - 4,66E-02	2
Digestive System Development and Function	9,92E-04 - 1,77E-02	1
Embryonic Development	9,92E-04 - 4,37E-02	5
Hematological System Development and Function	9,92E-04 - 4,85E-02	4

(c) 2000-2013 Ingenuity Systems, Inc. All rights reserved.

Top Canonical Pathways

Name	p-value	Ratio
Interferon Signaling	3,32E-02	1/34 (0,029)
Neurotrophin/TRK Signaling	6,54E-02	1/71 (0,014)
Ephrin B Signaling	7,09E-02	1/80 (0,012)
Mouse Embryonic Stem Cell Pluripotency	9,02E-02	1/99 (0,01)
SAPK/JNK Signaling	9,02E-02	1/102 (0,01)

Top Molecules

Fold Change up-regulated

Molecules	Exp. Value	Exp. Chart
IGF2BP3	↑4,676	
FAM127A	↑3,450	
CDR1	↑3,324	
CBWD3/CBWD6	↑3,193	
C21orf62	↑3,009	
LAMP5	↑2,847	
AMICA1	↑2,519	
HNRNPK	↑2,351	
TMSB4Y	↑2,346	
ZFY	↑2,320	

Fold Change down-regulated

Molecules	Exp. Value	Exp. Chart
ID2	↓-2,879	
IFIT3	↓-2,606	
GIMAP4	↓-2,588	

(c) 2000-2013 Ingenuity Systems, Inc. All rights reserved.

INGENUITY® SYSTEMS

LOC100134868	↓-2,512
HBB	↓-2,504
RGS2	↓-2,252
MROH9	↓-2,148
CD96	↓-2,066
SPRY1	↓-2,023
C11orf74	↓-2,017

Top Upstream Regulators

Upstream Regulator	p-value of overlap	Predicted Activation State
MTA2	8,99E-05	
CHD4	1,38E-04	
COMMD3-BMI1	2,41E-04	
SUPT16H	2,65E-04	
SSRP1	4,96E-04	

(c) 2000-2013 Ingenuity Systems, Inc. All rights reserved.

Top My Lists

Name	p-value	Ratio
PreBI vs PreBII L barn_miR_utvidet mRNA_Core TFs	1,09E-02	1/16 (0,062)
PreBI vs PreBII_miR and mRNA_network1_adults	3,13E-02	1/34 (0,029)
PreBI vs PreBII_miR target filter_cell cycle_ID2_c	4,75E-02	1/51 (0,02)
PreBI vs PreBIIL_miR_and mRNA_utvidet_adults	5,13E-02	1/60 (0,017)
PreBI vs PreBIIL-miR target filter_cell cycle_adul	1,05E-01	1/111 (0,009)

Top My Pathways

Name	p-value	Ratio
PreBI vs PreBII L_miR og mRNA_core TF_barn	2,06E-02	1/26 (0,038)
PreBI vs PreBIIL_miRs and mRNA_voksne	5,13E-02	1/60 (0,017)

Top Tox Lists

Name	p-value	Ratio
Reversible Glomerulonephritis Biomarker Panel (Rat)	2,65E-02	1/27 (0,037)
Cardiac Hypertrophy	2,92E-01	1/344 (0,003)

(c) 2000-2013 Ingenuity Systems, Inc. All rights reserved.

Top Tox Functions

Assays: Clinical Chemistry and Hematology

Name	p-value	# Molecules
Increased Levels of Red Blood Cells	9,11E-02 - 9,11E-02	1
Increased Levels of Hematocrit	9,20E-02 - 9,20E-02	1

Cardiotoxicity

Name	p-value	# Molecules
Cardiac Hypertrophy	3,13E-02 - 3,13E-02	1

Hepatotoxicity

Name	p-value	# Molecules
Liver Cirrhosis	8,93E-02 - 8,93E-02	1

Nephrotoxicity

Name	p-value	# Molecules
Renal Hydronephrosis	8,47E-02 - 8,47E-02	1

(c) 2000-2013 Ingenuity Systems, Inc. All rights reserved.

Analysis Name: Child Immature B vs Adult Immature B
Analysis Creation Date: 2013-06-09
Build version: 220217
Content version: 16542223 (Release Date: 2013-05-13)

Analysis settings

View
Reference set: Ingenuity Knowledge Base (Genes Only)
Relationship to include: Direct and Indirect
Includes Endogenous Chemicals
Optional Analyses: My Pathways My List

Filter Summary:
Consider only molecules and/or relationships where
(species = Rat OR Human OR Mouse) AND
(confidence = Experimentally Observed OR High (predicted))
Cutoff:

Top Networks

ID	Associated Network Functions	Score
1	Cell Cycle, Cellular Assembly and Organization, DNA Replication, Recombination, and Repair	62
2	Nucleic Acid Metabolism, Small Molecule Biochemistry, Cancer	47

(c) 2000-2013 Ingenuity Systems, Inc. All rights reserved.

3	Cell Death and Survival, Embryonic Development, Protein Degradation	30
4	Embryonic Development, Organ Development, Organismal Development	27
5	Cellular Function and Maintenance, Hematological System Development and Function, Humoral Immune Response	25

(c) 2000-2013 Ingenuity Systems, Inc. All rights reserved.

Top Bio Functions

Diseases and Disorders

Name	p-value	# Molecules
Cancer	3,62E-09 - 1,35E-02	75
Reproductive System Disease	3,62E-09 - 8,93E-03	30
Gastrointestinal Disease	1,97E-08 - 1,10E-02	41
Hepatic System Disease	2,56E-06 - 1,10E-02	17
Skeletal and Muscular Disorders	6,69E-06 - 6,77E-03	13

Molecular and Cellular Functions

Name	p-value	# Molecules
Cell Cycle	5,65E-17 - 1,35E-02	47
Cellular Assembly and Organization	5,65E-17 - 1,35E-02	32
DNA Replication, Recombination, and Repair	5,65E-17 - 1,35E-02	44
Cell Death and Survival	1,59E-05 - 1,35E-02	33
Cellular Growth and Proliferation	3,05E-05 - 1,35E-02	50

Physiological System Development and Function

Name	p-value	# Molecules
Embryonic Development	1,36E-04 - 1,35E-02	27
Nervous System Development and Function	1,36E-04 - 8,45E-03	10
Hematological System Development and Function	1,92E-04 - 1,35E-02	20
Humoral Immune Response	1,92E-04 - 1,33E-02	8
Tissue Morphology	1,92E-04 - 1,35E-02	17

(c) 2000-2013 Ingenuity Systems, Inc. All rights reserved.

Top Canonical Pathways

Name	p-value	Ratio
Mitotic Roles of Polo-Like Kinase	6,01E-06	6/70 (0,086)
Cell Cycle: G2/M DNA Damage Checkpoint Regulation	1,14E-05	5/49 (0,102)
Role of CHK Proteins in Cell Cycle Checkpoint Control	5,2E-04	4/57 (0,07)
Role of BRCA1 in DNA Damage Response	7,25E-04	4/63 (0,063)
ATM Signaling	7,25E-04	4/62 (0,065)

Top Molecules

Fold Change up-regulated

Molecules	Exp. Value	Exp. Chart
IGF2BP3	↑7,947	
HGF	↑3,914	
CDR1	↑3,860	
FOXD4L3/FOXD4L6	↑3,540	
GPR174	↑3,424	
TAS2R4	↑3,094	
PCDH11X/PCDH11Y	↑2,966	
PLEK	↑2,929	
GRAPL	↑2,893	
LCE2C (includes others)	↑2,816	

Fold Change down-regulated

Molecules	Exp. Value	Exp. Chart
HBB	↓-6,710	
HIST1H2BB	↓-5,242	
RGS2	↓-3,762	

(c) 2000-2013 Ingenuity Systems, Inc. All rights reserved.

SPC25	↓-3,617
MROH9	↓-3,525
HIST2H2BE (includes others)	↓-3,349
MND1	↓-3,202
MTFR2	↓-3,189
DNTT	↓-3,131
MKI67	↓-3,108

Top Upstream Regulators

Upstream Regulator	p-value of overlap	Predicted Activation State
CDKN1A	6,47E-26	Activated
E2F4	7,32E-26	
CCND1	1,82E-21	Inhibited
TP53	1,13E-17	Activated
CDK4	2,22E-17	

(c) 2000-2013 Ingenuity Systems, Inc. All rights reserved.

Top My Lists

Name	p-value	Ratio
PreBI vs PreBIIL-miR target filter_cell cycle_adul	1,16E-11	12/111 (0,108)
PreBI vs PreBII_miR and mRNA_network1_adults	1,97E-02	2/34 (0,059)
PreBI vs PreBII_miR target filter_cell cycle_ID2_c	4,34E-02	2/51 (0,039)
PreBI vs PreBIIL_miR_and mRNA_utvidet_adults	5E-02	2/60 (0,033)
PreBI vs PreBII L barn_miR_utvidet mRNA_Core TFs	7,2E-02	1/16 (0,062)

Top My Pathways

Name	p-value	Ratio
PreBI vs PreBIIL_miRs and mRNA_voksne	5E-02	2/60 (0,033)
PreBI vs PreBII L_miR og mRNA_core TF_barn	1,33E-01	1/26 (0,038)

Top Tox Lists

Name	p-value	Ratio
Cell Cycle: G2/M DNA Damage Checkpoint Regulation	1,77E-05	5/48 (0,104)
Increases Liver Hyperplasia/Hyperproliferation	6,8E-04	4/59 (0,068)
Reversible Glomerulonephritis Biomarker Panel (Rat)	1,43E-02	2/27 (0,074)
Aryl Hydrocarbon Receptor Signaling	2,34E-02	4/160 (0,025)
Increases Transmembrane Potential of Mitochondria and Mitochondrial Membrane	4,5E-02	2/50 (0,04)

(c) 2000-2013 Ingenuity Systems, Inc. All rights reserved.

INGENUITY SYSTEMS

Top Tox Functions

Assays: Clinical Chemistry and Hematology

Name	p-value	# Molecules
Increased Levels of Red Blood Cells	2,74E-02 - 2,74E-02	3
Increased Levels of Hematocrit	1,40E-01 - 1,40E-01	2

Cardiotoxicity

Name	p-value	# Molecules
Cardiac Regeneration	1,35E-02 - 1,35E-02	1
Cardiac Hyperplasia/Hyperproliferation	2,02E-02 - 2,02E-02	1
Cardiac Output	2,68E-02 - 2,68E-02	1
Cardiac Inflammation	1,03E-01 - 1,03E-01	1
Congenital Heart Anomaly	1,21E-01 - 2,74E-01	1

Hepatotoxicity

Name	p-value	# Molecules
Liver Hyperplasia/Hyperproliferation	2,56E-06 - 3,26E-01	17
Hepatocellular Carcinoma	2,11E-05 - 3,26E-01	13
Liver Necrosis/Cell Death	1,07E-03 - 3,34E-02	3
Liver Cholestasis	2,02E-02 - 2,02E-02	1
Liver Failure	2,68E-02 - 9,07E-02	2

Nephrotoxicity

Name	p-value	# Molecules
Renal Inflammation	1,35E-02 - 4,40E-01	3
Renal Nephritis	1,35E-02 - 4,40E-01	3
Renal Regeneration	1,35E-02 - 1,35E-02	1

(c) 2000-2013 Ingenuity Systems, Inc. All rights reserved.

INGENUITY® SYSTEMS

Renal Necrosis/Cell Death	6,57E-02 - 5,19E-01	3
Renal Damage	8,35E-02 - 9,69E-02	2

(c) 2000-2013 Ingenuity Systems, Inc. All rights reserved.
