# Supplementary material for: Transcriptional Profiling of mRNAs and microRNAs in Human Bone Marrow Precursor B Cells Identifies Subset- and Age-Specific Variations
Source: PLoS One. 2013 Jul 30;8(7):e70721. doi: 10.1371/journal.pone.0070721 (PMC3728296; doi:10.1371/journal.pone.0070721)
Supplement: Table S8 — (PDF) [file pone.0070721.s012.pdf]

Transcripts up- or down-regulated in all subsets in children versus adults
(2-way ANOVA, FDR 1%)

Transcript	Gene	p-value	Fold change (higher in children)	Pathway (functional analysis)
3041409	IGF2BP3	9,99E-22	7,18	
3876084	C20orf103	1,13E-05	1,91	
3508330	HSPH1	2,11E-06	1,69	Aldosterone signaling in epithelial cells, Protein ubiquitination pathway
2998333	C7orf36	6,65E-06	1,54	
3321512	PDE3B	4,17E-06	1,42	tRNA splicing, Leptin signaling in obesity, TR/RXR activation, Nitric oxide signaling in the cardiovascular system, Cellular effects of sildenafil, Insulin receptor signaling, Relaxin signaling, Cardiac β-adrenergic signaling, cAMP-mediated signaling, G-protein coupled receptor signaling, Protein kinase A signaling
3395416	HSPA8	3,88E-06	1,23	Aldosterone signaling in epithelial cells, Protein ubiquitination pathway, eNOS signaling, Clathrin-mediated endocytosis signaling, Huntington´s disease signaling, Glucocorticoid receptor signaling
			(higher in adults)	
3377044	SF1	2,64E-06	-1,11	
3442205	ZNF384	6,35E-06	-1,17	
3216931	C9orf156	1,59E-07	-1,39	
3737874	BAHCC1	6,73E-08	-1,57	
2635741	CD96	4,23E-06	-1,62	
3986230	CXorf57	4,94E-08	-1,85	
3802602	CDH2	6,50E-10	-1,95	Gα12/13 signaling, Epithelial adherens junction signaling, Germ cell-Sertoli cell junction signaling, Wnt/β-catenin signaling, Rho GDI signaling, Regulation of the epithelial-mesenchymal transition pathway, Signaling by Rho-family GTPases
3740479	PRPF8	1,35E-06	-2,17	
2536965	FLJ38379	2,64E-06	-2,28	
3916290	FLJ42200	1,35E-10	-2,81	

© 2000-2013 Ingenuity Systems, Inc. All rights reserved.
