# Supplementary material for: Transcriptional Profiling of mRNAs and microRNAs in Human Bone Marrow Precursor B Cells Identifies Subset- and Age-Specific Variations
Source: PLoS One. 2013 Jul 30;8(7):e70721. doi: 10.1371/journal.pone.0070721 (PMC3728296; doi:10.1371/journal.pone.0070721)
Supplement: Table S9 — (PDF) [file pone.0070721.s013.pdf]

A number of 17 microRNAs being at least once differentially expressed between the various maturation stages (FDR 10 %, p ≤ 3.6 x 10^{-3})

Column ID	p-value
hsa-miR-520g	0,00201
hsa-miR-137	0,00393
hsa-miR-511	0,00015
hsa-miR-657	0,00302
hsa-miR-145	0,00310
hsa-miR-149	0,00035
hsa-miR-200c	0,00016
hsa-miR-200c*	0,00223
hsa-miR-126	0,00005
hsa-miR-339-5p	0,00004
hsa-miR-642	0,00024
hsa-miR-483-5p	0,00131
hsa-miR-141	0,00259
hsa-miR-126*	0,00124
hsa-miR-30a*	0,00196
hsa-miR-25*	0,00093
hsa-miR-451	0,00368
