# Supplementary material for: Transcriptional Profiling of mRNAs and microRNAs in Human Bone Marrow Precursor B Cells Identifies Subset- and Age-Specific Variations
Source: PLoS One. 2013 Jul 30;8(7):e70721. doi: 10.1371/journal.pone.0070721 (PMC3728296; doi:10.1371/journal.pone.0070721)
Supplement: Table S10 — (PDF) [file pone.0070721.s014.pdf]

Differentially expressed microRNAs comparing children and adults at each differentiation step

ProB comparisons:

miR	p-value	fold change		
hsa-miR-657	0,0315	58,44		higher in children
hsa-miR-579	0,0326	56,10		
hsa-miR-454*	0,0356	49,06		
hsa-let-7a*	0,0453	13,54		
hsa-miR-100*	0,0300	7,50		

PreBI comparisons:

miR	p-value	fold change		
hsa-miR-589*	0,0007	149,59		higher in children
hsa-miR-149	0,0019	57,79		
hsa-miR-505	0,0454	41,94		
hsa-miR-361-3p	0,0262	21,19		
hsa-miR-339-5p	0,0421	17,79		
hsa-miR-501-3p	0,0102	17,76		
hsa-miR-500	0,0403	9,11		
hsa-let-7c	0,0217	0,43	-2,31	lower in children
hsa-miR-623	0,0476	0,23	-4,34	
hsa-miR-145	0,0184	0,13	-7,54	
hsa-miR-410	0,0278	0,10	-10,16	
hsa-miR-411	0,0307	0,09	-10,94	
hsa-miR-184	0,0313	0,09	-11,12	
hsa-miR-626	0,0150	0,08	-13,32	
hsa-miR-641	0,0161	0,07	-13,91	
hsa-miR-409-3p	0,0437	0,06	-15,42	
hsa-miR-194*	0,0288	0,05	-20,50	
hsa-miR-200c*	0,0374	0,04	-25,36	
hsa-miR-641	0,0409	0,04	-27,51	
hsa-miR-638	0,0417	0,02	-47,42	
hsa-miR-518d-3p	0,0183	0,02	-52,68	
hsa-miR-628-3p	0,0173	0,02	-55,01	
hsa-miR-133a	0,0075	0,01	-131,29	

PreBII large comparisons:

miR	p-value	fold change		
hsa-miR-33a*	0,0282	22,60		higher in children
hsa-miR-129-3p	0,0046	19,16		
hsa-miR-551a	0,0024	17,67		
hsa-miR-589	0,0007	17,57		
hsa-miR-20b*	0,0106	14,09		
hsa-miR-148b*	0,0150	6,22		
hsa-miR-190b	0,0249	6,20		
hsa-miR-628-3p	0,0428	0,31	-3,27	lower in children
hsa-miR-151-3p	0,0290	0,30	-3,36	
hsa-miR-768-3p	0,0097	0,30	-3,36	
hsa-miR-768-3p	0,0023	0,27	-3,74	
hsa-miR-584	0,0082	0,25	-3,98	
hsa-miR-485-3p	0,0057	0,18	-5,59	
hsa-miR-145	0,0493	0,13	-7,61	
hsa-miR-875-5p	0,0089	0,12	-8,15	
hsa-miR-638	0,0368	0,06	-16,95	
hsa-miR-545	0,0418	0,03	-28,78	

PreBII small comparisons:

miR	p-value	fold change	
hsa-miR-210	0,0016	563,34	higher in children
hsa-miR-34a*	0,0000	248,78	
hsa-miR-149	0,0032	169,84	
hsa-miR-27b	0,0075	112,76	
hsa-miR-7-2*	0,0015	85,84	
hsa-miR-455-3p	0,0289	84,17	
hsa-miR-744*	0,0294	36,15	
hsa-miR-148b*	0,0002	34,74	
hsa-miR-455-5p	0,0010	32,63	
hsa-let-7i*	0,0002	30,53	
hsa-miR-17*	0,0417	28,64	
hsa-miR-331-5p	0,0373	28,32	
hsa-miR-642	0,0250	27,39	
hsa-miR-380*	0,0142	27,15	
hsa-miR-107	0,0241	24,92	
hsa-miR-629*	0,0364	24,72	
hsa-miR-31	0,0451	23,91	

hsa-miR-129-3p	0,0456	20,79
hsa-miR-19a*	0,0164	14,19
hsa-miR-20b*	0,0206	14,12
hsa-let-7g*	0,0255	13,90
hsa-miR-18b	0,0114	12,92
hsa-miR-339-5p	0,0046	10,28
hsa-miR-215	0,0238	9,26
hsa-miR-15a*	0,0112	8,71
hsa-miR-362-3p	0,0332	7,97
hsa-miR-425*	0,0046	7,92
hsa-miR-296-5p	0,0033	7,21
hsa-let-7f	0,0246	7,02
hsa-miR-301a	0,0146	6,84
hsa-miR-30a*	0,0089	6,59
hsa-miR-590-5p	0,0127	6,12
hsa-miR-142-5p	0,0100	5,68
hsa-miR-18a*	0,0021	5,41
hsa-miR-324-5p	0,0043	5,30
hsa-miR-301b	0,0227	5,24
hsa-miR-181a*	0,0188	5,15
hsa-miR-15a	0,0070	5,08
hsa-miR-19a	0,0099	5,06
hsa-miR-106b	0,0270	5,01
hsa-miR-30e*	0,0040	4,99
hsa-miR-652	0,0024	4,94
hsa-miR-363	0,0376	4,75
hsa-miR-7-1*	0,0165	4,73
hsa-miR-142-3p	0,0200	4,68
hsa-miR-95	0,0047	4,53
hsa-miR-18a	0,0254	4,53
hsa-miR-339-3p	0,0010	4,41
hsa-miR-345	0,0073	4,29
hsa-miR-200c	0,0073	4,14
hsa-miR-625	0,0051	4,10
hsa-miR-20a	0,0128	4,04
hsa-miR-30e	0,0492	4,01
hsa-miR-671-3p	0,0341	3,99
hsa-miR-21	0,0262	3,99
hsa-miR-16-1*	0,0444	3,97
hsa-miR-19b	0,0151	3,93
hsa-miR-378	0,0225	3,81
hsa-miR-93	0,0084	3,79
hsa-miR-140-5p	0,0171	3,78
hsa-miR-181c	0,0093	3,71

hsa-miR-93*	0,0018	3,71		
hsa-miR-103	0,0025	3,71		
hsa-miR-532-3p	0,0157	3,69		
hsa-miR-744	0,0166	3,47		
hsa-miR-766	0,0072	3,45		
hsa-miR-17	0,0167	3,35		
hsa-miR-25	0,0154	3,34		
hsa-miR-181a	0,0367	3,31		
hsa-miR-106a	0,0191	3,25		
hsa-miR-130b	0,0266	3,21		
hsa-miR-769-5p	0,0483	3,13		
hsa-miR-766	0,0066	3,13		
hsa-miR-331-3p	0,0220	3,05		
hsa-miR-20b	0,0164	3,03		
hsa-miR-15b	0,0111	3,01		
hsa-miR-195	0,0181	3,00		
hsa-miR-16	0,0120	2,98		
hsa-miR-192	0,0089	2,92		
hsa-miR-28-5p	0,0112	2,89		
hsa-miR-128	0,0458	2,83		
hsa-miR-186	0,0329	2,76		
hsa-miR-140-3p	0,0297	2,76		
hsa-miR-30c	0,0190	2,74		
hsa-miR-30b	0,0405	2,63		
hsa-miR-362-5p	0,0331	2,63		
hsa-miR-625*	0,0081	2,56		
hsa-miR-374b	0,0436	2,51		
hsa-let-7g	0,0327	2,50		
hsa-miR-425	0,0175	2,39		
hsa-let-7d	0,0432	2,38		
hsa-miR-223	0,0435	2,30		
hsa-miR-191	0,0481	2,23		
hsa-miR-484	0,0442	2,05		
hsa-miR-125b	0,0049	0,21	-4,86	lower in children
hsa-miR-125a-5p	0,0359	0,04	-27,48	

Immature B comparisons:

miR	p-value	fold change		
hsa-miR-629*	0,0235	33,35		higher in children
hsa-miR-455-3p	0,0360	21,19		
hsa-miR-571	0,0315	0,39	-2,54	lower in children

hsa-miR-485-3p	0,0468	0,38	-2,65
hsa-miR-135a*	0,0369	0,36	-2,81
hsa-miR-346	0,0077	0,34	-2,93
hsa-miR-630	0,0480	0,34	-2,97
hsa-miR-610	0,0146	0,33	-3,07
hsa-miR-597	0,0042	0,32	-3,11
hsa-miR-138-1*	0,0105	0,31	-3,23
hsa-miR-509-3p	0,0019	0,30	-3,31
hsa-miR-323-3p	0,0003	0,30	-3,31
hsa-miR-188-5p	0,0025	0,29	-3,42
hsa-miR-513-3p	0,0361	0,29	-3,47
hsa-miR-877	0,0004	0,28	-3,52
hsa-miR-571	0,0003	0,28	-3,56
hsa-miR-760	0,0016	0,28	-3,62
hsa-miR-519a	0,0198	0,27	-3,64
hsa-miR-645	0,0029	0,27	-3,74
hsa-miR-520a-5p	0,0338	0,26	-3,82
hsa-miR-147	0,0044	0,24	-4,12
hsa-miR-632	0,0014	0,23	-4,37
hsa-miR-19b-1*	0,0413	0,22	-4,56
hsa-miR-645	0,0134	0,21	-4,70
hsa-miR-99b*	0,0013	0,21	-4,74
hsa-miR-646	0,0290	0,17	-6,02
hsa-miR-549	0,0355	0,13	-7,49
hsa-miR-18b*	0,0357	0,13	-7,51
hsa-let-7f-2*	0,0371	0,13	-7,62
hsa-miR-520a-3p	0,0328	0,13	-7,96
hsa-miR-20b*	0,0465	0,12	-8,39
hsa-miR-25*	0,0093	0,11	-9,04
hsa-miR-580	0,0405	0,11	-9,46
hsa-miR-656	0,0292	0,06	-15,84
hsa-let-7b*	0,0170	0,06	-16,69
hsa-miR-19a*	0,0167	0,06	-17,35
hsa-miR-374b*	0,0297	0,04	-24,95
hsa-miR-659	0,0113	0,04	-27,11
hsa-miR-127-3p	0,0467	0,03	-34,24
hsa-miR-214	0,0130	0,03	-35,05
hsa-miR-520d-5p	0,0110	0,03	-36,99
hsa-miR-145	0,0221	0,02	-40,31
hsa-miR-489	0,0056	0,02	-45,78
hsa-miR-511	0,0141	0,02	-48,62
hsa-miR-636	0,0303	0,01	-136,66
