# Supplementary material for: Transcriptional Profiling of mRNAs and microRNAs in Human Bone Marrow Precursor B Cells Identifies Subset- and Age-Specific Variations
Source: PLoS One. 2013 Jul 30;8(7):e70721. doi: 10.1371/journal.pone.0070721 (PMC3728296; doi:10.1371/journal.pone.0070721)
Supplement: Table S11 — (PDF) [file pone.0070721.s015.pdf]

Analysis Name: Child_PreBI vs ProB_miR
Analysis Creation Date: 2013-06-09
Build version: 220217
Content version: 16542223 (Release Date: 2013-05-13)

Analysis settings

View

Reference set: Ingenuity Knowledge Base (Genes + Endogenous Chemicals)

Relationship to include: Direct and Indirect

Includes Endogenous Chemicals

Optional Analyses: My Pathways My List

Filter Summary:

Consider only molecules and/or relationships where

(species = Rat OR Human OR Mouse) AND

(confidence = Experimentally Observed OR High (predicted))

Cutoff:

Top Networks

ID	Associated Network Functions	Score
1	Hereditary Disorder, Skeletal and Muscular Disorders, Developmental Disorder	27
2	Inflammatory Disease, Hereditary Disorder, Skeletal and Muscular Disorders	22

(c) 2000-2013 Ingenuity Systems, Inc. All rights reserved.

3		3
4		3
5	Developmental Disorder, Hereditary Disorder, Skeletal and Muscular Disorders	3

(c) 2000-2013 Ingenuity Systems, Inc. All rights reserved.

Top Bio Functions

Diseases and Disorders

Name	p-value	# Molecules
Hereditary Disorder	9,48E-18 - 4,77E-02	9
Skeletal and Muscular Disorders	9,48E-18 - 1,74E-09	8
Developmental Disorder	5,81E-17 - 4,77E-02	9
Dermatological Diseases and Conditions	1,47E-12 - 1,47E-12	6
Inflammatory Disease	1,47E-12 - 3,35E-06	8

Molecular and Cellular Functions

Name	p-value	# Molecules
Cell Death and Survival	1,64E-04 - 4,32E-02	6
Cellular Development	5,20E-04 - 4,66E-02	8
Cellular Growth and Proliferation	5,20E-04 - 2,95E-02	8
Cell Morphology	2,22E-03 - 3,70E-02	3
Cell-To-Cell Signaling and Interaction	3,32E-03 - 3,17E-02	3

Physiological System Development and Function

Name	p-value	# Molecules
Skeletal and Muscular System Development and Function	5,20E-04 - 3,57E-02	4
Connective Tissue Development and Function	8,26E-04 - 4,43E-03	4
Cardiovascular System Development and Function	2,22E-03 - 3,70E-02	2
Digestive System Development and Function	2,22E-03 - 2,22E-03	1
Hepatic System Development and Function	2,22E-03 - 2,22E-03	1

(c) 2000-2013 Ingenuity Systems, Inc. All rights reserved.

INGENUITY® SYSTEMS

Top Canonical Pathways

Name	p-value	Ratio
Regulation of the Epithelial-Mesenchymal Transition Pathway	1,68E-02	2/190 (0,011)
Systemic Lupus Erythematosus Signaling	2,2E-01	1/246 (0,004)

Top Molecules

Fold Change up-regulated

Molecules	Exp. Value	Exp. Chart
RNU6-2*	↑7,714	

Fold Change down-regulated

Molecules	Exp. Value	Exp. Chart
miR-133a-3p (and other miRNAs w/seed UUGGUCC)	↓-159,510	
mir-515*	↓-40,645	
mir-638	↓-40,434	
miR-628-3p (miRNAs w/seed CUAGUAA)	↓-30,221	
mir-657	↓-29,091	
mir-143	↓-27,143	
miR-511 (miRNAs w/seed UGUCUUU)	↓-20,112	
miR-875-5p (and other miRNAs w/seed AUACCUC)	↓-16,622	
mir-379	↓-14,074	
mir-137	↓-13,109	

Top Upstream Regulators

(c) 2000-2013 Ingenuity Systems, Inc. All rights reserved.

(c) 2000-2013 Ingenuity Systems, Inc. All rights reserved.

Top My Lists

Name	p-value	Ratio
PreBI vs PreBIIL_miR_adults	1,65E-02	1/18 (0,056)
PreBI vs PreBII_miR and mRNA_network2_adults	2,63E-02	1/28 (0,036)
PreBI vs PreBIIL_miRs only_adults	4,24E-02	1/51 (0,02)
PreBI vs PreBIIL_miR_and mRNA_adults	5,19E-02	1/63 (0,016)
PreBI vs PreBII_miR target filter_cell cycle_ID2_c	5,3E-02	1/51 (0,02)

Top My Pathways

Name	p-value	Ratio
PreBI vs PreBIIL_miRs and mRNA_voksne	5,72E-02	1/60 (0,017)

Top Tox Lists

Name	p-value	Ratio
Hepatic Fibrosis	1,01E-01	1/96 (0,01)

(c) 2000-2013 Ingenuity Systems, Inc. All rights reserved.

Top Tox Functions

Cardiotoxicity

Name	p-value	# Molecules
Cardiac Hypertrophy	1,87E-02 - 1,28E-01	1
Cardiac Hypoplasia	2,41E-02 - 2,41E-02	1
Cardiac Necrosis/Cell Death	3,27E-02 - 3,27E-02	1
Cardiac Infarction	6,24E-02 - 6,24E-02	1
Cardiac Proliferation	7,79E-02 - 7,79E-02	1

Hepatotoxicity

Name	p-value	# Molecules
Liver Hyperplasia/Hyperproliferation	4,46E-06 - 6,24E-02	7
Hepatocellular Carcinoma	9,07E-06 - 6,24E-02	6
Liver Fibrosis	6,63E-03 - 6,63E-03	1
Liver Steatosis	2,53E-01 - 2,53E-01	1
Liver Inflammation/Hepatitis	2,62E-01 - 2,62E-01	1

Nephrotoxicity

Name	p-value	# Molecules
Renal Inflammation	1,01E-01 - 1,01E-01	1
Renal Nephritis	1,01E-01 - 1,01E-01	1

(c) 2000-2013 Ingenuity Systems, Inc. All rights reserved.

Analysis Name: Child_PreBII L vs PreBI_miR
Analysis Creation Date: 2013-06-09
Build version: 220217
Content version: 16542223 (Release Date: 2013-05-13)

Analysis settings

View

Reference set: Ingenuity Knowledge Base (Genes + Endogenous Chemicals)

Relationship to include: Direct and Indirect

Includes Endogenous Chemicals

Optional Analyses: My Pathways My List

Filter Summary:

Consider only molecules and/or relationships where

(species = Rat OR Human OR Mouse) AND

(confidence = Experimentally Observed OR High (predicted))

Cutoff:

Top Networks

ID	Associated Network Functions	Score
1	Hereditary Disorder, Skeletal and Muscular Disorders, Developmental Disorder	37
2	Endocrine System Disorders, Reproductive System Disease, Hereditary Disorder	23

(c) 2000-2013 Ingenuity Systems, Inc. All rights reserved.

3	Cellular Growth and Proliferation, Connective Tissue Development and Function, Cellular Compromise	20
4	Tissue Morphology, Cell Morphology, Hematological System Development and Function	2
5	Hereditary Disorder, Skeletal and Muscular Disorders, Cell-To-Cell Signaling and Interaction	2

(c) 2000-2013 Ingenuity Systems, Inc. All rights reserved.

Top Bio Functions

Diseases and Disorders

Name	p-value	# Molecules
Cancer	2,64E-16 - 4,97E-02	28
Gastrointestinal Disease	2,64E-16 - 1,75E-03	25
Hereditary Disorder	1,18E-15 - 1,91E-02	14
Skeletal and Muscular Disorders	1,18E-15 - 4,12E-02	10
Connective Tissue Disorders	4,79E-14 - 4,12E-02	12

Molecular and Cellular Functions

Name	p-value	# Molecules
Cellular Movement	1,46E-05 - 2,25E-02	13
Cellular Development	2,28E-05 - 4,34E-02	16
Cellular Growth and Proliferation	2,28E-05 - 4,45E-02	16
Cell Cycle	1,75E-03 - 2,42E-02	2
Cell Death and Survival	1,75E-03 - 4,79E-02	14

Physiological System Development and Function

Name	p-value	# Molecules
Cardiovascular System Development and Function	1,32E-04 - 3,10E-02	8
Organismal Development	6,09E-04 - 3,10E-02	8
Tissue Development	6,09E-04 - 4,45E-02	7
Tumor Morphology	1,75E-03 - 4,79E-02	5
Hematological System Development and Function	3,05E-03 - 4,12E-02	4

(c) 2000-2013 Ingenuity Systems, Inc. All rights reserved.

Top Canonical Pathways

Name	p-value	Ratio
Regulation of the Epithelial-Mesenchymal Transition Pathway	3,97E-02	2/190 (0,011)

Top Molecules

Fold Change up-regulated

Molecules	Exp. Value	Exp. Chart
mir-210	↑103,788	
miR-455-3p (miRNAs w/seed CAGUCCA)	↑46,932	
miR-642a-5p (miRNAs w/seed UCCCUCU)	↑36,127	
mir-8	↑34,356	
miR-483-5p (miRNAs w/seed AGACGGG)	↑19,027	
mir-650	↑18,604	
miR-129-1-3p (and other miRNAs w/seed AGCCCUU)	↑14,197	

Fold Change down-regulated

Molecules	Exp. Value	Exp. Chart
miR-125b-5p (and other miRNAs w/seed CCCUGAG)	↓-96,336	
mir-196	↓-77,574	
mir-515	↓-71,383	
miR-7-1-3p (and other miRNAs w/seed AACAAAU)	↓-51,536	
miR-126-5p (and other miRNAs w/seed AUUAUUA)	↓-49,180	
mir-126	↓-45,020	
miR-29b-1-5p (miRNAs w/seed CUGGUUU)	↓-33,359	
miR-192-3p (miRNAs w/seed UGCCAAU)	↓-17,784	
mir-95	↓-17,509	
mir-31	↓-16,393	

(c) 2000-2013 Ingenuity Systems, Inc. All rights reserved.

INGENUITY® SYSTEMS

Top Upstream Regulators

(c) 2000-2013 Ingenuity Systems, Inc. All rights reserved.

Top My Lists

Name	p-value	Ratio
PreBI vs PreBIIL_miRs only_adults	6,38E-09	5/51 (0,098)
PreBI vs PreBIIL_miR_adults	1,03E-08	4/18 (0,222)
PreBI vs PreBII_miR and mRNA_network2_adults	9,55E-06	3/28 (0,107)
PreBI vs PreBIIL_miR_and mRNA_adults	7,93E-05	3/63 (0,048)
PreBI vs PreBII_miR target filter_cell cycle_ID2_c	8,44E-05	3/51 (0,059)

Top My Pathways

Name	p-value	Ratio
PreBI vs PreBII L_miR og mRNA_core TF_barn	6,3E-06	3/26 (0,115)
PreBI vs PreBIIL_miRs and mRNA_voksne	1,07E-04	3/60 (0,05)

Top Tox Lists

Name	p-value	Ratio
Renal Ischemia-Reperfusion Injury MicroRNA Biomarker Panel (Mouse)	1,39E-02	1/8 (0,125)
Liver Proliferation	4,93E-02	2/204 (0,01)
Liver Necrosis/Cell Death	7,65E-02	2/262 (0,008)
Increases Liver Hyperplasia/Hyperproliferation	9,83E-02	1/59 (0,017)
Cardiac Hypertrophy	4,56E-01	1/344 (0,003)

(c) 2000-2013 Ingenuity Systems, Inc. All rights reserved.

Top Tox Functions

Assays: Clinical Chemistry and Hematology

Name	p-value	# Molecules
Increased Levels of Red Blood Cells	1,55E-01 - 1,55E-01	1
Increased Levels of Hematocrit	1,57E-01 - 1,57E-01	1

Cardiotoxicity

Name	p-value	# Molecules
Cardiac Damage	8,72E-03 - 8,72E-03	1
Cardiac Infarction	1,74E-02 - 9,67E-02	2
Congenital Heart Anomaly	1,91E-02 - 1,91E-02	1
Pulmonary Hypertension	9,67E-02 - 9,67E-02	1
Cardiac Fibrosis	1,83E-01 - 1,83E-01	1

Hepatotoxicity

Name	p-value	# Molecules
Liver Hyperplasia/Hyperproliferation	5,58E-10 - 1,75E-03	12
Hepatocellular Carcinoma	1,17E-06 - 1,17E-06	8
Liver Necrosis/Cell Death	2,76E-02 - 2,07E-01	2
Liver Proliferation	4,34E-02 - 2,35E-01	2
Liver Inflammation/Hepatitis	5,12E-02 - 5,12E-02	1

Nephrotoxicity

Name	p-value	# Molecules
Renal Inflammation	1,22E-02 - 1,22E-02	2
Renal Nephritis	1,22E-02 - 1,22E-02	2

(c) 2000-2013 Ingenuity Systems, Inc. All rights reserved.

(c) 2000-2013 Ingenuity Systems, Inc. All rights reserved.

Analysis Name: Child_PreBII s vs PreBII L_miR
Analysis Creation Date: 2013-06-09
Build version: 220217
Content version: 16542223 (Release Date: 2013-05-13)

Analysis settings

View

Reference set: Ingenuity Knowledge Base (Genes Only)

Relationship to include: Direct and Indirect

Includes Endogenous Chemicals

Optional Analyses: My Pathways My List

Filter Summary:

Consider only molecules and/or relationships where

(species = Rat OR Human OR Mouse) AND

(confidence = Experimentally Observed OR High (predicted))

Cutoff:

Top Networks

ID	Associated Network Functions	Score
1	Cardiovascular System Development and Function, Cellular Development, Cellular Growth and Proliferation	31
2	Cell-To-Cell Signaling and Interaction, Tissue Development, Cellular Development	25

(c) 2000-2013 Ingenuity Systems, Inc. All rights reserved.

3	Cancer, Cell Morphology, Organ Morphology	25
4	Reproductive System Disease, Developmental Disorder, Hereditary Disorder	23
5	Cell Signaling, Molecular Transport, Nucleic Acid Metabolism	21

(c) 2000-2013 Ingenuity Systems, Inc. All rights reserved.

Top Bio Functions

Diseases and Disorders

Name	p-value	# Molecules
Reproductive System Disease	9,02E-87 - 6,48E-05	73
Inflammatory Disease	1,27E-32 - 3,94E-02	43
Inflammatory Response	1,27E-32 - 4,13E-02	33
Renal and Urological Disease	1,27E-32 - 4,66E-06	50
Cancer	5,24E-19 - 4,94E-02	81

Molecular and Cellular Functions

Name	p-value	# Molecules
Cellular Development	5,82E-07 - 4,64E-02	10
Cellular Growth and Proliferation	1,90E-03 - 1,67E-02	6
Cell Cycle	8,40E-03 - 4,64E-02	6
Cell-To-Cell Signaling and Interaction	8,40E-03 - 4,13E-02	3
Cellular Assembly and Organization	8,40E-03 - 8,40E-03	1

Physiological System Development and Function

Name	p-value	# Molecules
Tissue Morphology	1,03E-03 - 1,67E-02	3
Digestive System Development and Function	2,00E-03 - 2,00E-03	3
Hepatic System Development and Function	2,00E-03 - 2,00E-03	3
Organ Development	2,00E-03 - 4,13E-02	4
Hair and Skin Development and Function	8,40E-03 - 8,40E-03	1

(c) 2000-2013 Ingenuity Systems, Inc. All rights reserved.

INGENUITY® SYSTEMS

Top Canonical Pathways

Name	p-value	Ratio

Top Molecules

Fold Change up-regulated

Molecules	Exp. Value	Exp. Chart
miR-657 (miRNAs w/seed GCAGGUU)*	↑773,362	
miR-7-1-3p (and other miRNAs w/seed AACAAAU)	↑173,045	
miR-383 (and other miRNAs w/seed GAUCAGA)	↑81,149	
miR-758-3p (and other miRNAs w/seed UUGUGAC)	↑33,475	
miR-17-5p (and other miRNAs w/seed AAAGUGC)	↑28,690	
miR-518a-3p (and other miRNAs w/seed AAAGCGC)*	↑27,522	
let-7i-3p (miRNAs w/seed UGCGCAA)	↑26,723	
miR-376a-3p (and other miRNAs w/seed UCAUAGA)	↑18,001	
miR-10a-3p (miRNAs w/seed AAAUUCG)	↑16,825	
miR-669d-5p (and other miRNAs w/seed CUUGUGU)	↑16,825	

Fold Change down-regulated

Molecules	Exp. Value	Exp. Chart

Top Upstream Regulators

(c) 2000-2013 Ingenuity Systems, Inc. All rights reserved.

INGENUITY® SYSTEMS

Top My Lists

Name	p-value	Ratio
PreBI vs PreBIIL_miR_adults	6,85E-03	3/18 (0,167)
PreBI vs PreBIIL_miRs only_adults	4,24E-02	3/51 (0,059)
PreBI vs PreBII_miR target filter_cell cycle_ID2_c	6,38E-02	3/51 (0,059)
PreBI vs PreBII_miR and mRNA_network2_adults	1,83E-01	1/28 (0,036)
PreBI vs PreBIIL_miR_and mRNA_adults	3,33E-01	1/63 (0,016)

Top My Pathways

Name	p-value	Ratio
PreBI vs PreBIIL_miRs and mRNA_voksne	3,61E-01	1/60 (0,017)

Top Tox Lists

Name	p-value	Ratio
Renal Ischemia-Reperfusion Injury MicroRNA Biomarker Panel (Mouse)	1,9E-03	2/8 (0,25)

(c) 2000-2013 Ingenuity Systems, Inc. All rights reserved.

Top Tox Functions

Cardiotoxicity

Name	p-value	# Molecules
Cardiac Inflammation	7,01E-05 - 7,01E-05	2
Cardiac Infarction	8,54E-02 - 1,87E-01	3
Pulmonary Hypertension	2,24E-01 - 2,24E-01	1
Cardiac Fibrosis	5,52E-01 - 6,22E-01	2
Cardiac Arteriopathy	1,00E00 - 1,00E00	2

Hepatotoxicity

Name	p-value	# Molecules
Liver Hyperplasia/Hyperproliferation	3,35E-06 - 1,48E-01	19
Hepatocellular Carcinoma	1,99E-04 - 1,99E-04	13
Liver Inflammation/Hepatitis	2,00E-03 - 2,00E-03	3
Liver Steatosis	6,77E-03 - 6,77E-03	3
Liver Cirrhosis	1,05E-01 - 1,05E-01	3

Nephrotoxicity

Name	p-value	# Molecules
Renal Inflammation	1,27E-32 - 1,27E-32	26
Renal Nephritis	1,27E-32 - 1,27E-32	26

(c) 2000-2013 Ingenuity Systems, Inc. All rights reserved.

INGENUITY® SYSTEMS

Analysis Name: Child_Immature B vs PreBII s_miR
Analysis Creation Date: 2013-06-09
Build version: 220217
Content version: 16542223 (Release Date: 2013-05-13)

Analysis settings

View

Reference set: Ingenuity Knowledge Base (Genes Only)

Relationship to include: Direct and Indirect

Includes Endogenous Chemicals

Optional Analyses: My Pathways My List

Filter Summary:

Consider only molecules and/or relationships where

(species = Rat OR Human OR Mouse) AND

(confidence = Experimentally Observed OR High (predicted))

Cutoff:

Top Networks

ID	Associated Network Functions	Score
1	Carbohydrate Metabolism, Lipid Metabolism, Small Molecule Biochemistry	28
2	Gene Expression, Cell Morphology, Cellular Compromise	21

(c) 2000-2013 Ingenuity Systems, Inc. All rights reserved.

3	Cancer, Hematological Disease, Hereditary Disorder	21
4	Endocrine System Disorders, Reproductive System Disease, Cancer	19
5	Cancer, Immunological Disease, Inflammatory Disease	19

(c) 2000-2013 Ingenuity Systems, Inc. All rights reserved.

Top Bio Functions

Diseases and Disorders

Name	p-value	# Molecules
Reproductive System Disease	9,62E-63 - 4,90E-02	44
Inflammatory Disease	5,65E-20 - 1,35E-02	25
Inflammatory Response	5,65E-20 - 2,65E-02	23
Renal and Urological Disease	5,65E-20 - 1,07E-02	30
Cancer	1,05E-11 - 4,90E-02	48

Molecular and Cellular Functions

Name	p-value	# Molecules
Cellular Development	1,23E-05 - 4,73E-02	7
Cell Death and Survival	4,12E-03 - 4,73E-02	5
Cell Cycle	5,37E-03 - 3,70E-02	4
Cell-To-Cell Signaling and Interaction	5,37E-03 - 2,65E-02	3
Cellular Assembly and Organization	5,37E-03 - 4,25E-02	3

Physiological System Development and Function

Name	p-value	# Molecules
Digestive System Development and Function	5,11E-07 - 5,11E-07	5
Hepatic System Development and Function	5,11E-07 - 5,11E-07	5
Organ Development	5,11E-07 - 2,65E-02	6
Tissue Morphology	4,21E-04 - 1,07E-02	3
Organismal Development	1,00E-03 - 4,73E-02	3

(c) 2000-2013 Ingenuity Systems, Inc. All rights reserved.

Top Canonical Pathways

Name	p-value	Ratio

Top Molecules

Fold Change up-regulated

Molecules	Exp. Value	Exp. Chart

Fold Change down-regulated

Molecules	Exp. Value	Exp. Chart
miR-149-5p (miRNAs w/seed CUGGCUC)	↓-72,129	
miR-20b-3p (miRNAs w/seed CUGUAGU)	↓-52,528	
miR-636 (miRNAs w/seed GUGCUUG)	↓-49,265	
miR-383 (and other miRNAs w/seed GAUCAGA)	↓-48,168	
miR-130a-3p (and other miRNAs w/seed AGUGCAA)*	↓-47,013	
miR-551b-3p (and other miRNAs w/seed CGACCCA)	↓-41,643	
miR-758-3p (and other miRNAs w/seed UUGUGAC)	↓-37,857	
miR-23a-3p (and other miRNAs w/seed UCACAUU)*	↓-36,758	
miR-25-5p (miRNAs w/seed GGCGGAG)	↓-35,200	
miR-659-3p (miRNAs w/seed UUGGUUC)	↓-31,450	

Top Upstream Regulators

(c) 2000-2013 Ingenuity Systems, Inc. All rights reserved.

Top My Lists

Name	p-value	Ratio
miR-126 PreBI/PreBII large children	6,76E-02	1/15 (0,067)
PreBI vs PreBIIL_miR_adults	7,76E-02	3/18 (0,167)
PreBI vs PreBII_miR and mRNA_network2_adults	1,21E-01	2/28 (0,071)
PreBI vs PreBIIL_miRs only_adults	1,89E-01	3/51 (0,059)
PreBI vs PreBIIL_miR_and mRNA_adults	2,28E-01	2/63 (0,032)

Top My Pathways

Name	p-value	Ratio
PreBI vs PreBII L_miR og mRNA_core TF_barn	5,6E-03	3/26 (0,115)
mir-126 PreBI/PreBII large children	6,76E-02	1/15 (0,067)
PreBI vs PreBIIL_miRs and mRNA_voksne	2,48E-01	2/60 (0,033)

Top Tox Lists

Name	p-value	Ratio
Renal Ischemia-Reperfusion Injury MicroRNA Biomarker Panel (Mouse)	4,21E-02	1/8 (0,125)

(c) 2000-2013 Ingenuity Systems, Inc. All rights reserved.

Top Tox Functions

Cardiotoxicity

Name	p-value	# Molecules
Congenital Heart Anomaly	5,75E-02 - 5,75E-02	1
Pulmonary Hypertension	1,49E-01 - 1,49E-01	1
Cardiac Arteriopathy	2,11E-01 - 2,11E-01	3
Cardiac Infarction	2,68E-01 - 2,68E-01	1
Cardiac Fibrosis	4,63E-01 - 4,63E-01	1

Hepatotoxicity

Name	p-value	# Molecules
Liver Hyperplasia/Hyperproliferation	4,81E-07 - 9,72E-02	16
Liver Inflammation/Hepatitis	5,11E-07 - 5,11E-07	5
Hepatocellular Carcinoma	2,41E-04 - 2,41E-04	10
Liver Cirrhosis	7,99E-04 - 7,99E-04	5
Liver Steatosis	2,53E-02 - 2,53E-02	2

Nephrotoxicity

Name	p-value	# Molecules
Renal Inflammation	5,65E-20 - 5,65E-20	16
Renal Nephritis	5,65E-20 - 5,65E-20	16
Renal Necrosis/Cell Death	1,00E00 - 1,00E00	1

(c) 2000-2013 Ingenuity Systems, Inc. All rights reserved.

INGENUITY® SYSTEMS
