# Supplementary material for: Transcriptional Profiling of mRNAs and microRNAs in Human Bone Marrow Precursor B Cells Identifies Subset- and Age-Specific Variations
Source: PLoS One. 2013 Jul 30;8(7):e70721. doi: 10.1371/journal.pone.0070721 (PMC3728296; doi:10.1371/journal.pone.0070721)
Supplement: Table S12 — (PDF) [file pone.0070721.s016.pdf]

Analysis Name: Adult_PreBI vs ProB_miR
Analysis Creation Date: 2013-06-09
Build version: 220217
Content version: 16542223 (Release Date: 2013-05-13)

Analysis settings

View

Reference set: Ingenuity Knowledge Base (Genes Only)

Relationship to include: Direct and Indirect

Includes Endogenous Chemicals

Optional Analyses: My Pathways My List

Filter Summary:

Consider only molecules and/or relationships where

(species = Rat OR Human OR Mouse) AND

(confidence = Experimentally Observed OR High (predicted))

Cutoff:

Top Networks

ID	Associated Network Functions	Score
1	Cell Cycle, Cellular Assembly and Organization, Cellular Function and Maintenance	21
2	Cancer, Reproductive System Disease, Tumor Morphology	18

(c) 2000-2013 Ingenuity Systems, Inc. All rights reserved.

3		3
4	Cellular Development, Cellular Growth and Proliferation, Nervous System Development and Function	3
5	Cancer, Cellular Development, Tumor Morphology	3

(c) 2000-2013 Ingenuity Systems, Inc. All rights reserved.

INGENUITY® SYSTEMS

Top Bio Functions

Diseases and Disorders

Name	p-value	# Molecules
Inflammatory Disease	1,26E-10 - 4,79E-02	6
Inflammatory Response	1,26E-10 - 4,54E-02	6
Renal and Urological Disease	1,26E-10 - 4,87E-04	9
Reproductive System Disease	9,85E-08 - 3,50E-03	8
Cancer	1,66E-06 - 4,29E-02	8

Molecular and Cellular Functions

Name	p-value	# Molecules
Cellular Development	3,50E-03 - 1,86E-02	4
Cellular Growth and Proliferation	4,37E-03 - 1,86E-02	4
RNA Post-Transcriptional Modification	4,37E-03 - 4,37E-03	1
Cellular Function and Maintenance	6,98E-03 - 6,98E-03	1
Cell Cycle	2,59E-02 - 2,76E-02	2

Physiological System Development and Function

Name	p-value	# Molecules
Hair and Skin Development and Function	8,75E-04 - 1,74E-02	1
Hematological System Development and Function	3,50E-03 - 6,98E-03	1
Organ Morphology	3,50E-03 - 3,50E-03	1
Respiratory System Development and Function	3,50E-03 - 3,50E-03	1
Tumor Morphology	3,50E-03 - 3,50E-03	1

(c) 2000-2013 Ingenuity Systems, Inc. All rights reserved.

INGENUITY SYSTEMS

Top Canonical Pathways

Name	p-value	Ratio

Top Molecules

Fold Change up-regulated

Molecules	Exp. Value	Exp. Chart
miR-96-5p (and other miRNAs w/seed UUGGCAC)	↑191,009	
miR-657 (miRNAs w/seed GCAGGUU)	↑90,929	
miR-16-1-3p (miRNAs w/seed CAGUAUU)	↑42,322	
miR-185-5p (and other miRNAs w/seed GGAGAGA)	↑34,615	
miR-744-3p (miRNAs w/seed UGUUGCC)	↑30,555	
miR-450b-3p (and other miRNAs w/seed UGGGAUC)*	↑29,175	
miR-582-5p (miRNAs w/seed UACAGUU)	↑27,237	
miR-675-5p (and other miRNAs w/seed GGUGCGG)	↑25,738	
miR-579 (and other miRNAs w/seed UCAUUUG)	↑24,618	
miR-31-5p (and other miRNAs w/seed GGCAAGA)	↑21,869	

Fold Change down-regulated

Molecules	Exp. Value	Exp. Chart
miR-615-3p (miRNAs w/seed CCGAGCC)	↓-48,168	

Top Upstream Regulators

(c) 2000-2013 Ingenuity Systems, Inc. All rights reserved.

Top My Lists

Name	p-value	Ratio
PreBI vs PreBIIL_miRs only_adults	5,2E-04	2/51 (0,039)
PreBI vs PreBIIL_miR_and mRNA_adults	7,87E-04	2/63 (0,032)
PreBI vs PreBII_miR target filter_cell cycle_ID2_c	8,21E-04	2/51 (0,039)
PreBI vs PreBIIL_miR_and mRNA_utvidet_adults	9,6E-04	2/60 (0,033)
PreBI vs PreBIIL-miR target filter_cell cycle_adul	4,13E-03	2/111 (0,018)

Top My Pathways

Name	p-value	Ratio
PreBI vs PreBIIL_miRs and mRNA_voksne	9,6E-04	2/60 (0,033)

Top Tox Lists

Name	p-value	Ratio

(c) 2000-2013 Ingenuity Systems, Inc. All rights reserved.

INGENUITY® SYSTEMS

Top Tox Functions

Cardiotoxicity

Name	p-value	# Molecules
Congenital Heart Anomaly	9,59E-03 - 9,59E-03	1
Cardiac Fibrosis	9,61E-02 - 9,61E-02	1
Cardiac Hypertrophy	1,02E-01 - 1,02E-01	1

Hepatotoxicity

Name	p-value	# Molecules
Liver Steatosis	3,95E-02 - 3,95E-02	1
Hepatocellular Carcinoma	3,46E-01 - 3,46E-01	1
Liver Hyperplasia/Hyperproliferation	3,46E-01 - 3,46E-01	1

Nephrotoxicity

Name	p-value	# Molecules
Renal Inflammation	1,26E-10 - 1,26E-10	6
Renal Nephritis	1,26E-10 - 1,26E-10	6

(c) 2000-2013 Ingenuity Systems, Inc. All rights reserved.

INGENUITY® SYSTEMS

Analysis Name: Adult_PreBII L vs PreBI_miR
Analysis Creation Date: 2013-06-09
Build version: 220217
Content version: 16542223 (Release Date: 2013-05-13)

Analysis settings

View

Reference set: Ingenuity Knowledge Base (Genes + Endogenous Chemicals)

Relationship to include: Direct and Indirect

Includes Endogenous Chemicals

Optional Analyses: My Pathways My List

Filter Summary:

Consider only molecules and/or relationships where

(species = Rat OR Human OR Mouse) AND

(confidence = Experimentally Observed OR High (predicted))

Cutoff:

Top Networks

ID	Associated Network Functions	Score
1	Reproductive System Disease, Cell-To-Cell Signaling and Interaction, Cellular Compromise	27
2	Cell Cycle, Cell Death and Survival, Cancer	19

(c) 2000-2013 Ingenuity Systems, Inc. All rights reserved.

3	Tissue Morphology, Cell Morphology, Hematological System Development and Function	3
4		3
5	Developmental Disorder, Hereditary Disorder, Skeletal and Muscular Disorders	3

(c) 2000-2013 Ingenuity Systems, Inc. All rights reserved.

Top Bio Functions

Diseases and Disorders

Name	p-value	# Molecules
Reproductive System Disease	6,91E-09 - 1,06E-02	13
Endocrine System Disorders	1,17E-07 - 6,63E-03	5
Developmental Disorder	5,09E-06 - 1,70E-02	6
Hereditary Disorder	5,09E-06 - 1,70E-02	6
Skeletal and Muscular Disorders	5,09E-06 - 1,33E-03	3

Molecular and Cellular Functions

Name	p-value	# Molecules
Cell Death and Survival	1,11E-03 - 1,11E-03	1
Cell-To-Cell Signaling and Interaction	1,11E-03 - 1,11E-03	1
Cellular Function and Maintenance	1,11E-03 - 3,49E-02	2
Cellular Movement	1,11E-03 - 4,24E-02	3
Cellular Development	3,32E-03 - 4,98E-02	3

Physiological System Development and Function

Name	p-value	# Molecules
Cardiovascular System Development and Function	1,11E-03 - 3,92E-02	1
Tissue Development	1,11E-03 - 1,10E-02	1
Tumor Morphology	3,32E-03 - 3,32E-03	1
Hematopoiesis	5,53E-03 - 5,53E-03	1
Organismal Development	1,10E-02 - 3,92E-02	1

(c) 2000-2013 Ingenuity Systems, Inc. All rights reserved.

Top Canonical Pathways

Name	p-value	Ratio

Top Molecules

Fold Change up-regulated

Molecules	Exp. Value	Exp. Chart
miR-339-5p (and other miRNAs w/seed CCCUGUC)	↑77,619	
miR-589-3p (miRNAs w/seed CAGAACA)	↑63,302	
miR-642a-5p (miRNAs w/seed UCCCUCU)	↑38,586	
mir-149	↑29,548	

Fold Change down-regulated

Molecules	Exp. Value	Exp. Chart
mir-573	↓-90,562	
mir-675	↓-87,174	
miR-1231-3p (and other miRNAs w/seed GCCCUGU)	↓-48,279	
mir-196	↓-44,170	
miR-450b-3p (and other miRNAs w/seed UGGGAUC)*	↓-41,908	
miR-582-5p (miRNAs w/seed UACAGUU)	↓-41,643	
mir-31	↓-33,436	
miR-519a-3p (and other miRNAs w/seed AAGUGCA)	↓-31,761	
mir-623	↓-30,030	
miR-576-3p (miRNAs w/seed AGAUGUG)	↓-29,754	

Top Upstream Regulators

(c) 2000-2013 Ingenuity Systems, Inc. All rights reserved.

(c) 2000-2013 Ingenuity Systems, Inc. All rights reserved.

Top My Lists

Name	p-value	Ratio
PreBI vs PreBIIL_miRs only_adults	8,41E-04	2/51 (0,039)
PreBI vs PreBIIL_miR_adults	1,65E-02	1/18 (0,056)

Top My Pathways

Name	p-value	Ratio

Top Tox Lists

Name	p-value	Ratio

(c) 2000-2013 Ingenuity Systems, Inc. All rights reserved.

INGENUITY® SYSTEMS

Top Tox Functions

Cardiotoxicity

Name	p-value	# Molecules
Cardiac Damage	5,53E-03 - 5,53E-03	1

Hepatotoxicity

Name	p-value	# Molecules
Liver Hyperplasia/Hyperproliferation	3,60E-02 - 3,60E-02	3

Nephrotoxicity

Name	p-value	# Molecules
Renal Inflammation	4,99E-03 - 4,99E-03	2
Renal Nephritis	4,99E-03 - 4,99E-03	2

(c) 2000-2013 Ingenuity Systems, Inc. All rights reserved.

INGENUITY SYSTEMS

IPA® Interactive pathway analysis of complex 'omics data

INGENUITY® SYSTEMS

Analysis Name: Adult_PreBII s vs PreBII L_miR
Analysis Creation Date: 2013-06-09
Build version: 220217
Content version: 16542223 (Release Date: 2013-05-13)

Analysis settings

View

Reference set: Ingenuity Knowledge Base (Genes Only)

Relationship to include: Direct and Indirect

Includes Endogenous Chemicals

Optional Analyses: My Pathways My List

Filter Summary:

Consider only molecules and/or relationships where

(species = Rat OR Human OR Mouse) AND

(confidence = Experimentally Observed OR High (predicted))

Cutoff:

Top Networks

ID	Associated Network Functions	Score
1	Reproductive System Disease, Cancer, Renal and Urological Disease	28
2		3

(c) 2000-2013 Ingenuity Systems, Inc. All rights reserved.

(c) 2000-2013 Ingenuity Systems, Inc. All rights reserved.

Top Bio Functions

Diseases and Disorders

Name	p-value	# Molecules
Reproductive System Disease	4,30E-09 - 2,18E-02	6
Cancer	2,62E-07 - 4,88E-02	9
Gastrointestinal Disease	2,62E-07 - 3,66E-02	8
Hepatic System Disease	2,62E-07 - 1,56E-02	6
Renal and Urological Disease	7,04E-07 - 4,93E-02	4

Molecular and Cellular Functions

Name	p-value	# Molecules
Cellular Movement	3,20E-04 - 3,85E-02	3
Cell-To-Cell Signaling and Interaction	5,25E-04 - 1,05E-03	2
Cellular Assembly and Organization	5,25E-04 - 5,25E-04	1
Cellular Function and Maintenance	5,25E-04 - 5,25E-04	1
Cell Cycle	1,05E-03 - 2,32E-02	3

Physiological System Development and Function

Name	p-value	# Molecules
Nervous System Development and Function	5,25E-04 - 5,25E-04	1
Tissue Development	5,25E-04 - 1,05E-03	2
Cardiovascular System Development and Function	1,05E-03 - 1,72E-02	2
Skeletal and Muscular System Development and Function	1,05E-03 - 1,05E-03	1
Tissue Morphology	1,05E-03 - 1,05E-03	1

(c) 2000-2013 Ingenuity Systems, Inc. All rights reserved.

Top Canonical Pathways

Name	p-value	Ratio

Top Molecules

Fold Change up-regulated

Molecules	Exp. Value	Exp. Chart
miR-574-3p (miRNAs w/seed ACGCUCA)	↑10,520	
miR-224-5p (miRNAs w/seed AAGUCAC)	↑9,781	

Fold Change down-regulated

Molecules	Exp. Value	Exp. Chart
miR-149-5p (miRNAs w/seed CUGGCUC)	↓-86,372	
miR-331-5p (miRNAs w/seed UAGGUAU)	↓-53,631	
miR-27a-3p (and other miRNAs w/seed UCACAGU)	↓-50,650	
miR-455-5p (and other miRNAs w/seed AUGUGCC)	↓-27,809	
miR-744-3p (miRNAs w/seed UGUUGCC)	↓-16,393	
miR-1 (and other miRNAs w/seed GGAAUGU)	↓-15,834	
miR-198 (miRNAs w/seed GUCCAGA)	↓-9,815	
miR-132-3p (and other miRNAs w/seed AACAGUC)	↓-9,714	
miR-193a-3p (and other miRNAs w/seed ACUGGCC)	↓-6,797	

Top Upstream Regulators

(c) 2000-2013 Ingenuity Systems, Inc. All rights reserved.

Top My Lists

Name	p-value	Ratio
PreBI vs PreBIIL_miR_adults	7,85E-03	2/18 (0,111)
PreBI vs PreBII_miR and mRNA_network2_adults	1,25E-02	2/28 (0,071)
PreBI vs PreBIIL_miRs only_adults	2,03E-02	2/51 (0,039)
PreBI vs PreBIIL_miR_and mRNA_adults	2,49E-02	2/63 (0,032)
PreBI vs PreBII_miR target filter_cell cycle_ID2_c	2,54E-02	2/51 (0,039)

Top My Pathways

Name	p-value	Ratio
PreBI vs PreBIIL_miRs and mRNA_voksne	2,75E-02	2/60 (0,033)

Top Tox Lists

Name	p-value	Ratio

(c) 2000-2013 Ingenuity Systems, Inc. All rights reserved.

Top Tox Functions

Cardiotoxicity

Name	p-value	# Molecules
Cardiac Infarction	3,00E-02 - 3,00E-02	1
Cardiac Hypertrophy	6,28E-02 - 6,28E-02	1
Cardiac Arrythmia	9,35E-02 - 9,35E-02	1
Cardiac Arteriopathy	1,45E-01 - 1,45E-01	1

Hepatotoxicity

Name	p-value	# Molecules
Liver Hyperplasia/Hyperproliferation	2,62E-07 - 6,73E-05	6
Hepatocellular Carcinoma	6,73E-05 - 6,73E-05	4
Liver Inflammation/Hepatitis	1,56E-02 - 1,56E-02	1
Liver Cirrhosis	6,87E-02 - 6,87E-02	1

Nephrotoxicity

Name	p-value	# Molecules
Renal Inflammation	4,93E-02 - 4,93E-02	1
Renal Nephritis	4,93E-02 - 4,93E-02	1

(c) 2000-2013 Ingenuity Systems, Inc. All rights reserved.

INGENUITY® SYSTEMS

Analysis Name: Adult_Immature B vs PreBII s_miR
Analysis Creation Date: 2013-06-09
Build version: 220217
Content version: 16542223 (Release Date: 2013-05-13)

Analysis settings

View

Reference set: Ingenuity Knowledge Base (Genes Only)

Relationship to include: Direct and Indirect

Includes Endogenous Chemicals

Optional Analyses: My Pathways My List

Filter Summary:

Consider only molecules and/or relationships where

(species = Rat OR Human OR Mouse) AND

(confidence = Experimentally Observed OR High (predicted))

Cutoff:

Top Networks

ID	Associated Network Functions	Score
1	Reproductive System Disease, Cell Death and Survival, Cancer	30
2	Hereditary Disorder, Skeletal and Muscular Disorders, Cancer	13

(c) 2000-2013 Ingenuity Systems, Inc. All rights reserved.

3	Cellular Development, Cellular Growth and Proliferation, Hematological System Development and Function	3
4	Cellular Development, Cellular Growth and Proliferation, Tumor Morphology	3
5	Developmental Disorder, Hereditary Disorder, Skeletal and Muscular Disorders	3

(c) 2000-2013 Ingenuity Systems, Inc. All rights reserved.

Top Bio Functions

Diseases and Disorders

Name	p-value	# Molecules
Reproductive System Disease	6,07E-15 - 1,40E-02	9
Cancer	1,85E-05 - 4,84E-02	15
Hereditary Disorder	1,40E-04 - 2,29E-02	4
Skeletal and Muscular Disorders	1,40E-04 - 1,40E-04	2
Endocrine System Disorders	3,08E-04 - 4,72E-02	3

Molecular and Cellular Functions

Name	p-value	# Molecules
Cell Morphology	1,05E-03 - 3,15E-03	2
Cellular Function and Maintenance	1,05E-03 - 1,05E-03	1
Cellular Development	2,10E-03 - 2,90E-02	3
Cell Cycle	7,33E-03 - 7,33E-03	1
Cellular Growth and Proliferation	7,33E-03 - 2,90E-02	2

Physiological System Development and Function

Name	p-value	# Molecules
Behavior	1,05E-03 - 1,05E-03	1
Nervous System Development and Function	1,05E-03 - 1,05E-03	1
Tumor Morphology	2,10E-03 - 5,24E-03	1
Hematological System Development and Function	6,28E-03 - 2,90E-02	2
Hematopoiesis	6,28E-03 - 1,87E-02	1

(c) 2000-2013 Ingenuity Systems, Inc. All rights reserved.

INGENUITY® SYSTEMS

Top Canonical Pathways

Name	p-value	Ratio

Top Molecules

Fold Change up-regulated

Molecules	Exp. Value	Exp. Chart
miR-876-5p (and other miRNAs w/seed GGAUUUC)	↑79,067	
miR-34a-3p (miRNAs w/seed AAUCAGC)	↑59,921	
miR-451a (and other miRNAs w/seed AACCGUU)	↑54,192	
miR-223-5p (miRNAs w/seed GUGUAUU)	↑38,055	
miR-642a-5p (miRNAs w/seed UCCCUCU)	↑37,142	
miR-138-5p (miRNAs w/seed GCUGGUG)	↑34,836	
miR-329 (and other miRNAs w/seed ACACACC)	↑30,910	
miR-149-5p (miRNAs w/seed CUGGCUC)	↑26,218	
miR-489 (and other miRNAs w/seed UGACAUC)	↑26,173	
miR-326-5p (and other miRNAs w/seed GGGGCAG)	↑23,835	

Fold Change down-regulated

Molecules	Exp. Value	Exp. Chart
miR-874-3p (and other miRNAs w/seed UGCCCUG)	↓-35,630	

Top Upstream Regulators

(c) 2000-2013 Ingenuity Systems, Inc. All rights reserved.

Top My Lists

Name	p-value	Ratio
PreBI vs PreBIIL_miR_adults	1,56E-02	1/18 (0,056)
PreBI vs PreBII_miR and mRNA_network1_adults	3,31E-02	1/34 (0,029)
PreBI vs PreBIIL_miRs only_adults	4,02E-02	1/51 (0,02)
PreBI vs PreBIIL_miR_and mRNA_adults	4,92E-02	1/63 (0,016)
PreBI vs PreBII_miR target filter_cell cycle_ID2_c	5,02E-02	1/51 (0,02)

Top My Pathways

Name	p-value	Ratio
PreBI vs PreBIIL_miRs and mRNA_voksne	5,42E-02	1/60 (0,017)

Top Tox Lists

Name	p-value	Ratio

(c) 2000-2013 Ingenuity Systems, Inc. All rights reserved.

Top Tox Functions

Hepatotoxicity

Name	p-value	# Molecules
Liver Hyperplasia/Hyperproliferation	3,12E-02 - 3,12E-02	3
Liver Steatosis	4,72E-02 - 4,72E-02	1

Nephrotoxicity

Name	p-value	# Molecules
Renal Inflammation	4,48E-03 - 4,48E-03	2
Renal Nephritis	4,48E-03 - 4,48E-03	2

(c) 2000-2013 Ingenuity Systems, Inc. All rights reserved.
