# Supplementary material for: Transcriptional Profiling of mRNAs and microRNAs in Human Bone Marrow Precursor B Cells Identifies Subset- and Age-Specific Variations
Source: PLoS One. 2013 Jul 30;8(7):e70721. doi: 10.1371/journal.pone.0070721 (PMC3728296; doi:10.1371/journal.pone.0070721)
Supplement: Table S13 — (PDF) [file pone.0070721.s017.pdf]

Analysis Name: Child ProB vs Adult ProB_miR
Analysis Creation Date: 2013-06-09
Build version: 220217
Content version: 16542223 (Release Date: 2013-05-13)

Analysis settings

View
Reference set: Ingenuity Knowledge Base (Genes Only)
Relationship to include: Direct and Indirect
Includes Endogenous Chemicals
Optional Analyses: My Pathways My List

Filter Summary:
Consider only molecules and/or relationships where
(species = Rat OR Human OR Mouse) AND
(confidence = Experimentally Observed OR High (predicted))
Cutoff:

Top Networks

ID	Associated Network Functions	Score
1	Hereditary Disorder, Skeletal and Muscular Disorders, Reproductive System Disease	33
2	Inflammatory Disease, Inflammatory Response, Renal Inflammation	30

(c) 2000-2013 Ingenuity Systems, Inc. All rights reserved.

3	Cancer, Gastrointestinal Disease, Cardiovascular System Development and Function	17
4	Tissue Morphology, Cancer, Cell Morphology	2
5	Developmental Disorder, Hereditary Disorder, Skeletal and Muscular Disorders	2

(c) 2000-2013 Ingenuity Systems, Inc. All rights reserved.

INGENUITY® SYSTEMS

Top Bio Functions

Diseases and Disorders

Name	p-value	# Molecules
Reproductive System Disease	7,01E-23 - 3,32E-02	21
Inflammatory Disease	3,05E-21 - 5,82E-03	15
Inflammatory Response	3,05E-21 - 1,02E-02	15
Renal and Urological Disease	3,05E-21 - 2,56E-02	17
Cancer	4,74E-14 - 4,98E-02	23

Molecular and Cellular Functions

Name	p-value	# Molecules
Cell Cycle	3,05E-05 - 3,02E-02	6
Cellular Development	1,44E-04 - 4,98E-02	11
Cell Death and Survival	2,04E-03 - 4,01E-02	9
Cellular Compromise	2,04E-03 - 6,11E-03	3
Cellular Growth and Proliferation	2,86E-03 - 4,90E-02	10

Physiological System Development and Function

Name	p-value	# Molecules
Connective Tissue Development and Function	3,05E-05 - 3,05E-05	3
Organismal Development	1,44E-04 - 4,20E-02	3
Digestive System Development and Function	1,70E-03 - 1,70E-03	2
Hepatic System Development and Function	1,70E-03 - 1,70E-03	2
Organ Development	1,70E-03 - 1,02E-02	3

(c) 2000-2013 Ingenuity Systems, Inc. All rights reserved.

Top Canonical Pathways

Name	p-value	Ratio

Top Molecules

Fold Change up-regulated

Molecules	Exp. Value	Exp. Chart
let-7a-5p (and other miRNAs w/seed GAGGUAG)	↑81,855	
miR-657 (miRNAs w/seed GCAGGUU)	↑58,384	
miR-579 (and other miRNAs w/seed UCAUUUG)	↑56,103	
miR-362-5p (and other miRNAs w/seed AUCCUUG)	↑54,948	
miR-454-5p (miRNAs w/seed CCCUAUC)	↑49,095	
miR-16-5p (and other miRNAs w/seed AGCAGCA)*	↑29,600	
miR-660-5p (miRNAs w/seed ACCCAUU)	↑29,344	
miR-16-1-3p (miRNAs w/seed CAGUAUU)	↑23,670	
miR-941 (miRNAs w/seed ACCCGGC)	↑23,223	
miR-524-3p (and other miRNAs w/seed AAGGCGC)	↑20,008	

Fold Change down-regulated

Molecules	Exp. Value	Exp. Chart
miR-615-3p (miRNAs w/seed CCGAGCC)	↓-52,985	
miR-451a (and other miRNAs w/seed AACCGUU)	↓-10,144	

Top Upstream Regulators

(c) 2000-2013 Ingenuity Systems, Inc. All rights reserved.

INGENUITY® SYSTEMS

Top My Lists

Name	p-value	Ratio
PreBI vs PreBIIL_miR_adults	7,79E-11	5/18 (0,278)
PreBI vs PreBIIL_miRs only_adults	1,43E-10	6/51 (0,118)
PreBI vs PreBII_miR target filter_cell cycle_ID2_c	6,05E-10	6/51 (0,118)
PreBI vs PreBIIL_miR_and mRNA_adults	4,23E-08	5/63 (0,079)
PreBI vs PreBIIL_miR_and mRNA_utvidet_adults	7,04E-08	5/60 (0,083)

Top My Pathways

Name	p-value	Ratio
PreBI vs PreBIIL_miRs and mRNA_voksne	7,04E-08	5/60 (0,083)
PreBI vs PreBII L_miR og mRNA_core TF_barn	1,01E-05	3/26 (0,115)
mir-126 PreBI/PreBII large children	2,62E-02	1/15 (0,067)

Top Tox Lists

Name	p-value	Ratio
Renal Ischemia-Reperfusion Injury MicroRNA Biomarker Panel (Mouse)	1,62E-02	1/8 (0,125)
Decreases Transmembrane Potential of Mitochondria and Mitochondrial Membrane	2,12E-01	1/116 (0,009)

(c) 2000-2013 Ingenuity Systems, Inc. All rights reserved.

INGENUITY® SYSTEMS

Top Tox Functions

Cardiotoxicity

Name	p-value	# Molecules
Congenital Heart Anomaly	2,20E-04 - 2,20E-04	2
Cardiac Fibrosis	1,65E-03 - 1,65E-03	3
Cardiac Infarction	2,02E-02 - 2,02E-02	1
Cardiac Dilation	4,98E-02 - 4,98E-02	1
Cardiac Necrosis/Cell Death	5,67E-02 - 3,19E-01	2

Hepatotoxicity

Name	p-value	# Molecules
Liver Hyperplasia/Hyperproliferation	5,80E-07 - 4,28E-05	10
Hepatocellular Carcinoma	4,28E-05 - 4,28E-05	7
Liver Inflammation/Hepatitis	1,70E-03 - 1,70E-03	2
Liver Cirrhosis	3,09E-02 - 3,09E-02	2
Liver Steatosis	8,98E-02 - 8,98E-02	1

Nephrotoxicity

Name	p-value	# Molecules
Renal Inflammation	3,05E-21 - 3,05E-21	13
Renal Nephritis	3,05E-21 - 3,05E-21	13

(c) 2000-2013 Ingenuity Systems, Inc. All rights reserved.

INGENUITY SYSTEMS

Analysis Name: Child PreBI vs Adult PreBI_miR
Analysis Creation Date: 2013-06-09
Build version: 220217
Content version: 16542223 (Release Date: 2013-05-13)

Analysis settings

View

Reference set: Ingenuity Knowledge Base (Genes + Endogenous Chemicals)

Relationship to include: Direct and Indirect

Includes Endogenous Chemicals

Optional Analyses: My Pathways My List

Filter Summary:

Consider only molecules and/or relationships where

(species = Rat OR Human OR Mouse) AND

(confidence = Experimentally Observed OR High (predicted))

Cutoff:

Top Networks

ID	Associated Network Functions	Score
1	Developmental Disorder, Hereditary Disorder, Skeletal and Muscular Disorders	29
2	Reproductive System Disease, Cancer, Gastrointestinal Disease	29

(c) 2000-2013 Ingenuity Systems, Inc. All rights reserved.

3	Reproductive System Disease	3
4	Cellular Development, Cellular Growth and Proliferation, Nervous System Development and Function	3
5		3

(c) 2000-2013 Ingenuity Systems, Inc. All rights reserved.

Top Bio Functions

Diseases and Disorders

Name	p-value	# Molecules
Hereditary Disorder	6,76E-10 - 2,56E-03	6
Skeletal and Muscular Disorders	6,76E-10 - 2,55E-02	6
Developmental Disorder	1,99E-09 - 2,79E-02	6
Cancer	6,43E-08 - 4,77E-02	16
Gastrointestinal Disease	6,43E-08 - 4,77E-02	14

Molecular and Cellular Functions

Name	p-value	# Molecules
Cell Cycle	1,28E-03 - 3,39E-02	2
Cell Death and Survival	1,28E-03 - 3,78E-02	3
Cellular Assembly and Organization	7,68E-03 - 2,66E-02	1
Cellular Function and Maintenance	7,68E-03 - 2,66E-02	1
Cellular Movement	1,02E-02 - 4,89E-02	3

Physiological System Development and Function

Name	p-value	# Molecules
Embryonic Development	2,49E-03 - 1,53E-02	2
Organismal Development	2,49E-03 - 1,53E-02	2
Reproductive System Development and Function	2,49E-03 - 1,53E-02	2
Skeletal and Muscular System Development and Function	7,68E-03 - 2,66E-02	1
Tissue Morphology	7,68E-03 - 7,68E-03	1

(c) 2000-2013 Ingenuity Systems, Inc. All rights reserved.

INGENUITY® SYSTEMS

Top Canonical Pathways

Name	p-value	Ratio
Systemic Lupus Erythematosus Signaling	2,5E-01	1/246 (0,004)

Top Molecules

Fold Change up-regulated

Molecules	Exp. Value	Exp. Chart
miR-589-3p (miRNAs w/seed CAGAACA)	↑149,517	
mir-149	↑47,258	
miR-339-5p (and other miRNAs w/seed CCCUGUC)	↑19,916	
RNU6-2*	↑7,652	

Fold Change down-regulated

Molecules	Exp. Value	Exp. Chart
mir-96	↓-387,352	
miR-133a-3p (and other miRNAs w/seed UUGGUCC)	↓-111,045	
miR-200a-5p (and other miRNAs w/seed AUCUUAC)	↓-71,136	
miR-628-3p (miRNAs w/seed CUAGUAA)	↓-55,012	
mir-638	↓-47,395	
mir-657	↓-45,307	
miR-518a-3p (and other miRNAs w/seed AAAGCGC)	↓-44,554	
miR-519a-3p (and other miRNAs w/seed AAGUGCA)	↓-32,881	
mir-566	↓-29,293	
let-7	↓-27,617	

(c) 2000-2013 Ingenuity Systems, Inc. All rights reserved.

Top Upstream Regulators

(c) 2000-2013 Ingenuity Systems, Inc. All rights reserved.

Top My Lists

Name	p-value	Ratio
PreBI vs PreBII L barn_miR_utvidet mRNA_Core TFs	1,4E-02	1/16 (0,062)
PreBI vs PreBIIL_miR_adults	1,91E-02	1/18 (0,056)
PreBI vs PreBII_miR and mRNA_network2_adults	3,04E-02	1/28 (0,036)
PreBI vs PreBIIL_miRs only_adults	4,89E-02	1/51 (0,02)
PreBI vs PreBIIL_miR_and mRNA_adults	5,99E-02	1/63 (0,016)

Top My Pathways

Name	p-value	Ratio
PreBI vs PreBII L_miR og mRNA_core TF_barn	2,66E-02	1/26 (0,038)
PreBI vs PreBIIL_miRs and mRNA_voksne	6,59E-02	1/60 (0,017)

Top Tox Lists

Name	p-value	Ratio

(c) 2000-2013 Ingenuity Systems, Inc. All rights reserved.

Top Tox Functions

Cardiotoxicity

Name	p-value	# Molecules
Cardiac Hypertrophy	2,16E-02 - 1,47E-01	1
Cardiac Hypoplasia	2,79E-02 - 2,79E-02	1
Cardiac Necrosis/Cell Death	3,78E-02 - 3,78E-02	1
Cardiac Infarction	7,19E-02 - 7,19E-02	1
Cardiac Proliferation	8,97E-02 - 8,97E-02	1

Hepatotoxicity

Name	p-value	# Molecules
Liver Hyperplasia/Hyperproliferation	1,51E-04 - 2,41E-02	6
Hepatocellular Carcinoma	2,93E-03 - 2,93E-03	4

Nephrotoxicity

Name	p-value	# Molecules
Renal Inflammation	2,43E-04 - 2,43E-04	3
Renal Nephritis	2,43E-04 - 2,43E-04	3

(c) 2000-2013 Ingenuity Systems, Inc. All rights reserved.

Analysis Name: Child PreBII L vs Adult PreBII L_miR
Analysis Creation Date: 2013-06-09
Build version: 220217
Content version: 16542223 (Release Date: 2013-05-13)

Analysis settings

View

Reference set: Ingenuity Knowledge Base (Genes Only)

Relationship to include: Direct and Indirect

Includes Endogenous Chemicals

Optional Analyses: My Pathways My List

Filter Summary:

Consider only molecules and/or relationships where

(species = Rat OR Human OR Mouse) AND

(confidence = Experimentally Observed OR High (predicted))

Cutoff:

Top Networks

ID	Associated Network Functions	Score
1	Reproductive System Disease, Inflammatory Disease, Inflammatory Response	31
2	Reproductive System Disease, Cell Death and Survival, Endocrine System Disorders	25

(c) 2000-2013 Ingenuity Systems, Inc. All rights reserved.

3 Embryonic Development, Hair and Skin Development and Function, Organ Development 3
4 Cancer, Reproductive System Disease, Connective Tissue Disorders 3

(c) 2000-2013 Ingenuity Systems, Inc. All rights reserved.

Top Bio Functions

Diseases and Disorders

Name	p-value	# Molecules
Reproductive System Disease	3,06E-15 - 7,78E-04	9
Inflammatory Disease	2,08E-06 - 9,73E-03	5
Inflammatory Response	2,08E-06 - 2,08E-06	4
Renal and Urological Disease	2,08E-06 - 4,16E-04	6
Cancer	4,75E-06 - 4,85E-02	9

Molecular and Cellular Functions

Name	p-value	# Molecules
Cell-To-Cell Signaling and Interaction	9,92E-04 - 9,92E-04	1
Cellular Assembly and Organization	9,92E-04 - 9,92E-04	1
Cellular Function and Maintenance	9,92E-04 - 9,92E-04	1
Cell Cycle	9,87E-03 - 9,87E-03	1
Lipid Metabolism	2,55E-02 - 2,55E-02	1

Physiological System Development and Function

Name	p-value	# Molecules
Nervous System Development and Function	9,92E-04 - 9,92E-04	1
Tissue Development	9,92E-04 - 9,92E-04	1
Cardiovascular System Development and Function	1,98E-03 - 1,98E-03	1
Skeletal and Muscular System Development and Function	1,98E-03 - 1,98E-03	1
Tissue Morphology	1,98E-03 - 1,98E-03	1

(c) 2000-2013 Ingenuity Systems, Inc. All rights reserved.

INGENUITY® SYSTEMS

Top Canonical Pathways

Name	p-value	Ratio

Top Molecules

Fold Change up-regulated

Molecules	Exp. Value	Exp. Chart
miR-374b-3p (miRNAs w/seed UUAGCAG)	↑60,863	
miR-129-1-3p (and other miRNAs w/seed AGCCCUU)	↑27,954	
miR-551b-3p (and other miRNAs w/seed CGACCCA)	↑17,722	
miR-20b-3p (miRNAs w/seed CUGUAGU)	↑14,099	
miR-574-3p (miRNAs w/seed ACGCUCA)	↑9,126	

Fold Change down-regulated

Molecules	Exp. Value	Exp. Chart
miR-1 (and other miRNAs w/seed GGAAUGU)	↓-39,877	
miR-657 (miRNAs w/seed GCAGGUU)	↓-37,336	
miR-326-5p (and other miRNAs w/seed GGGGCAG)	↓-30,169	
miR-198 (miRNAs w/seed GUCCAGA)	↓-24,718	
miR-545-3p (miRNAs w/seed CAGCAAA)	↓-22,549	
miR-193a-3p (and other miRNAs w/seed ACUGGCC)	↓-17,119	
miR-638 (miRNAs w/seed GGGAUCG)	↓-16,912	
miR-383 (and other miRNAs w/seed GAUCAGA)	↓-15,616	
miR-495-3p (and other miRNAs w/seed AACAAAC)	↓-13,086	
miR-377-3p (miRNAs w/seed UCACACA)	↓-12,597	

(c) 2000-2013 Ingenuity Systems, Inc. All rights reserved.

INGENUITY® SYSTEMS

Top Upstream Regulators

(c) 2000-2013 Ingenuity Systems, Inc. All rights reserved.

Top My Lists

Name	p-value	Ratio
PreBI vs PreBIIL_miR_adults	1,48E-02	1/18 (0,056)
PreBI vs PreBII_miR and mRNA_network2_adults	2,35E-02	1/28 (0,036)
PreBI vs PreBIIL_miRs only_adults	3,8E-02	1/51 (0,02)
PreBI vs PreBIIL_miR_and mRNA_adults	4,66E-02	1/63 (0,016)
PreBI vs PreBII_miR target filter_cell cycle_ID2_c	4,75E-02	1/51 (0,02)

Top My Pathways

Name	p-value	Ratio
PreBI vs PreBIIL_miRs and mRNA_voksne	5,13E-02	1/60 (0,017)

Top Tox Lists

Name	p-value	Ratio

(c) 2000-2013 Ingenuity Systems, Inc. All rights reserved.

Top Tox Functions

Cardiotoxicity

Name	p-value	# Molecules
Cardiac Infarction	5,60E-02 - 5,60E-02	1
Cardiac Hypertrophy	1,15E-01 - 1,15E-01	1
Cardiac Arrythmia	1,69E-01 - 1,69E-01	1
Cardiac Arteriopathy	2,56E-01 - 2,56E-01	1

Hepatotoxicity

Name	p-value	# Molecules
Liver Hyperplasia/Hyperproliferation	2,67E-02 - 8,01E-02	3
Hepatocellular Carcinoma	8,01E-02 - 8,01E-02	2

Nephrotoxicity

Name	p-value	# Molecules
Renal Inflammation	2,08E-06 - 2,08E-06	4
Renal Nephritis	2,08E-06 - 2,08E-06	4

(c) 2000-2013 Ingenuity Systems, Inc. All rights reserved.

Analysis Name: Child PreBII s vs Adult PreBII s_miR
Analysis Creation Date: 2013-06-09
Build version: 220217
Content version: 16542223 (Release Date: 2013-05-13)

Analysis settings

View

Reference set: Ingenuity Knowledge Base (Genes + Endogenous Chemicals)

Relationship to include: Direct and Indirect

Includes Endogenous Chemicals

Optional Analyses: My Pathways My List

Filter Summary:

Consider only molecules and/or relationships where

(species = Rat OR Human OR Mouse) AND

(confidence = Experimentally Observed OR High (predicted))

Cutoff:

Top Networks

ID	Associated Network Functions	Score
1	Developmental Disorder, Hereditary Disorder, Skeletal and Muscular Disorders	31
2	Cancer, Gastrointestinal Disease, Developmental Disorder	31

(c) 2000-2013 Ingenuity Systems, Inc. All rights reserved.

3	Cancer, Gastrointestinal Disease, Cellular Development	26
4	Cellular Movement, Cancer, Reproductive System Disease	11
5	Connective Tissue Disorders, Inflammatory Disease, Inflammatory Response	2

(c) 2000-2013 Ingenuity Systems, Inc. All rights reserved.

Top Bio Functions

Diseases and Disorders

Name	p-value	# Molecules
Hereditary Disorder	1,82E-17 - 4,52E-02	12
Skeletal and Muscular Disorders	1,82E-17 - 6,29E-03	11
Endocrine System Disorders	1,14E-16 - 3,16E-02	13
Reproductive System Disease	1,14E-16 - 3,11E-02	23
Developmental Disorder	1,44E-16 - 2,29E-02	11

Molecular and Cellular Functions

Name	p-value	# Molecules
Cellular Movement	3,08E-05 - 3,18E-02	8
Cellular Development	6,40E-05 - 4,94E-02	9
Cellular Growth and Proliferation	6,40E-05 - 4,74E-02	9
Cell Death and Survival	1,13E-03 - 4,12E-02	7
Cell Cycle	6,29E-03 - 4,68E-02	4

Physiological System Development and Function

Name	p-value	# Molecules
Tumor Morphology	6,40E-05 - 4,74E-02	7
Organismal Development	1,53E-04 - 4,72E-02	5
Cardiovascular System Development and Function	2,04E-03 - 4,72E-02	5
Embryonic Development	2,10E-03 - 4,72E-02	1
Hair and Skin Development and Function	2,10E-03 - 2,10E-03	1

(c) 2000-2013 Ingenuity Systems, Inc. All rights reserved.

Top Canonical Pathways

Name	p-value	Ratio
Regulation of the Epithelial-Mesenchymal Transition Pathway	5,53E-02	2/190 (0,011)

Top Molecules

Fold Change up-regulated

Molecules	Exp. Value	Exp. Chart
mir-210	↑639,145	
miR-34a-3p (miRNAs w/seed AAUCAGC)	↑249,000	
mir-149	↑129,787	
mir-27	↑114,365	
miR-7-1-3p (and other miRNAs w/seed AACAAAU)	↑85,925	
miR-331-5p (miRNAs w/seed UAGGUAU)	↑59,714	
miR-455-3p (miRNAs w/seed CAGUCCA)	↑59,404	
mir-657	↑42,740	
miR-744-3p (miRNAs w/seed UGUUGCC)	↑36,190	
miR-148b-5p (miRNAs w/seed AGUUCUG)	↑34,836	

Fold Change down-regulated

Molecules	Exp. Value	Exp. Chart
mir-145	↓-31,724	
miR-125b-5p (and other miRNAs w/seed CCCUGAG)	↓-21,593	

Top Upstream Regulators

(c) 2000-2013 Ingenuity Systems, Inc. All rights reserved.

(c) 2000-2013 Ingenuity Systems, Inc. All rights reserved.

Top My Lists

Name	p-value	Ratio
PreBI vs PreBII L barn_miR_utvidet mRNA_Core TFs	2,29E-02	1/16 (0,062)
PreBI vs PreBIIL_miR_adults	3,11E-02	2/18 (0,111)
PreBI vs PreBII_miR and mRNA_network2_adults	4,92E-02	2/28 (0,071)
PreBI vs PreBIIL_miRs only_adults	7,88E-02	2/51 (0,039)
PreBI vs PreBIIL_miR_and mRNA_adults	9,61E-02	2/63 (0,032)

Top My Pathways

Name	p-value	Ratio
PreBI vs PreBII L_miR og mRNA_core TF_barn	4,32E-02	1/26 (0,038)
PreBI vs PreBIIL_miRs and mRNA_voksne	1,06E-01	2/60 (0,033)

Top Tox Lists

Name	p-value	Ratio
Increases Renal Proliferation	2,22E-01	1/119 (0,008)
Cardiac Hypertrophy	5,18E-01	1/344 (0,003)

(c) 2000-2013 Ingenuity Systems, Inc. All rights reserved.

INGENUITY® SYSTEMS

Top Tox Functions

Cardiotoxicity

Name	p-value	# Molecules
Congenital Heart Anomaly	2,29E-02 - 2,29E-02	1
Cardiac Fibrosis	2,15E-01 - 2,15E-01	1
Cardiac Hypertrophy	4,01E-01 - 4,01E-01	1
Heart Failure	4,06E-01 - 4,06E-01	1

Hepatotoxicity

Name	p-value	# Molecules
Liver Hyperplasia/Hyperproliferation	7,25E-08 - 5,24E-06	11
Hepatocellular Carcinoma	5,24E-06 - 5,24E-06	8
Liver Steatosis	9,23E-02 - 9,23E-02	1

Nephrotoxicity

Name	p-value	# Molecules
Renal Inflammation	1,73E-02 - 1,73E-02	2
Renal Nephritis	1,73E-02 - 1,73E-02	2
Renal Proliferation	2,97E-01 - 2,97E-01	1

(c) 2000-2013 Ingenuity Systems, Inc. All rights reserved.

Analysis Name: Child Immature B vs Adult Immature B_miR
Analysis Creation Date: 2013-06-09
Build version: 220217
Content version: 16542223 (Release Date: 2013-05-13)

Analysis settings

View

Reference set: Ingenuity Knowledge Base (Genes + Endogenous Chemicals)

Relationship to include: Direct and Indirect

Includes Endogenous Chemicals

Optional Analyses: My Pathways My List

Filter Summary:

Consider only molecules and/or relationships where

(species = Rat OR Human OR Mouse) AND

(confidence = Experimentally Observed OR High (predicted))

Cutoff:

Top Networks

ID	Associated Network Functions	Score
1	Cancer, Cellular Development, Cellular Growth and Proliferation	23
2	Hereditary Disorder, Skeletal and Muscular Disorders, Developmental Disorder	20

(c) 2000-2013 Ingenuity Systems, Inc. All rights reserved.

3	Connective Tissue Disorders, Inflammatory Disease, Inflammatory Response	15
4	Embryonic Development, Hair and Skin Development and Function, Organ Development	2
5	Developmental Disorder, Hereditary Disorder, Skeletal and Muscular Disorders	2

(c) 2000-2013 Ingenuity Systems, Inc. All rights reserved.

Top Bio Functions

Diseases and Disorders

Name	p-value	# Molecules
Reproductive System Disease	3,69E-13 - 2,48E-02	16
Hereditary Disorder	1,03E-11 - 3,08E-02	10
Skeletal and Muscular Disorders	1,03E-11 - 2,55E-02	8
Developmental Disorder	3,84E-11 - 3,08E-02	7
Endocrine System Disorders	4,47E-07 - 2,25E-02	8

Molecular and Cellular Functions

Name	p-value	# Molecules
Cell Death and Survival	1,52E-03 - 4,16E-02	4
Cell Morphology	1,52E-03 - 1,52E-03	1
Cell-To-Cell Signaling and Interaction	1,52E-03 - 4,74E-02	4
Cellular Function and Maintenance	1,52E-03 - 4,74E-02	3
Cellular Growth and Proliferation	1,52E-03 - 4,89E-02	5

Physiological System Development and Function

Name	p-value	# Molecules
Cardiovascular System Development and Function	1,52E-03 - 4,74E-02	3
Tissue Development	1,52E-03 - 3,87E-02	2
Hematological System Development and Function	9,07E-03 - 2,70E-02	3
Hematopoiesis	9,07E-03 - 2,70E-02	2
Skeletal and Muscular System Development and Function	9,07E-03 - 4,89E-02	2

(c) 2000-2013 Ingenuity Systems, Inc. All rights reserved.

INGENUITY® SYSTEMS

Top Canonical Pathways

Name	p-value	Ratio

Top Molecules

Fold Change up-regulated

Molecules	Exp. Value	Exp. Chart
miR-455-3p (miRNAs w/seed CAGUCCA)	↑52,985	

Fold Change down-regulated

Molecules	Exp. Value	Exp. Chart
miR-876-5p (and other miRNAs w/seed GGAUUUC)	↓-118,193	
miR-335-3p (miRNAs w/seed UUUUCAU)	↓-96,169	
mir-636	↓-71,630	
mir-214	↓-61,287	
mir-145	↓-47,095	
mir-489	↓-39,124	
miR-125b-5p (and other miRNAs w/seed CCCUGAG)	↓-38,854	
mir-588	↓-36,002	
miR-511 (miRNAs w/seed UGUCUUU)	↓-34,237	
MIR659	↓-27,096	

Top Upstream Regulators

(c) 2000-2013 Ingenuity Systems, Inc. All rights reserved.

INGENUITY® SYSTEMS

Top My Lists

Name	p-value	Ratio
PreBI vs PreBIIL_miRs only_adults	1,58E-03	2/51 (0,039)
PreBI vs PreBII L barn_miR_utvidet mRNA_Core TFs	1,66E-02	1/16 (0,062)
PreBI vs PreBIIL_miR_adults	2,25E-02	1/18 (0,056)
PreBI vs PreBII_miR and mRNA_network2_adults	3,58E-02	1/28 (0,036)
PreBI vs PreBIIL_miR_and mRNA_adults	7,04E-02	1/63 (0,016)

Top My Pathways

Name	p-value	Ratio
PreBI vs PreBII L_miR og mRNA_core TF_barn	3,14E-02	1/26 (0,038)
PreBI vs PreBIIL_miRs and mRNA_voksne	7,74E-02	1/60 (0,017)

Top Tox Lists

Name	p-value	Ratio

(c) 2000-2013 Ingenuity Systems, Inc. All rights reserved.

INGENUITY® SYSTEMS

Top Tox Functions

Cardiotoxicity

Name	p-value	# Molecules
Cardiac Damage	7,56E-03 - 7,56E-03	1
Cardiac Fibrosis	1,61E-01 - 1,61E-01	1

Hepatotoxicity

Name	p-value	# Molecules
Liver Hyperplasia/Hyperproliferation	4,07E-04 - 3,48E-02	6
Hepatocellular Carcinoma	3,48E-02 - 3,48E-02	3
Liver Steatosis	6,75E-02 - 6,75E-02	1

Nephrotoxicity

Name	p-value	# Molecules
Renal Inflammation	1,36E-01 - 1,36E-01	1
Renal Nephritis	1,36E-01 - 1,36E-01	1

(c) 2000-2013 Ingenuity Systems, Inc. All rights reserved.
